# Versatile cobalt-catalyzed regioselective chain-walking double hydroboration of 1,n-dienes to access *gem*-bis(boryl)alkanes

**DOI:** 10.1038/s41467-020-14543-2

**Published:** 2020-02-07

**Authors:** Ming Hu, Shaozhong Ge

**Affiliations:** Department of Chemistry, National University of Singapore, 117543 Singapore, Singapore

**Keywords:** Catalytic mechanisms, Homogeneous catalysis, Synthetic chemistry methodology

## Abstract

Double hydroboration of dienes is the addition of a hydrogen and a boryl group to the two double bonds of a diene molecule and represents a straightforward and effective protocol to prepare synthetically versatile bis(boryl)alkanes, provided that this reaction occurs selectively. However, this reaction can potentially yield several isomeric organoboron products, and it still remains a challenge to control the regioselectivity of this reaction, which allows the selective production of a single organoboron product, in particular, for a broad scope of dienes. By employing a readily available cobalt catalyst, here we show that this double hydroboration yields synthetically useful *gem*-bis(boryl)alkanes with excellent regioselectivity. In addition, the scope of dienes for this reaction is broad and encompasses a wide range of conjugated and non-conjugated dienes. Furthermore, mechanistic studies indicate that this cobalt-catalyzed double hydroboration occurs through boryl-directed chain-walking hydroboration of alkenylboronates generated from *anti*-Markovnikov 1,2-hydroboration of 1,n-diene.

## Abstract (web summary)

Control of regioselectivity in the double hydroboration of dienes to obtain a single organoboron compound is a considerable synthetic challenge. Here, the authors show a cobalt-catalyzed chain-walking double hydroboration of 1,n-dienes to access *gem*-bis(boryl)alkanes with regioselective control.

## Introduction

Organoboronates are versatile building blocks for chemical synthesis because of their diverse reactivity in organic reactions^1,2^. As an important family of organoboron compounds, *gem*-bis(boryl)alkanes have recently gained increasing attention in organic synthesis due to their unique reactivity^3–7^. For example, different reactivity of the two boryl groups in *gem*-bis(boryl)alkanes allows stepwise functionalization of their two C–B bonds^8–10^. Interestingly, *gem*-bis(boryl)alkanes can generate two types of carboanions, monoboryl- or *gem*-bis(boryl)-functionalized carboanions, via deprotonation by LiTMP (lithium tetramethylpiperidide) or alkoxide-induced deborylation^11,12^. Over past decades, series of catalytic reactions have been developed to access these *gem*-bis(boryl) compounds^13–17^, such as double hydroboration of alkynes^18–20^, diborylation of alkenes^21,22^, hydroboration of 1-borylalkene^23–25^, or C–H borylation reactions^26–29^. Nevertheless, a general and practical approach that combines high catalytic activity, easy accessibility and handling of starting materials, and structural diversity of *gem*-bis(boryl) products is still lacking.

Metal-catalyzed hydroboration of 1,3-dienes has been developed into selective approaches to prepare allylic or homoallylic organoboron compounds^30–35^. However, double hydroboration of 1,3-dienes has been barely studied, mainly because alkenylboron products from the first hydroboration contain an unactivated internal alkene that does not readily undergo the second hydroboration. There was only one early example of rhodium-catalyzed double hydroboration of 1,3-dienes reported by Hayashi, and this reaction selectively afforded 1,3-bis(boryl)alkane products^36^. Catalysts for selective double hydroboration of 1,n-dienes to synthesize *gem*-bis(boryl)alkanes still remains unknown.

**Fig. 1:**
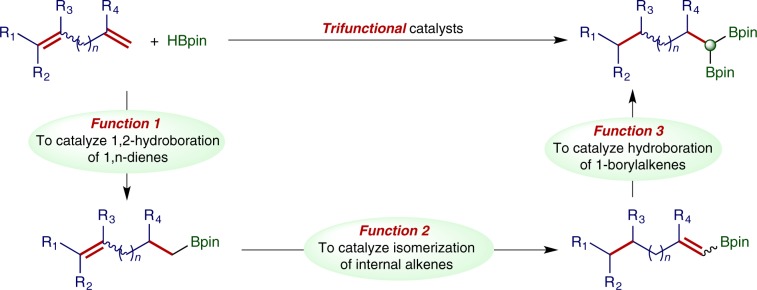
Hydroboration of dienes. Trifunctional catalysts for chain-walking double hydroboration of 1,n-dienes.

Recently, metal-mediated chain walking has been emerging as a useful tool for remote functionalization of organic molecules^37–54^. The directions for chain walking can be controlled by catalysts or directing groups in organic substrates. For example, aryl or boryl groups in the substrates can direct the chain walking towards them^46,48,51,54^. Therefore, double hydroboration of 1,n-dienes can be potentially developed into an effective and selective protocol to prepare *gem*-bis(boryl)alkanes, provided that a trifunctional catalyst can be identified to catalyze these three transformations: selective hydroboration of 1,n-dienes to produce alkenylboronates, the isomerization of alkenylboronates to 1-borylalkenes, and the subsequent hydroboration of 1-borylalkenes to yield *gem*-bis(boryl)alkanes (Fig. 1).

In recent years, cobalt compounds have been extensively studied as catalysts for hydroboration and isomerization of alkenes^55–63^. In 2015, Chirik and coworkers showed one example of cobalt-catalyzed hydroboration of a boryl-containing terminal alkene to give a *gem*-bis(boryl)alkane product by taking the advantage of boryl-directed alkene isomerization^59^. During our continuous efforts in developing cobalt-catalyzed hydroboration of unsaturated hydrocarbons^64–66^, we become interested in identifying a selective cobalt catalyst for double hydroboration of 1,n-dienes to synthesize *gem*-bis(boryl)alkanes. We envisioned that it would be more challenging to develop a selective double hydroboration of aryl-substituted 1,n-dienes because both aryl and boryl groups in alkenylboronate products of the first hydroboration can control the direction of subsequent alkene isomerization, which would probably decrease the selectivity for the second hydroboration. Indeed, such decreased selectivity has been encountered in a recent study on NiH-catalyzed remote hydroarylation of a phenyl-containing alkenylboronate^54^. Here, we show that double hydroboration of these 1,n-dienes takes place to yield synthetically versatile *gem*-bis(boryl)alkanes with high regioselectivity in the presence of Co(acac)_2_ and 1,2-bis(dicyclohexylphosphino)ethane (dcpe).

## Results

### Evaluation of reaction conditions

**Fig. 2:**
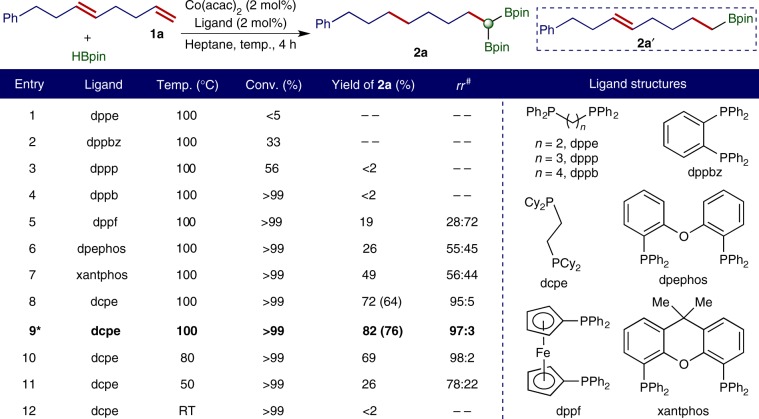
Evaluation of conditions for cobalt-catalyzed double hydroboration of 1,5-diene **1a**. Reaction conditions: **1a** (0.200 mmol), HBpin (0.500 mmol), Co(acac)_2_ (4.0 μmol), ligand (4.0 μmol), heptane (0.5 mL), 4 h, yield was determined by GC (gas chromatography) analysis using tridecane as internal standard and yield in parentheses was isolated yield of **2a**. ^#^*rr* is regioisomeric ratio and represents the ratio of the desired *gem*-bis(boryl)alkane product to the sum of all other bis(boryl)alkane isomers as determined by gas chromatography analysis.*3 mol % cobalt catalyst.

We chose the reaction of (*E*)-octa-3,7-dien-1-ylbenzene (**1a**) with HBpin to identify a cobalt catalyst and the conditions that favor the formation of the *gem*-bis(boryl)alkane product **2a** (Fig. 2). The cobalt catalysts we intended to evaluate were generated in situ from Co(acac)_2_ and bisphosphine ligands and activated by the reaction with HBpin. In general, the targeted double hydroboration reactions were conducted with 2 mol% cobalt catalyst in the presence of 2.5 equivalents of HBpin for 4 h at 100 °C. The selected examples of these reactions are summarized in Fig. 2.

The reaction conducted with Co(acac)_2_ and dppe proceeded sluggishly to very low conversion (<5%) of **1a** and the desired product **2a** was not formed (Fig. 2, entry 1). The reaction catalyzed by Co(acac)_2_ and dppbz occurred to a low conversion (33%) of **1a** and only a trace amount of **2a** was detected together with several other isomeric 1,n-dienes that were resulted from the isomerization of **1a** (Fig. 2, entry 2). The reactions of **1a** with HBpin afforded alkenylboronate **2a′** as a major product when conducted with Co(acac)_2_ and dppp or dppb ligand (Fig. 2, entries 3 and 4). The reactions run with Co(acac)_2_ and dppf, dpephos, or xantphos afforded eight isomeric bis(boryl)alkane products (Fig. 2, entries 5−7), and the selectivity for the desired product **2a** was only low to modest (28−56%). To our delight, the reaction catalyzed by 2 mol % Co(acac)_2_/dcpe produced **2a** in good yield (72%) and high regioselectivity (95% rr, Fig. 2, entry 8). In particular, the reaction conducted with 3 mol% catalyst afforded **2a** in high isolated yield (76%) with excellent regioselectivity (97% rr, Fig. 2, entry 9). We also tested various temperatures for this double hydroboration reaction catalyzed by Co(acac)_2_/dcpe. Similar results were obtained for the reactions run at 100 °C and 80 °C (Fig. 2, entries 8 and 10). Further lowering the temperature to 50 °C led to a lower yield of **2a** with a diminished regioselectivity (Fig. 2, entry 11). Particularly, the reaction performed at room temperature afforded only the alkenylboronate product **2a′**, which was formed from hydroboration of the terminal double bond of **1a** (Fig. 2, entry 12).

### Substrate scope of conjugated and non-conjugated 1,n-dienes

**Fig. 3:**
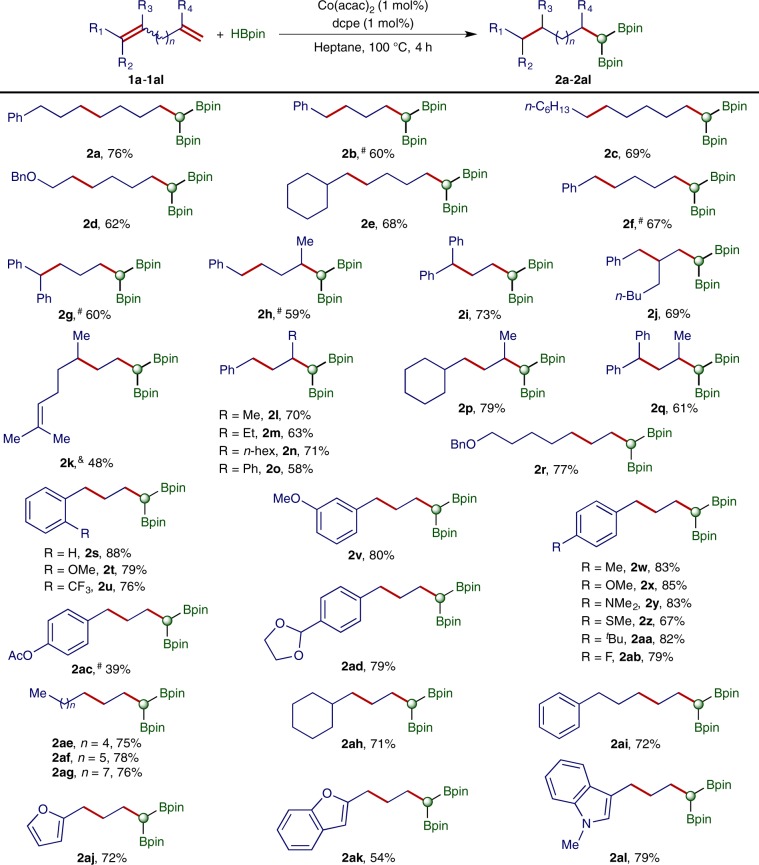
Scope of 1,n-dienes. Reaction conditions: 1,n-diene (0.300 mmol), HBpin (0.750 mmol), Co(acac)_2_ (3.0 μmol), dcpe (3.0 μmol), heptane (0.5 mL), 100 °C, 4 h, and isolated yields. ^#^3 mol % catalyst; ^&^80 °C.

With the identified cobalt catalyst and reliable conditions in hand, we studied the scope of 1,n-dienes that undergo this cobalt-catalyzed double hydroboration for the synthesis of *gem*-bis(boryl)alkanes, and the results are summarized in Fig. 3. In general, a wide range of non-conjugated (*Z*/*E*)−1,n-dienes (**1a**−**1h**) and conjugated (*Z*/*E*)−1,3-dienes (**1i**−**1al**) smoothly reacted with HBpin in the presence of 1 mol% Co(acac)_2_ and 1 mol% dcpe, yielding the corresponding *gem*-bis(boryl)alkanes (**2a**−**2al**) in modest to high isolated yields (58−88%) with excellent regioselectivity (up to 99%). Noticeably, carbocyclic products resulted from cyclization of 1,6-diene **1c** were not detected.

The scope of 1,n-dienes encompasses both alkyl- and aryl-substituted 1,n-dienes with substituents at various positions (e.g. **1g**−**1q**). Particularly, the double hydroboration of 1,n-dienes (**1h** and **1l**−**1q**) containing 1,1-disubstituted alkene units afforded β-branched *gem*-bis(boryl)alkanes (**2h** and **2l**−**2q**), which are not accessible via double hydroboration of terminal alkynes. In addition, 1,3-dienes containing *ortho*-substituted aryl groups also smoothly reacted under identified conditions to afforded the corresponding *gem*-bis(boryl)alkanes (**2t** and **2u**) in high isolated yields. This cobalt-catalyzed double hydroboration can tolerate a range of functional groups, such as ether (**2d** and **2v**), tertiary amine (**2y**), thioether (**2z**), fluoride (**2ab**), carboxylic ester (**2ac**), and acetal (**2ad**). Nitrogen- and oxygen-containing 1,3-dienes also reacted to afford the desired products (**2aj**−**2al**) in high isolated yields.

**Fig. 4:**
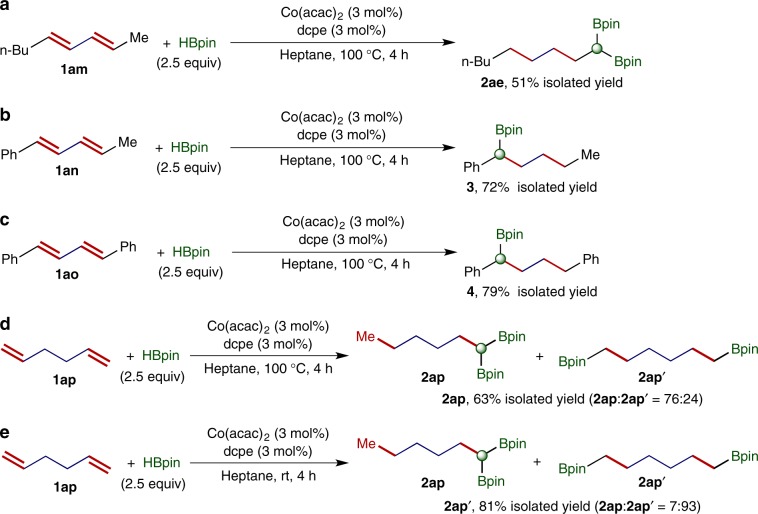
Hydroboration of dienes containing two internal double bonds or two terminal double bonds. **a** Double hydroboration of diene **1am**. **b** Sequential hydroboration/hydrogenation of diene **1an**. **c** Sequential hydroboration/hydrogenation of diene **1ao**. **d** Double hydroboration of diene **1ap** at 100 °C. **e** Hydroboration of diene **1ap** at room temperature.

In addition, we also tested this cobalt catalyst for hydroboration reactions of dienes containing two internal or two terminal carbon-carbon double bonds, and the results are shown in Fig. 4. Under standard conditions, bis(alkyl)-substituted 1,3-diene **1am** reacted smoothly with HBpin to afford *gem*-bis(boryl)alkane product **2ae** in 51% isolated yield (Fig. 4a). Product **2ae** contains two Bpin groups on the terminal sp^3^-carbon, indicating the isomerization of both internal double bonds to terminal double bonds. The hydroboration reactions of aryl,alkyl- or aryl,aryl-disubstituted 1,3-dienes **1an** and **1ao** produced benzylic boronates **3** and **4**, respectively, in high yields (Fig. 4b, c). These benzylic boronate products were formed by cobalt-catalyzed sequential hydroboration and hydrogenation of **1an** and **1ao**. The double hydroboration of 1,5-hexadiene **1ap**, a diene containing two terminal double bonds, yielded two bis(boryl)alkane products, 1,1-bis(boryl)hexane **2ap** and 1,6-bis(boryl)hexane **2ap′**, and the ratios of these two products were temperature dependent. For example, the reaction conducted at 100 °C afforded 1,1-bis(boryl)hexane **2ap** as the major product with **2ap**:**2ap′** of 76:24, and *gem*-bis(boryl)alkane product **2ap** was isolated in 63% yield (Fig. 4d). However, the corresponding reaction run at room temperature gave 1,6-bis(boryl)hexane **2ap’** in 81% isolated yield with high regioselectivity (**2ap**:**2ap′** = 7:93, Fig. 4e).

### Synthetic utilities

**Fig. 5:**
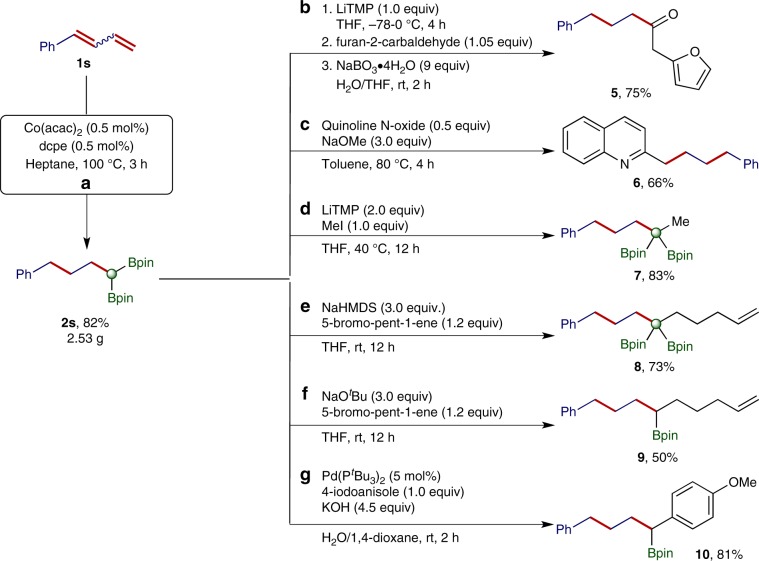
Gram-scale synthesis of *gem*-bis(boryl)alkane 2s and its further transformations. **a** Gram-scale synthesis of **2s**. **b** Synthesis of **5**. **c** Synthesis of **6**. **d** Synthesis of **7**. **e** Synthesis of **8**. **f** Synthesis of **9**. **g** Synthesis of **10**.

This cobalt-catalyzed chain-walking double hydroboration of 1,n-dienes can be conducted on gram-scales with a decreased catalyst loading. For example, the reaction of (*Z*/*E*)-buta-1,3-dien-1-ylbenzene **1s** (*Z*:*E* = 45:55) with HBpin on a 8.0-mmol scale proceeded to full conversion of **1s** in the presence of 0.5 mol% Co(acac)_2_/dcpe and afforded the desired product **2s** (2.53 g) in 82% isolated yield (Fig. 5a). We subsequently show the utility of *gem*-Bis(boryl)alkane **2s** as a versatile building block in organic synthesis by conducting a series of organic transformations with **2s** (Fig. 5b−f). For example, **2s** could undergo a Boron-Wittig reaction with an aldehyde to afford ketone **5** after oxidative workup with NaBO_3_•4H_2_O (ref.^10^) (Fig. 5b). **2s** could also be used as an alkylating reagent for the alkylation of quinoline N-oxide to prepare 2-alkylquinoline **6** (ref.^67^) (Fig. 5c). In addition, the carbanion generated by the deprotonation of **2s** with LiTMP, NaHMDS, or NaO^*t*^Bu reacted with iodomethane or 5-bromopent-1-ene to afford boryl-functionalized alkenes **7, 8,** and **9**, respectively^11^ (Fig. 5d–f). Furthermore, *gem*-bis(boryl)alkane **2** **s** readily underwent Pd-catalyzed Suzuki-Miyaura coupling with 4-iodoanisole to give alkylboronate **10** in 81% isolated yield (Fig. 5g).

## Discussion

**Fig. 6:**
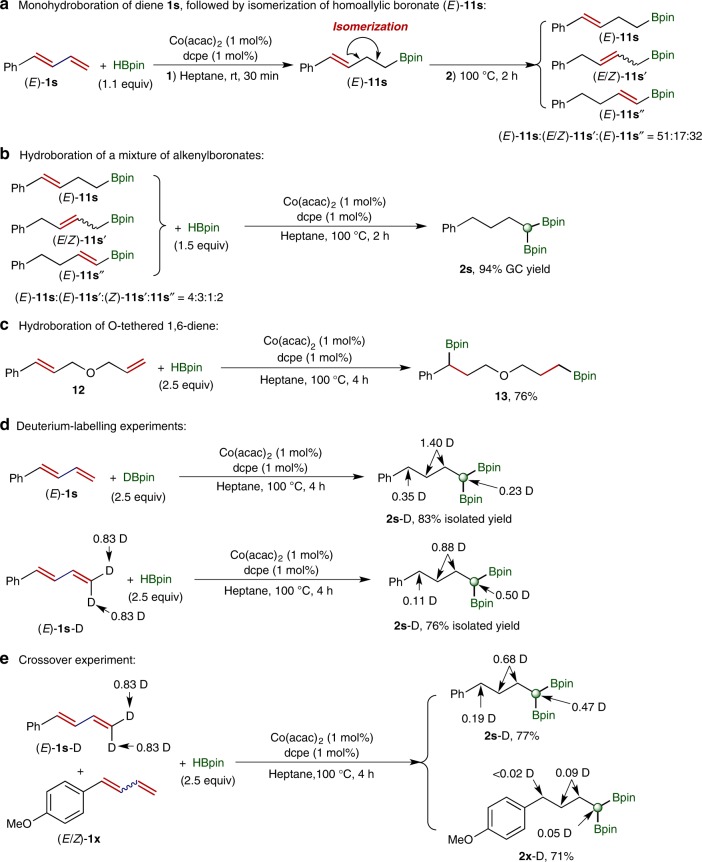
Deuterium-labeling experiment and control experiments. a Monohydroboration of diene 1s and isomerization of alkenylboronate (*E*)-11s. **b** Hydroboration of a mixture of alkenylboronates. **c** Hydroboration of O-tethered 1,6-diene **12**. **d** Deuterium-labeling experiments. **e** Crossover experiment.

We subsequently conducted a series of experiments to gain insights into the mechanism of this cobalt-catalyzed double hydroboration reactions of 1,n-dienes, and the results of these experiments are summarized in Fig. 6. Similar to monohydroboration of 1,5-diene **1a** at room temperature (Fig. 2, entry 12), monohydroboration of 1,3-diene **1s** with 1.1 equiv. of HBpin occurred to completion in 30 min at room temperature and afforded alkenylboronate **11s** selectively (Fig. 6a). Subsequent heating the reaction mixture at 100 °C for 2 h resulted in the isomerization of **11s** to a mixture of alkenylboronates **11s**, **11s′**, and **11s″** with a ratio of 51:17:32 (Fig. 6a). We then tested a mixture of these alkenylboronates for hydroboration with HBpin in the presence of 1 mol % of Co(acac)_2_/dcpe, and all these isomeric alkenylboronates were converted to *gem*-bis(boryl)alkane **2s** in high yield (Fig. 6b). The conversion of **11s**, **11s′**, and **11s″** to a single product **2s** indicates that boryl group has a stronger directing ability for this cobalt-catalyzed chain-walking hydroboration than phenyl group, which may stem from the interaction of the *d*-electrons of the cobalt catalyst with the empty *p*-orbital on boron^68^.

A control experiment of this double hydroboration was conducted with a substrate containing an oxygen-tethered 1,6-diene **12** (Fig. 6c). This reaction afforded 1,7-bis(boryl)alkane **13** and chain-walking double hydroboration was not observed. This suggests that the chain walking takes place through reversible β-hydrogen elimination and reinsertion steps. A deuterium-labeling experiment of the double hydroboration of 1,3-diene (*E*)-**1s** was then conducted with DBpin, and deuterium incorporation at all positions of the aliphatic chain of *gem*-bis(boryl)alkane **2s**-D was observed (Fig. 6d). When the deuterium-labeling experiment was performed with (*E*)-**1s**-D and HBpin, deuterium scrambling at all positions of the aliphatic chain of **2s**-D was observed as well (Fig. 6d). In addition, when a crossover experiment of this double hydroboration was run with (*E*)-**1s**-D and (*E*/*Z*)-**1×**, similar deuterium incorporation and deuterium scrambling map was also observed for *gem*-bis(boryl)alkane products **2s**-D and **2×**-D (Fig. 6e). The results of this crossover experiment indicate that dissociation and re-association of Co-H/D from the Co-H/D-olefin complex occurs during the chain walking process.

**Fig. 7:**
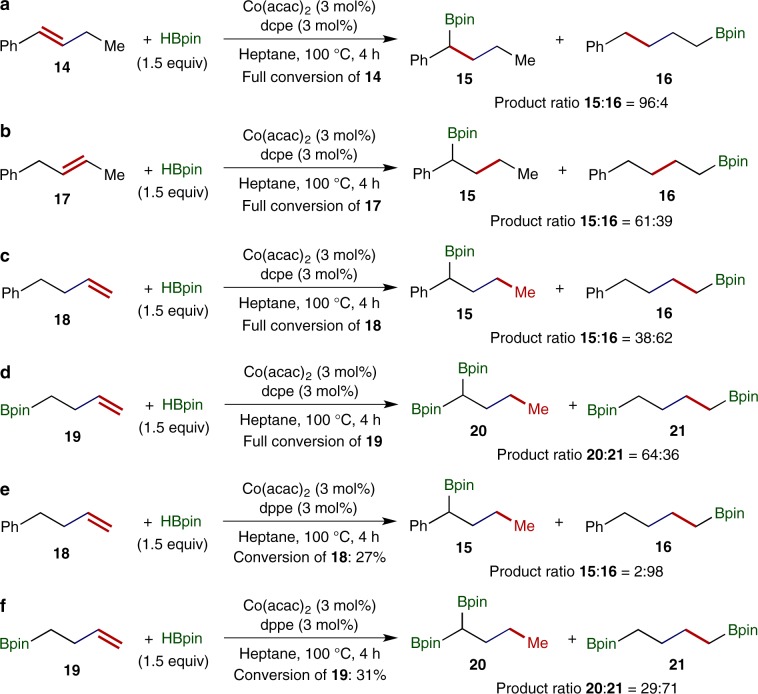
Chaining-walking hydroboration of phenyl- and boryl-containing alkenes. **a** Hydroboration of alkene **14**. **b** Hydroboration of alkene **17**. **c** Hydroboration of alkene **18**. **d** Hydroboration of alkene **19**. **e** Hydroboration of alkene **18** catalyzed by Co(acac)_2_/dppe. **f** Hydroboration of alkene **19** catalyzed by Co(acac)_2_/dppe.

We then tested this cobalt catalyst, Co(acac)_2_/dcpe, for chain-walking hydroboration of phenyl- and boryl-containing alkenes with double bonds at various positions, and the results are summarized in Fig. 7. (*E*)-But-1-en-1-ylbenzene (**14**) reacted smoothly with 1.5 equivalents HBpin in the presence of 3 mol% Co(acac)_2_/dcpe at 100 °C to give alkylboroante **15** as a major product together with a trace amount (4%) of alkylboroante **16**, a byproduct from chain-walking hydroboration (Fig. 7a). Similarly, the reaction between (*E*)-but-2-en-1-ylbenzene (**17**) and HBpin under standard conditions afforded alkylboroante products **15** and **16**, albeit with a low regioselectivity (**15**:**16** = 61:39), and both products were resulted from chain-walking hydroboration (Fig. 7b). But-3-en-1-ylbenzene (**18**) also underwent this cobalt-catalyzed chain-walking hydroboration, but the major product (**16**) of this reaction was from the hydroboration of terminal double bond of **18** (Fig. 7c). For comparison, we also conducted the reaction between a boryl-containing terminal alkene **19** and HBpin under standard conditions (Fig. 7d), and the major product, *gem*-bis(boryl)alkane **20**, was formed by the cobalt-catalyzed chain-walking hydroboration. The results of chain-walking hydrobration reactions conducted with but-3-en-1-ylbenzene (**18**) and but-3-en-1-ylboronic pinacol ester (**19**) indicate that the Bpin group has a stronger directing ability for chain-walking hydroboration than the phenyl group.

In addition, we also tested a cobalt catalyst generated from Co(acac)_2_ and dppe (1,2-bis(diphenylphosphino)ethane), a bisphosphine ligand having a similar steric but different electronic property, for hydroboration of terminal alkenes **18** and **19** with HBpin (Fig. 7e and f). These two reactions proceeded very sluggishly and approximately 30% of **18** and **19** were converted at 100 °C in 24 h. Interestingly, major products (**16** and **21**) of these two reactions were resulted from direct *anti*-Markovnikov hydroboration of **18** and **19**, and much less byproducts (**15** and **20**) were formed by chain-walking hydroboration relative to the corresponding reactions catalyzed by the cobalt catalyst generated from Co(acac)_2_ and dcpe (Fig. 7c, d). The results of these four reactions (Fig. 7c–f) suggest that the electron-rich property of dcpe ligand facilitates the chain-walking process, thus promoting chain-walking hydroboration.

**Fig. 8:**
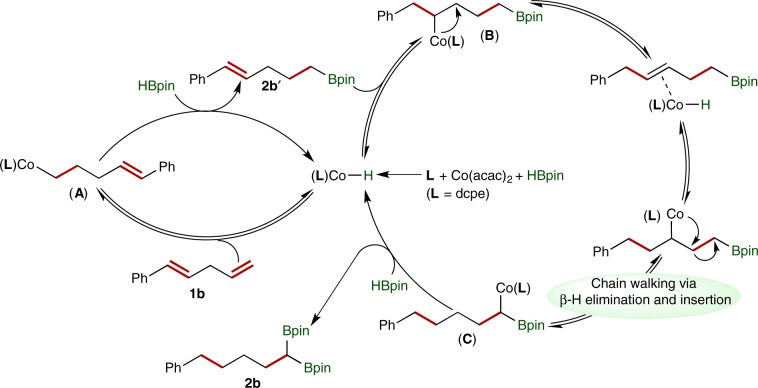
Proposed mechanism. The proposed catalytic cycle for this cobalt-catalyzed chain-walking double hydroboration.

Based on the results of mechanistic studies, we proposed a catalytic pathway for this cobalt-catalyzed chain-walking double hydroboration of 1,4-diene **1b**, as depicted in Fig. 8. The activation of Co(acac)_2_ with HBpin in the presence of dcpe (**L**) forms a cobalt hydride species (**L**)Co-H (ref. ^69^). Migratory insertion of the terminal alkene of **1b** into (**L**)Co-H generates an alkenylcobalt intermediate **A**, which then undergoes σ-bond metathesis with HBpin to produce alkenylboronate **2b′** and regenerates (**L**)Co-H. The double bond in **2b′** then inserts into (**L**)Co-H to form an alkylcobalt species **B**, which undergoes isomerization to form the alkylcobalt intermediate **C** through reversible β-hydrogen elimination and reinsertion. In the last step, σ-bond metathesis between alkylcobalt species **C** and HBpin yields *gem*-bis(boryl)alkane **2b** and regenerates the catalytically active (**L**)Co-H intermediate.

In summary, we have developed an effective and convenient protocol to prepare *gem*-bis(boryl)alkanes via a selective cobalt-catalyzed double hydroboration of 1,n-dienes. A wide range of conjugated and non-conjugated 1,n-dienes reacted with pinacolborane to produce *gem*-bis(boryl)alkanes in high isolated yields with excellent regioselectivity in the presence of a catalyst generated in situ from Co(acac)_2_ and dcpe ligand. Mechanistic studies suggest that this cobalt-catalyzed double hydroboration occurs through an initial *anti*-Markovnikov monohydroboration of 1,n-dienes followed by a sequential boryl-directed chain-walking hydroboration of the resulting alkenylboronates. This cobalt-catalyzed double hydroboration provides a straightforward approach to access structurally diverse and synthetically versatile *gem*-bis(boryl)alkanes from readily available 1,n-dienes.

## Methods

### General procedure for double hydroboration of 1,n-dienes

In an Argon-filled glovebox, a 4-mL screw-capped vial was charged with Co(acac)_2_ (0.8 mg, 3.0 µmol), dcpe (1.3 mg, 3.0 μmol), 1,n-diene (0.30 mmol), heptane (0.5 mL) and a magnetic stirring bar. The solution was stirred for 5 min and pincolborane (96.0 mg, 0.75 mmol) was added to the vial. The vial was sealed with a cap containing a PTFE septum and removed from the glovebox. The mixture was then heated at 100 ^°^C for 4 h until complete consumption of starting material as monitored by TLC and GC-MS analysis. Subsequently, the solvent was removed under reduced pressure. The residue was purified by silica gel flash column chromatography (hexane/ethyl acetate = 40:1) to afford the desired products. See the Supplementary Information for detailed experimental procedures and the characterization data of all the products.

## Supplementary information

Supplementary Information

## Data Availability

The authors declare that all data supporting the findings of this study are available within the article and Supplementary Information files, and also are available from the corresponding author upon reasonable request.
