# Supplementary material for: Versatile cobalt-catalyzed regioselective chain-walking double hydroboration of 1,n-dienes to access gem-bis(boryl)alkanes
Source: Nat Commun. 2020 Feb 7;11:765. doi: 10.1038/s41467-020-14543-2 (PMC7005816; doi:10.1038/s41467-020-14543-2)
Supplement: Supplementary file 1 — Supplementary Information [file 41467_2020_14543_MOESM1_ESM.pdf]

Supplementary Information

Versatile Cobalt-Catalyzed Regioselective Chain-Walking Double Hydroboration of 1,n-Dienes to Access *gem*-Bis(boryl)alkanes

Hu et al

Supplementary methods

All manipulations were performed in an Ar-filled glovebox, unless mentioned otherwise. THF, toluene, and hexane were purified by passing the degassed solvents (N_2) through a column of activated alumina (solvent purification system purchased from Innovative Technologies, Newburyport, MA). The following chemicals were purchased and used as received: Cobalt(II) acetylacetonate (99%, Sigma-Aldrich), pinacolborane (98%, Oakwood Chemical). All bisphosphine ligands were purchase from Sigma-Aldrich in high purity (>98.0%, Sigma-Aldrich). DBpin was synthesized based on the literature.[1] All other reagents and solvents were purchased from commercial sources and used without purification.

^{1}H and ^{13}C{^{1}H} spectra were recorded using Bruker 400 MHz, or 500 MHz NMR spectrometersand done in $CDCl_3$ unless otherwise staed. ^{1}H NMR and ^{13}C{^{1}H} NMR spectra were referenced to resonances of the residual signals of the deuterated solvents. Multiplicities are recorded as: s = singlet, d = doublet, t = triplet, dd = doublet of doublets, dt = doublet of triplets and m = multiplet. GC analysis was acquired on Agilent 6850 gas chromatograph equipped with a flame-ionization detector. HR-MS analyses were performed using Agilent GC-QTOF. GC-MS analysis was performed on Shimadzu GC-2010 gas chromatograph coupled to a Shimadzu QP2010 mass selective detector. HR-MS analyses were performed using Bruker micrOTOFQII (ESI).

Preparation of substrates 1,n-dienes:

1,n-Diene Substrates were prepared according to literature procedures.[2-6]

(i) Synthesis of 1,3-dienes

The ketone or aldehyde (5.0 mol) was dissolved in THF (3 mL) and slowly added to a stirred mixture of allyltriphenylphosphonium bromide (1.5 equiv, 7.5 mmol) and potassium *tert*-butoxide (1.5 equiv, 7.5 mmol) in THF (10 mL) at 0 °C. The mixture was warmed to room temperature and stirred for 2 hours. After the reaction was finished, the reaction mixture was washed with brine. The aqueous phase was extracted with EtOAc (3 × 10 mL). The combined organic extracts were dried over Na_2SO_4 and concentrated in vacuum. The residue was purified by flash column chromatography silica gel (hexane as an eluent) to attain the 1,3-dienes.

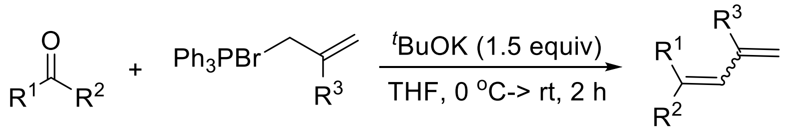

Supplementary Equation 1. Synthesis of 1,3-dienes

(ii) Synthesis of 1,n-dienes: as described for 1,3-dienes

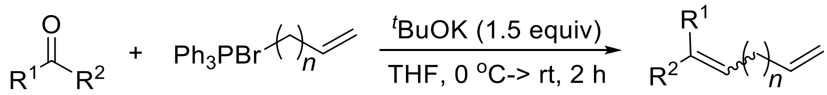

Supplementary Equation 2. Synthesis of 1,n-dienes

General procedures for cobalt-catalyzed dihydroboration of 1,n-dienes:

In an Ar-filled glovebox, a 4-mL screw-capped vial was charged with $Co(acac)_2$ (0.8 mg, 3.0 μmol), dcpe (1.3 mg, 3.0 μmol), 1,n-diene (0.30 mmol), heptane (0.5 mL) and a magnetic stirring bar. The solution was stirred for 5 min and pincolborane (96.0 mg, 0.75 mmol) was added to the vial. The vial was sealed with a cap

containing a PTFE septum and removed from the glovebox. The reaction was allowed to stir at 100 °C for 4 h until complete consumption of starting material as monitored by TLC and GC-MS analysis. Subsequently, the solvent was removed under reduced pressure. The residue was purified by silica gel flash column chromatography (hexane/ethyl acetate = 40:1) to afford the desired products **2**. The characterization data for these gem-bis(boryl)alkane products were listed in the following section.

2,2'-(8-Phenyloctane-1,1-diyl)bis(4,4,5,5-tetramethyl-1,3,2-dioxaborolane) (2a):

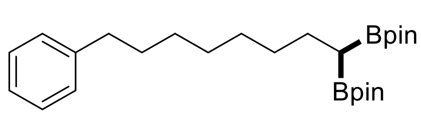

Colorless oil (101 mg, 76% yield); ^{1}H NMR (500 MHz, $CDCl_3$) δ: 7.26 (t, *J* = 7.5 Hz, 2H), 7.17-7.14 (m, 3H), 2.58 (t, *J* = 8.0 Hz, 2H), 1.60-1.52 (m, 4H), 1.35-1.26 (m, 8H), 1.23 (s, 12H), 1.22 (s, 12H), 0.71 (t, *J* = 8.0 Hz, 1H); ^{13}C{^{1}H} NMR (126 MHz, $CDCl_3$) δ: 143.0, 128.4, 128.2, 125.5, 82.7, 36.0, 32.5, 31.5, 29.5, 29.4, 29.3, 25.7, 24.8, 24.5; The boron-bound carbon was not detected due to quadrupolar relaxation. HRMS *m/z* (ESI) calcd for $C_{26}H_{44}B_2NaO_4$ $(M+Na)^+$: 465.3318; Found: 465.3332.

(*E*)-4,4,5,5-Tetramethyl-2-(8-phenyloct-5-en-1-yl)-1,3,2-dioxaborolane (2a′):

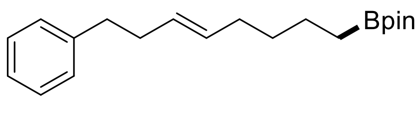

Colorless oil; ^{1}H NMR (500 MHz, $CDCl_3$) δ: 7.29-7.25 (m, 2H), 7.21-7.16 (m, 3H), 5.42-5.36 (m, 2H), 2.65 (t, *J* = 8.0 Hz, 2H), 2.37-2.33 (m, 2H), 2.01-1.97 (m, 2H), 1.43-1.36 (m, 2H), 1.34-1.28 (m, 2H), 1.24 (s, 12H), 0.76 (t, *J* = 8.0 Hz, 1H); ^{13}C{^{1}H} NMR (126 MHz, $CDCl_3$) δ: 142.2, 130.7, 128.6, 128.4, 128.2, 125.7, 82.9, 36.0, 32.4, 29.1, 27.1, 24.8, 23.7; The boron-bound carbon was not detected due to quadrupolar relaxation. GCMS *m/z* (EI) calcd for $C_{20}H_{31}BO_2$ $(M)^+$ 314.24, found 314.30.

2,2'-(5-Phenylpentane-1,1-diyl)bis(4,4,5,5-tetramethyl-1,3,2-dioxaborolane) (2b):

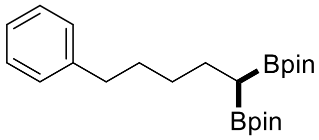

Colorless oil (72.0 mg, 60% yield); ^{1}H NMR (500 MHz, $CDCl_3$) δ: 7.26-7.23 (m, 2H), 7.17-7.11 (m, 3H), 2.59 (t, *J* = 8.0 Hz, 2H), 1.64-1.56 (m, 4H), 1.35-1.30 (m, 2H), 1.22 (s, 12H), 1.20 (s, 12H), 0.72 (t, *J* = 8.0 Hz, 1H); ^{13}C{^{1}H} NMR (126 MHz, $CDCl_3$) δ: 142.9, 128.4, 128.1, 125.4, 82.9, 35.8, 32.1, 31.3, 25.5, 24.8, 24.5; The boron-bound carbon was not detected due to quadrupolar relaxation. HRMS *m/z* (ESI) calcd for $C_{23}H_{38}B_2NaO_4$ $(M+Na)^+$: 423.2848; Found: 423.2861.

2,2'-(Tridecane-1,1-diyl)bis(4,4,5,5-tetramethyl-1,3,2-dioxaborolane) (2c):

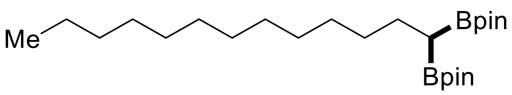

Colorless oil (90.3 mg, 69% yield); ^{1}H NMR (500 MHz, $CDCl_3$) δ: 1.53-1.48 (m, 2H), 1.26-1.19 (m, 44H), 0.85 (t, *J* = 7.0 Hz, 3H), 0.68 (t, *J* = 8.0 Hz, 1H); ^{13}C{^{1}H} NMR (126 MHz, $CDCl_3$) δ: 82.8, 32.5, 31.9, 29.7, 29.6, 29.5 (2C), 29.3, 25.6, 24.8, 24.7, 24.4, 22.6, 14.0; The boron-bound carbon was not detected due to quadrupolar relaxation. HRMS *m/z* (ESI) calcd for $C_{25}H_{50}B_2NaO_4$ $(M+Na)^+$: 459.3787; Found: 459.3799.

2,2'-(7-(Benzyloxy)heptane-1,1-diyl)bis(4,4,5,5-tetramethyl-1,3,2-dioxaborolane) (2d):

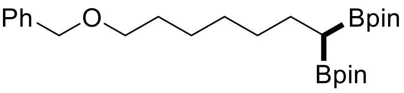

Colorless oil (85.2 mg, 62% yield); ^{1}H NMR (500 MHz, $CDCl_3$) δ: 7.35-7.32 (m, 4H), 7.29-7.25 (m, 1H), 4.49 (s, 2H), 3.44 (t, *J* = 7.0 Hz, 1H), 1.62-1.57 (m, 2H), 1.56-1.51 (s, 2H), 1.35-1.25 (m, 6H), 1.22 (s, 12H), 1.21 (s, 12H), 0.70 (t, *J* = 8.0 Hz, 1H); ^{13}C{^{1}H} NMR

(126 MHz, $CDCl_3$) δ: 138.7, 128.3, 127.6, 127.4, 82.7, 72.8, 70.6, 32.5, 29.7, 29.5, 26.1, 25.6, 24.8, 24.5; The boron-bound carbon was not detected due to quadrupolar relaxation. GCMS *m/z* (EI) calcd for $C_{26}H_{44}B_2O_5$ $(M)^+$: 458.34; Found: 458.30.

2,2'-(6-Cyclohexylhexane-1,1-diyl)bis(4,4,5,5-tetramethyl-1,3,2-dioxaborolane) (2e):

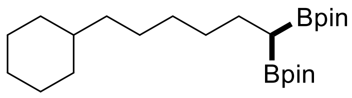

Colorless oil (85.7 mg, 68% yield); ^1H NMR (500 MHz, $CDCl_3$) δ: 1.67-1.60 (m, 5H), 1.54-1.50 (m, 2H), 1.23-1.07 (m, 36H), 0.87-0.79 (m, 2H), 0.70 (t, J = 8.0 Hz, 1H); $^{13}C\{^1H\}$ NMR (126 MHz, $CDCl_3$) δ: 82.8, 37.6, 37.4, 33.4 (Two signals overlap), 32.6, 29.9, 26.8, 26.7, 26.4 (Two signals overlap), 25.7, 24.8 (Two signals overlap), 24.5; The boron-bound carbon was not detected due to quadrupolar relaxation. GCMS *m/z* (EI) calcd for $C_{24}H_{46}B_2O_4$ $(M)^+$: 420.36; Found: 420.30.

2,2'-(6-Phenylhexane-1,1-diyl)bis(4,4,5,5-tetramethyl-1,3,2-dioxaborolane) (2f):

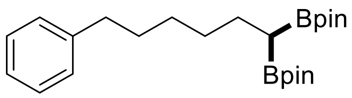

Colorless oil (83.2 mg, 67% yield); ^1H NMR (500 MHz, $CDCl_3$) δ: 7.27-7.23 (m, 2H), 7.17-7.11 (m, 3H), 2.56 (t, J = 8.0 Hz, 2H), 1.63-1.53 (m, 4H), 1.34-1.31 (m, 4H), 1.23 (s, 12H), 1.22 (s, 12H), 0.72 (t, J = 7.5 Hz, 1H); $^{13}C\{^1H\}$ NMR (126 MHz, $CDCl_3$) δ: 143.0, 128.4, 128.1, 125.4, 82.9, 35.9, 32.3, 31.4, 29.3, 25.6, 24.8, 24.5; The boron-bound carbon was not detected due to quadrupolar relaxation. GCMS *m/z* (EI) calcd for $C_{24}H_{46}B_2O_4$ $(M)^+$: 414.31; Found: 414.45.

2,2'-(5,5-Diphenylpentane-1,1-diyl)bis(4,4,5,5-tetramethyl-1,3,2-dioxaborolane) (2g):

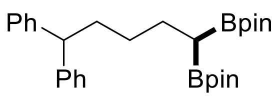

Colorless oil (85.7 mg, 60% yield); ^{1}H NMR (500 MHz, $CDCl_3$) δ: 7.26-7.21 (m, 8H), 7.15-7.11 (m, 2H), 3.87 (t, *J* = 8.0 Hz, 1H), 2.05-2.01 (m, 2H), 1.62-1.57 (m, 2H), 1.29-1.22 (m, 2H), 1.18 (s, 12H), 1.15 (s, 12H), 0.69 (t, *J* = 8.0 Hz, 1H); ^{13}C{^{1}H} NMR (126 MHz, $CDCl_3$) δ: 145.4, 128.2, 127.9, 125.8, 82.9, 51.2, 35.7, 30.9, 25.8, 24.8, 24.4; The boron-bound carbon was not detected due to quadrupolar relaxation. HRMS *m/z* (ESI) calcd for $C_{29}H_{42}B_2NaO_4$ $(M+Na)^+$: 499.3161; Found: 499.3175.

2,2'-(2-Methyl-5-phenylpentane-1,1-diyl)bis(4,4,5,5-tetramethyl-1,3,2-dioxaborolane) (2h):

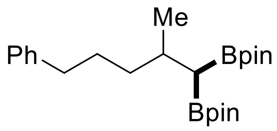

Colorless oil (73.3 mg, 59% yield); ^{1}H NMR (500 MHz, $CDCl_3$) δ: 7.26-7.23 (m, 2H), 7.17-7.13 (m, 3H), 2.57 (t, *J* = 8.0 Hz, 2H), 1.97-1.88 (m, 1H), 1.72-1.63 (m, 2H), 1.59-1.50 (m, 1H), 1.47-1.40 (m, 1H), 1.22 (s, 6H), 1.21 (s, 6H), 1.19 (s, 6H), 1.17 (s, 6H), 0.95 (d, *J* = 7.0 Hz, 3H), 0.70 (d, *J* = 10.0 Hz, 1H); ^{13}C{^{1}H} NMR (126 MHz, $CDCl_3$) δ: 143.0, 128.4, 128.1, 125.4, 82.8, 39.2, 36.2, 31.0, 28.9, 24.9, 24.8, 24.5, 21.7; The boron-bound carbon was not detected due to quadrupolar relaxation. HRMS *m/z* (ESI) calcd for $C_{24}H_{40}B_2NaO_4$ $(M+Na)^+$: 437.3004; Found: 437.3019.

2,2'-(4,4-Diphenylbutane-1,1-diyl)bis(4,4,5,5-tetramethyl-1,3,2-dioxaborolane) (2i):

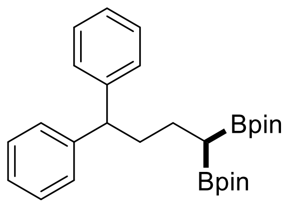

Colorless oil (101 mg, 73% yield); ^{1}H NMR (500 MHz, $CDCl_3$) δ: 7.28-7.22 (m, 9H), 7.15-7.12 (m, 1H), 3.91 (t, *J* = 7.5 Hz, 1H), 2.07-2.02 (m, 2H), 1.58-1.53 (m, 2H), 1.22 (s, 12H), 1.21 (s, 12H), 0.76 (t, *J* = 7.5 Hz, 1H); ^{13}C{^{1}H} NMR (126 MHz, $CDCl_3$) δ: 145.4, 128.2, 127.9, 125.8, 82.9, 51.4, 38.6, 24.9, 24.5, 24.1; The boron-bound carbon was not detected due to quadrupolar relaxation. HRMS *m/z* (ESI) calcd for $C_{28}H_{40}B_2NaO_4$ $(M+Na)^+$: 485.3005; Found: 485.3019.

2,2'-(3-Benzyloctane-1,1-diyl)bis(4,4,5,5-tetramethyl-1,3,2-dioxaborolane) (2j):

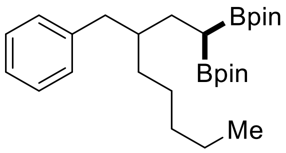

Colorless oil (94.4 mg, 69% yield); ^{1}H NMR (500 MHz, $CDCl_3$) δ: 7.24 (t, *J* = 7.5 Hz, 2H), 7.15-7.13 (m, 3H), 2.68-2.64 (m, 1H), 2.37-2.33 (m, 1H), 1.67-1.62 (m, 1H), 1.56-1.46 (m, 2H), 1.26-1.09 (m, 33H), 0.83 (t, *J* = 7.0 Hz, 1H); ^{13}C{^{1}H} NMR (126 MHz, $CDCl_3$) δ: 142.0, 129.3, 127.9, 125.3, 82.9, 82.9, 41.9, 40.2, 32.7, 32.2, 29.5, 26.1, 24.9, 24.8, 24.6, 24.5, 22.6, 14.1; The boron-bound carbon was not detected due to quadrupolar relaxation. HRMS *m/z* (ESI) calcd for $C_{27}H_{46}B_2NaO_4$ $(M+Na)^+$: 479.3474; Found: 479.3488.

2,2'-(4,8-Dimethylnon-7-ene-1,1-diyl)bis(4,4,5,5-tetramethyl-1,3,2-dioxaborolane) (2k):

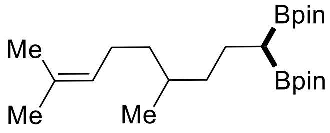

Colorless oil (58.5 mg, 48% yield); ^{1}H NMR (500 MHz, $CDCl_3$) δ: 5.10-5.08 (m, 1H), 1.95-1.91 (m, 2H), 1.67 (s,

3H), 1.59 (s, 3H), 1.54-1.50 (m, 1H), 1.40-1.27 (m, 4H), 1.23 (s, 12H), 1.22 (s, 12H), 1.14-1.07 (m, 2H), 0.85 (d, *J* = 6.5 Hz, 3H), 0.67 (d, *J* = 7.5 Hz, 1H); $^{13}C\{1H\}$ NMR (126 MHz, $CDCl_3$) δ: 130.8, 125.2, 82.9, 40.0, 37.2, 32.6, 25.7, 25.6, 24.9, 24.5, 23.1, 19.7, 17.6; The boron-bound carbon was not detected due to quadrupolar relaxation. HRMS *m/z* (ESI) calcd for $C_{23}H_{44}B_2NaO_4$ $(M+Na)^+$: 429.3318; Found: 429.3327.

2,2'-(2-Methyl-4-phenylbutane-1,1-diyl)bis(4,4,5,5-tetramethyl-1,3,2-dioxaborolane) (2l):

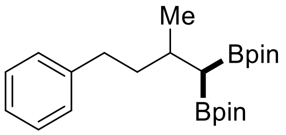

Colorless oil (84.0 mg, 70% yield); ^1H NMR (500 MHz, $CDCl_3$) δ: 8.35 (s, 1H), 7.33 (s, 5H), 7.16 (t, *J* = 7.6 Hz, 1H), 6.86-6.83 (m, 2H), 6.73 (t, *J* = 7.6 Hz, 1H), 4.12-4.05 (m, 1H), 3.80-3.71 (m, 2H), 3.58-3.52 (m, 1H), 3.50-3.42 (m, 2H), 2.73 (d, *J* = 14.4 Hz, 1H), 2.52 (d, *J* = 14.8 Hz, 1H), 1.49 (s, 3H); $^{13}C\{^1H\}$ NMR (126 MHz, $CDCl_3$) δ: 143.4, 128.3, 128.1, 125.3, 82.9, 41.5, 33.6, 31.1, 25.0, 24.9, 24.5 (2C), 21.6; The boron-bound carbon was not detected due to quadrupolar relaxation. HRMS *m/z* (ESI) calcd for $C_{23}H_{38}B_2NaO_4$ $(M+Na)^+$: 423.2848; Found: 423.2854.

2,2'-(2-Ethyl-4-phenylbutane-1,1-diyl)bis(4,4,5,5-tetramethyl-1,3,2-dioxaborolane) (2m):

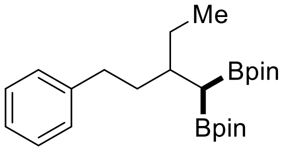

Colorless oil (78.2 mg, 63% yield); ^1H NMR (500 MHz, $CDCl_3$) δ: 7.26-7.23 (m, 2H), 7.18-7.13 (m, 3H), 2.61-2.49 (m, 2H), 1.98-1.91 (m, 1H), 1.71-1.60 (m, 3H), 1.54-1.40 (m, 2H), 1.23-1.21 (m, 24H), 0.94 (d, *J* = 10.0 Hz, 1H), 0.86 (t, *J* = 7.5 Hz, 0.7H); $^{13}C\{^1H\}$ NMR (126 MHz, $CDCl_3$) δ: 143.6, 128.3, 128.1, 125.3, 82.9, 36.7, 36.6, 32.8, 27.2,

24.9, 24.8, 24.6 (Two signals overlap), 10.3; The boron-bound carbon was not detected due to quadrupolar relaxation. HRMS *m/z* (ESI) calcd for $C_{24}H_{40}B_2NaO_4$ $(M+Na)^+$: 437.3005; Found: 437.3015.

2,2'-(2-Phenethyloctane-1,1-diyl)bis(4,4,5,5-tetramethyl-1,3,2-dioxaborolane) (2n):

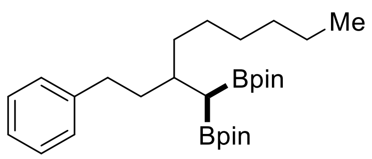

Colorless oil (100.1 mg, 71% yield); ^{1}H NMR (500 MHz, $CDCl_3$) δ: 7.44 (t, *J* = 7.6 Hz, 1.2H), 7.36-7.23 (m, 6.4H), 7.08-7.01 (m, 1.4H), 4.75 (s, 0.7H), 4.71 (s, 0.3H), 3.96-3.83 (m, 1.7H), 3.77-3.66 (m, 5.8H), 3.45-3.20 (m, 3.0H), 3.04 (d, *J* = 17.6 Hz, 0.7H); ^{13}C{^{1}H} NMR (126 MHz, $CDCl_3$) δ: 143.6, 128.3, 128.1, 125.3, 82.9, 37.2, 35.4, 35.1, 32.5, 31.9, 29.7, 26.1, 24.9 (two signals overlap), 24.6, 22.7, 14.1; The boron-bound carbon was not detected due to quadrupolar relaxation. GCMS *m/z* (EI) calcd for $C_{28}H_{48}B_2O_4$ $(M)^+$: 470.37; Found: 470.45.

2,2'-(2,4-Diphenylbutane-1,1-diyl)bis(4,4,5,5-tetramethyl-1,3,2-dioxaborolane) (2o):

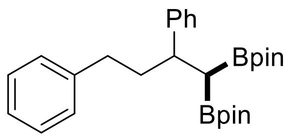

White solid (80.4 mg, 58% yield); ^{1}H NMR (500 MHz, $CDCl_3$) δ: 7.28-7.22 (m, 6H), 7.17-7.13 (m, 2H), 7.10-7.08 (m, 2H), 3.10-3.05 (m, 1H), 2.40-2.36 (m, 2H), 2.12-2.06 (m, 1H), 1.87-1.79 (m, 1H), 1.34 (d, *J* = 12.0 Hz, 1H), 1.28 (s, 6H), 1.27 (s, 6H), 0.90 (s, 6H); ^{13}C{^{1}H} NMR (126 MHz, $CDCl_3$) δ: 146.9, 142.7, 128.3, 128.1, 128.1, 127.9, 125.7, 125.4, 83.1, 82.8, 42.9, 41.6, 33.9, 25.0, 24.4, 24.3, 24.2; The boron-bound

carbon was not detected due to quadrupolar relaxation. HRMS *m/z* (ESI) calcd for $C_{28}H_{40}B_2NaO_4$ (M+Na)$^+$: 485.3005; Found: 485.3020.

2,2'-(4-Cyclohexyl-2-methylbutane-1,1-diyl)bis(4,4,5,5-tetramethyl-1,3,2-dioxaborolane) (2p):

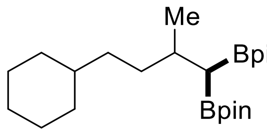

Colorless oil (96.2 mg, 79% yield); ^{1}H NMR (500 MHz, $CDCl_3$) δ: 1.87-1.82 (m, 1H), 1.71-1.59 (m, 5H), 1.41-1.36 (m, 1H), 1.22 (s, 12H), 1.21 (s, 12H), 1.19-1.07 (m, 7H), 0.91 (d, *J* = 6.5 Hz, 3H), 0.87-0.77 (m, 2H), 0.68 (d, *J* = 10.0 Hz, 1H); $^{13}C\{^1H\}$ NMR (126 MHz, $CDCl_3$) δ: 82.8, 37.8, 36.8, 34.8, 33.6, 33.2, 31.2, 26.8, 26.4 (two signals overlap), 24.9 (two signals overlap), 24.5 (two signals overlap), 21.6; The boron-bound carbon was not detected due to quadrupolar relaxation. HRMS *m/z* (ESI) calcd for $C_{23}H_{44}B_2NaO_4$ (M+Na)$^+$: 429.3318; Found: 429.3330.

2,2'-(2-Methyl-4,4-diphenylbutane-1,1-diyl)bis(4,4,5,5-tetramethyl-1,3,2-dioxaborolane) (2q):

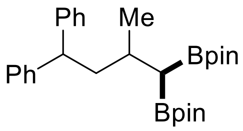

Colorless oil (87.1 mg, 61% yield); ^{1}H NMR (500 MHz, $CDCl_3$) δ: 7.35 (d, *J* = 7.5 Hz, 2H), 7.29-7.21 (m, 6H), 7.18-7.11 (m, 2H), 4.07-4.04 (m, 1H), 2.42-2.36 (m, 1H), 1.92-1.86 (m, 1H), 1.77-1.45 (m, 2H), 1.26 (s, 12H), 1.22 (d, *J* = 6.0 Hz, 12H), 1.00 (d, *J* = 6.5 Hz, 3H), 0.78 (d, *J* = 9.0 Hz, 1H); $^{13}C\{^1H\}$ NMR (126 MHz, $CDCl_3$) δ:146.7, 144.1, 128.4, 128.2 (two signals overlap), 127.6, 125.8, 125.6, 82.8 (two signals overlap), 49.0, 45.5, 28.5, 25.0, 24.8, 24.5, 24.4, 14.1; The boron-bound carbon was not detected due to quadrupolar relaxation. HRMS *m/z* (ESI) calcd for $C_{29}H_{42}B_2NaO_4$ (M+Na)$^+$: 499.3161; Found: 499.3164.

2,2'-(8-(Benzyloxy)-2-methyloctane-1,1-diyl)bis(4,4,5,5-tetramethyl-1,3,2-dioxaborolane) (2r):

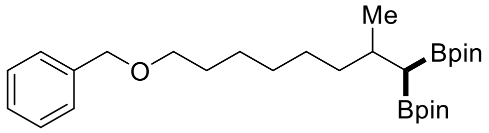

Colorless oil (112.3mg, 77% yield); ^{1}H NMR (500 MHz, $CDCl_3$) δ: 7.33-7.28 (m, 4H), 7.26-7.22 (m, 1H), 4.46 (s, 2H), 3.42 (t, *J* = 7.0 Hz, 2H), 1.89-1.82 (m, 2H), 1.60-1.54 (m, 2H), 1.36-1.18 (m, 30H), 1.12-1.04 (m, 1H), 0.90 (d, *J* = 6.5 Hz, 3H), 0.67 (d, *J* = 6.5 Hz, 1H); ^{13}C{1H} NMR (126 MHz, $CDCl_3$) δ: 138.7, 128.3, 127.6, 127.4, 82.8, 72.8, 70.5, 39.5, 30.9, 29.7, 29.6, 26.9, 26.1, 24.9, 24.8, 24.5, 24.5, 21.6; The boron-bound carbon was not detected due to quadrupolar relaxation. HRMS *m/z* (ESI) calcd for $C_{28}H_{48}B_2NaO_5$ $(M+Na)^+$: 509.3580; Found: 509.3596.

2,2'-(4-Phenylbutane-1,1-diyl)bis(4,4,5,5-tetramethyl-1,3,2-dioxaborolane) (2s):

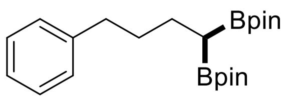

Colorless oil (101.9 mg, 88% yield); ^{1}H NMR (500 MHz, $CDCl_3$) δ: 7.25 (t, *J* = 7.5 Hz, 2H), 7.17-7.13 (m, 3H), 2.26 (t, *J* = 7.0 Hz, 2H), 1.64-1.60 (m, 4H), 1.23 (s, 12H), 1.22 (s, 12H), 0.78 (t, *J* = 6.0 Hz, 1H); ^{13}C{^{1}H} NMR (126 MHz, $CDCl_3$) δ: 142.9, 128.3, 128.1, 125.3, 82.9, 36.0, 34.3, 25.5, 24.8, 24.4; The boron-bound carbon was not detected due to quadrupolar relaxation. HRMS *m/z* (ESI) calcd for $C_{22}H_{36}B_2NaO_4$ $(M+Na)^+$: 409.2691; Found: 409.2710.

2,2'-(4-(2-Methoxyphenyl)butane-1,1-diyl)bis(4,4,5,5-tetramethyl-1,3,2-dioxaborolane) (2t):

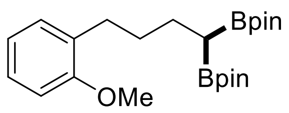

Colorless oil (98.6 mg, 79% yield); ^{1}H NMR (500 MHz, $CDCl_3$) δ: 7.15-7.12 (m, 2H), 6.86 (t, *J* = 7.5 Hz, 1H), 6.81 (d,

J = 8.0 Hz, 1H), 3.79 (s, 3H), 2.59 (t, J =7.5 Hz, 2H), 1.66-1.54 (m, 4H), 1.23 (s, 12H), 1.22 (s, 12H), 0.78 (t, J = 7.5 Hz, 1H); $^{13}C\{^1H\}$ NMR (126 MHz, $CDCl_3$) δ: 157.4, 131.4, 129.5, 126.5, 120.2, 110.0, 82.8, 55.1, 32.7, 30.2, 25.7, 24.8, 24.5; The boron-bound carbon was not detected due to quadrupolar relaxation. HRMS *m/z* (ESI) calcd for $C_{23}H_{38}B_2NaO_5$ $(M+Na)^+$: 439.2798; Found: 439.2802.

2,2'-(4-(2-(Trifluoromethyl)phenyl)butane-1,1-diyl)bis(4,4,5,5-tetramethyl-1,3,2-dioxaborolane) (2u):

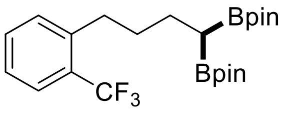

Colorless oil (103.5 mg, 76% yield); ^1H NMR (500 MHz, $CDCl_3$) δ: 7.57 (d, J = 8.0 Hz, 1H), 7.42 (t, J = 7.5 Hz, 1H), 7.30 (d, J = 7.5 Hz, 1H), 7.23 (t, J = 8.0 Hz, 1H), 2.75 (t, J = 6.5 Hz, 1H), 1.70-1.66 (m, 2H), 1.64-1.58 (m, 1H), 1.23 (s, 12H), 1.22 (s, 12H), 0.79 (t, J = 7.5 Hz, 1H); $^{13}C\{^1H\}$ NMR (126 MHz, $CDCl_3$) δ: 141.9, 131.5, 130.9, 128.3 (q, J = 29.6 Hz), 125.7 (q, J = 5.7 Hz), 125.5, 123.6, 82.9, 34.7, 32.8, 26.0, 24.8, 24.5; The boron-bound carbon was not detected due to quadrupolar relaxation. ^{19}F NMR (377 MHz, $CDCl_3$) δ: -59.7; HRMS *m/z* (ESI) calcd for $C_{23}H_{35}B_2{}^{19}F_3NaO_4$ $(M+Na)^+$: 477.2566; Found: 477.2585.

2,2'-(4-(3-Methoxyphenyl)butane-1,1-diyl)bis(4,4,5,5-tetramethyl-1,3,2-dioxabor olane) (2v):

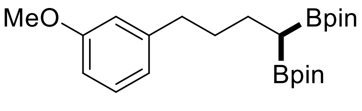

Colorless oil (99.8 mg, 80% yield); ^1H NMR (500 MHz, $CDCl_3$) δ: 7.16 (t, J = 8.0 Hz, 1H), 6.76 (d, J = 7.5 Hz, 1H), 6.72-6.69 (m, 2H), 3.78 (s, 3H), 2.58-2.56 (m, 2H), 1.63-1.61 (m, 4H), 1.23 (s, 12H), 1.22 (s, 12H), 0.76 (t, J = 6.5 Hz, 1H); $^{13}C\{^1H\}$ NMR (126 MHz, $CDCl_3$)

δ: 159.5, 144.7, 129.1, 120.8, 114.0, 110.9, 82.9, 55.1, 36.1, 34.2, 25.5, 24.9, 24.5; The boron-bound carbon was not detected due to quadrupolar relaxation. HRMS *m/z* (ESI) calcd for $C_{23}H_{38}B_2NaO_5$ $(M+Na)^+$: 439.2798; Found: 439.2799.

2,2'-(4-(*p*-Tolyl)butane-1,1-diyl)bis(4,4,5,5-tetramethyl-1,3,2-dioxaborolane) (2w):

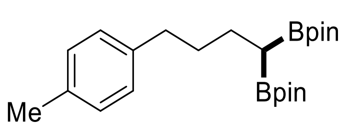

Colorless oil (99.6 mg, 83% yield); ^{1}H NMR (500 MHz, $CDCl_3$) δ: 7.06 (s, 4H), 2.55 (t, *J* = 7.5 Hz, 2H), 2.30 (s, 3H), 1.65-1.58 (m, 4H), 1.23 (s, 12H), 1.22 (s, 12H), 0.77 (t, *J* = 7.5 Hz, 1H); $^{13}C\{^1H\}$ NMR (126 MHz, $CDCl_3$) δ: 139.9, 134.7, 128.8, 128.1, 82.9, 35.5, 34.4, 25.5, 24.8, 24.5, 20.9; The boron-bound carbon was not detected due to quadrupolar relaxation. HRMS *m/z* (ESI) calcd for $C_{23}H_{38}B_2NaO_4$ $(M+Na)^+$: 423.2848; Found: 423.2860.

2,2'-(4-(4-Methoxyphenyl)butane-1,1-diyl)bis(4,4,5,5-tetramethyl-1,3,2-dioxaborolane) (2x):

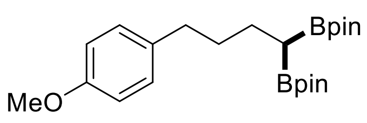

Colorless oil (106.1 mg, 85% yield); ^{1}H NMR (500 MHz, $CDCl_3$) δ: 7.07 (d, *J* = 8.5 Hz, 2H), 6.79 (d, *J* = 8.5 Hz, 2H), 3.77 (s, 3H), 2.53 (t, *J* = 7.5 Hz, 2H), 1.63-1.54 (m, 4H), 1.22 (s, 12H), 1.21 (s, 12H), 0.76 (t, *J* = 7.0 Hz, 1H); $^{13}C\{^1H\}$ NMR (126 MHz, $CDCl_3$) δ: 157.4, 135.1, 129.1, 113.5, 82.9, 55.2, 35.0, 34.5, 25.4, 24.8, 24.5; The boron-bound carbon was not detected due to quadrupolar relaxation. HRMS *m/z* (ESI) calcd for $C_{23}H_{38}B_2NaO_5$ $(M+Na)^+$: 439.2798; Found: 439.2803.

4-(4,4-Bis(4,4,5,5-tetramethyl-1,3,2-dioxaborolan-2-yl)butyl)-N,N-dimethylanilin e (2y):

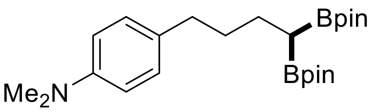

White solid (106.8 mg, 83% yield); ^{1}H NMR (500 MHz, $CDCl_3$) δ: 7.07-7.05 (m, 2H), 6.71-6.68 (m, 2H), 2.90 (s, 6H), 2.52 (t, *J* = 7.5 Hz, 2H), 1.66-1.77 (m, 4H), 1.24 (s, 12H), 1.23 (s, 12H), 0.78 (t, *J* = 7.5 Hz, 1H); ^{13}C{^{1}H} NMR (126 MHz, $CDCl_3$) δ: 148.8, 131.4, 128.8, 113.0, 82.8, 40.9, 34.9, 34.6, 25.5, 24.8, 24.5; The boron-bound carbon was not detected due to quadrupolar relaxation. HRMS *m/z* (ESI) calcd for $C_{24}H_{42}B_2NO_4$ $(M+H)^+$: 430.3295; Found: 430.3317.

2,2'-(4-(4-(Methylthio)phenyl)butane-1,1-diyl)bis(4,4,5,5-tetramethyl-1,3,2-dioxa borolane) (2z):

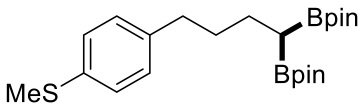

Colorless oil (86.8 mg, 67% yield); ^{1}H NMR (500 MHz, $CDCl_3$) δ: 7.19-7.17 (m, 2H), 7.10-7.08 (m, 2H), 2.55 (t, *J* = 7.0 Hz, 2H), 2.46 (s, 3H), 1.61-1.58 (m, 4H), 1.22 (s, 12H), 1.21 (s, 12H), 0.75 (t, *J* = 7.0 Hz, 1H); ^{13}C{^{1}H} NMR (126 MHz, $CDCl_3$) δ: 140.3, 134.7, 128.9, 127.3, 82.9, 35.4, 34.2, 25.4, 24.9, 24.5, 16.5; The boron-bound carbon was not detected due to quadrupolar relaxation. HRMS *m/z* (ESI) calcd for $C_{23}H_{38}B_2NaO_4S$ $(M+Na)^+$: 455.2569; Found: 455.2578.

2,2'-(4-(4-(*tert*-Butyl)phenyl)butane-1,1-diyl)bis(4,4,5,5-tetramethyl-1,3,2-dioxab orolane) (2aa):

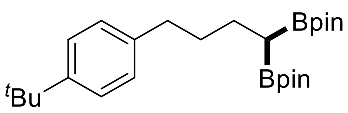

Colorless oil (108.7 mg, 82% yield); ^{1}H NMR (500 MHz, $CDCl_3$) δ: 7.34-7.21 (m, 6H), 7.03 (d, *J* = 8.4 Hz,

1H), 6.99-6.97 (m, 1H), 6.79 (t, J = 7.6 Hz, 1H), 3.60-3.52 (m, 2H), 3.42 (s, 3H), 2.46-2.36 (m, 2H), 1.36 (s, 3H), 1.31 (t, J = 7.2 Hz, 3H), 1.23 (s, 3H); $^{13}C\{^1H\}$ NMR (100 MHz, $CDCl_3$) δ: 148.04, 139.9, 127.9, 125.0, 82.9, 35.4, 34.2 (2C), 31.4, 25.6, 24.8, 24.5; The boron-bound carbon was not detected due to quadrupolar relaxation. HRMS m/z (ESI) calcd for $C_{26}H_{44}B_2NaO_4$ $(M+Na)^+$: 465.3318; Found: 465.3326.

2,2'-(4-(4-Fluorophenyl)butane-1,1-diyl)bis(4,4,5,5-tetramethyl-1,3,2-dioxaborolane) (2ab):

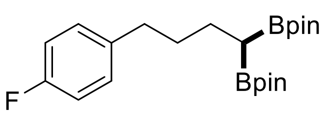

Colorless oil (86.8 mg, 79% yield); ^1H NMR (500 MHz, $CDCl_3$) δ: 7.14-7.11 (m, 2H), 6.97-693 (m, 2H), 2.58 (t, J = 7.0 Hz, 2H), 1.63-1.61 (m, 4H), 1.25 (s, 12H), 1.24 (s, 12H), 0.78 (t, J = 7.0 Hz, 1H); $^{13}C\{^1H\}$ NMR (126 MHz, $CDCl_3$) δ: 161.1 (q, J = 242.5 Hz), 138.5 (q, J = 3.1 Hz), 129.6 (q, J = 7.7 Hz), 114.8 (q, J = 21.0 Hz), 82.9, 35.1, 34.3, 25.3, 24.8, 24.5; The boron-bound carbon was not detected due to quadrupolar relaxation. ^{19}F NMR (377 MHz, $CDCl_3$) δ: -118.5; HRMS m/z (ESI) calcd for $C_{22}H_{35}B2^{19}FNaO_4$ $(M+Na)^+$: 427.2598; Found: 427.2606.

4-(4,4-Bis(4,4,5,5-tetramethyl-1,3,2-dioxaborolan-2-yl)butyl)phenyl acetate (2ac):

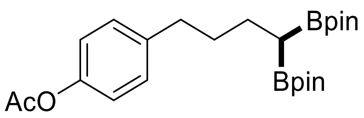

White solid (51.9 mg, 39% yield); ^1H NMR (500 MHz, $CDCl_3$) δ: 7.18 (d, J = 8.5 Hz, 2H), 6.98 (d, J = 8.5 Hz, 2H), 2.60 (t, J = 7.0 Hz, 2H), 2.30 (s, 3H), 1.64-1.62 (m, 4H), 1.25 (s, 12H), 1.24 (s, 12H), 0.79 (t, J = 7.0 Hz, 1H); $^{13}C\{^1H\}$ NMR (126 MHz, $CDCl_3$) δ: 169.7, 148.5, 140.5, 129.2, 121.1, 83.0, 35.3, 34.1, 25.5, 24.8, 24.5, 21.1; The boron-bound carbon was not

detected due to quadrupolar relaxation. HRMS *m/z* (ESI) calcd for $C_{24}H_{38}B_2NaO_6$ $(M+Na)^+$: 467.2747; Found: 467.2743.

2,2'-(4-(4-(1,3-Dioxolan-2-yl)phenyl)butane-1,1-diyl)bis(4,4,5,5-tetramethyl-1,3,2-dioxaborolane) (2ad):

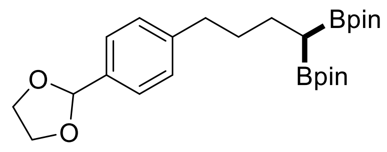

Colorless oil (108.5 mg, 79% yield); ^1H NMR (500 MHz, $CDCl_3$) δ: 7.35 (d, J = 8.0 Hz, 2H), 7.17 (d, J = 8.0 Hz, 2H), 5.77 (s, 1H), 4.12-4.10 (m, 2H), 4.02-3.99 (m, 2H), 2.59 (t, J = 7.0 Hz, 2H), 1.61-1.59 (m, 4H), 1.22 (s, 12H), 1.21 (s, 12H), 0.75 (t, J = 6.5 Hz, 1H); $^{13}C\{1H\}$ NMR (126 MHz, $CDCl_3$) δ: 144.1, 134.9, 128.3, 126.2, 103.8, 82.9, 65.2, 35.7, 34.1, 25.4, 24.8, 24.5; The boron-bound carbon was not detected due to quadrupolar relaxation. HRMS *m/z* (ESI) calcd for $C_{25}H_{41}B_2O_6$ $(M+H)^+$: 459.3083; Found: 459.3098.

2,2'-(Nonane-1,1-diyl)bis(4,4,5,5-tetramethyl-1,3,2-dioxaborolane) (2ae):

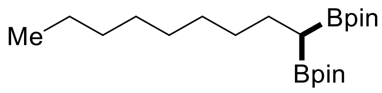

Colorless oil (85.5 mg, 75% yield); ^1H NMR (500 MHz, $CDCl_3$) δ: 1.52-1.49 (m, 2H), 1.26-1.23 (m, 12H), 1.21 (m, 12H), 1.20 (m, 12H), 0.85 (t, J = 7.0 Hz, 3H), 0.69 (t, J = 8.0 Hz, 1H); $^{13}C\{^1H\}$ NMR (126 MHz, $CDCl_3$) δ: 82.8, 32.5, 31.9, 29.6, 29.5, 29.2, 25.6, 24.8 (two signals overlap), 24.5, 22.7, 14.1; The boron-bound carbon was not detected due to quadrupolar relaxation. HRMS *m/z* (ESI) calcd for $C_{21}H_{42}B_2NaO_4$ $(M+Na)^+$: 403.3161; Found: 403.3166.

2,2'-(Decane-1,1-diyl)bis(4,4,5,5-tetramethyl-1,3,2-dioxaborolane) (2af):

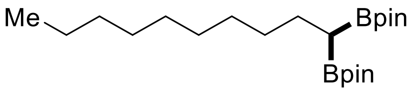

Colorless oil (92.2 mg, 78% yield); ^{1}H NMR (500 MHz, $CDCl_3$) δ: 1.54-1.50 (m, 2H), 1.29-1.23 (m, 14H), 1.22 (s, 12H), 1.21 (s, 12H), 0.86 (t, *J* =7.0 Hz, 3H), 0.70 (t, *J* = 8.0 Hz, 1H); ^{13}C{^{1}H} NMR (126 MHz, $CDCl_3$) δ: 82.8, 32.6, 31.9, 29.6, 29.5 (two signals overlap), 29.3, 25.7, 24.8, 24.5, 22.7, 14.1; The boron-bound carbon was not detected due to quadrupolar relaxation. HRMS *m/z* (ESI) calcd for $C_{22}H_{44}B_2NaO_4$ $(M+Na)^+$: 417.3318; Found: 417.3324.

2,2'-(Dodecane-1,1-diyl)bis(4,4,5,5-tetramethyl-1,3,2-dioxaborolane) (2ag):

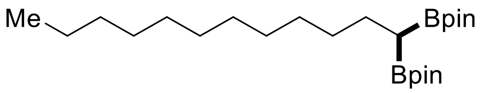

Colorless oil (96.2 mg, 76% yield); ^{1}H NMR (500 MHz, $CDCl_3$) δ: 1.53-1.50 (m, 2H), 1.29-1.23 (m, 18H), 1.21 (s, 12H), 1.20 (s, 12H), 0.86 (t, *J* = 7.0 Hz, 3H), 0.70 (t, *J* = 8.0 Hz, 1H); ^{13}C{^{1}H} NMR (126 MHz, $CDCl_3$) δ: 82.8, 32.5, 31.9, 29.6 (two signals overlap), 29.5, 29.3, 25.7, 24.8 (two signals overlap), 24.5, 22.7, 14.1; The boron-bound carbon was not detected due to quadrupolar relaxation. HRMS *m/z* (ESI) calcd for $C_{24}H_{48}B_2NaO_4$ $(M+Na)^+$: 445.3631; Found: 445.3645.

2,2'-(4-Cyclohexylbutane-1,1-diyl)bis(4,4,5,5-tetramethyl-1,3,2-dioxaborolane) (2ah):

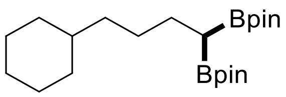

Colorless oil (83.5 mg, 71% yield); ^{1}H NMR (500 MHz, $CDCl_3$) δ: 1.66-1.62 (m, 5H), 1.52-1.47 (m, 2H), 1.29-1.24 (m, 3H), 1.22 (s, 12H), 1.21 (s, 12H), 1.17-1.11 (m, 5H), 0.85-0.77 (m, 2H), 0.70 (t, *J* = 8.0 Hz, 1H); ^{13}C{^{1}H} NMR (126 MHz, $CDCl_3$) δ: 82.8, 37.4 (two signals overlap),

33.4 (two signals overlap), 29.7, 26.8, 26.4, 25.9, 24.8, 24.5; The boron-bound carbon was not detected due to quadrupolar relaxation. HRMS *m/z* (ESI) calcd for $C_{22}H_{42}B_2NaO_4$ (M+Na)$^+$: 415.3161; Found: 415.3164.

2,2'-(4-(Furan-2-yl)butane-1,1-diyl)bis(4,4,5,5-tetramethyl-1,3,2-dioxaborolane) (2aj):

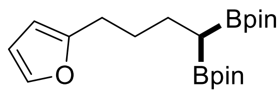

Colorless oil (81.2 mg, 72% yield); ^{1}H NMR (500 MHz, $CDCl_3$) δ: 7.26 (s, 1H), 6.25-6.24 (m, 1H), 5.96-5.95 (m, 1H), 2.61-2.58 (m, 2H), 1.64-1.60 (m, 4H), 1.23 (s, 12H), 1.22 (s, 12H), 0.75 (d, *J* = 6.5 Hz, 1H);^{13}C{^{1}H} NMR (126 MHz, $CDCl_3$) δ: 156.7, 140.4, 110.0, 104.3, 82.9, 30.6, 28.1, 25.4, 24.8, 24.5; The boron-bound carbon was not detected due to quadrupolar relaxation. HRMS *m/z* (ESI) calcd for $C_{20}H_{34}B_2NaO_5$ (M+Na)$^+$: 399.2485; Found: 399.2494.

2,2'-(4-(Benzofuran-2-yl)butane-1,1-diyl)bis(4,4,5,5-tetramethyl-1,3,2-dioxaborolane) (2ak):

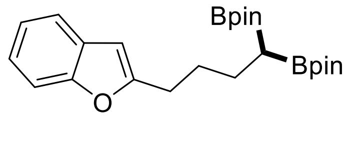

Colorless oil (69.0 mg, 54% yield); ^{1}H NMR (500 MHz, $CDCl_3$) δ: 7.47-7.45 (m, 1H), 7.38 (d, *J* = 7.5 Hz, 1H), 7.20-7.14 (m, 2H), 6.38 (s, 1H), 2.75 (t, *J* = 7.5 Hz, 2H), 1.78-1.72 (m, 2H), 1.70-1.65 (m, 2H), 1.23 (s, 12H), 1.22 (s, 12H), 0.79 (t, *J* = 8.0 Hz, 1H);^{13}C{^{1}H} NMR (126 MHz, $CDCl_3$) δ:159.9, 154.6, 129.1, 122.8, 122.2, 120.1, 110.6, 101.5, 83.0, 30.2, 28.5, 25.3, 24.8, 24.5; The boron-bound carbon was not detected due to quadrupolar relaxation. GCMS *m/z* (EI) calcd for $C_{24}H_{36}B_2O_5$ (M)$^+$: 426.27; Found 426.25.

3-(4,4-Bis(4,4,5,5-tetramethyl-1,3,2-dioxaborolan-2-yl)butyl)-1-methyl-1*H*-indole (2al):

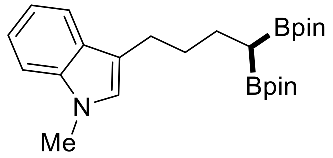

White solid (104.0 mg, 79% yield); ^{1}H NMR (500 MHz, $CDCl_3$) δ: 7.59 (d, *J* = 8.0 Hz, 1H), 7.27 (t, *J* = 8.0 Hz, 1H), 7.21-7.18 (m, 1H), 7.09-7.06 (m, 1H), 6.84 (s, 1H), 3.73 (s, 3H), 2.74 (t, *J* = 7.0 Hz, 2H), 1.76-1.69 (m, 4H), 1.25 (s, 12H), 1.24 (s, 12H), 0.82 (t, *J* = 7.0 Hz, 1H); ^{13}C{^{1}H} NMR (126 MHz, $CDCl_3$) δ:137.0, 128.0, 125.8, 121.2, 119.1, 118.2, 115.6, 108.9, 82.9, 33.2, 32.4, 25.8, 25.1, 24.8, 24.5; The boron-bound carbon was not detected due to quadrupolar relaxation. HRMS *m/z* (ESI) calcd for $C_{25}H_{40}B_2NO_4$ $(M+H)^+$: 440.3138; Found: 440.3155.

4,4,5,5-Tetramethyl-2-(1-phenylpentyl)-1,3,2-dioxaborolane (3):

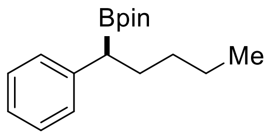

Colorless oil (59.2 mg, 72% yield); ^{1}H NMR (500 MHz, $CDCl_3$) δ: 7.26-7.23 (m, 2H), 7.21-7.20 (m, 2H), 7.14-7.11 (m, 1H), 2.86 (t, *J* = 8.0 Hz, 1H), 1.87-1.80 (m, 1H), 1.68-1.61 (m, 1H), 1.35-1.22 (m, 4H), 1.20 (s, 6H), 1.18 (s, 6H), 0.86 (t, *J* = 7.0 Hz, 1H); ^{13}C{^{1}H} NMR (126 MHz, $CDCl_3$) δ: 143.5, 128.3, 128.2, 125.0, 83.2, 32.3, 31.5, 24.6, 24.6, 22.7, 14.0; The boron-bound carbon was not detected due to quadrupolar relaxation. GCMS *m/z* (EI) calcd for $C_{17}H_{27}BO_2$ $(M)^+$: 274.21; Found: 274.20.

(*S*)-2-(1,4-Diphenylbutyl)-4,4,5,5-tetramethyl-1,3,2-dioxaborolane (4):

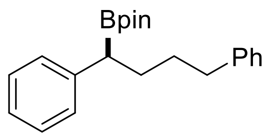

Colorless oil (79.6 mg, 79% yield); ^{1}H NMR (500 MHz, $CDCl_3$) δ: 7.27-7.23 (m, 2H), 7.17-7.11 (m, 3H), 2.56 (t, *J* = 8.0 Hz, 2H), 1.63-1.53 (m, 4H), 1.34-1.31 (m, 4H), 1.23 (s, 12H), 1.22 (s, 12H), 0.72

(t, J = 7.5 Hz, 1H); $^{13}C\{^{1}H\}$ NMR (126 MHz, $CDCl_3$) δ: 143.2, 142.7, 128.3 (2C), 128.2 (2C), 125.5, 125.1, 83.3, 35.9, 32.3, 31.0, 24.6, 24.6; The boron-bound carbon was not detected due to quadrupolar relaxation. GCMS *m/z* (EI) calcd for $C_{22}H_{29}BO_2$ $(M)^+$: 336.23; Found: 336.20.

2,2'-(Hexane-1,1-diyl)bis(4,4,5,5-tetramethyl-1,3,2-dioxaborolane) (2ap):

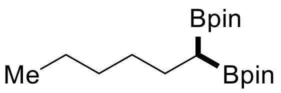

Colorless oil (63.9 mg, 63% yield); ^{1}H NMR (500 MHz, $CDCl_3$) δ: 1.54-1.49 (m, 2H), 1.33-1.20 (m, 30H), 0.84 (t, J = 7.0 Hz, 3H), 0.70 (t, J = 8.0 Hz, 1H); $^{13}C\{^{1}H\}$ NMR (126 MHz, $CDCl_3$) δ: 82.8, 32.2, 31.8, 25.6, 24.8, 24.5, 22.5, 14.0; The boron-bound carbon was not detected due to quadrupolar relaxation. GCMS *m/z* (EI) calcd for $C_{18}H_{36}B_2O_4$ $(M)^+$: 338.28; Found: 338.20.

1,6-Bis(4,4,5,5-tetramethyl-1,3,2-dioxaborolan-2-yl)hexane (2ap'):

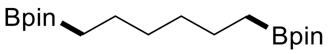

Colorless oil (82.1 mg, 81% yield); ^{1}H NMR (400 MHz, $CDCl_3$) δ: 1.41-1.35 (m, 4H), 1.29-1.25 (m, 4H), 1.23 (s, 24H), 0.75 (t, J = 7.6 Hz, 4H); $^{13}C\{^{1}H\}$ NMR (101 MHz, $CDCl_3$) δ: 82.8, 32.2, 24.8, 23.9; The boron-bound carbon was not detected due to quadrupolar relaxation. GCMS *m/z* (EI) calcd for $C_{18}H_{36}B_2O_4$ $(M)^+$: 338.28; Found: 338.20.

Procedures for Derivatization of 2s

Gram-Scale Synthesis of 2s:

A flame-dried 25-mL Schlenk tube was charged with $Co(acac)_2$ (10.3 mg, 0.5 mol %), dcpe (17.7 mg, 0.5 mol %), (*Z*/*E*)-1,3-diene **1s** (8 mmol), dry THF (10 mL) and a magnetic stirring bar. The reaction mixture was charged with pinacolborane (20 mmol, 2.5 equiv) and stirred at 100 °C for 3 h. Then the solvent was removed under reduced pressure and the residue was purified by silica gel flash column chromatography with a mixture of hexane and ethyl acetate (40:1) as eluent, yielding the titled compound **2s** (2.53 g, 82% yield) as a colorless oil.

1-(Furan-2-yl)-5-phenylpentan-2-one (5):[7]

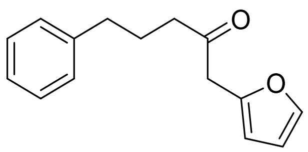

To a solution of **2s** (116 mg, 0.30 mmol) in THF (0.5 mL) was added a solution of LiTMP (1.0 equiv) in THF (0.4 mL) at 0 °C. The reaction mixture was then cooled to -78 °c and dropwise addition a solution of furan-2-carbaldehyde (1.05 equiv) in THF (0.5 mL). The reaction was maintained at 0 °C for 4 h, after which the solvent was removed and replaced with THF (2 mL). An aqueous solution of sodium perborate tetrahydrate (9.0 equiv, 2 mL) was added slowly at 0 °C. The reaction mixture was warm to room temperature and stirred for 2 h. The mixture was extracted with EtOAc (2 x 5 mL) and the combined extracts were washed with brine and dried with Na_2SO_4. The crude product was purified using silica gel flash column chromatography with 20:1 hexane/EtOAc to yield the title compound as a colorless oil (51.3 mg, 75% yield); ^{1}H NMR (500 MHz, $CDCl_3$) δ: 7.36 (s, 1H), 7.28 (t, *J* = 7.5 Hz, 2H), 7.19 (t, *J* = 7.5 Hz,

1H), 7.14 (d, J = 7.0 Hz, 2H), 6.35-6.34 (m, 1H), 6.18-6.17 (m, 1H), 3.68 (s, 2H), 2.60 (t, J = 7.5 Hz, 2H), 2.47 (d, J = 7.5 Hz, 2H), 1.93-1.87 (m, 2H); $^{13}C\{^1H\}$ NMR (126 MHz, $CDCl_3$) δ: 205.9, 148.2, 142.1, 141.5, 128.4, 128.3, 125.9, 110.6, 108.2, 42.5, 41.0, 34.9, 25.0; HRMS m/z (ESI) calcd for $C_{15}H_{16}NaO_2$ $(M+Na)^+$: 251.1042; Found: 251.1043.

2-(4-Phenylbutyl)quinoline (6):[8]

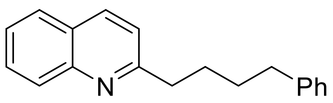

The compound was synthesized based on the known literature.[4] To a 4-mL screw-capped vial with a magnetic stirbar, quinoline N-oxide (0.15 mmol), NaOMe (3.0 equiv), 1,1-diborylalkane **2s** (2.0 equiv), and toluene (2.0 mL) were added at at 80 °C for 4 h. The crude mixture was purified by column chromatography on silica gel (n-hexanes:EtOAc, 10:1) to give compound **4** as a colorless oil (25.8 mg, 66% yield); ^1H NMR (500 MHz, $CDCl_3$) δ: 8.08-8.05 (m, 2H), 7.79-7.78 (m, 1H), 7.71-7.68 (m, 1H), 7.51-7.48 (m, 1H), 7.30-7.26 (m, 3H), 7.19-7.16 (m, 3H), 3.02 (t, J = 8.0 Hz, 2H), 2.69 (t, J = 8.0 Hz, 2H), 1.92-1.86 (m, 2H), 1.80-1.73 (m, 2H); $^{13}C\{^1H\}$ NMR (126 MHz, $CDCl_3$) δ: 162.7, 147.8, 142.4, 136.3, 129.4, 128.8, 128.4, 128.2, 127.5, 126.7, 125.7 (Two signals overlap), 121.3, 39.1, 35.8, 31.3, 29.6; HRMS m/z (ESI) calcd for $C_{19}H_{20}N$ $(M+H)^+$: 262.1590; Found: 262.1586.

2,2'-(5-Phenylpentane-2,2-diyl)bis(4,4,5,5-tetramethyl-1,3,2-dioxaborolane) (7):[9]

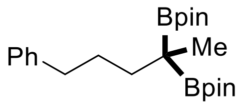

In an argon-filled dry box, a 4-mL screw-capped vial was charged with a solution of **2s** (115.8 mg, 0.3 mmol) in THF (0.5 mL). The vial was sealed with a cap containing a PTFE septum with silicone seal and

removed from the dry box. The reaction was cooled to 0 °C, and LiTMP (2.0 equiv) in THF (1 mL) was added. The mixture was allowed to stir at 0 °C for 20 minutes and was then transferred dropwise via syringe to a second flask containing a solution of iodomethane (1.2 equiv) in THF (0.5 mL) at 0 °C for 10 min. The mixture was allowed to react at room temperature for 4 h. The crude reaction mixture was purified on silica gel (hexanes: ethyl acetate = 40:1) to afford the desired product as a colorless oil (99.6 mg, 83% yield); ^{1}H NMR (500 MHz, $CDCl_3$) δ: 7.27-7.23 (m, 2H), 7.17-7.11 (m, 3H), 2.56 (t, J = 8.0 Hz, 2H), 1.63-1.53 (m, 4H), 1.34-1.31 (m, 4H), 1.23 (s, 12H), 1.22 (s, 12H), 0.72 (t, J = 7.5 Hz, 1H); ^{13}C{^{1}H} NMR (126 MHz, $CDCl_3$) δ: 143.2, 128.3, 128.1, 125.4, 82.9, 36.8, 33.9, 29.6, 24.7, 24.6, 15.9; The boron-bound carbon was not detected due to quadrupolar relaxation. GCMS m/z (EI) calcd for $C_{23}H_{38}B_2O_4$ $(M)^+$: 400.30; Found: 400.20.

2,2'-(1-Phenylnon-8-ene-4,4-diyl)bis(4,4,5,5-tetramethyl-1,3,2-dioxaborolane) (8):[9]

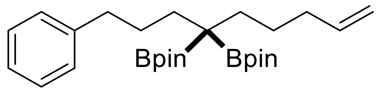

In an argon-filled dry box, a 4-mL screw-capped vial was charged with a solution of **2s** (115.8 mg, 0.3 mmol) in THF (0.5 mL). The vial was sealed with a cap containing a PTFE septum with silicone seal and removed from the dry box. The reaction was cooled to 0 °C, and NaHMDS (3.0 equiv) in THF (1 mL) was added. The mixture was allowed to stir at 0 °C for 20 minutes and was then transferred dropwise via syringe to a second flask containing a solution of 5-bromo-pent-1-ene (1.2 equiv) in THF (0.5 mL) at 0 °C for 10 min. The mixture was allowed to react at room temperature for 12 h. The crude reaction mixture was

purified on silica gel (hexanes: ethyl acetate = 40:1) to afford the desired product as a colorless oil (99.4 mg, 73% yield); ^{1}H NMR (500 MHz, $CDCl_3$) δ: 7.30-7.27 (m, 2H), 7.21-7.17 (m, 3H), 5.88-5.80 (m, 1H), 5.03-4.98 (m, 1H), 4.95-4.92 (m, 1H), 2.63 (d, *J* = 8.0 Hz, 2H), 2.06-2.02 (m, 2H), 1.75-1.70 (m, 2H), 1.66-1.63 (m, 2H), 1.60-1.54 (m, 2H), 1.33-1.28 (m, 2H), 1.25 (s, 24H); ^{13}C{^{1}H} NMR (126 MHz, $CDCl_3$) δ: 143.2, 139.4, 128.3, 128.1, 125.4, 113.8, 82.9, 36.8, 34.5, 29.2, 29.0, 28.6, 26.6, 24.7 (Two signals overlap); The boron-bound carbon was not detected due to quadrupolar relaxation. HRMS *m/z* (ESI) calcd for $C_{27}H_{44}B_2O_4$ $(M+H)^+$: 454.3426; Found: 454.3430.

(*R*)-4,4,5,5-Tetramethyl-2-(1-phenylnon-8-en-4-yl)-1,3,2-dioxaborolane (9):[9]

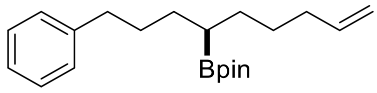

In an argon-filled dry box, a 4-mL screw-capped vial with magnetic stir bar was charged with **2s** (115.8 mg, 0.3 mmol), 5-bromo-pent-1-ene (1.2 equiv), THF (0.50 mL), and NaO^{t}Bu (0.9 mmol, 3.0 equiv). The vial was sealed with a polypropylene cap, removed from the dry box, and was then allowed to stir at room temperature for 12 h. The crude reaction mixture was purified on silica gel (hexanes: ethyl acetate = 100:1) to afford the desired product as a colorless oil (49.2 mg, 50% yield); ^{1}H NMR (500 MHz, $CDCl_3$) δ: 7.29-7.26 (m, 2H), 7.18-7.15 (m, 3H), 5.85-5.77 (m, 1H), 5.01-4.98 (m, 1H), 4.94-4.91 (m, 1H), 2.61 (t, *J* = 8.0 Hz, 2H), 2.06-2.02 (m, 2H), 1.66-1.60 (m, 2H), 1.53-1.35 (m, 6H), 1.24 (s, 12H), 1.05-1.00 (m, 1H); ^{13}C{^{1}H} NMR (126 MHz, $CDCl_3$) δ: 142.8, 139.1, 128.3, 128.1, 125.5, 114.1, 82.8, 36.2, 34.0, 31.1, 30.8, 28.5, 24.8 (Two signals overlap); The boron-bound carbon was not detected due to quadrupolar

relaxation. HRMS *m/z* (ESI) calcd for $C_{21}H_{33}BNaO_2$ (M+Na)$^+$: 351.2466; Found: 351.2464.

(*R*)-2-(1-(4-Methoxyphenyl)-4-phenylbutyl)-4,4,5,5-tetramethyl-1,3,2-dioxaborolane (10):[10]

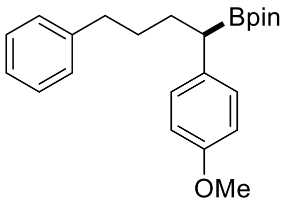

In an Ar-filled dry box, a 4-mL screw-capped vial was charged with Pd(P^{t}Bu$_3$)$_2$ (2.5 mg), **2s** (115.8 mg, 0.3 mmol), 4-iodoanisole (0.30 mmol), 1,4-dioxane (1.5 mL) and a magnetic stirring bar. The vial was sealed with a cap containing a PTFE septum with silicone seal and removed from the dry box. KOH (40% aq, 4.5 equiv) was added to the vial using a siringe and the mixure was then stirred at room temperature for 2 h. The reaction was extracted with EtOAc (3 x 10 mL) and the combined extracts were washed with brine and dried with Na_2SO_4. The crude product was purified using silica gel flash column chromatography with 25:1 hexane/EtOAc to attain the desired product as a yellow oil (88.9 mg, 81% yield); ^{1}H NMR (500 MHz, $CDCl_3$) δ: 7.31-7.28 (m, 2H), 7.21-7.16 (m, 5H), 6.85 (d, *J* = 9.0 Hz, 2H), 3.82 (s, 3H), 2.71-2.60 (m, 2H), 2.33 (d, *J* = 8.0 Hz, 2H), 1.96-1.89 (m, 1H), 1.77-1.69 (m, 1H), 1.68-1.62 (m, 2H), 1.26 (s, 6H), 1.24 (s, 6H); ^{13}C{^{1}H} NMR (126 MHz, $CDCl_3$) δ: 157.3, 142.7, 135.1, 129.2, 128.3, 128.1, 125.5, 113.7, 83.2, 55.1, 35.9, 32.5, 31.0, 24.6 (2C); HRMS *m/z* (ESI) calcd for $C_{23}H_{32}BO_3$ (M+H)$^+$: 367.2439; Found: 367.2437.

(*E*)-4,4,5,5-Tetramethyl-2-(4-phenylbut-3-en-1-yl)-1,3,2-dioxaborolane (11s):

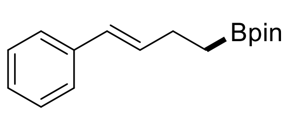

^{1}H NMR (500 MHz, $CDCl_3$) δ: 7.34-7.32 (m, 2H), 7.29-7.26 (m, 2H), 7.19-7.16 (m, 1H), 6.38 (d, *J* = 14.5 Hz, 1H),

6.31-6.25 (m, 1H), 2.36-2.31 (m, 2H), 1.24 (s, 12H), 0.98 (t, *J* = 7.5 Hz, 2H); $^{13}C\{^1H\}$ NMR (126 MHz, $CDCl_3$) δ: 138.0, 132.8, 128.8, 128.4, 126.7, 125.9, 83.1, 27.3, 24.8; The boron-bound carbon was not detected due to quadrupolar relaxation. GCMS *m/z* (EI) calcd for $C_{16}H_{23}BO_2$ $(M)^+$: 258.18; Found: 258.30.

4,4,5,5-Tetramethyl-2-(4-phenylbut-2-en-1-yl)-1,3,2-dioxaborolane and (*E*)-4,4,5,5-Tetramethyl-2-(4-phenylbut-3-en-1-yl)-1,3,2-dioxaborolane (11s′):[11]

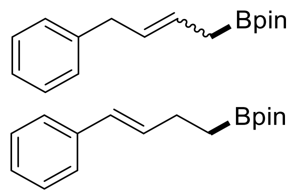

^1H NMR (500 MHz, $CDCl_3$) δ: 7.33 (d, *J* = 7.0 Hz, 2.2H), 7.30-7.26 (m, 4.1H), 7.20-7.16 (m, 3.8H), 6.38 (d, *J* = 16.0 Hz, 1.0H), 6.32-6.26 (m, 1.0H), 5.63-5.53 (m, 1.5H), 3.34 (d, *J* = 6.0 Hz, 1.7H), 2.37-2.33 (m, 2.0H), 1.71 (d, *J* = 6.0 Hz, 1.7H), 1.26 (s, 12.0H), 1.25 (s, 12.0H), 1.00 (d, *J* = 8.0 Hz, 2.1H); $^{13}C\{^1H\}$ NMR (126 MHz, $CDCl_3$) δ: 141.3, 138.0, 132.7, 129.2, 128.8, 128.4 (2C), 128.2, 126.7, 126.6, 125.9, 125.7, 83.2, 83.0, 39.1, 27.3, 24.8, 24.7; The boron-bound carbon was not detected due to quadrupolar relaxation. GCMS *m/z* (EI) calcd for $C_{16}H_{23}BO_2$ $(M)^+$: 258.18; Found: 258.30.

(*E*)-4,4,5,5-Tetramethyl-2-(4-phenylbut-1-en-1-yl)-1,3,2-dioxaborolane (11s″):

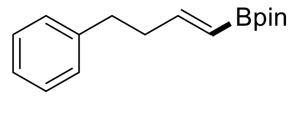

Colorless oil; ^1H NMR (500 MHz, $CDCl_3$) δ: 7.30 (t, *J* = 8.0 Hz, 2H), 7.22-7.19 (m, 3H), 6.77-6.71 (m, 1H), 5.54 (d, *J* = 18.0 Hz, 1H), 2.79-2.76 (m, 2H), 2.53-2.48 (m, 2H), 1.30 (s, 12H); $^{13}C\{^1H\}$ NMR (126 MHz, $CDCl_3$) δ: 153.3, 141.7, 128.3, 125.8, 83.0, 37.4, 34.5, 24.7; The boron-bound carbon was not detected due to quadrupolar relaxation. GCMS *m/z* (EI) calcd for $C_{16}H_{23}BO_2$ $(M)^+$: 258.18; Found: 258.30.

(*S*)-4,4,5,5-Tetramethyl-2-(1-phenyl-3-(3-(4,4,5,5-tetramethyl-1,3,2-dioxaborolan-2-yl)propoxy)propyl)-1,3,2-dioxaborolane (13):

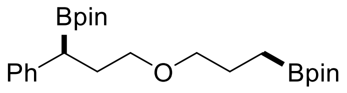

Colorless oil (98.4 mg, 76% yield); ^{1}H NMR (500 MHz, $CDCl_3$) δ: 7.24-7.18 (m, 4H), 7.12-7.08 (m, 1H), 3.41-3.26 (m, 4H), 2.43 (t, *J* = 8.0 Hz, 1H), 2.16-2.09 (m, 1H), 1.90-1.83 (m, 1H), 1.68-1.62 (m, 2H), 1.22 (s, 12H), 1.18 (s, 6H), 1.16 (s, 6H), 0.77 (t, *J* = 8.0 Hz, 2H),; ^{13}C{^{1}H} NMR (126 MHz, $CDCl_3$) δ:142.7, 128.3, 128.1, 125.1, 83.1, 82.8, 72.5, 69.5, 32.2, 24.7, 24.5 (Two signals overlap), 24.0; The boron-bound carbon was not detected due to quadrupolar relaxation. HRMS *m/z* (ESI) calcd for $C_{24}H_{40}B_2NaO_5$ $(M+Na)^+$: 453.2954; Found: 453.2966.

4,4,5,5-Tetramethyl-2-(1-phenylbutyl)-1,3,2-dioxaborolane (15):

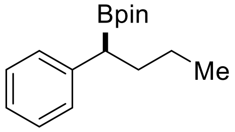

Colorless oil; ^{1}H NMR (500 MHz, $CDCl_3$) δ: 7.27-7.21 (m, 4H), 7.15-7.12 (m, 1H), 2.33 (t, *J* = 8.0 Hz, 1H), 1.87-1.80 (m, 1H), 1.69-1.61 (m, 1H), 1.34-1.28 (m, 2H), 1.22 (s, 6H), 1.20 (s, 6H), 0.90 (t, *J* = 7.5 Hz, 3H); ^{13}C{^{1}H} NMR (126 MHz, $CDCl_3$) δ: 132.4, 117.3, 117.2, 114.0, 72.2, 23.8, 13.6, 13.5, 11.3, 3.1; The boron-bound carbon was not detected due to quadrupolar relaxation. GCMS *m/z* (EI) calcd for $C_{16}H_{25}BO_2$ $(M)^+$: 260.18; Found: 260.20.

4,4,5,5-Tetramethyl-2-(4-phenylbutyl)-1,3,2-dioxaborolane (16):

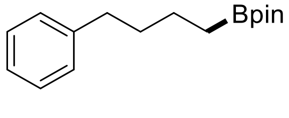

Colorless oil; ^{1}H NMR (500 MHz, $CDCl_3$) δ: 7.32-7.27 (m, 2H), 7.22-7.18 (m, 3H), 2.65 (t, *J* = 8.0 Hz, 2H), 1.70-1.64 (m, 2H), 1.55-1.49 (m, 2H), 1.28 (s, 12H), 0.86 (t, *J* = 8.0 Hz, 2H); ^{13}C{^{1}H} NMR (126 MHz, $CDCl_3$) δ: 142.9, 128.3, 128.1, 125.5, 82.8, 35.7, 34.1, 24.8, 23.7; The

boron-bound carbon was not detected due to quadrupolar relaxation. GCMS *m/z* (EI) calcd for $C_{16}H_{25}BO_2$ $(M)^+$: 260.19; Found: 260.15.

2,2'-(Butane-1,1-diyl)bis(4,4,5,5-tetramethyl-1,3,2-dioxaborolane) (20):

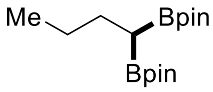

Colorless oil ; ^{1}H NMR (400 MHz, $CDCl_3$) δ: 1.53-1.48 (m, 2H), 1.32-1.25 (m, 2H), 1.21 (s, 12H), 1.20 (s, 12H), 0.85 (t, *J* = 7.2 Hz, 3H), 0.71 (t, *J* = 8.0 Hz, 1H); ^{13}C{^{1}H} NMR (101 MHz, $CDCl_3$) δ: 82.8, 27.9, 25.5, 24.8, 24.5, 14.1; The boron-bound carbon was not detected due to quadrupolar relaxation. GCMS *m/z* (EI) calcd for $C_{16}H_{32}B_2O_4$ $(M)^+$: 310.25; Found: 310.20.

Procedures for deuterium-labeling experiments

(i) Deuterium-labeling experiments

DBpin was prepared according to a previously reported literature.[1] The reaction was conducted in accordance with general procedure for double hydroboration of 1,3-diene. The ^{2}H NMR spectroscopy was measured with $CDCl_3$ (2 equiv) as an internal standard.

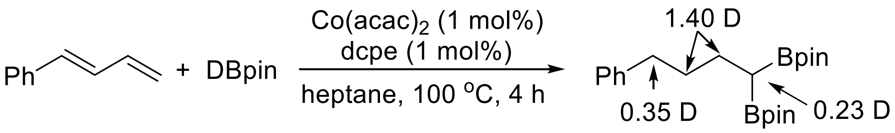

Supplementary Equation 3. Reaction of **1s** with DBpin.

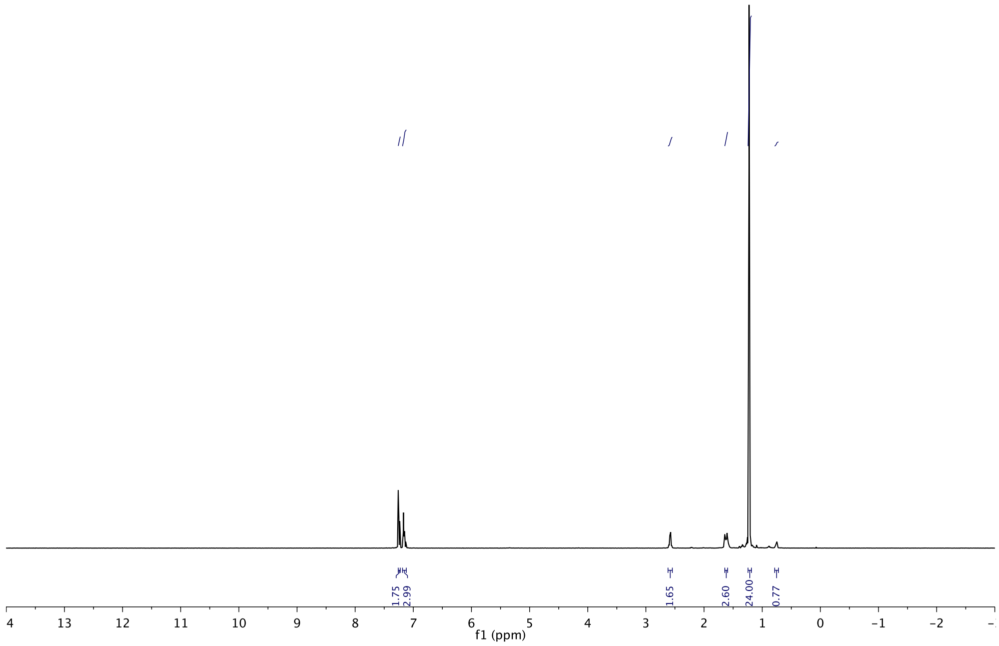

Supplementary Figure 1. ^{1}H NMR of the product from the reaction of **1s** with DBpin.

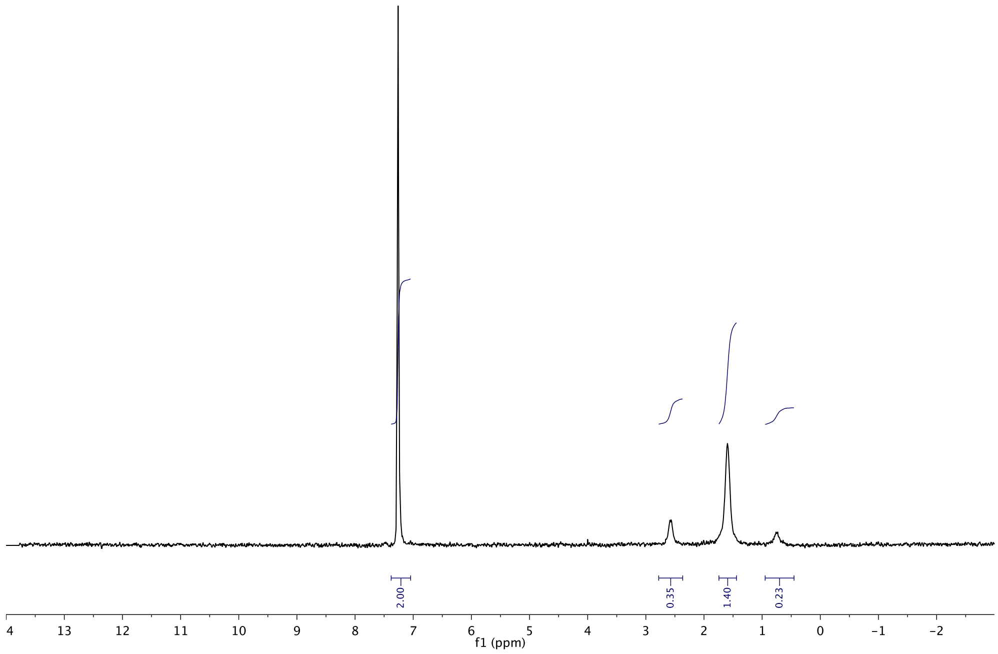

Supplementary Figure 2. ^{2}D NMR of the product from the reaction of **1s** with DBpin.

(E)-(buta-1,3-dien-1-yl-4,4-d₂)benzene synthesis.[12]

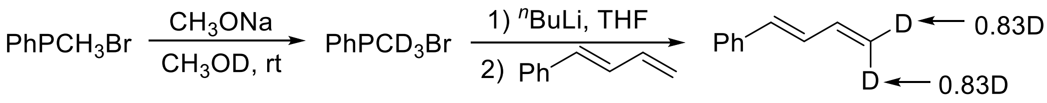

Supplementary Equation 4. Synthesis of **1s**-d_2.

Under the Ar atmosphere, the compounds of Ph_3PCH_3Br (5 mmol) and CH_3ONa (0.27 mmol) dissolved in CH_3OD (2 mL). After the mixture was stirred for 1h, the solvent was removed under vacuum. Then another fresh CH_3OD (2 mL) was added. The same procedure was repeated for three to four times to afford Ph_3PCD_3Br.

To asolution of deuterium Ph_3PCD_3Br (4.5 mmol) in dry THF (5mL) under Ar atmosphere, *n*-BuLi was drop wisely added at -78 °C. After 30 min, the solution of cinnamaldehyde (3 mmol) in dry THF (1 mL) was added, then allowing the temperature to room temperature for three hours. A saturated solution of NH_4Cl (10 mL) was added and the mixture was extracted with Et_2O (3 × 10 mL). The combined organic extracts were dried over Na_2SO_4 and concentrated in vacuum. The crude compound was purified by silica gel flash column chromatography (hexane) to attain the (*E*)-(buta-1,3-dien-1-yl-4,4-d_2)benzene.

The reaction was conducted in accordance with general procedure for double hydroboration of (*E*)-(buta-1,3-dien-1-yl-4,4-d_2)benzene with HBpin. The ^2H NMR spectroscopy was measured with $CDCl_3$ (2 equiv) as an internal standard.

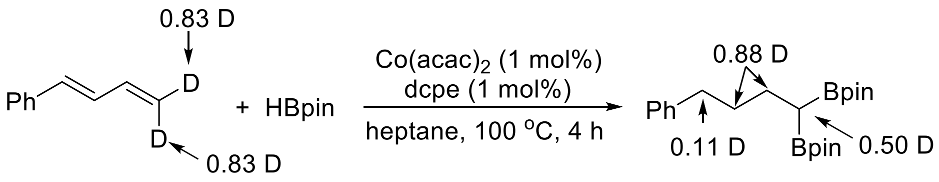

Supplementary Equation 5. Reaction of **1s**-d_2 with HBpin.

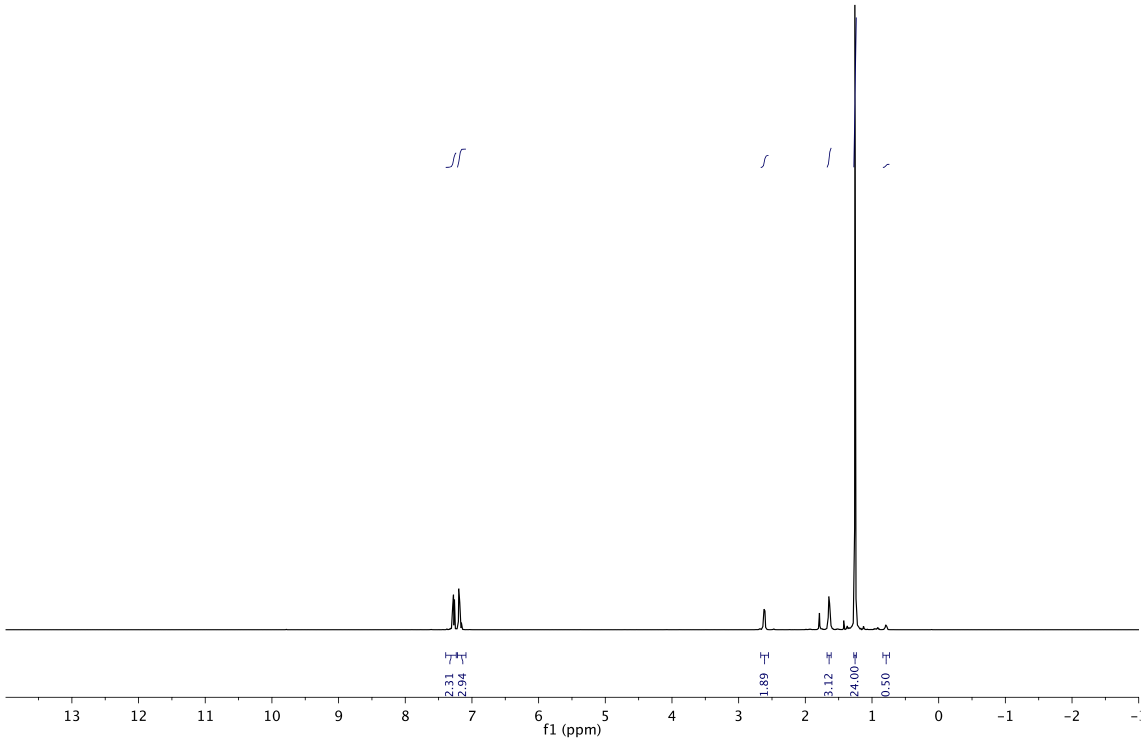

Supplementary Figure 3. ^{1}H NMR of the product from the reaction of **1s**-d_2 with HBpin.

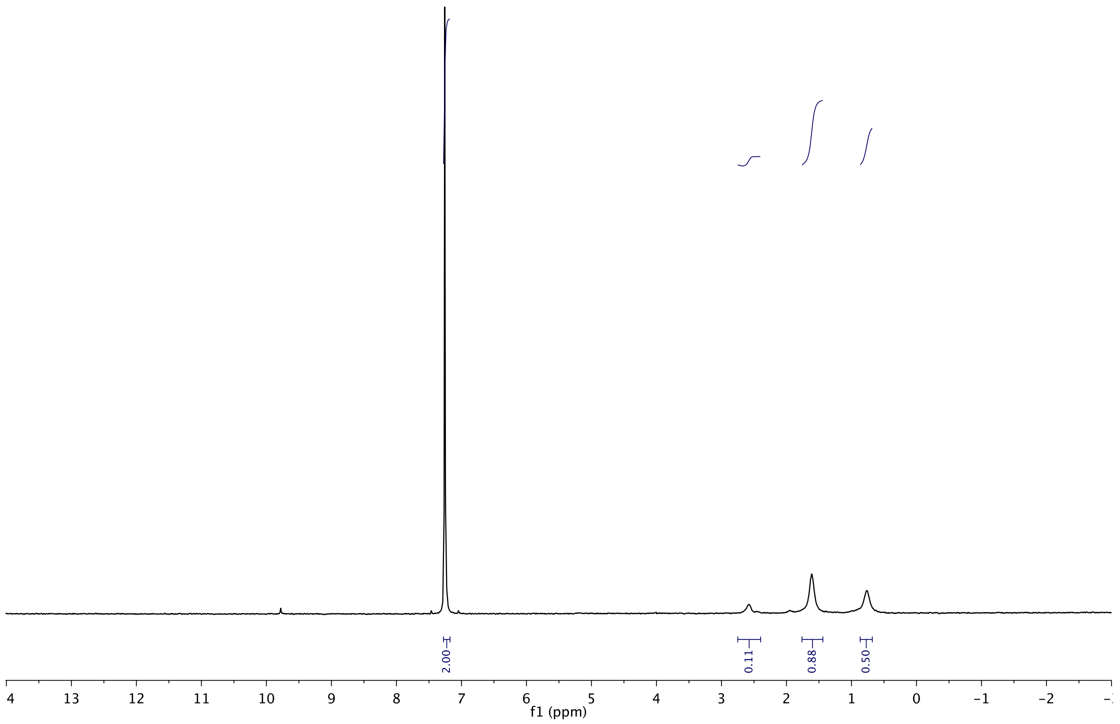

Supplementary Figure 4. ^{2}D NMR of the product from the reaction of **1s-**d_2 with HBpin.

(ii) Crossover experiment

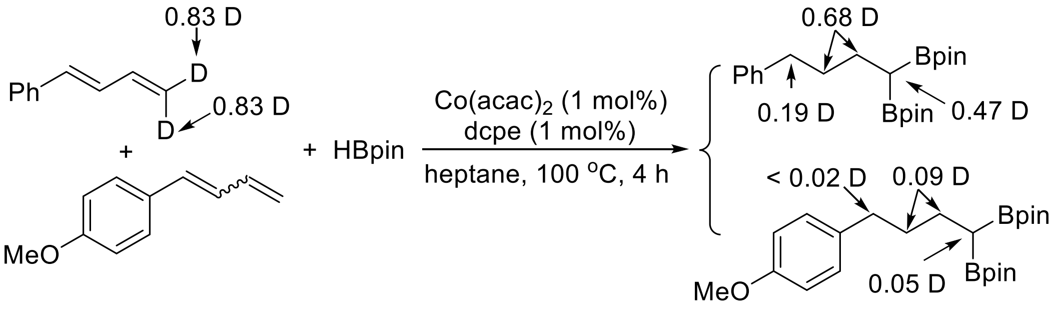

Supplementary Equation 6. Cross-over experiment of **1s-**d_2 and **1x**.

a) The ^{2}H NMR spectroscopy was measured with $CDCl_3$ (5 equiv) as an internal standard.

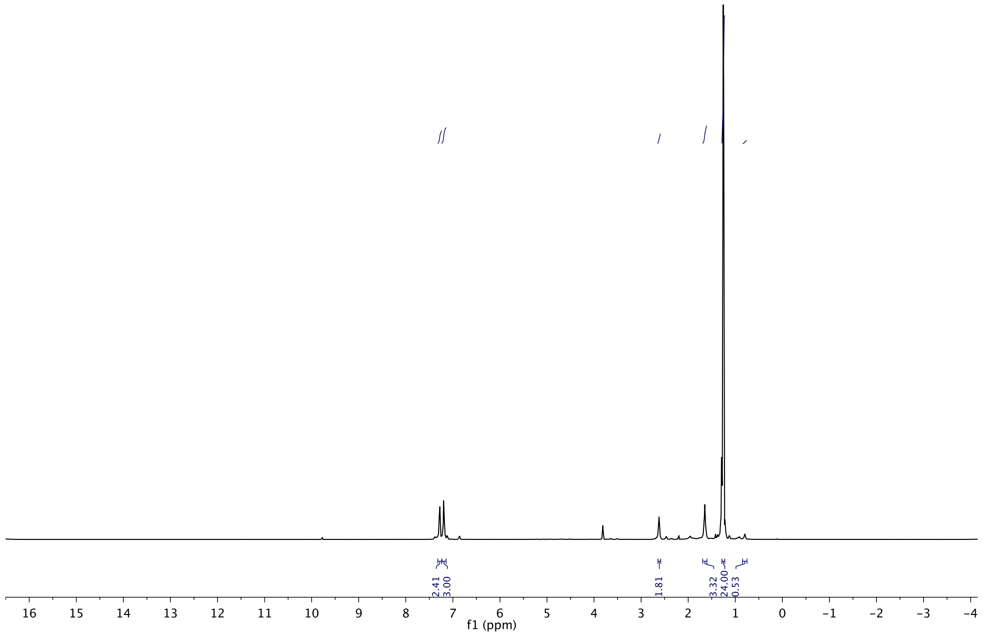

Supplementary Figure 5. ^{1}H NMR of the product from cross-over reactions (**1a-**d_2 with HBpin.)

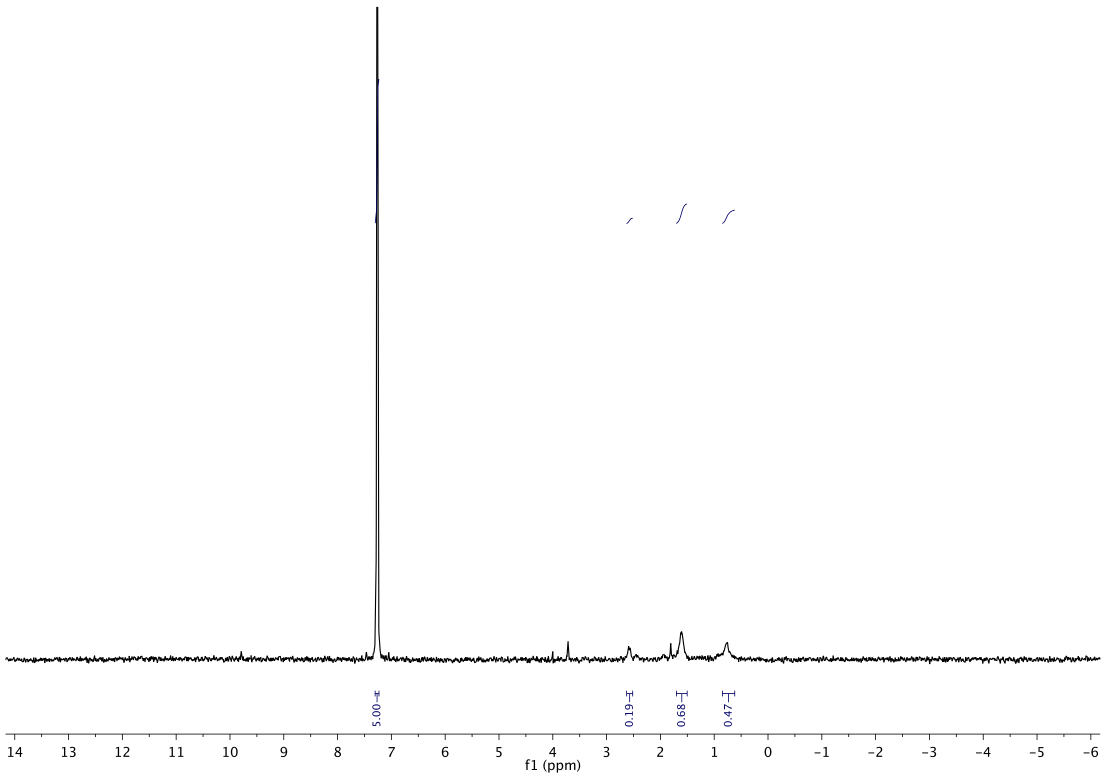

Supplementary Figure 6. ^{2}D NMR of the product from cross-over reactions (**1a-**d_2 with HBpin.)

b) The ^{2}H NMR spectrum was measured with $CDCl_3$ (1.5 equiv) as an internal standard.

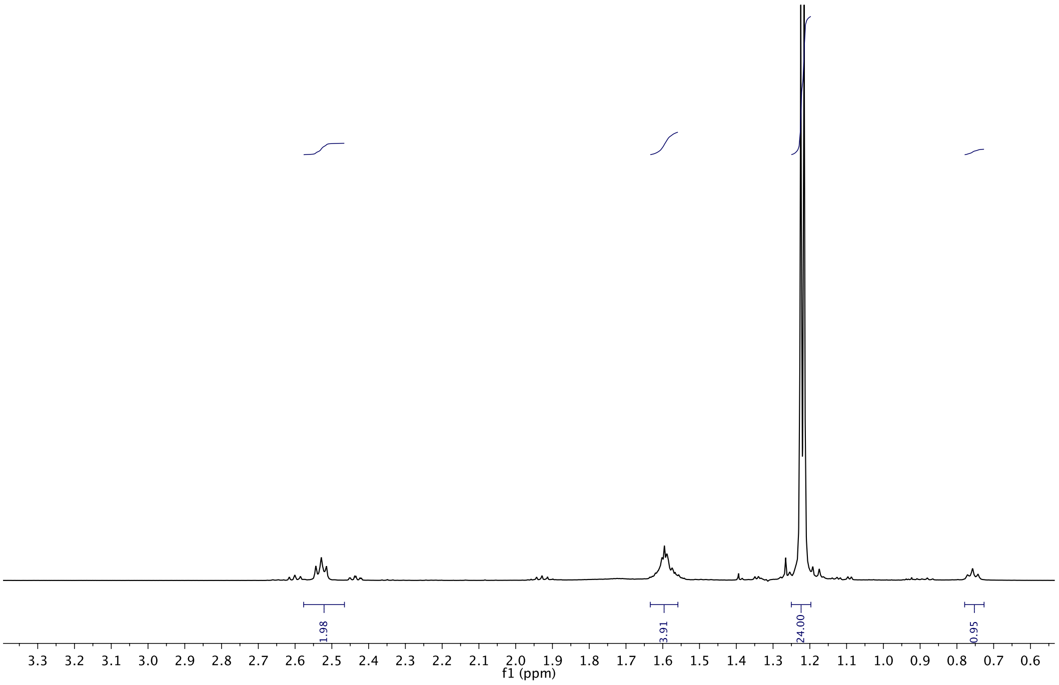

Supplementary Figure 7. ^{1}H NMR of the product from cross-over reactions (**1x** with HBpin.)

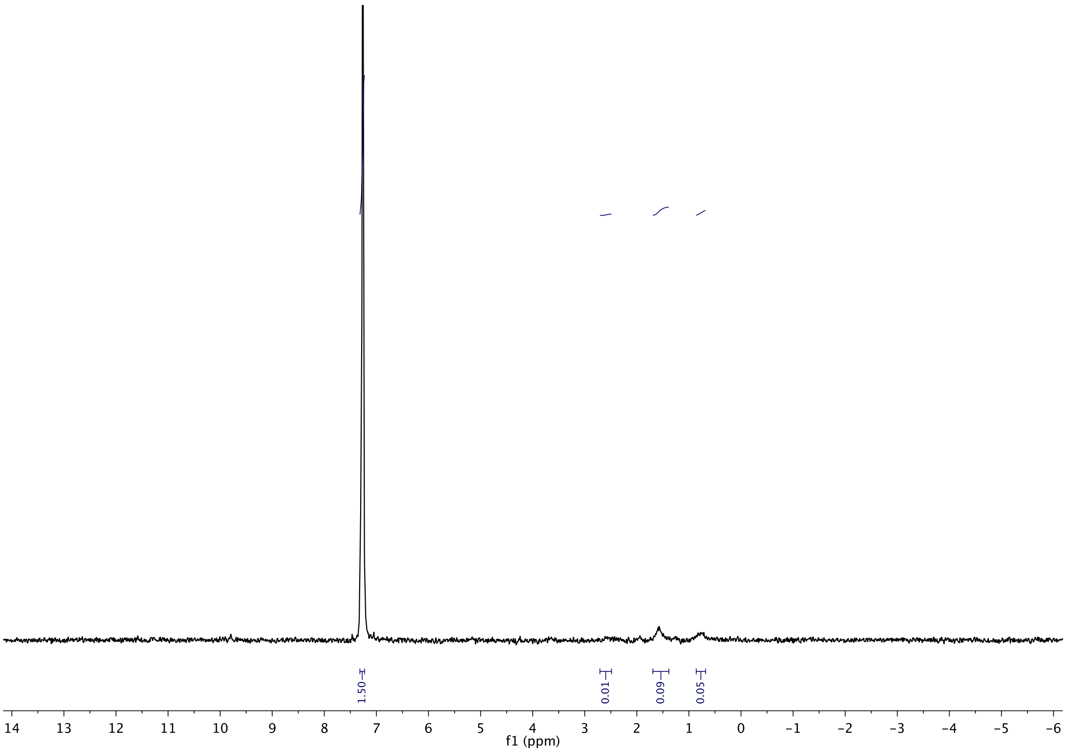

Supplementary Figure 8. ^{2}D NMR of the product from cross-over reactions (**1x** with HBpin.)

Copies of ^{1}H and $^{13}C\{^{1}H\}$ NMR spectra

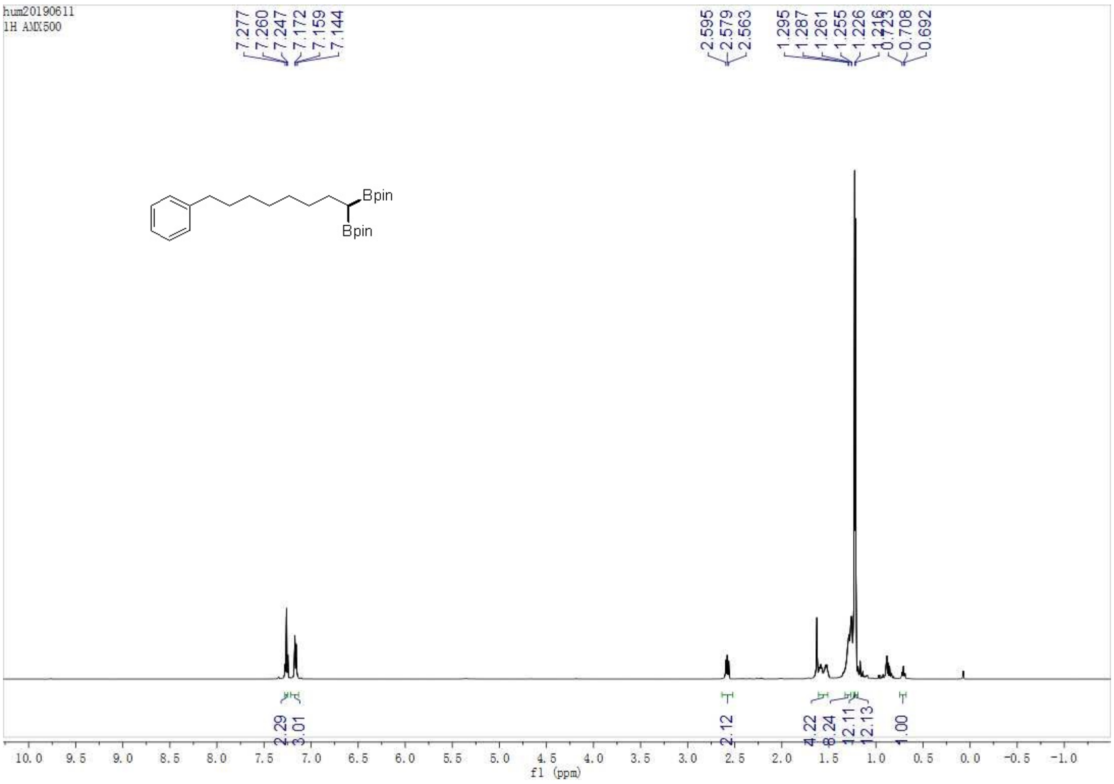

Supplementary Figure 9. ^{1}H NMR spectrum of compound **2a**.

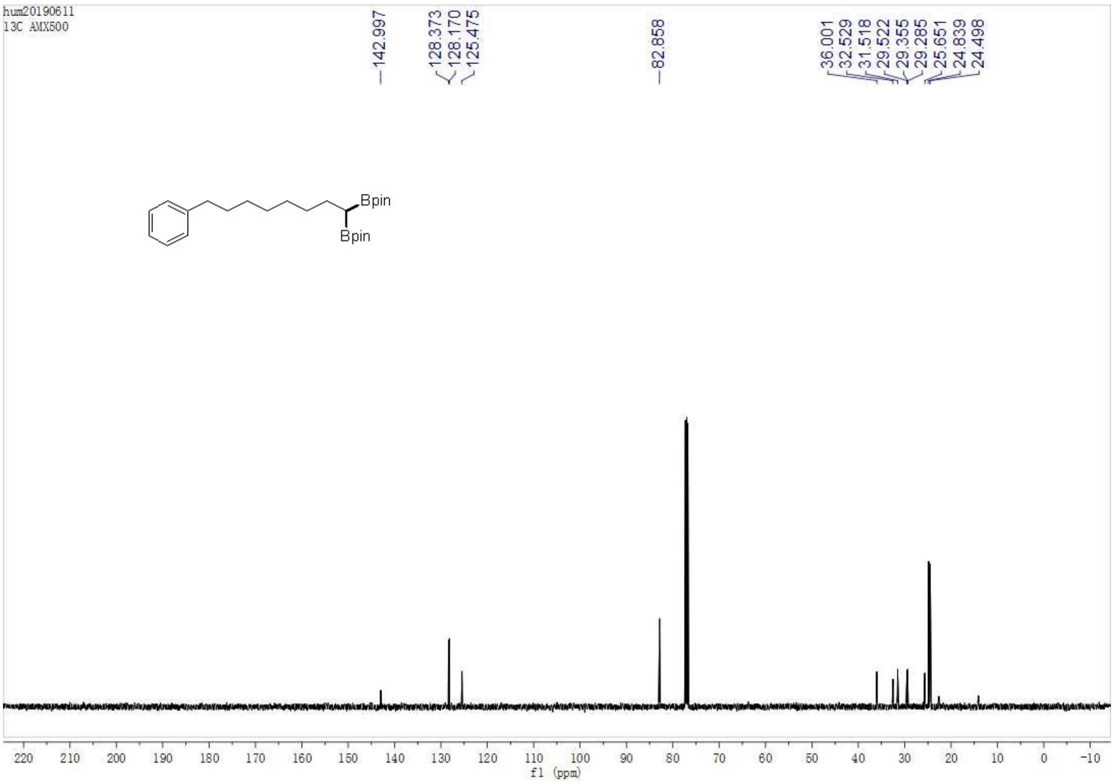

Supplementary Figure 10. $^{13}C\{^{1}H\}$ NMR spectrum of compound **2a**.

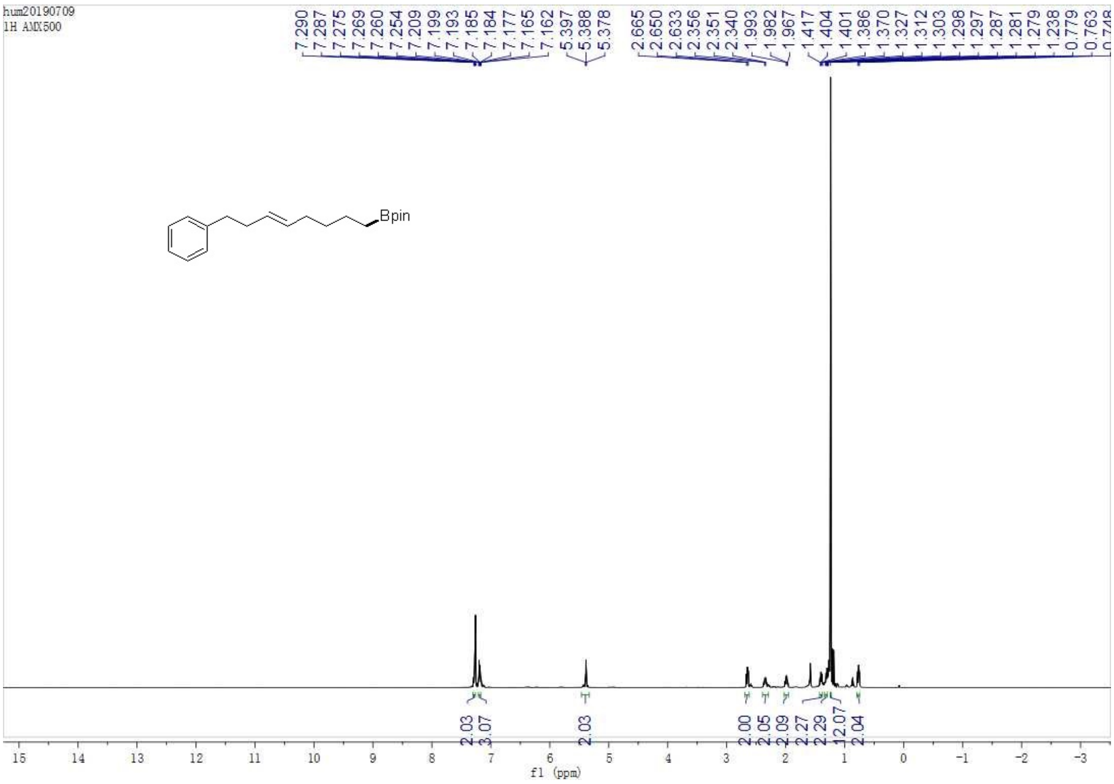

Supplementary Figure 11. ^{1}H NMR spectrum of compound **2a’**.

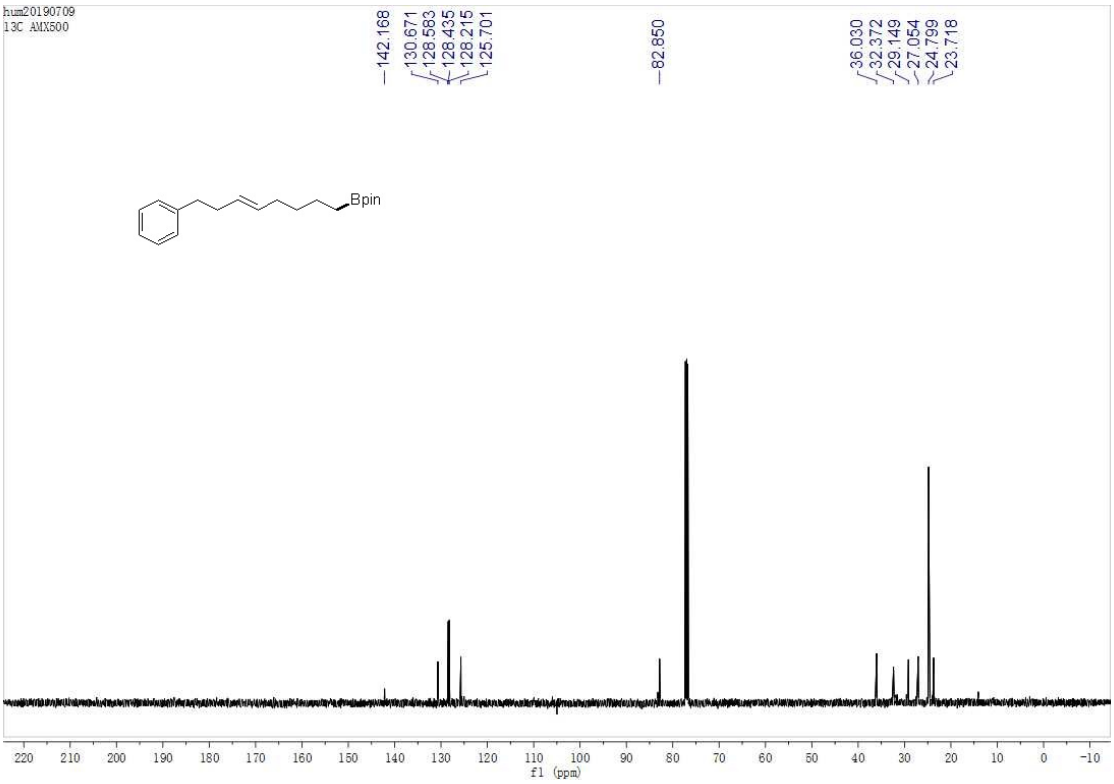

Supplementary Figure 12. ^{13}C{^{1}H} NMR spectrum of compound **2a’**.

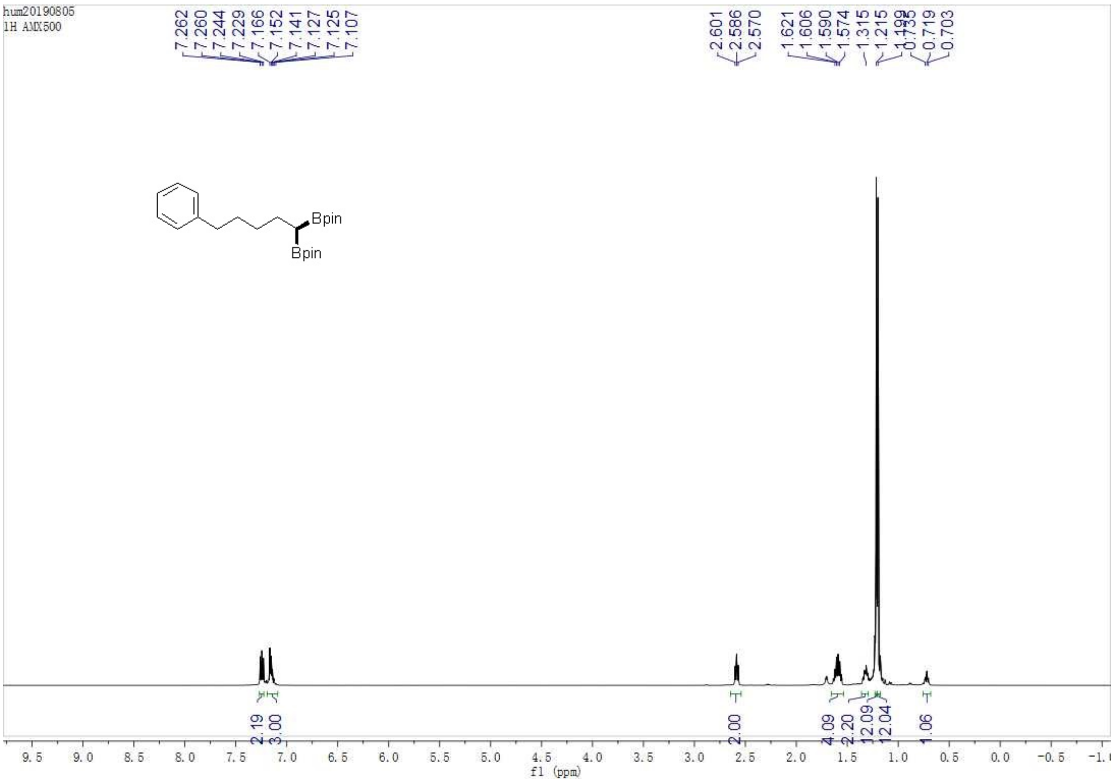

Supplementary Figure 13. ^{1}H NMR spectrum of compound **2b**.

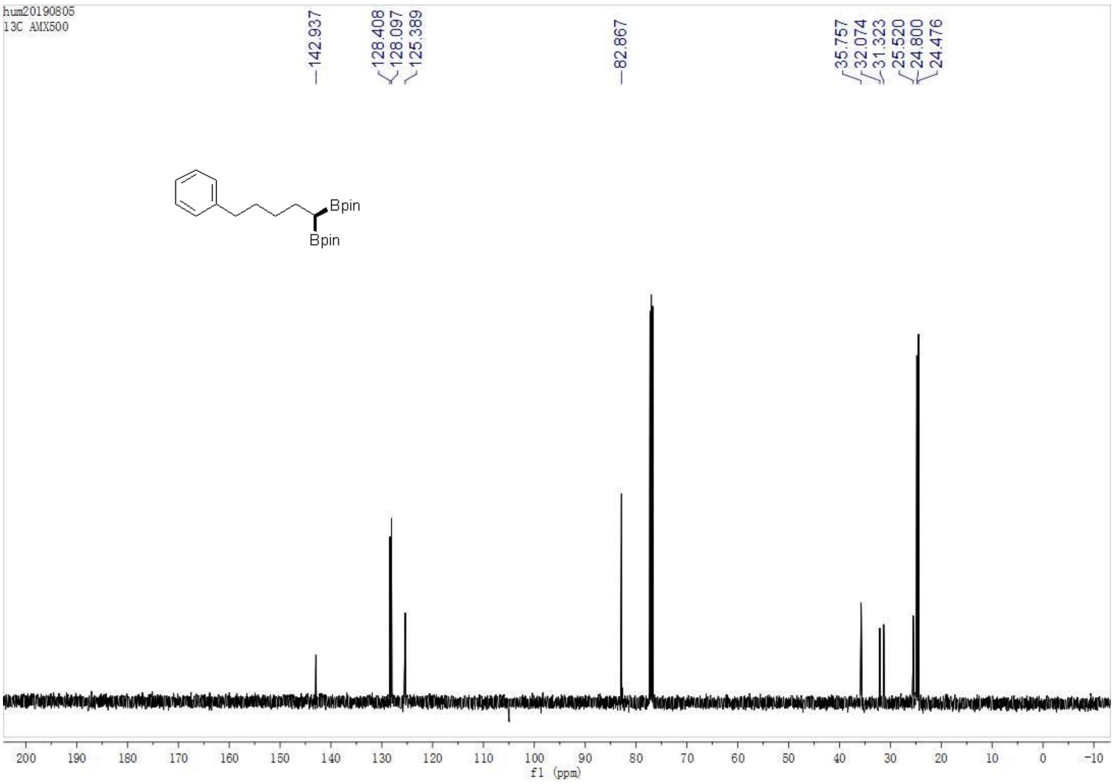

Supplementary Figure 14. $^{13}C\{^{1}H\}$ NMR spectrum of compound **2b**.

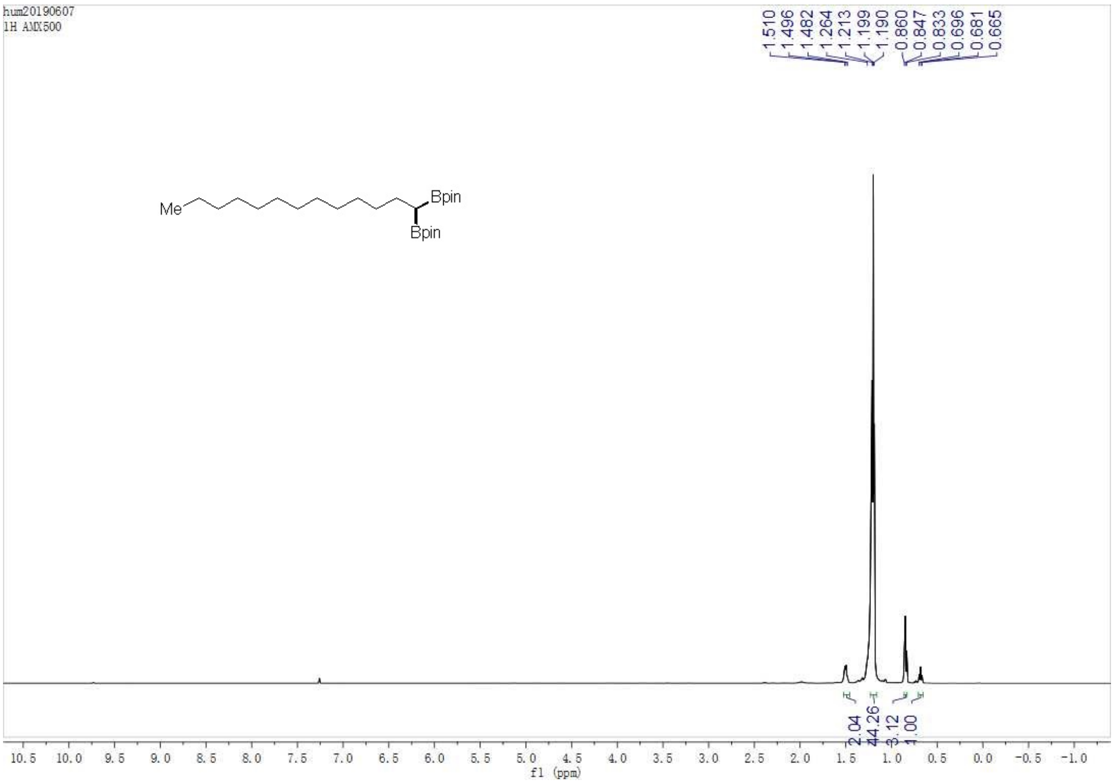

Supplementary Figure 15. ^{1}H NMR spectrum of compound **2c**.

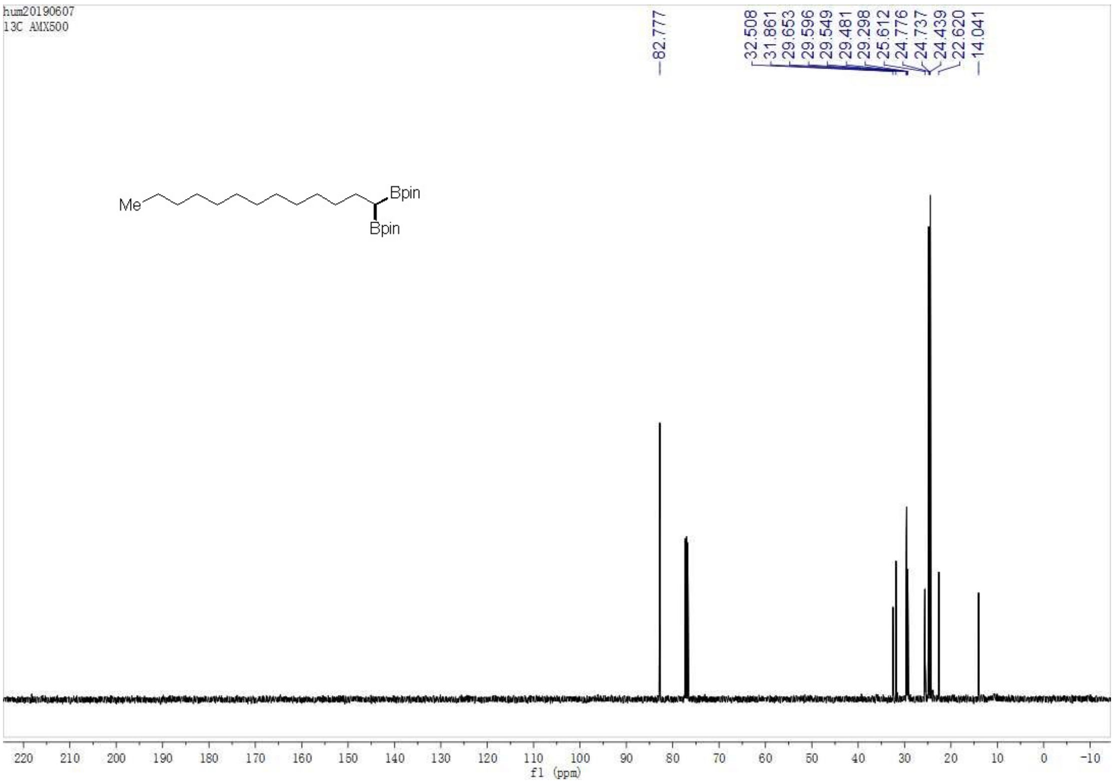

Supplementary Figure 16. $^{13}C\{^{1}H\}$ NMR spectrum of compound **2c**.

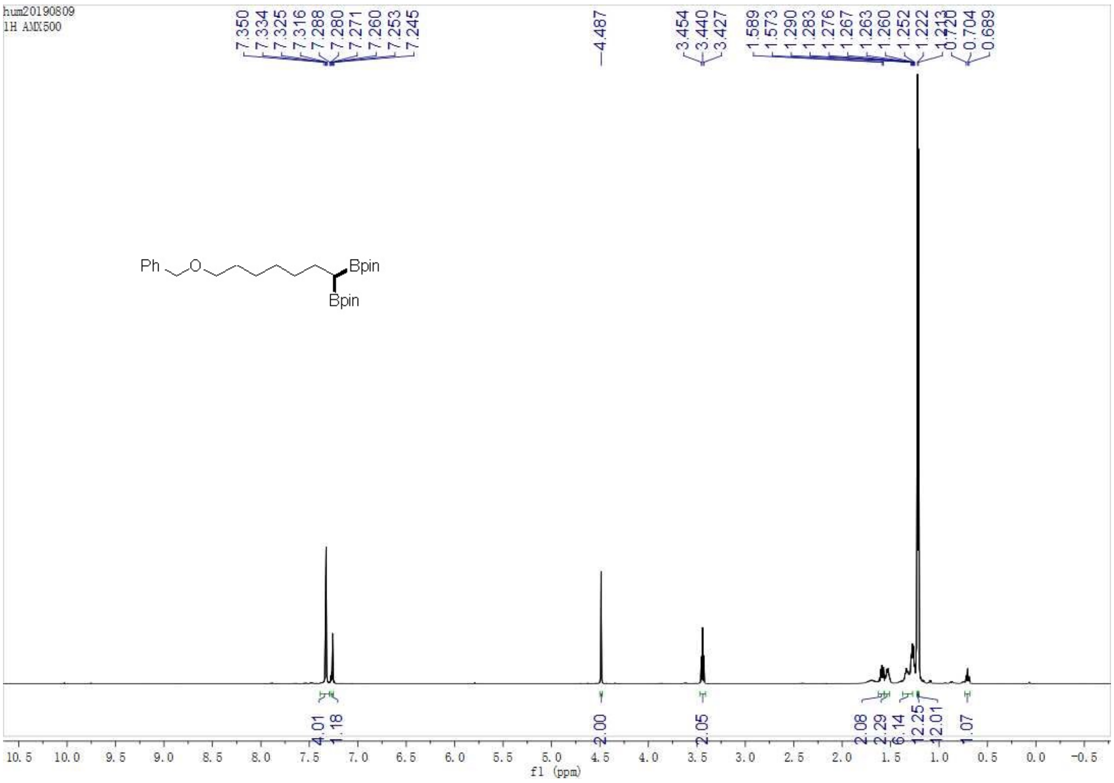

Supplementary Figure 17. ^{1}H NMR spectrum of compound **2d**.

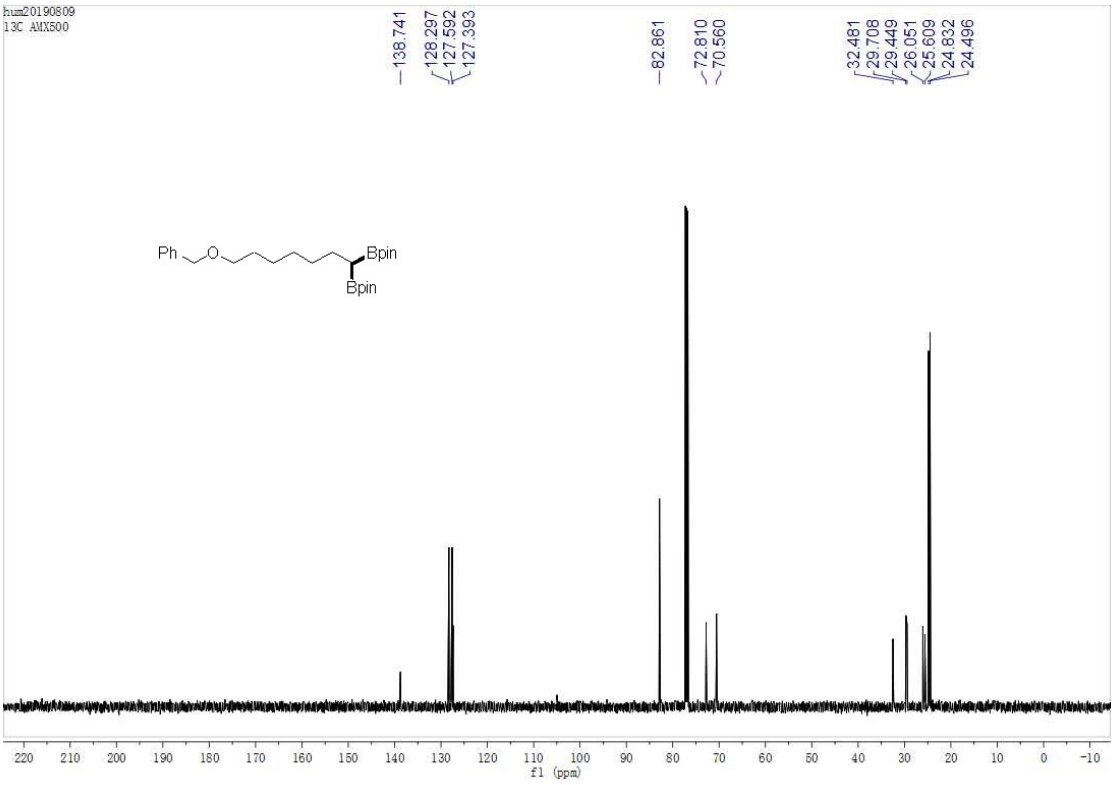

Supplementary Figure 18. ^{13}C{^{1}H} NMR spectrum of compound **2d**.

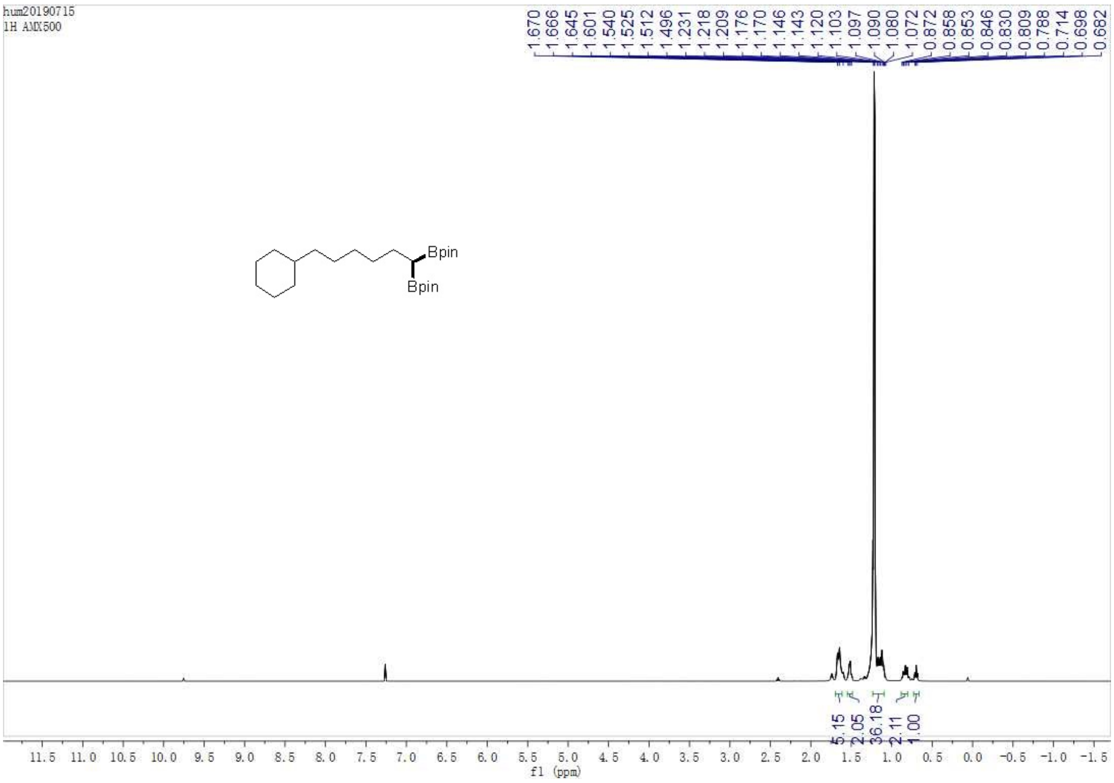

Supplementary Figure 19. ^{1}H NMR spectrum of compound **2e**.

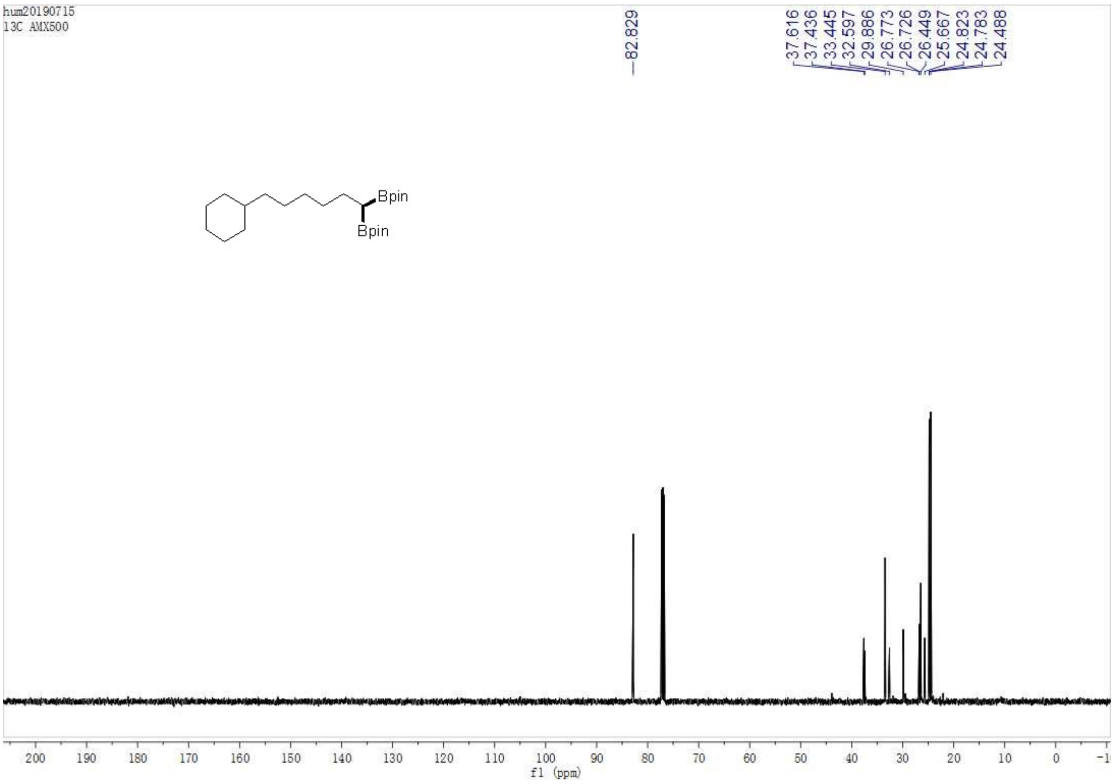

Supplementary Figure 20. $^{13}C\{^{1}H\}$ NMR spectrum of compound **2e**.

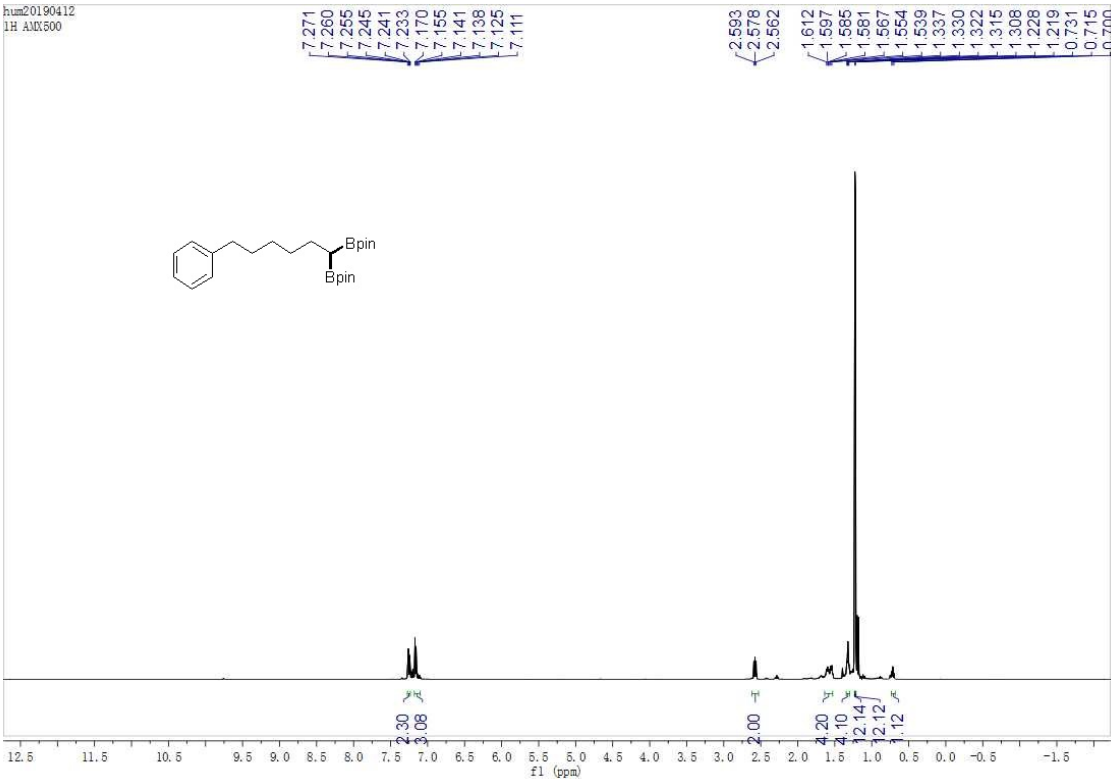

Supplementary Figure 21. ^{1}H NMR spectrum of compound **2f**.

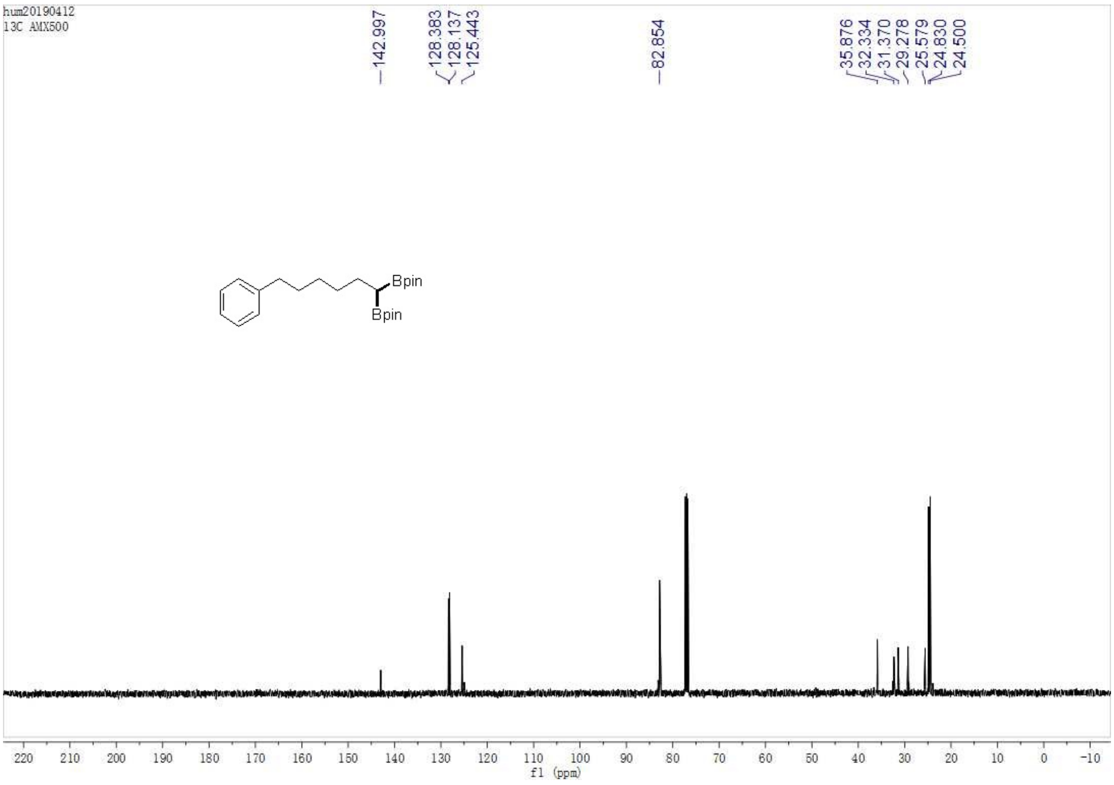

Supplementary Figure 22. $^{13}C\{^{1}H\}$ NMR spectrum of compound **2f**.

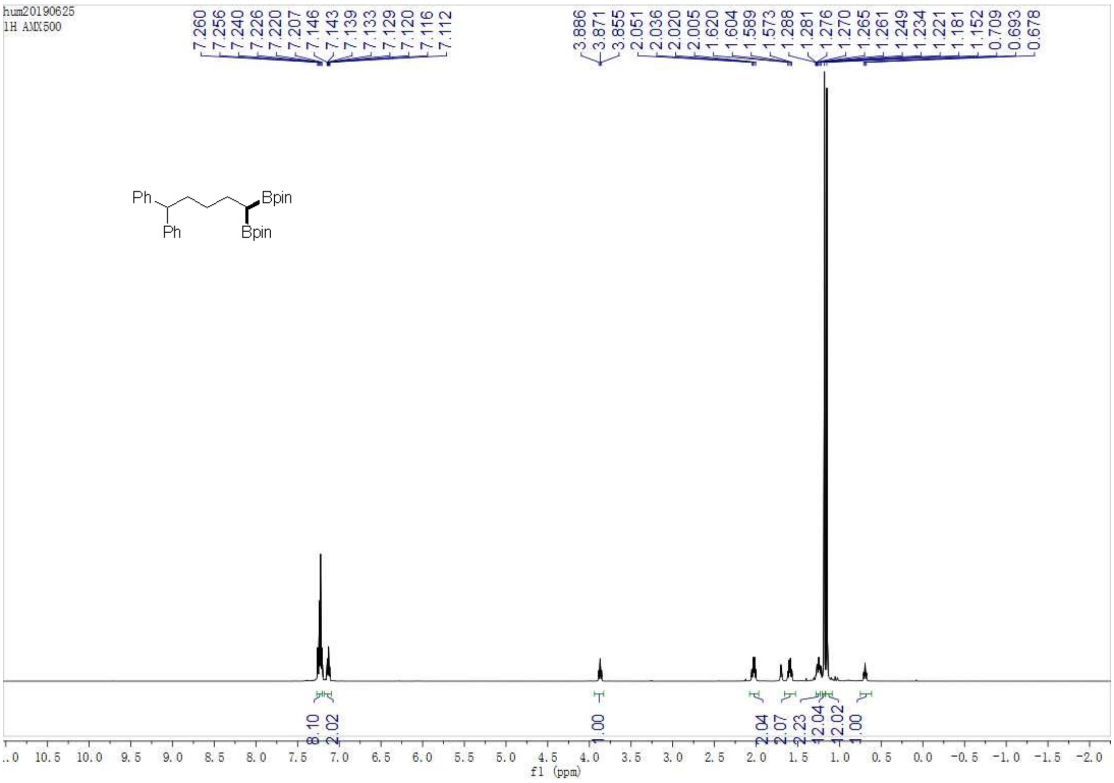

Supplementary Figure 23. ^{1}H NMR spectrum of compound **2g**.

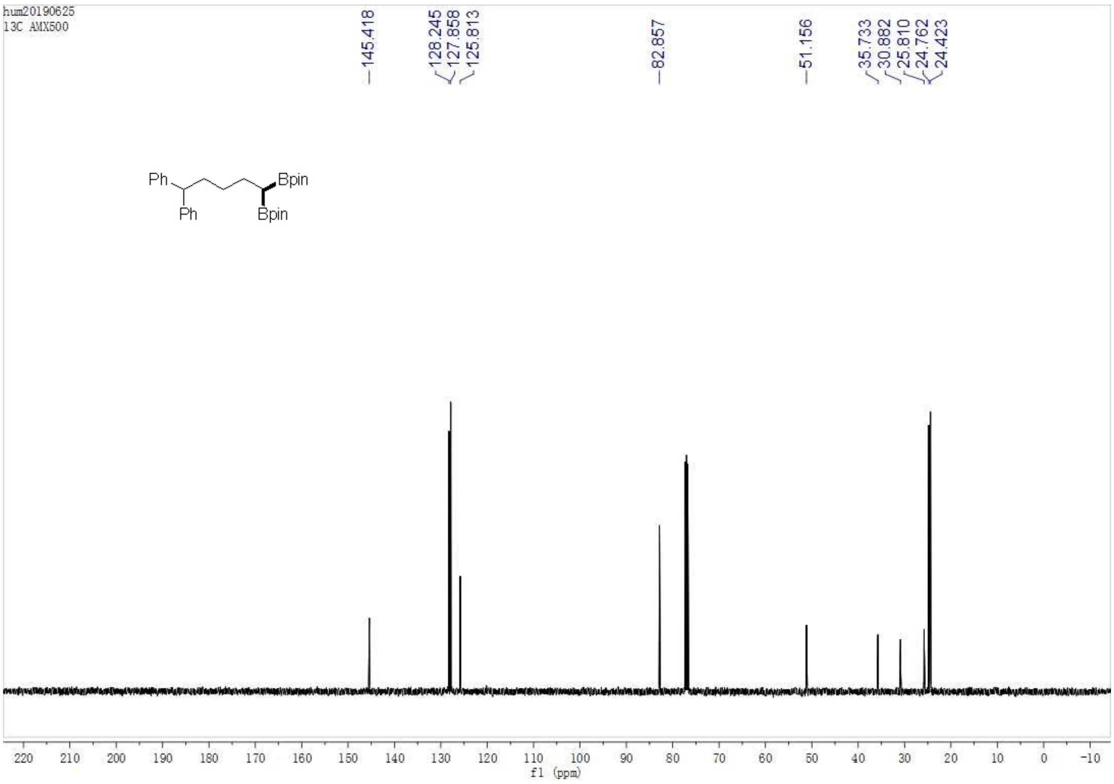

Supplementary Figure 24. $^{13}C\{^{1}H\}$ NMR spectrum of compound **2g**.

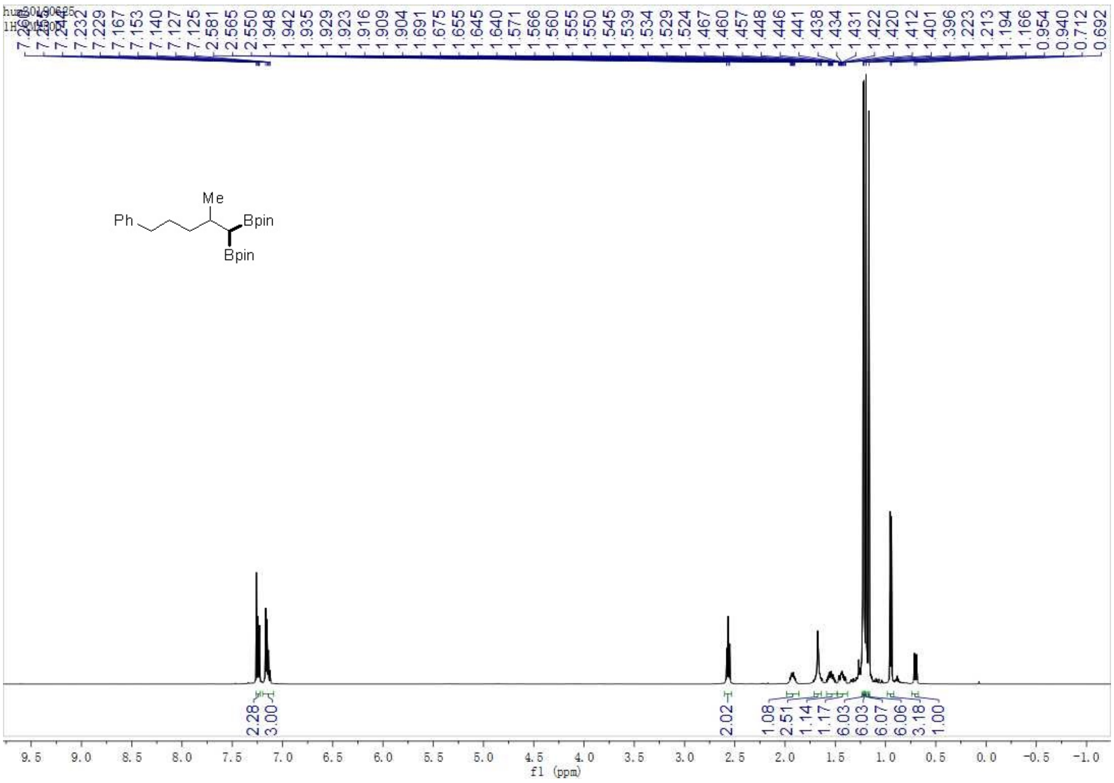

Supplementary Figure 25. ^{1}H NMR spectrum of compound **2h**.

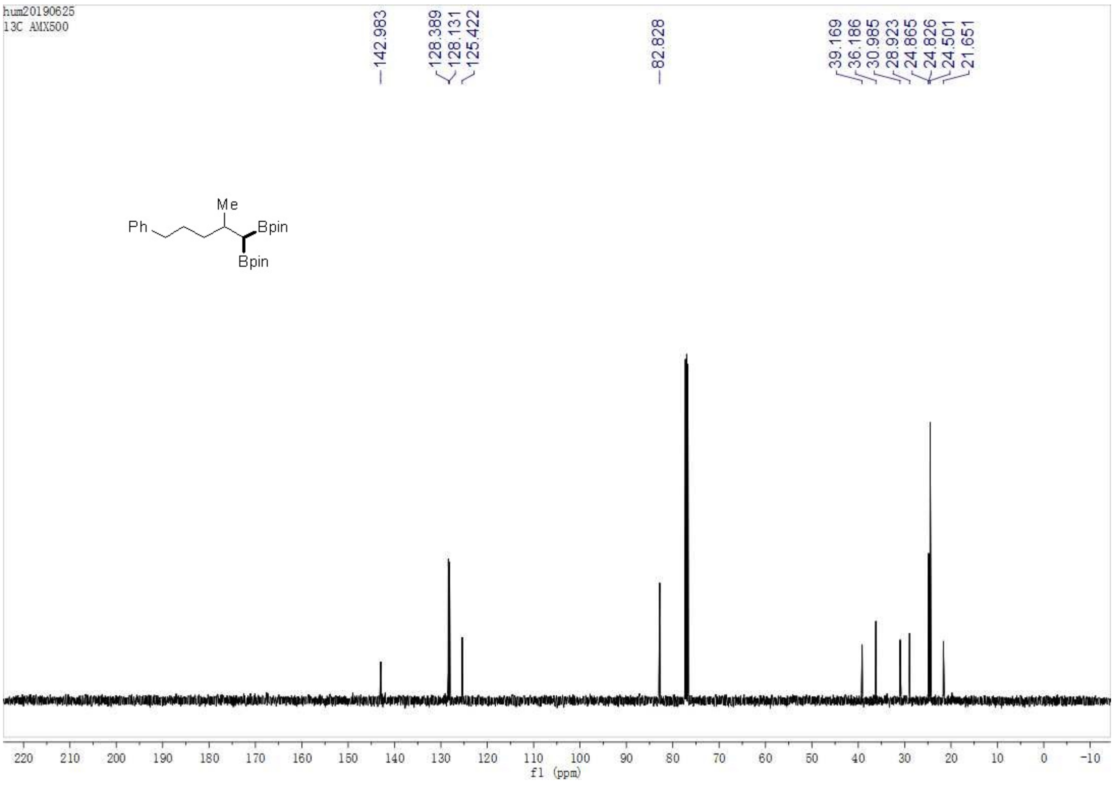

Supplementary Figure 26. $^{13}C\{^{1}H\}$ NMR spectrum of compound **2h**.

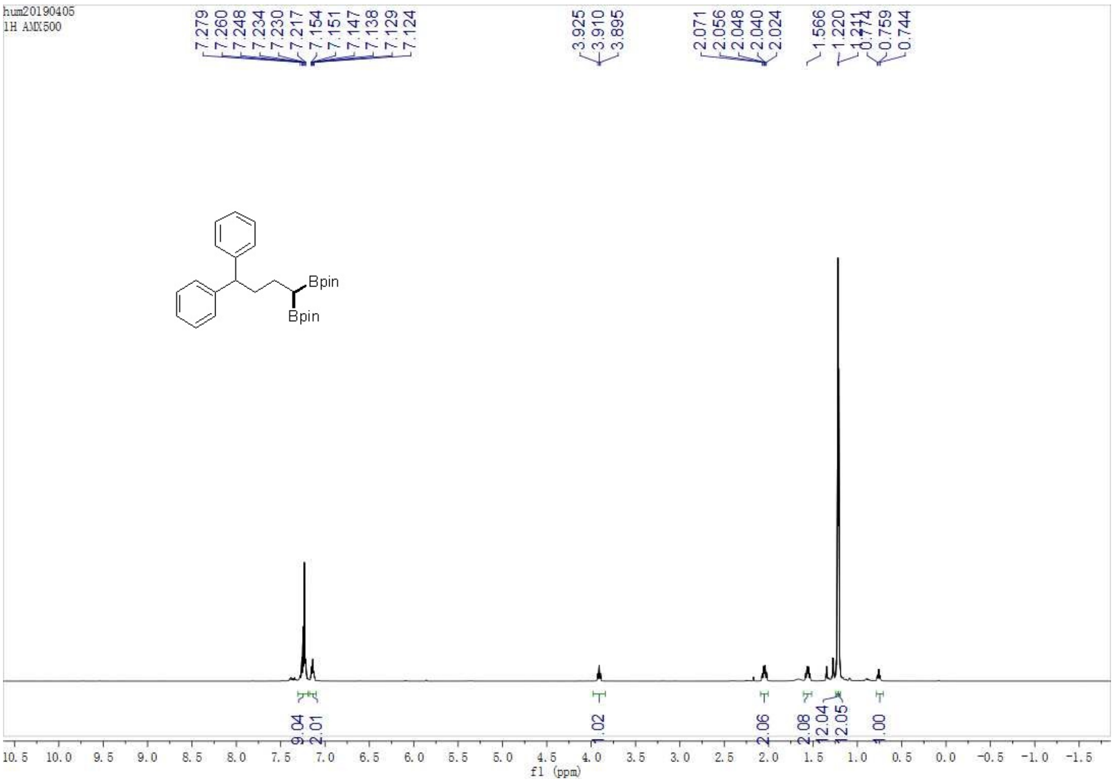

Supplementary Figure 27. ^{1}H NMR spectrum of compound **2i**.

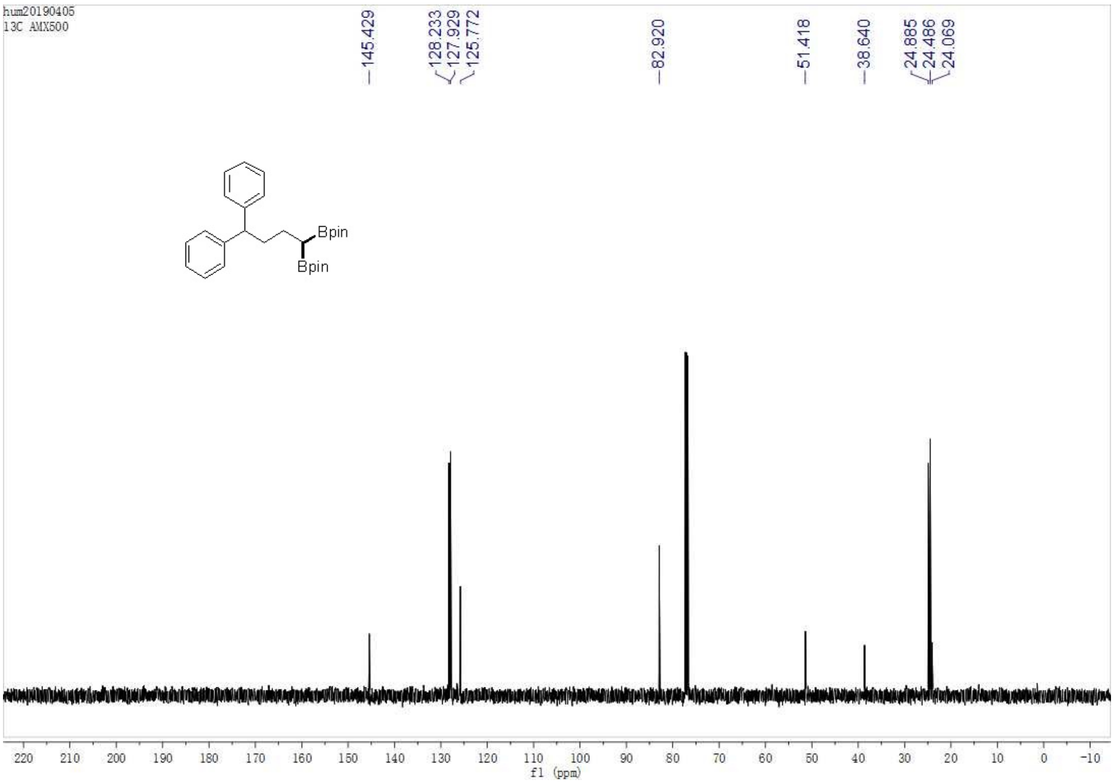

Supplementary Figure 28. $^{13}C\{^{1}H\}$ NMR spectrum of compound **2i**.

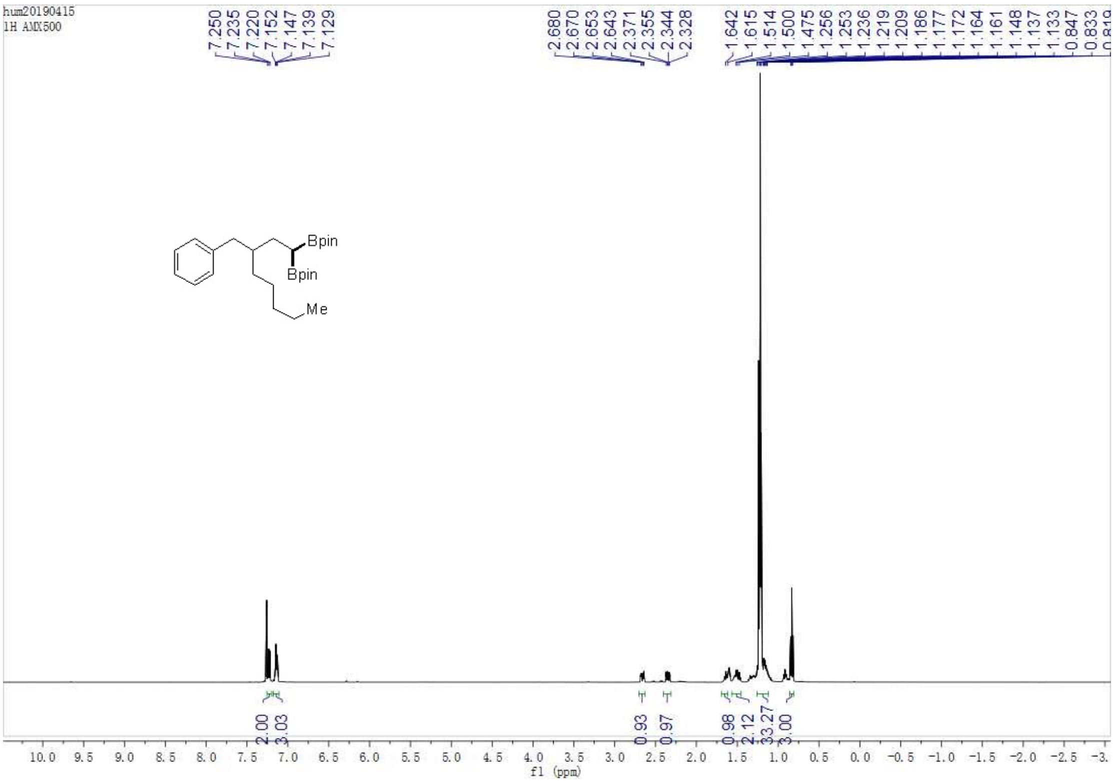

Supplementary Figure 29. ^{1}H NMR spectrum of compound **2j**.

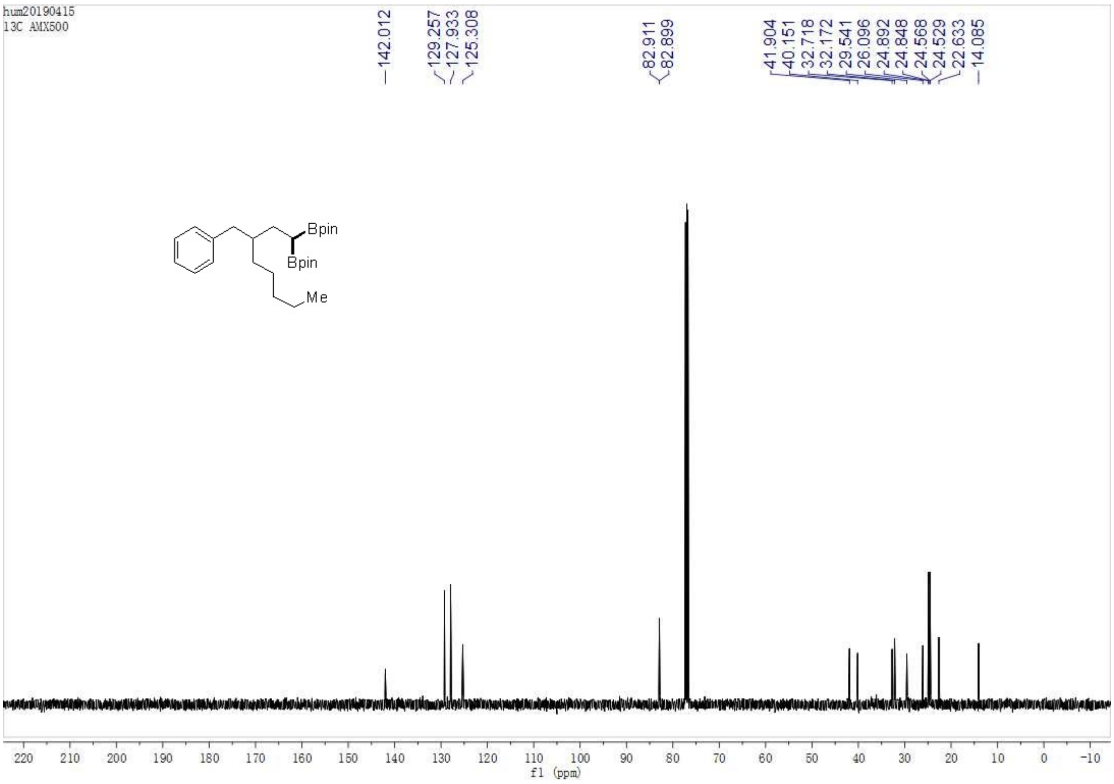

Supplementary Figure 30. $^{13}C\{^{1}H\}$ NMR spectrum of compound **2j**.

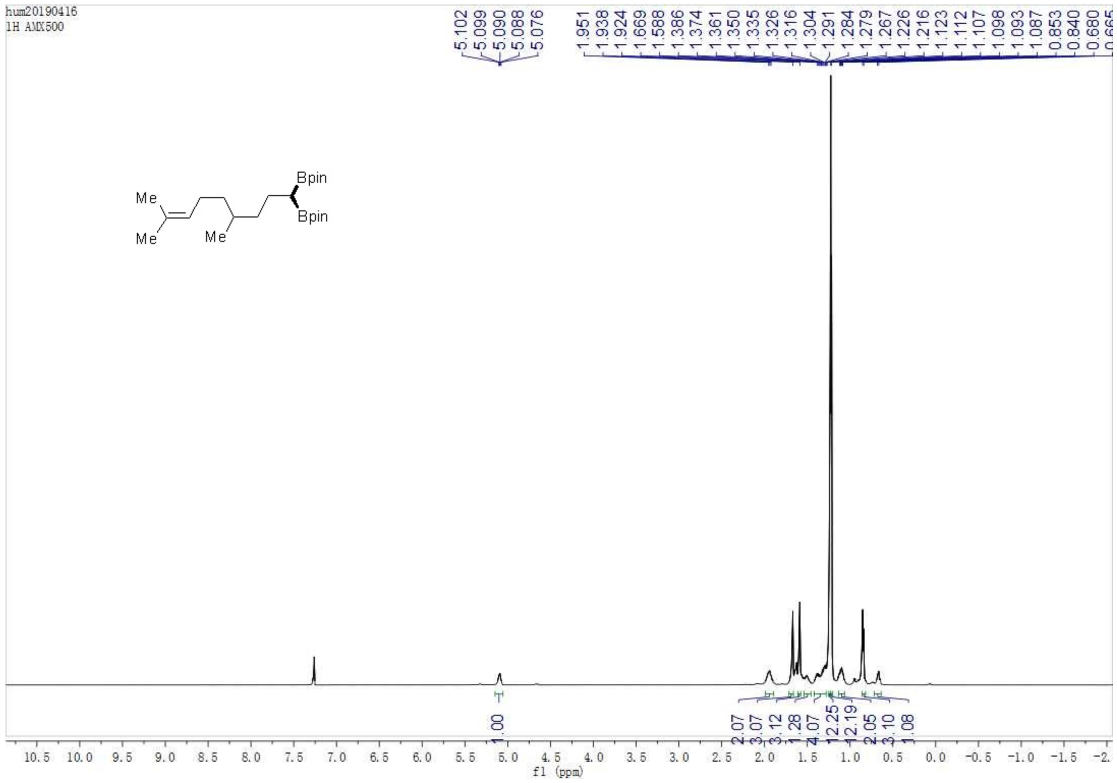

Supplementary Figure 31. ^{1}H NMR spectrum of compound **2k**.

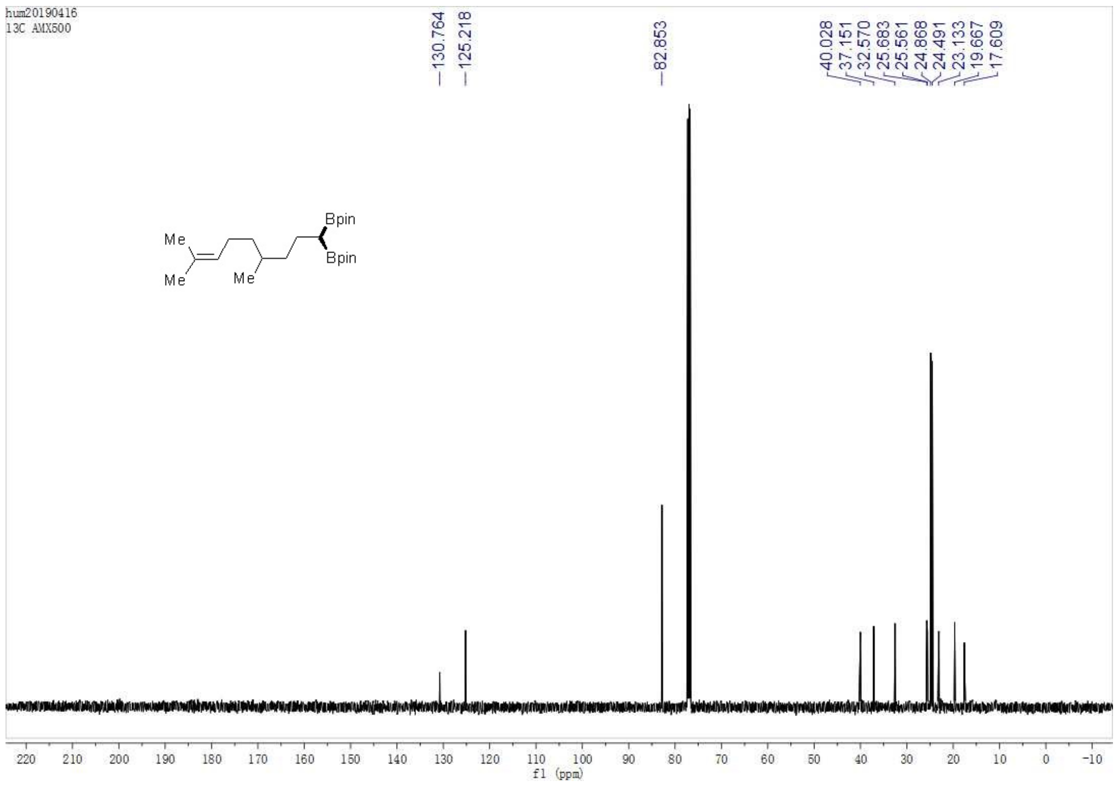

Supplementary Figure 32. $^{13}C\{^{1}H\}$ NMR spectrum of compound **2k**.

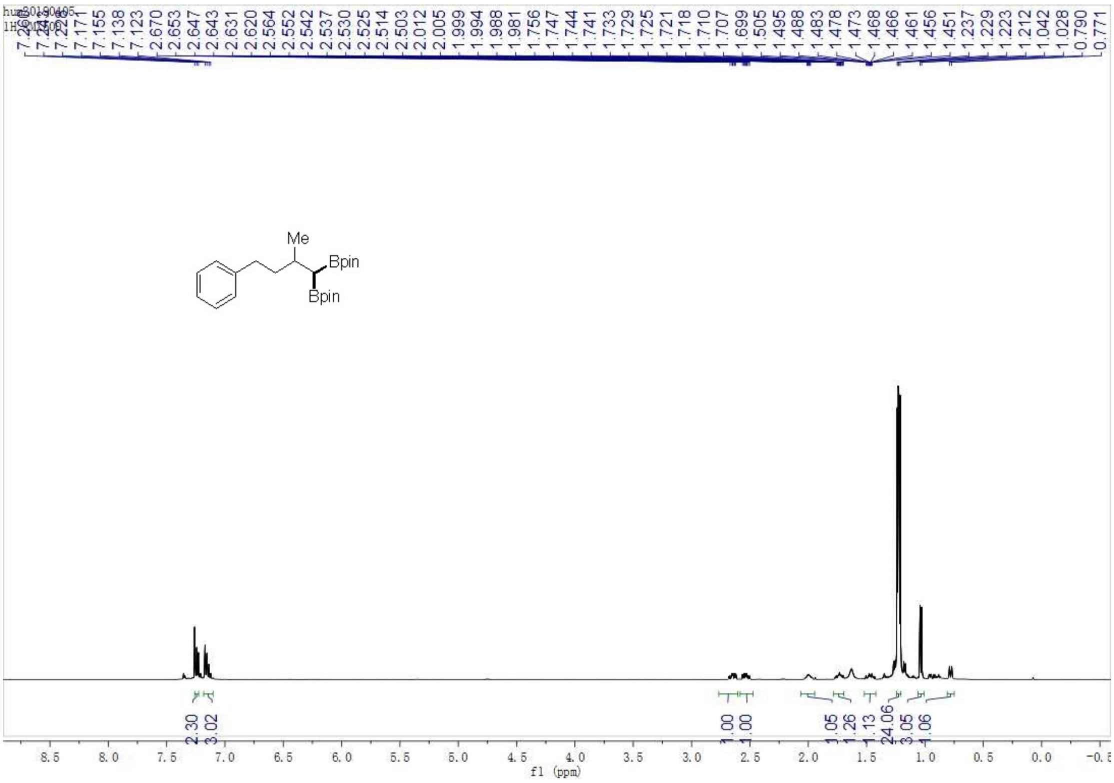

Supplementary Figure 33. ^{1}H NMR spectrum of compound **2l**.

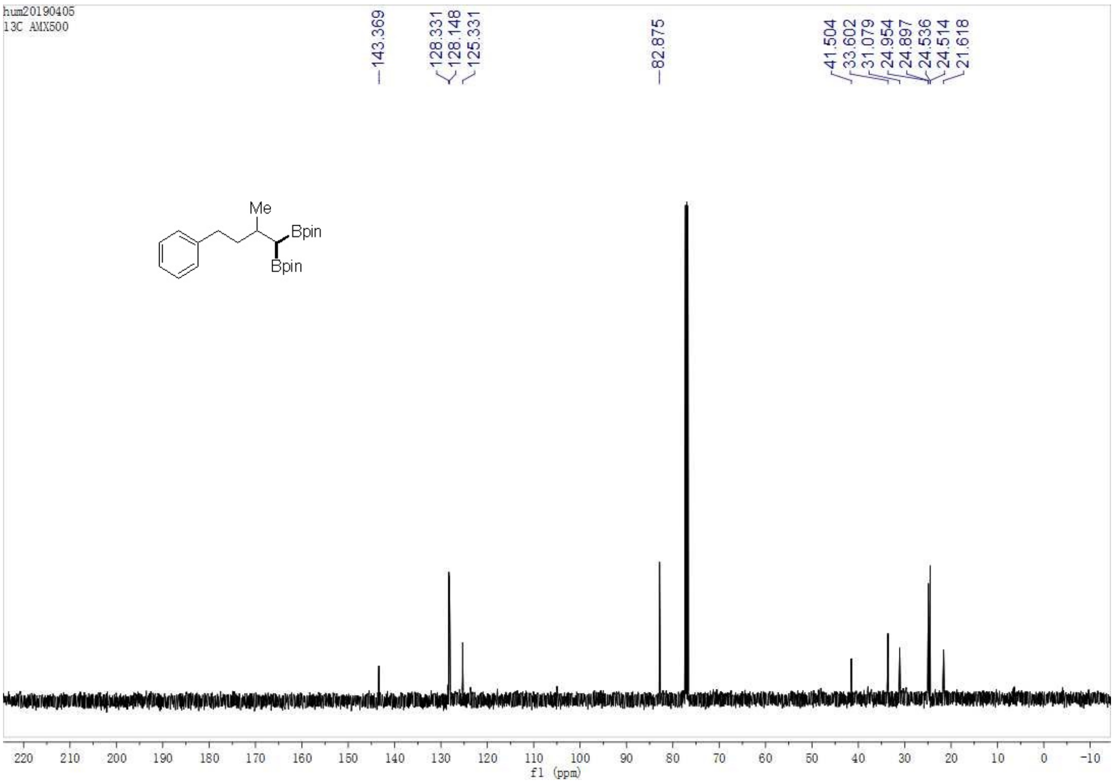

Supplementary Figure 34. $^{13}C\{^{1}H\}$ NMR spectrum of compound **2l**.

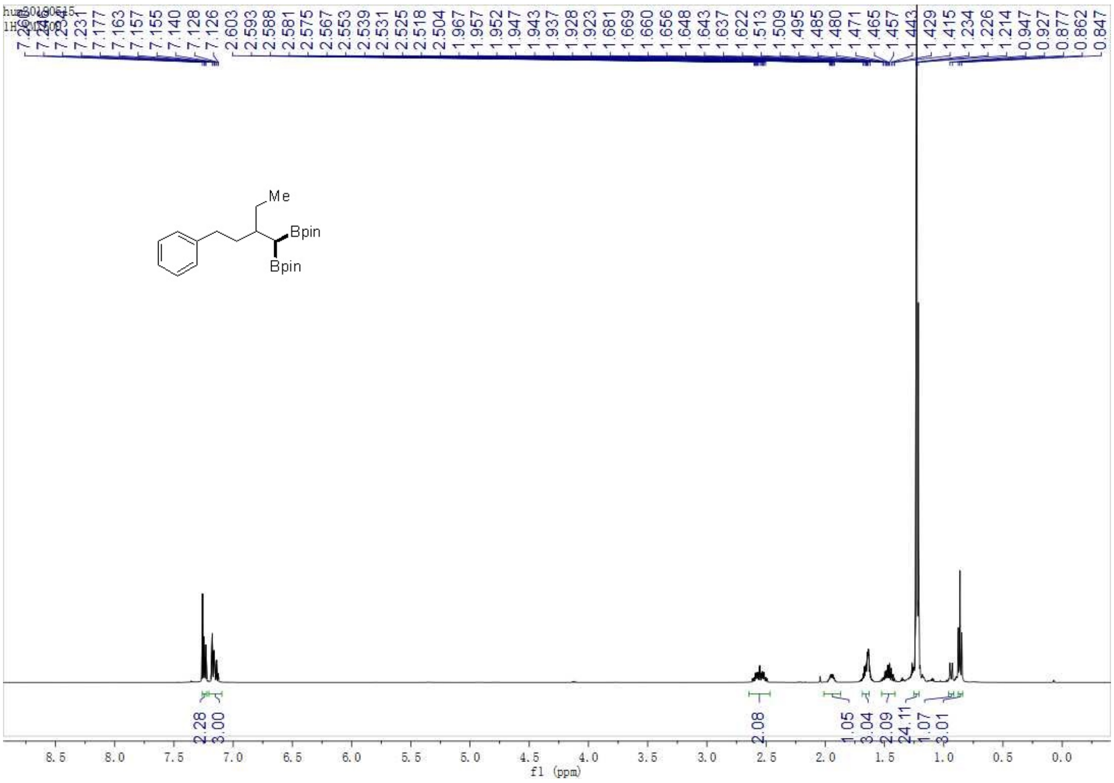

Supplementary Figure 35. ^{1}H NMR spectrum of compound **2m**.

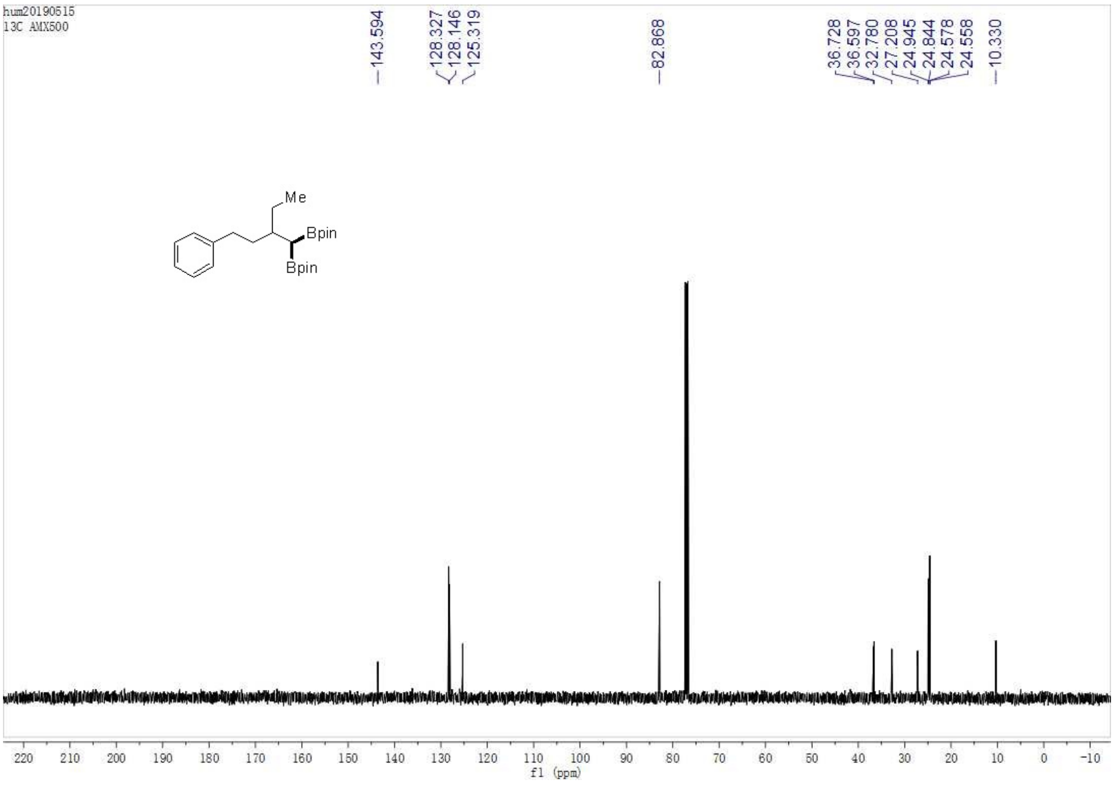

Supplementary Figure 36. $^{13}C\{^{1}H\}$ NMR spectrum of compound **2m**.

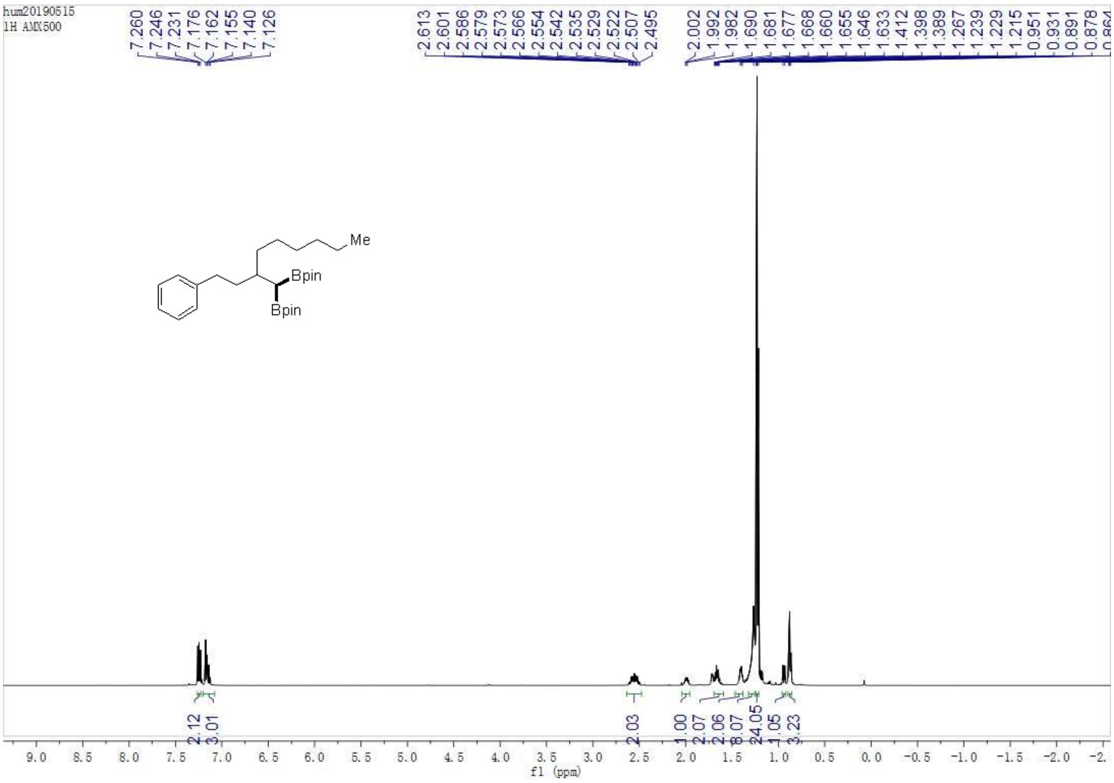

Supplementary Figure 37. ^{1}H NMR spectrum of compound **2n**.

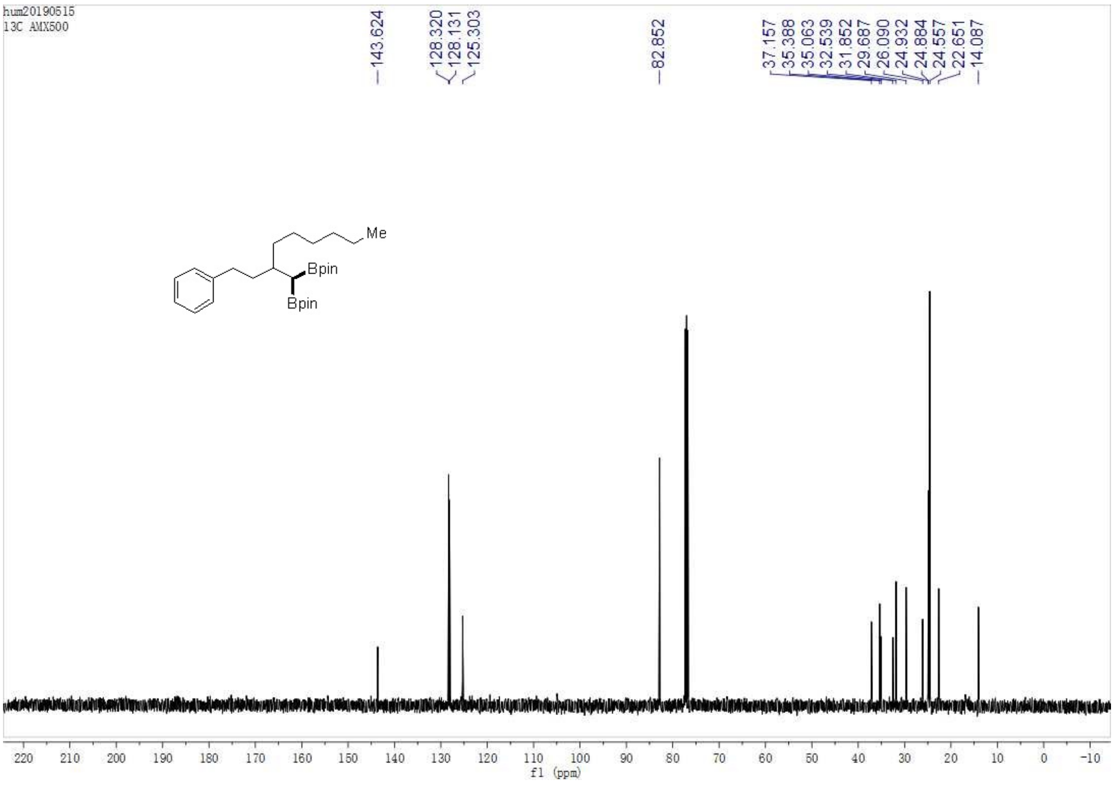

Supplementary Figure 38. $^{13}C\{^{1}H\}$ NMR spectrum of compound **2n**.

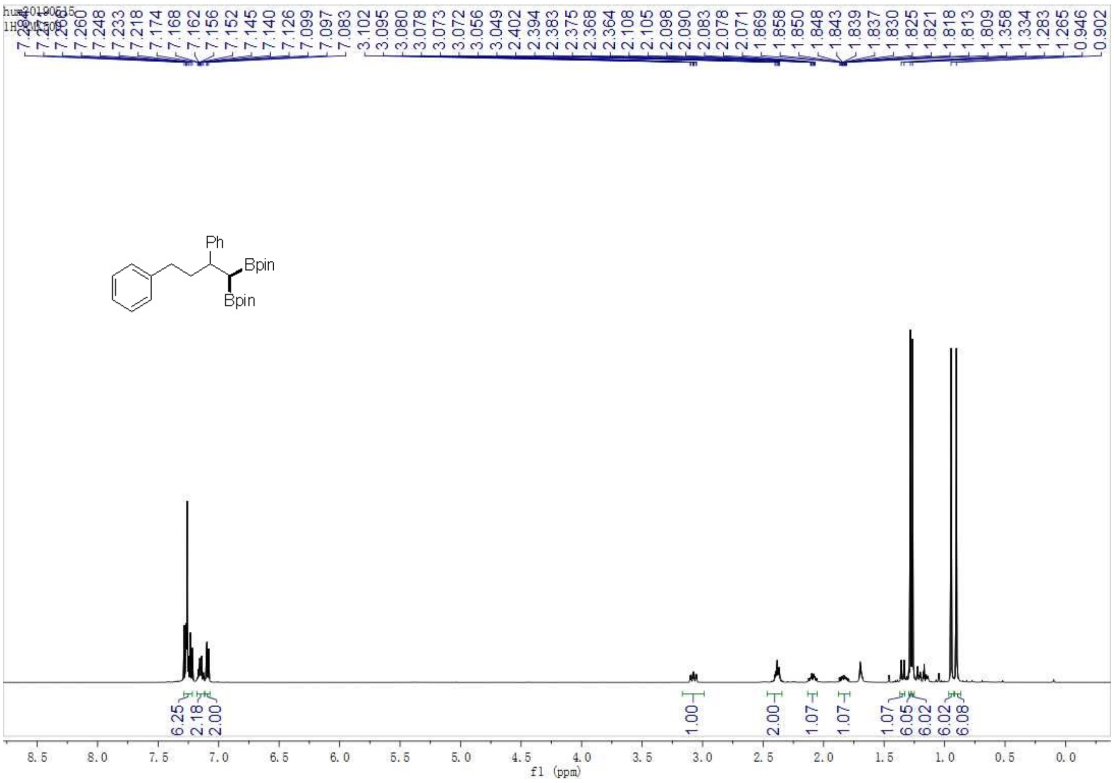

Supplementary Figure 39. ^{1}H NMR spectrum of compound **2o**.

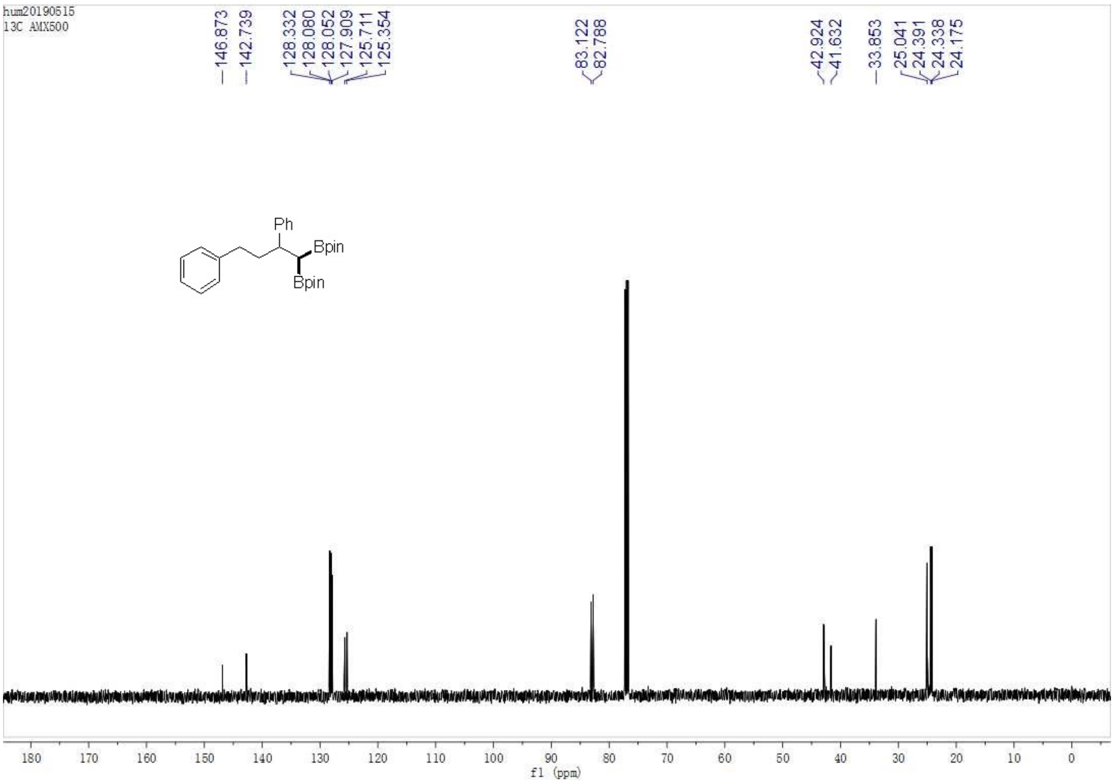

Supplementary Figure 40. ^{13}C{^{1}H} NMR spectrum of compound **2o**.

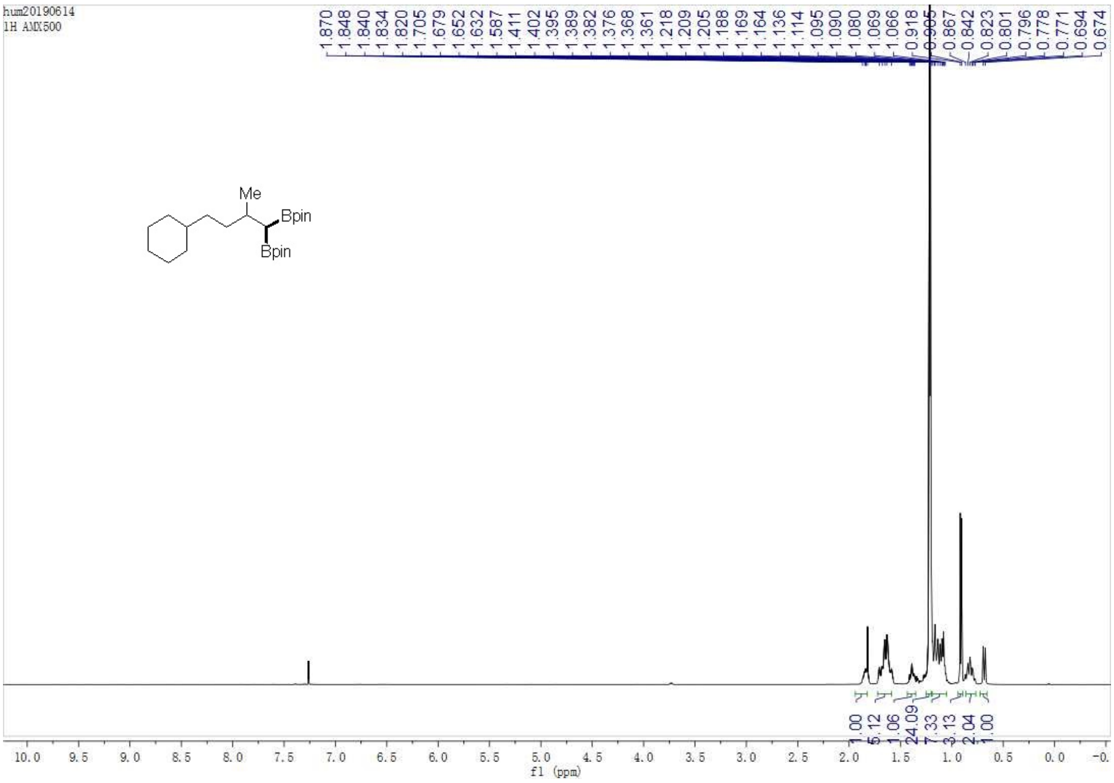

Supplementary Figure 41. ^{1}H NMR spectrum of compound **2p**.

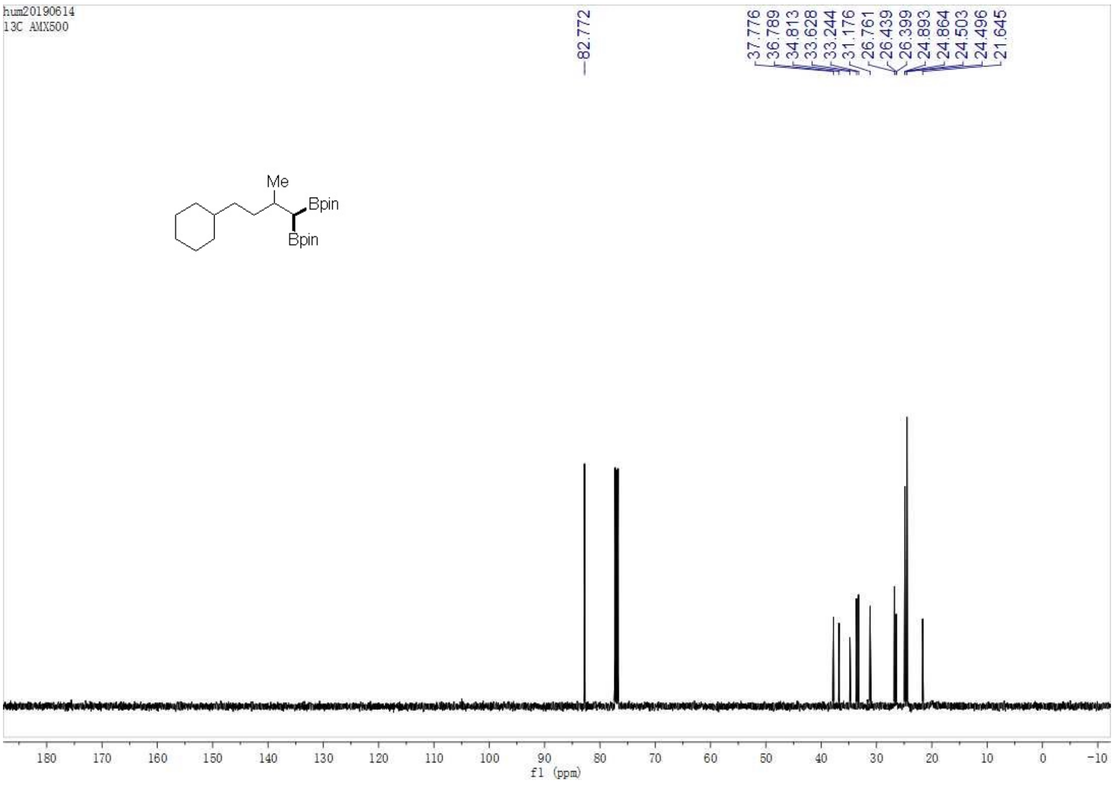

Supplementary Figure 42. $^{13}C\{^{1}H\}$ NMR spectrum of compound **2p**.

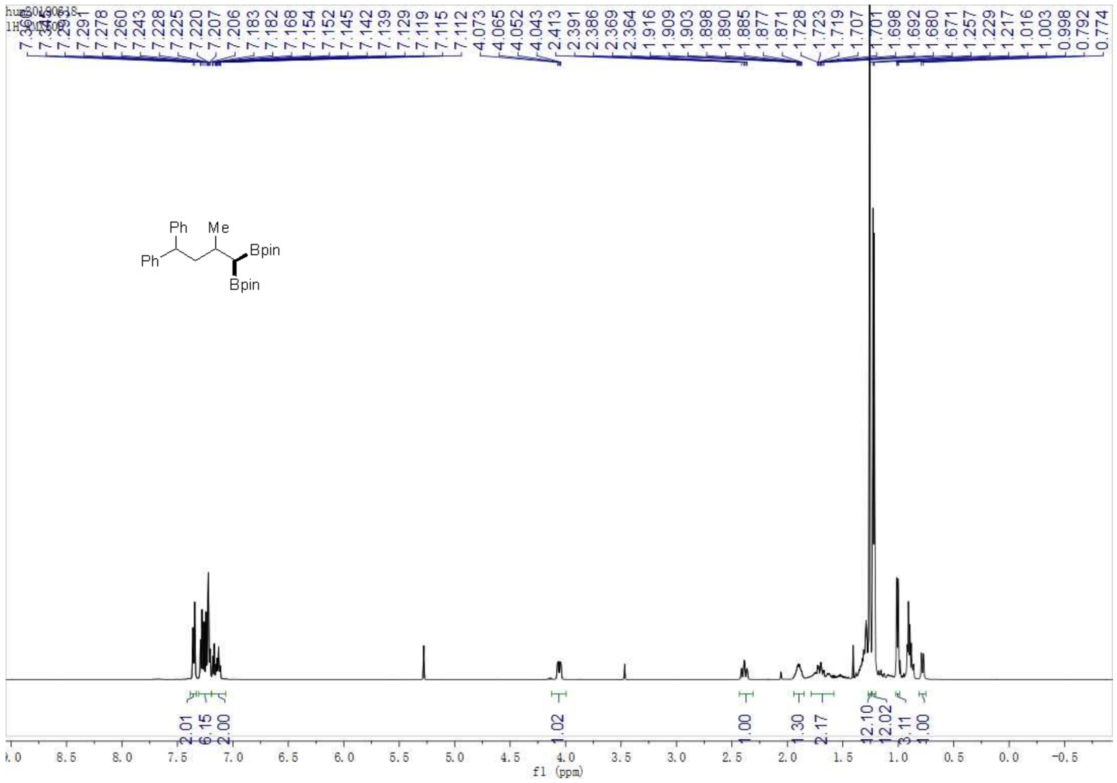

Supplementary Figure 43. ^{1}H NMR spectrum of compound **2q**.

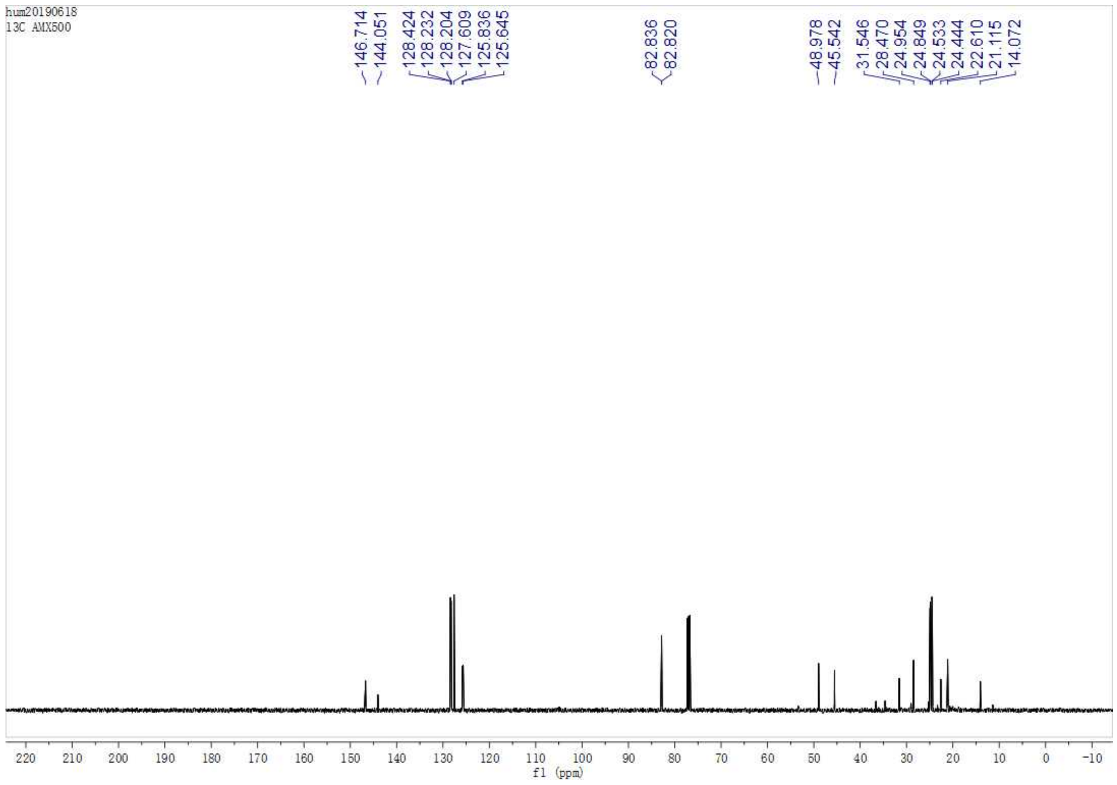

Supplementary Figure 44. $^{13}C\{^{1}H\}$ NMR spectrum of compound **2q**.

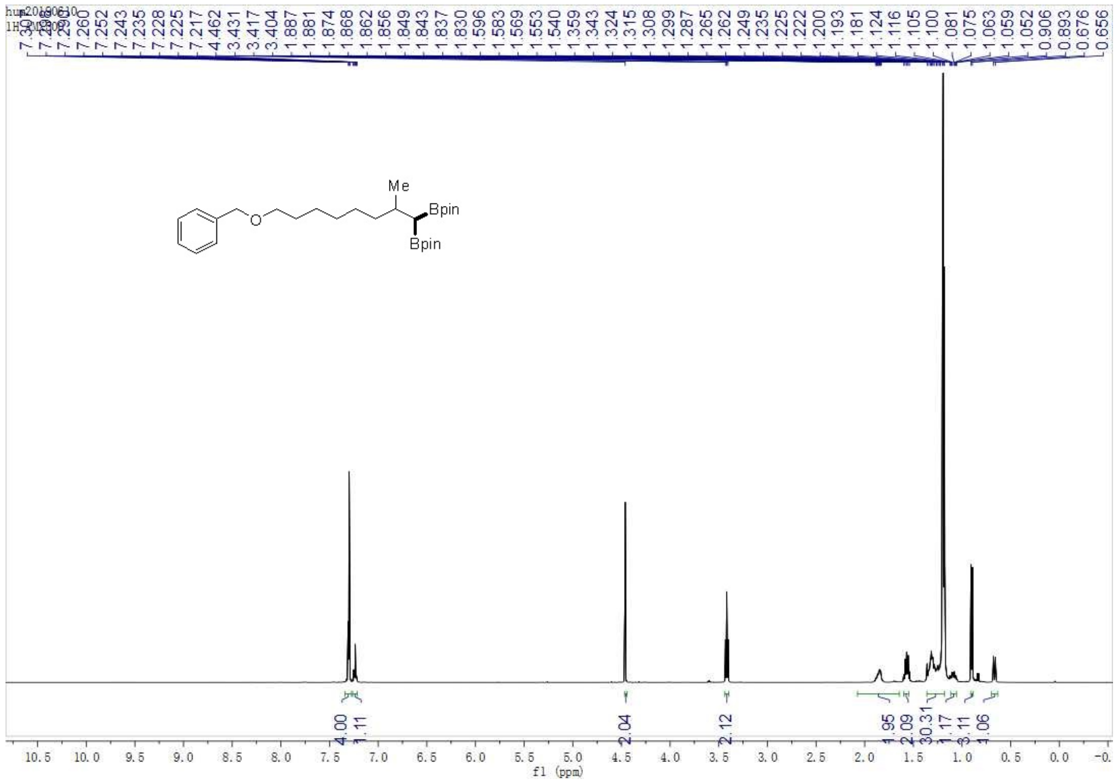

Supplementary Figure 45. ^{1}H NMR spectrum of compound **2r**.

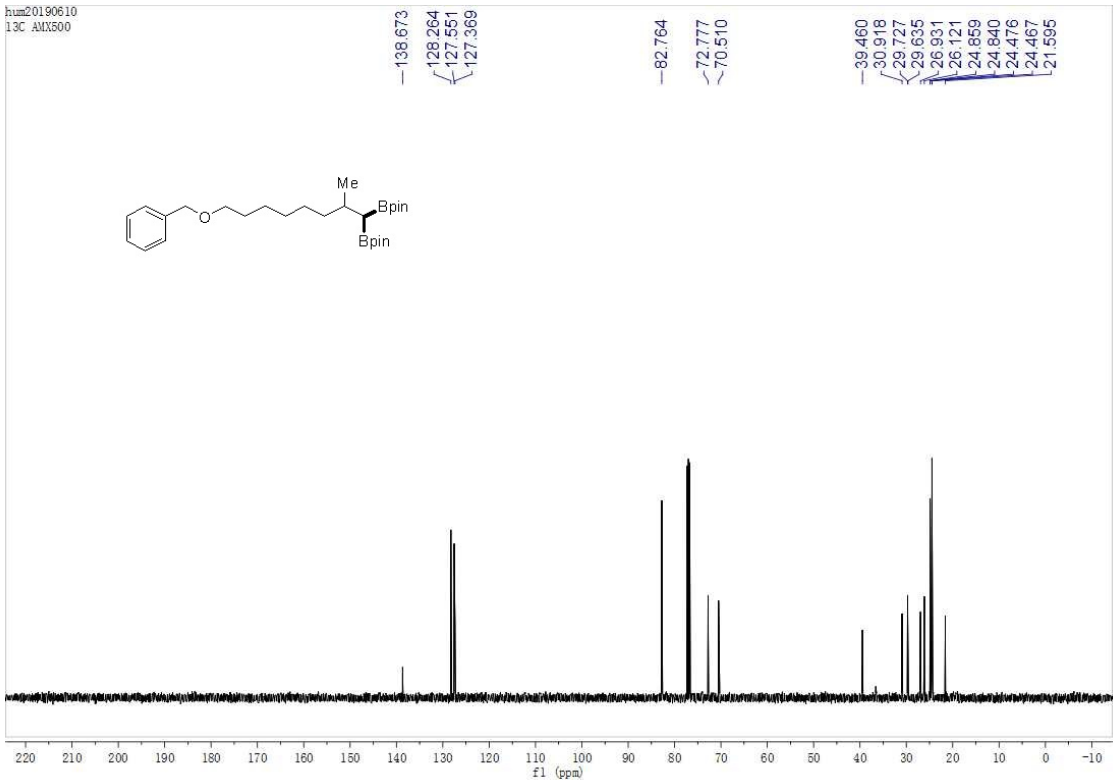

Supplementary Figure 46. $^{13}C\{^{1}H\}$ NMR spectrum of compound **2r**.

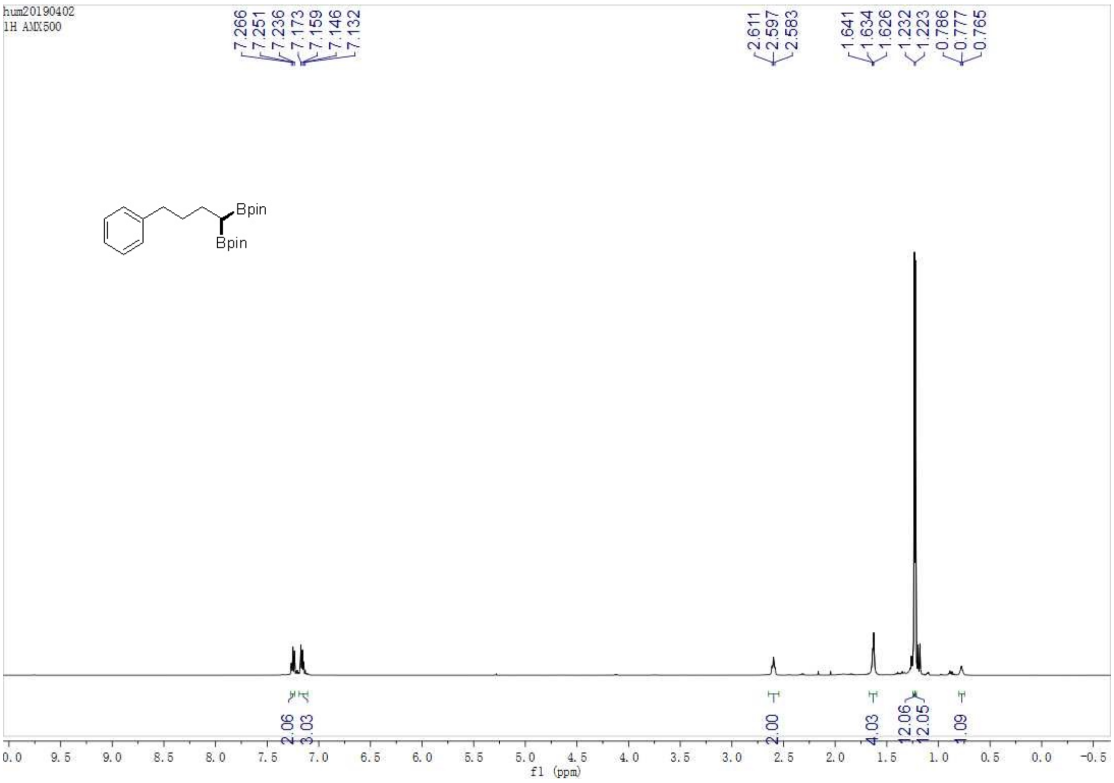

Supplementary Figure 47. ^{1}H NMR spectrum of compound **2s**.

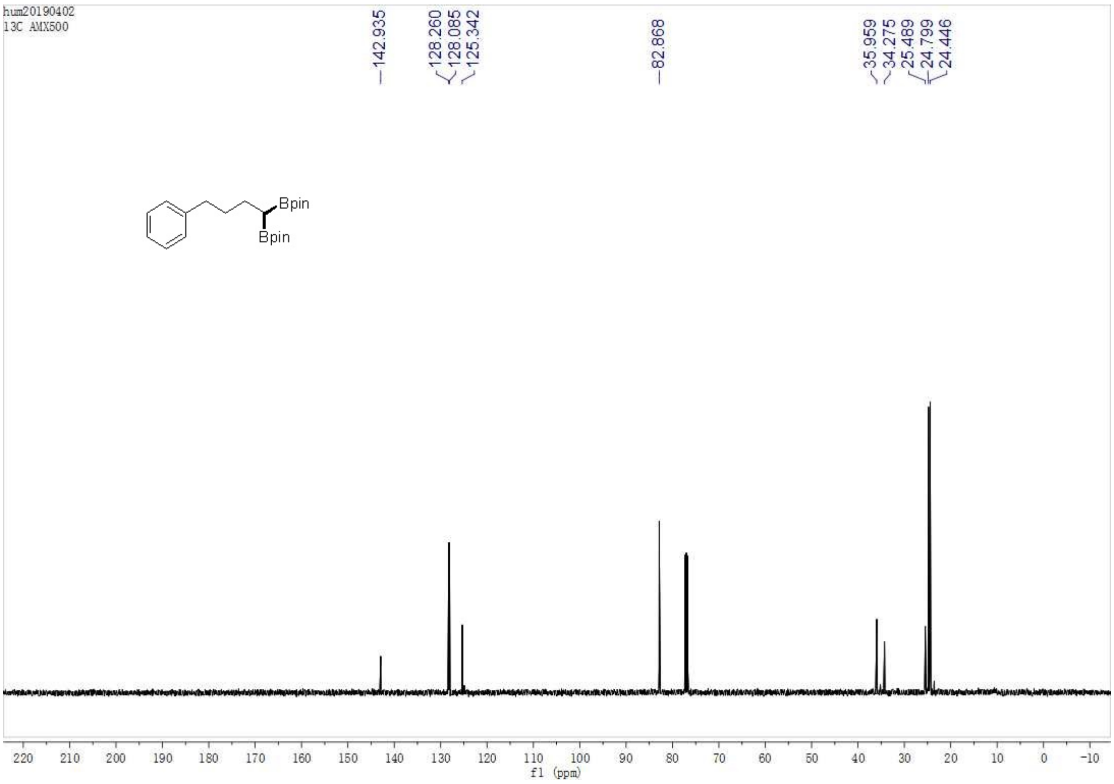

Supplementary Figure 48. $^{13}C\{^{1}H\}$ NMR spectrum of compound **2s**.

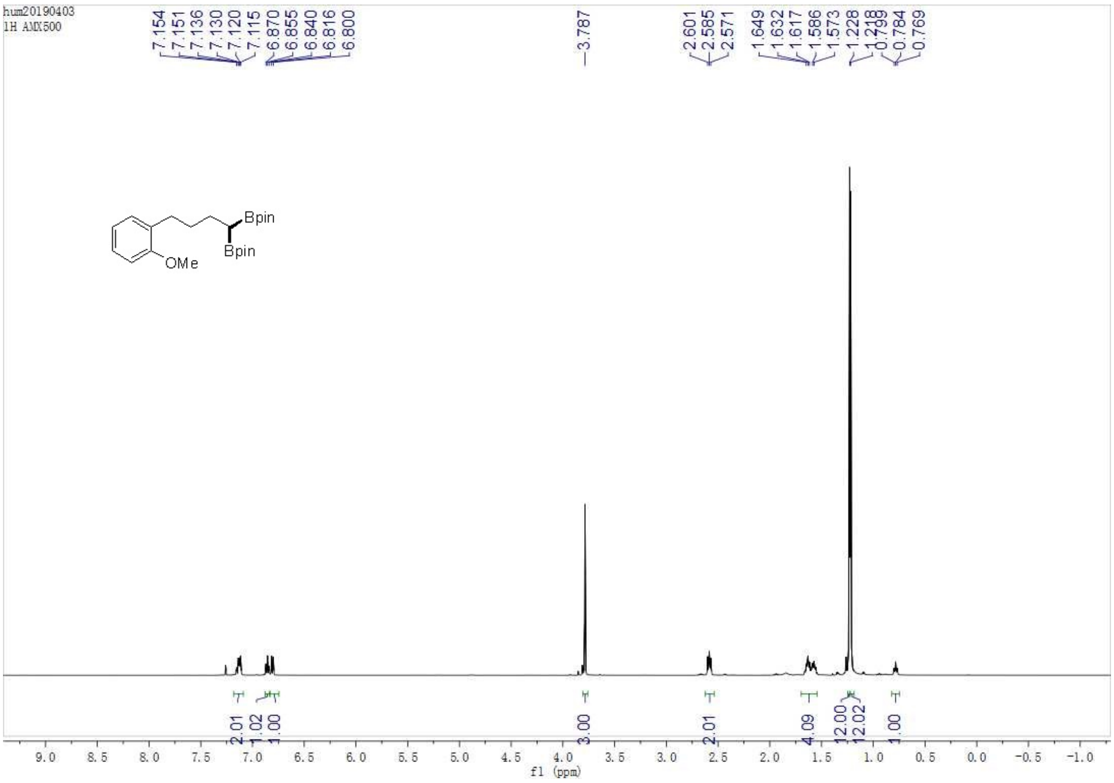

Supplementary Figure 49. ^{1}H NMR spectrum of compound **2t**.

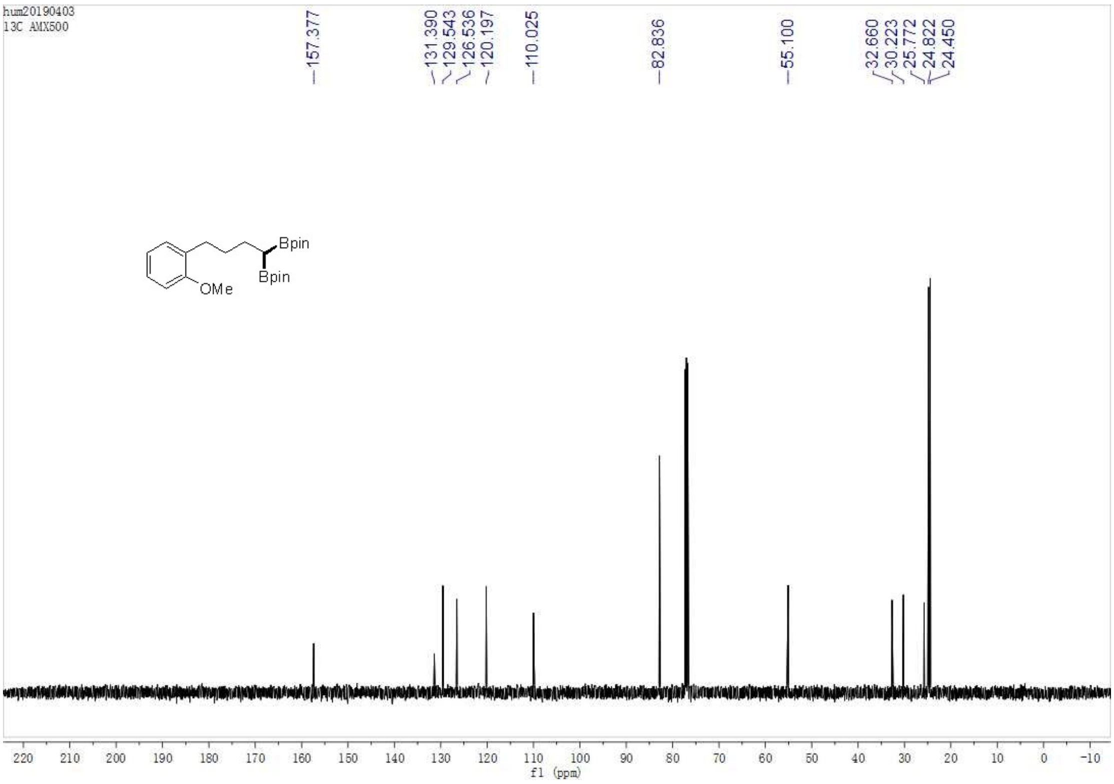

Supplementary Figure 50. $^{13}C\{^{1}H\}$ NMR spectrum of compound **2t**.

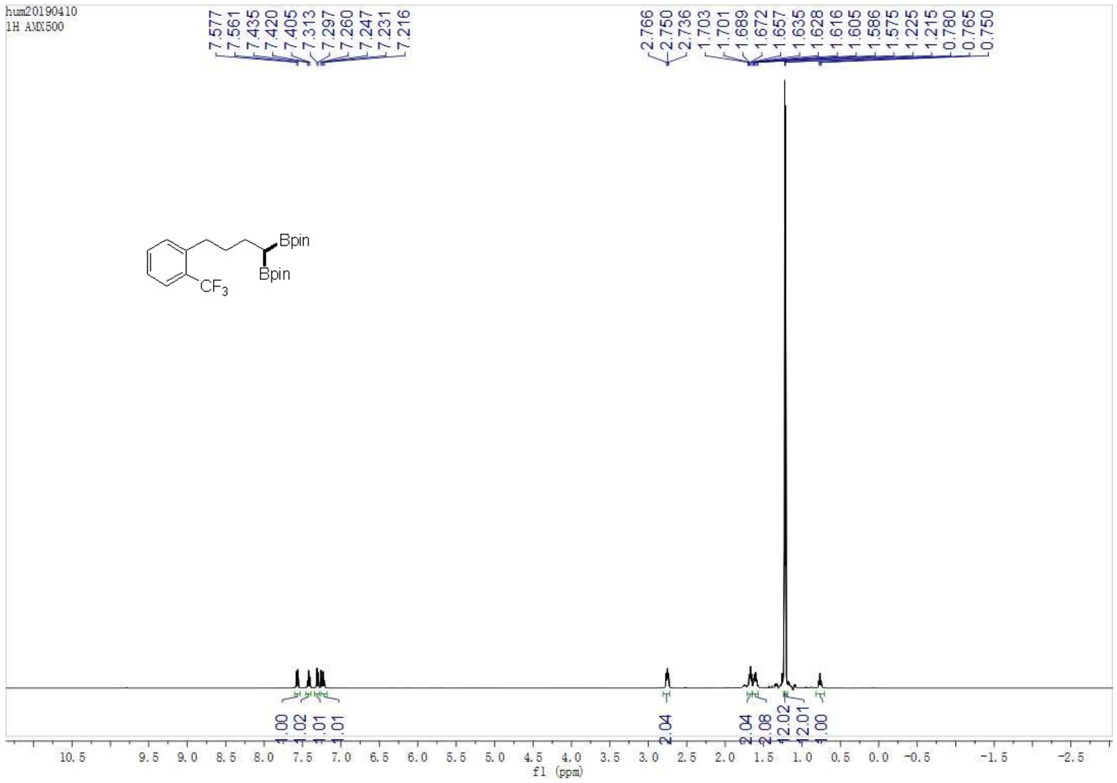

Supplementary Figure 51. ^{1}H NMR spectrum of compound **2u**.

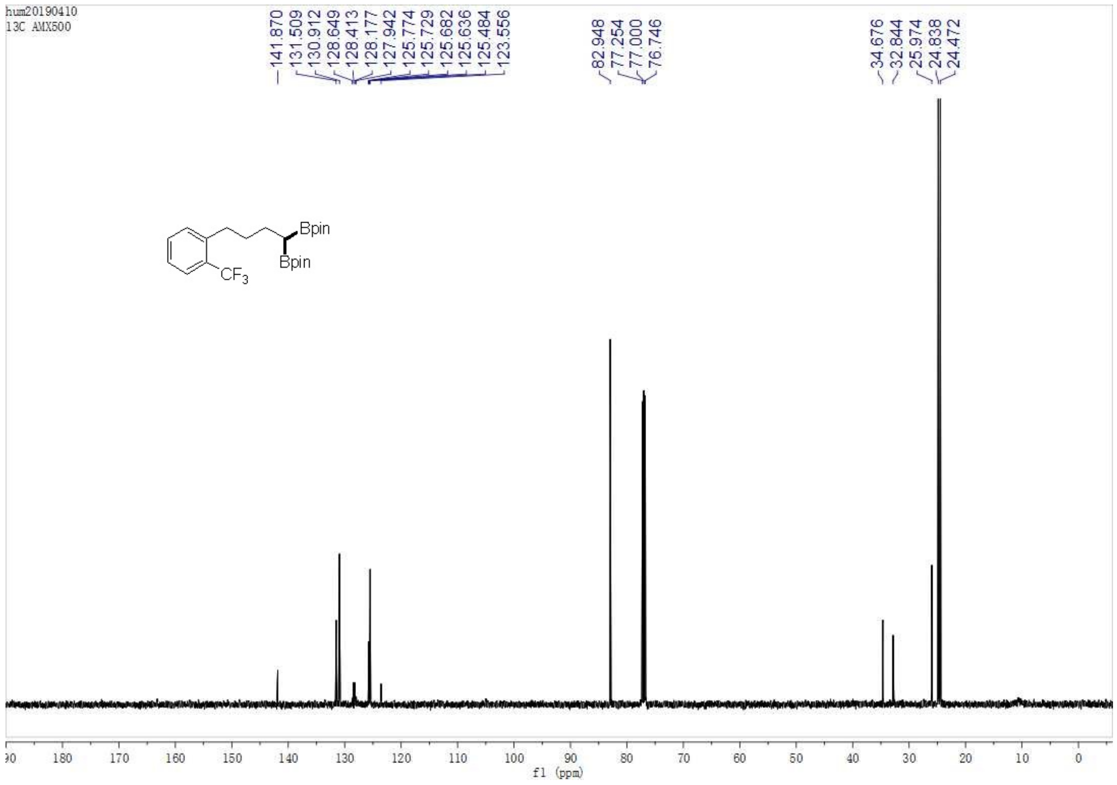

Supplementary Figure 52. $^{13}C\{^{1}H\}$ NMR spectrum of compound **2u**.

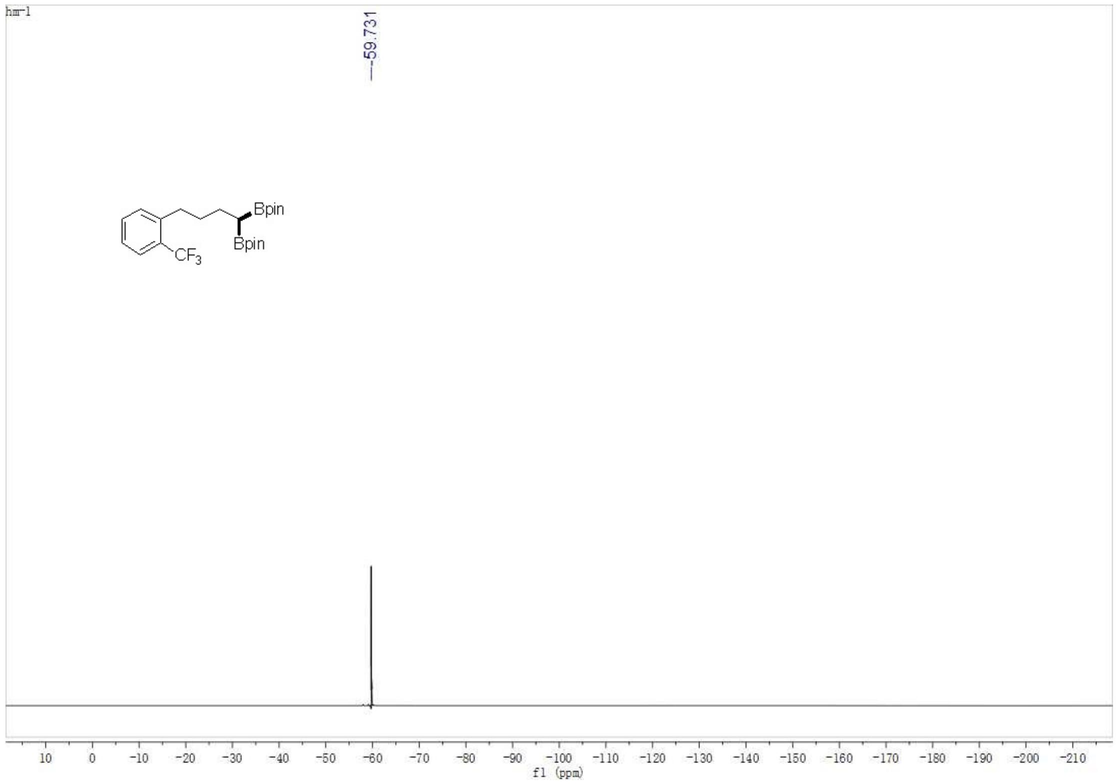

Supplementary Figure 53. ^{19}F NMR spectrum of compound **2u**.

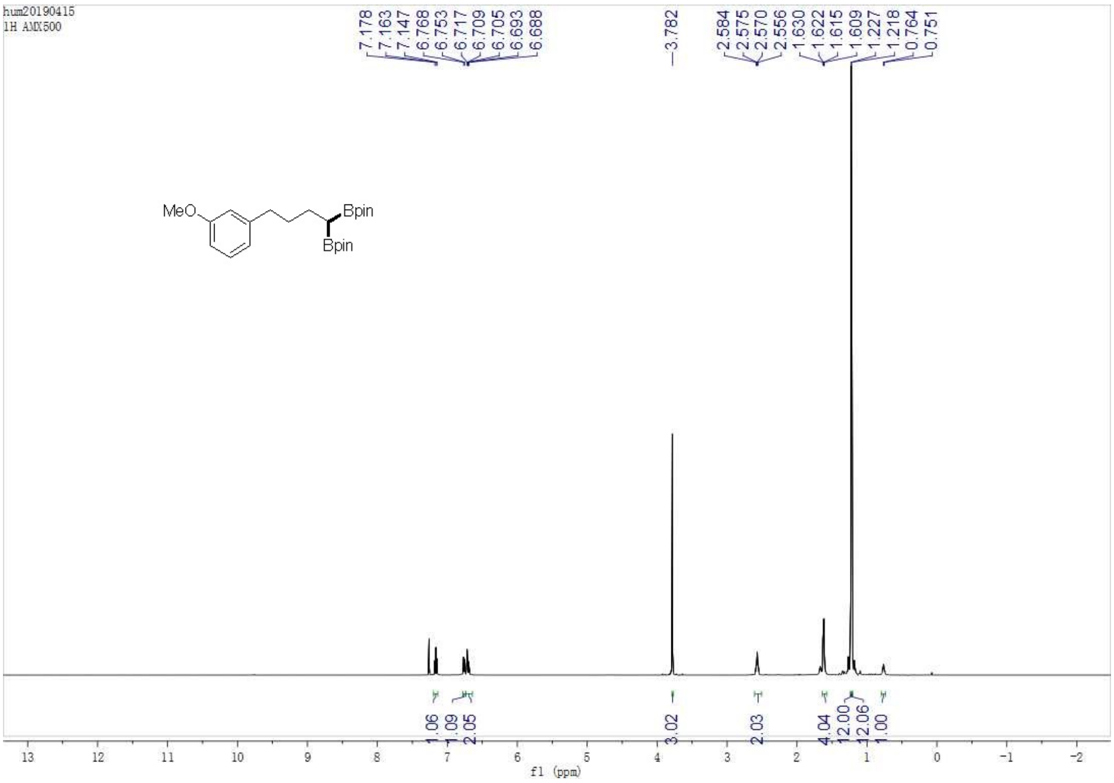

Supplementary Figure 54. ^{1}H NMR spectrum of compound **2v**.

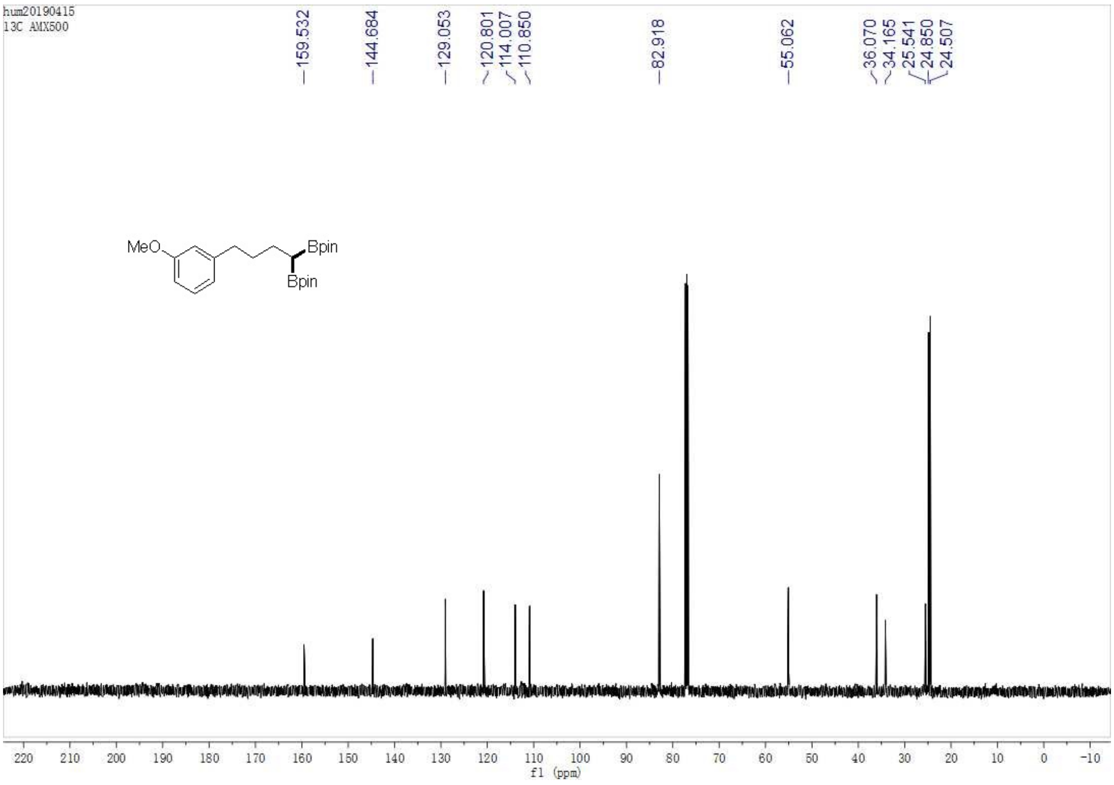

Supplementary Figure 55. ^{13}C{^{1}H} NMR spectrum of compound **2v**.

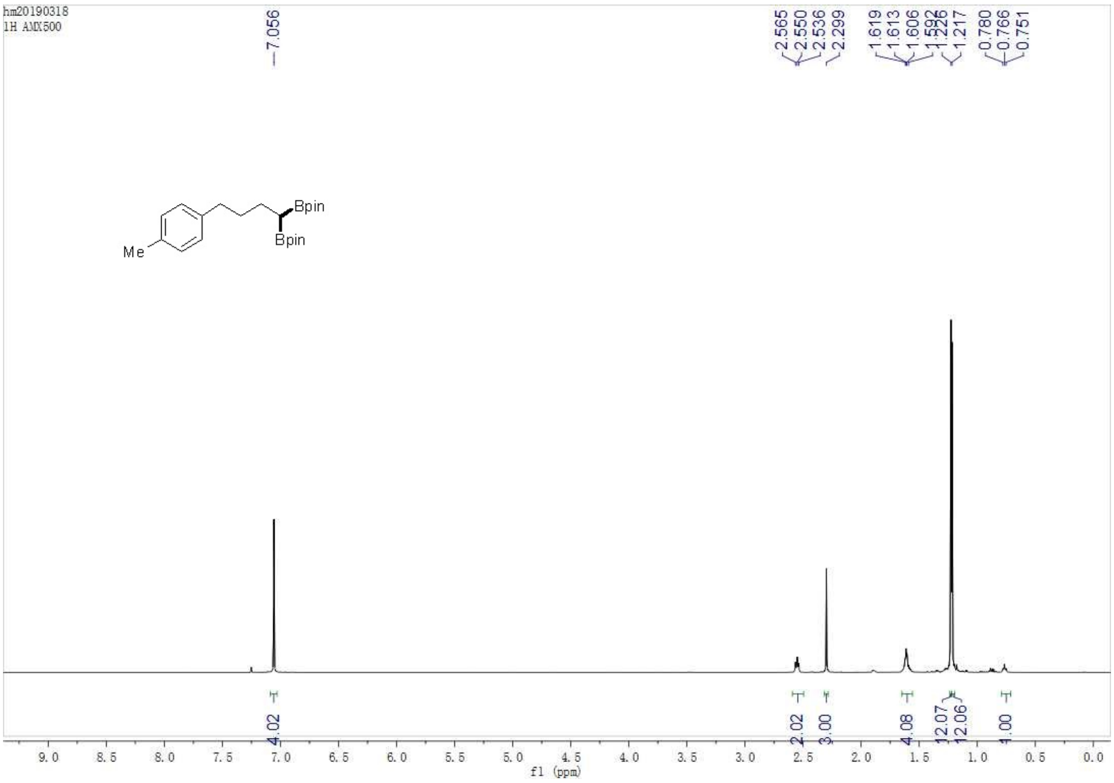

Supplementary Figure 56. ^{1}H NMR spectrum of compound **2w**.

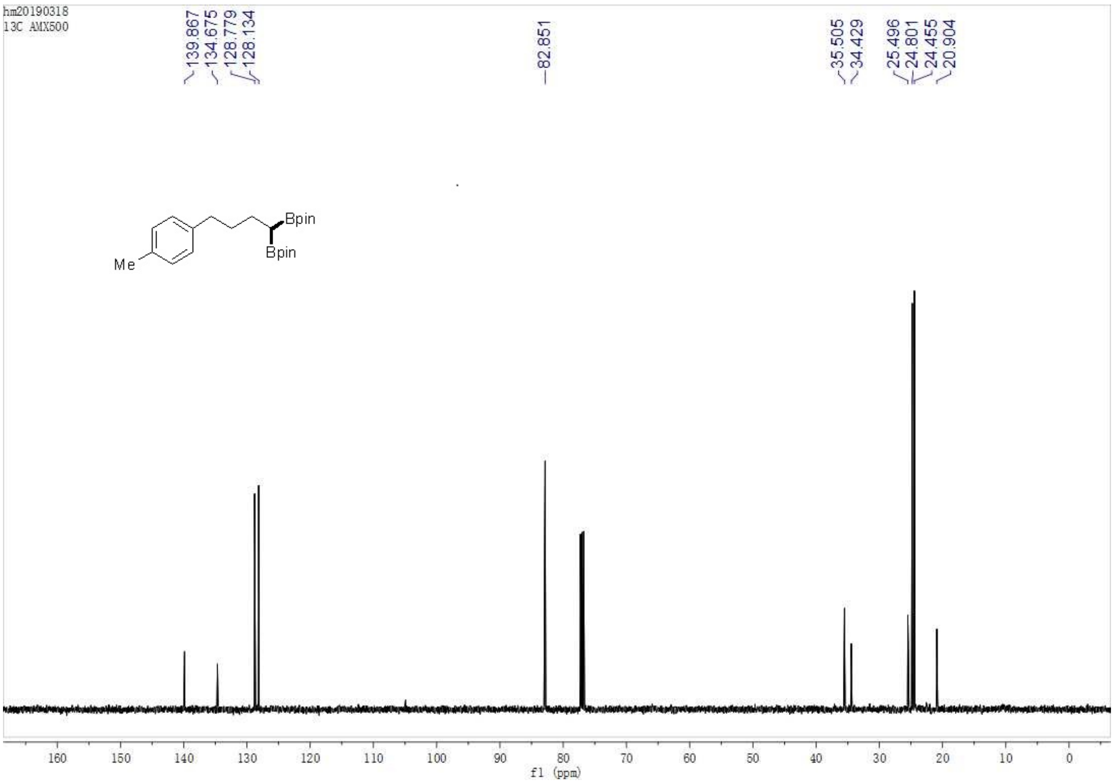

Supplementary Figure 57. ^{13}C{^{1}H} NMR spectrum of compound **2w**.

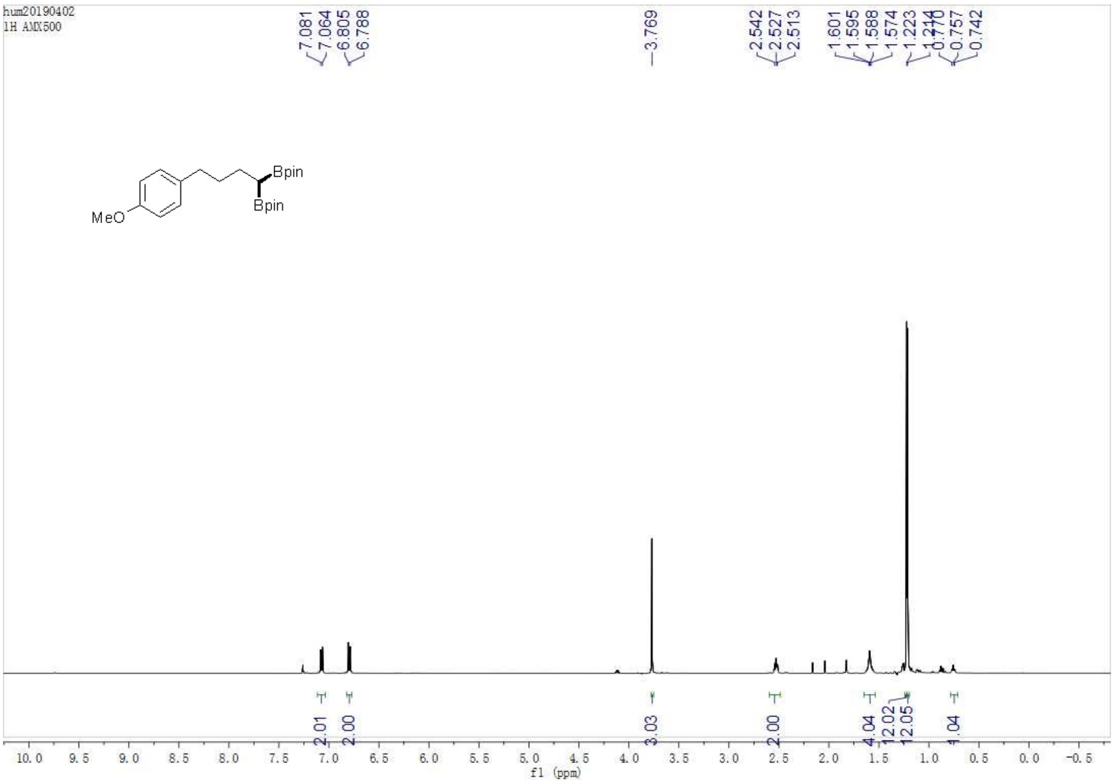

Supplementary Figure 58. ^{1}H NMR spectrum of compound **2x**.

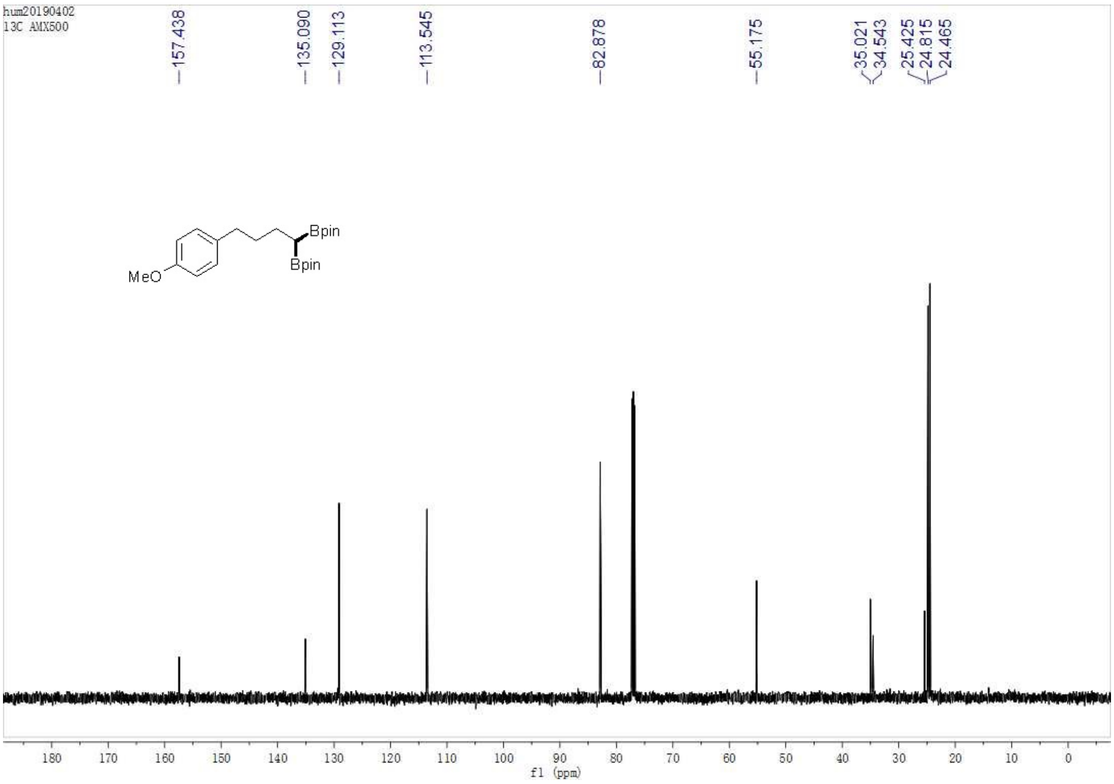

Supplementary Figure 59. $^{13}C\{^{1}H\}$ NMR spectrum of compound **2x**.

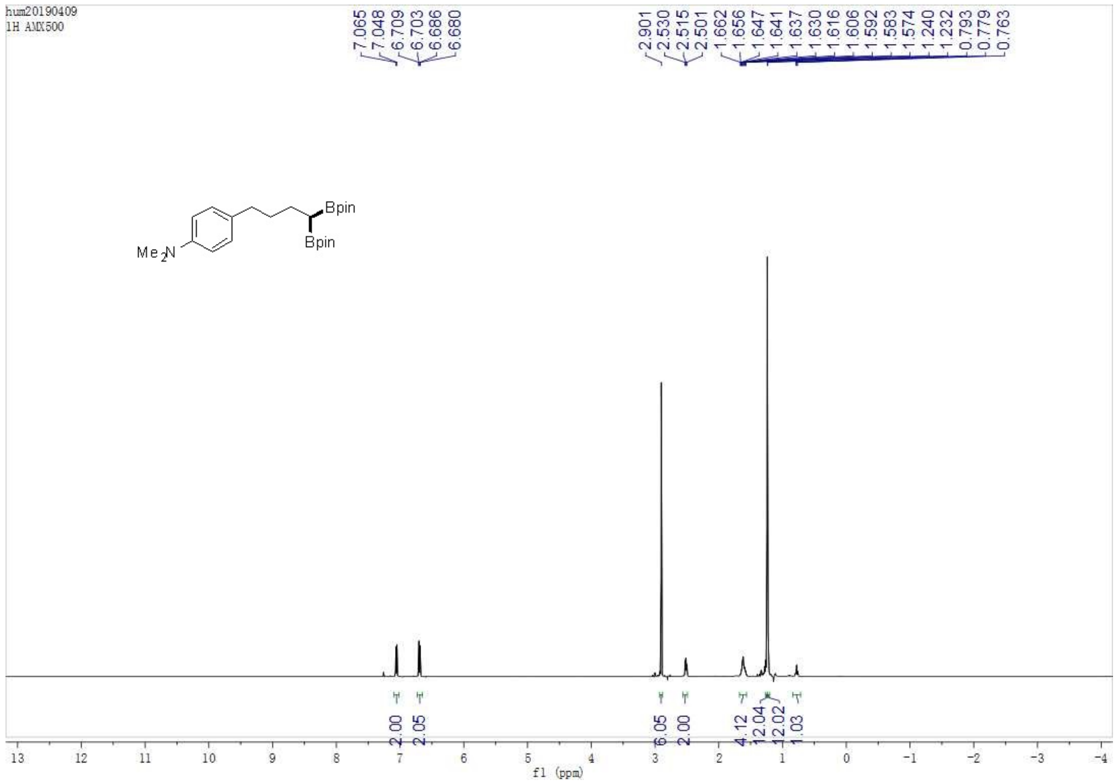

Supplementary Figure 60. ^{1}H NMR spectrum of compound **2y**.

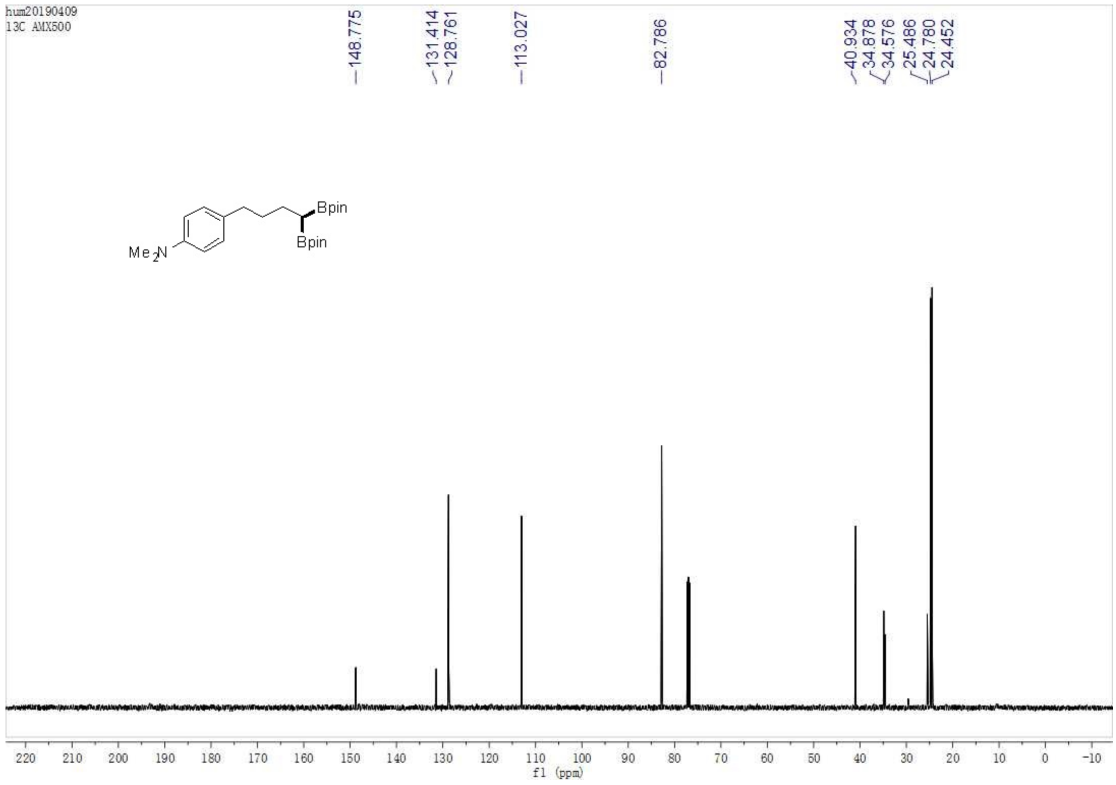

Supplementary Figure 61. $^{13}C\{^{1}H\}$ NMR spectrum of compound **2y**.

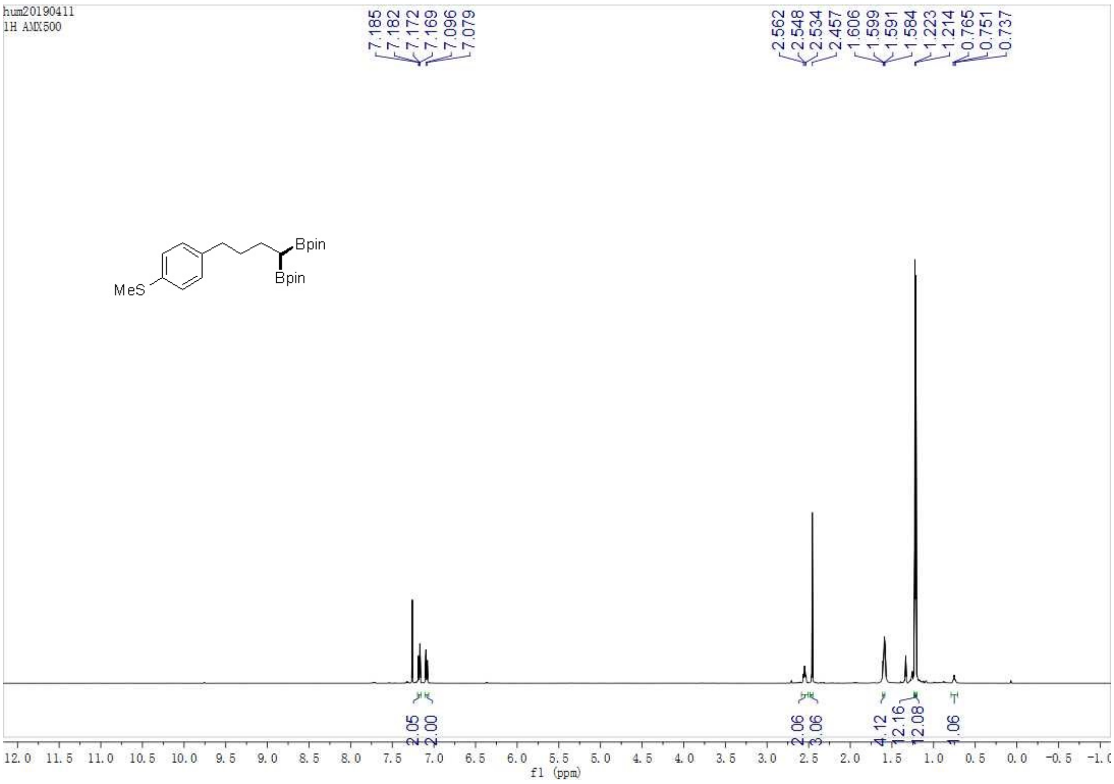

Supplementary Figure 62. ^{1}H NMR spectrum of compound **2z**.

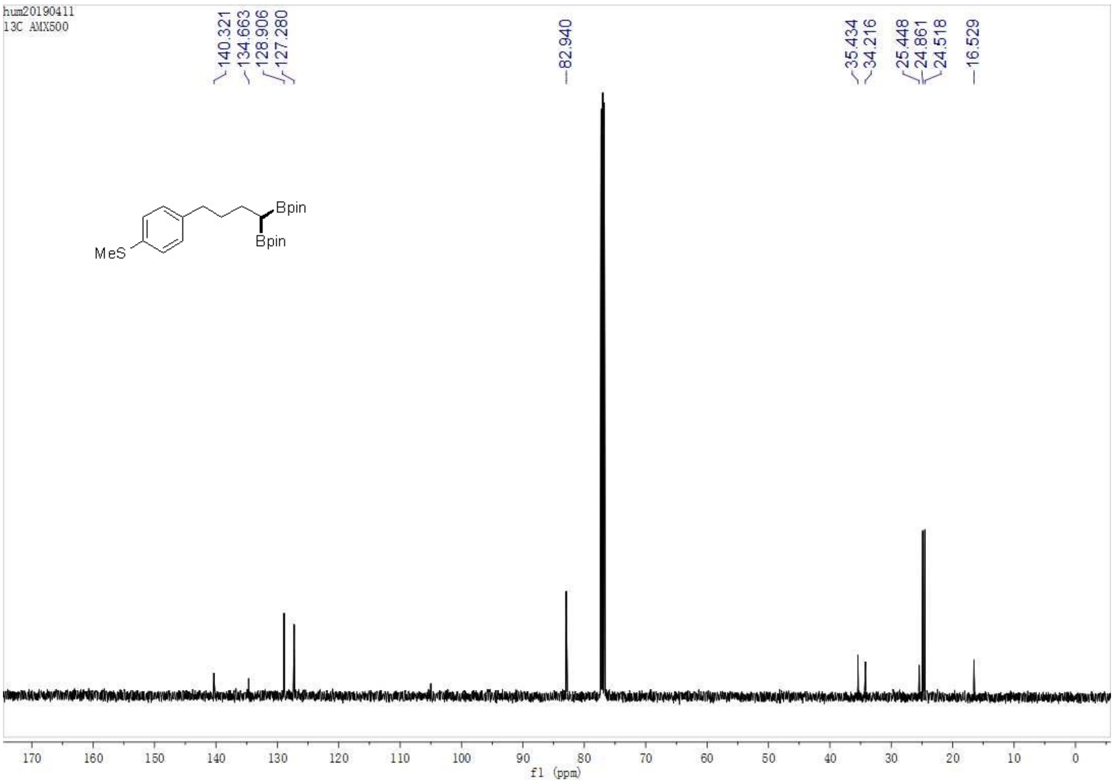

Supplementary Figure 63. ^{13}C{^{1}H} NMR spectrum of compound **2z**.

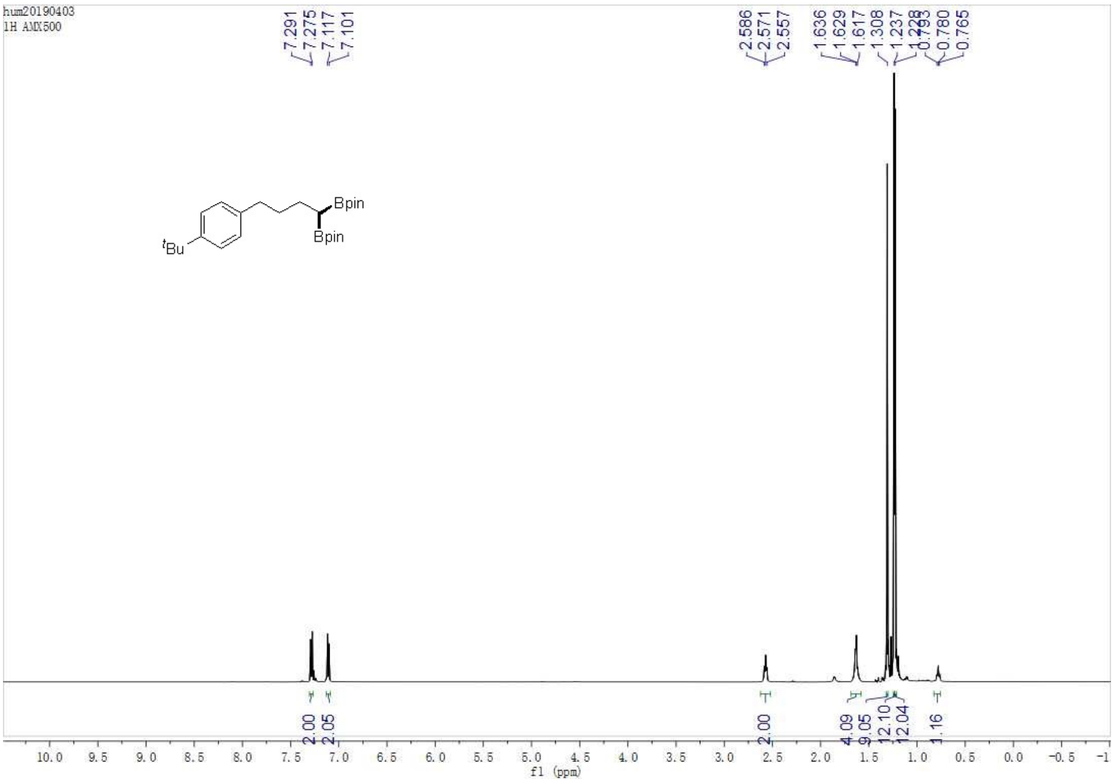

Supplementary Figure 64. ^{1}H NMR spectrum of compound **2aa**.

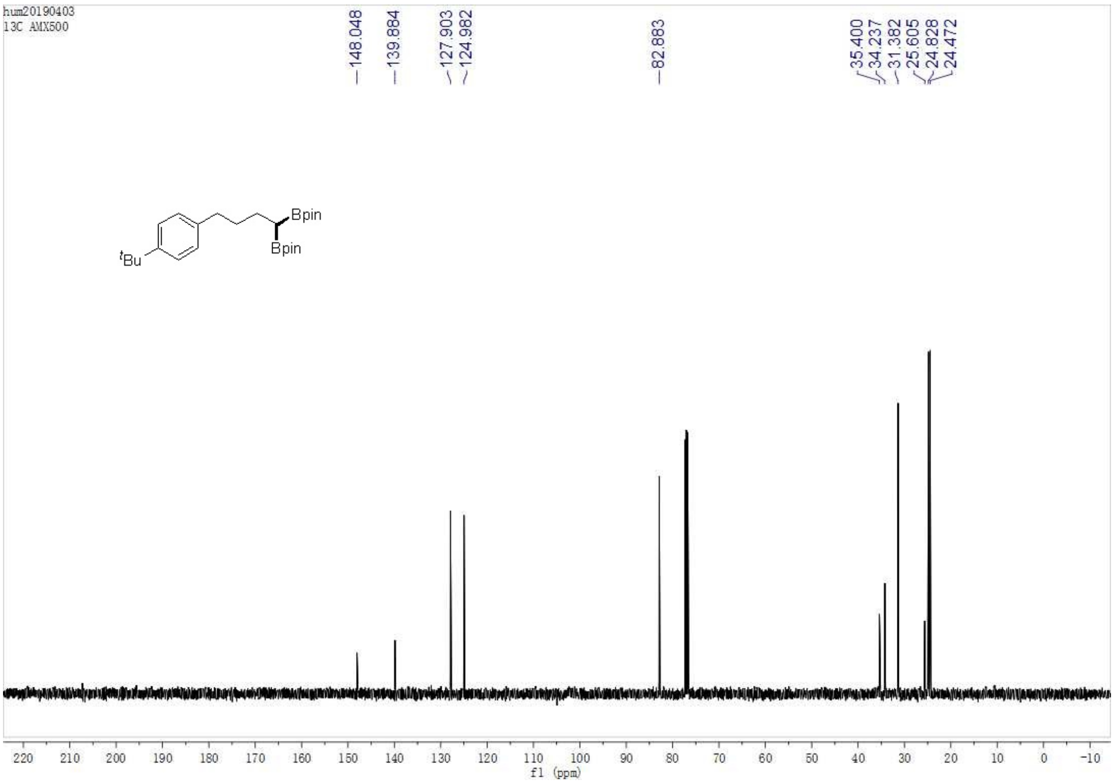

Supplementary Figure 65. ^{13}C{^{1}H} NMR spectrum of compound **2aa**.

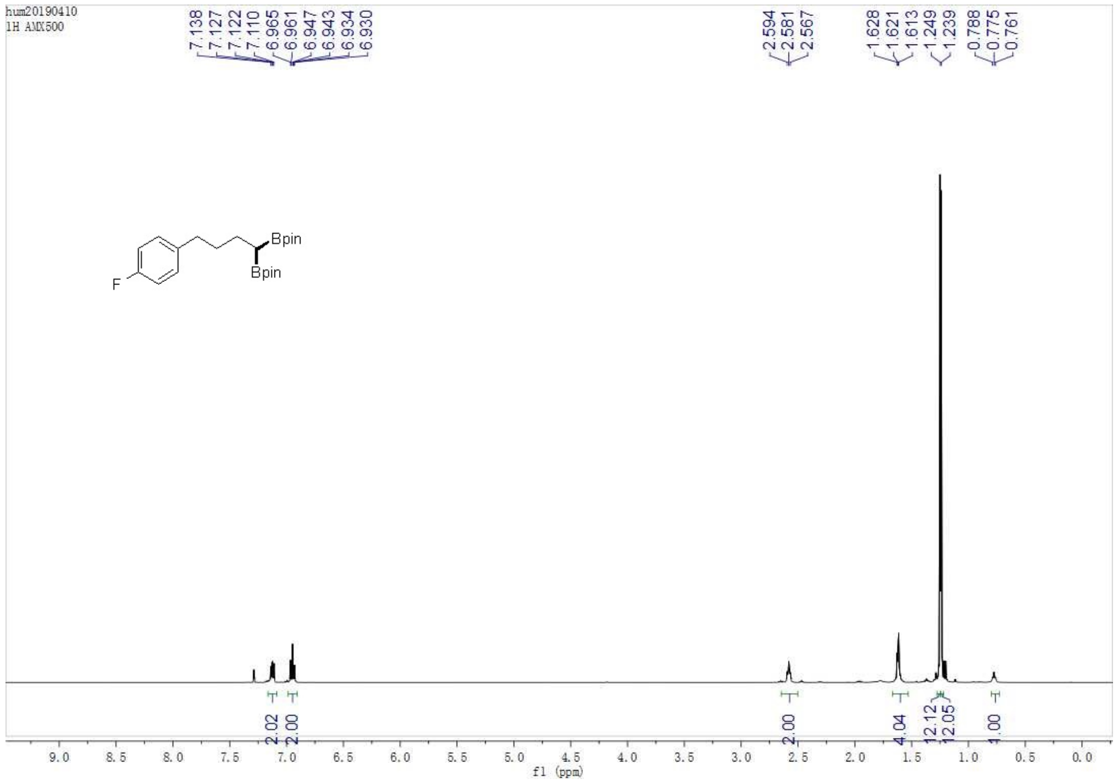

Supplementary Figure 66. ^{1}H NMR spectrum of compound **2ab**.

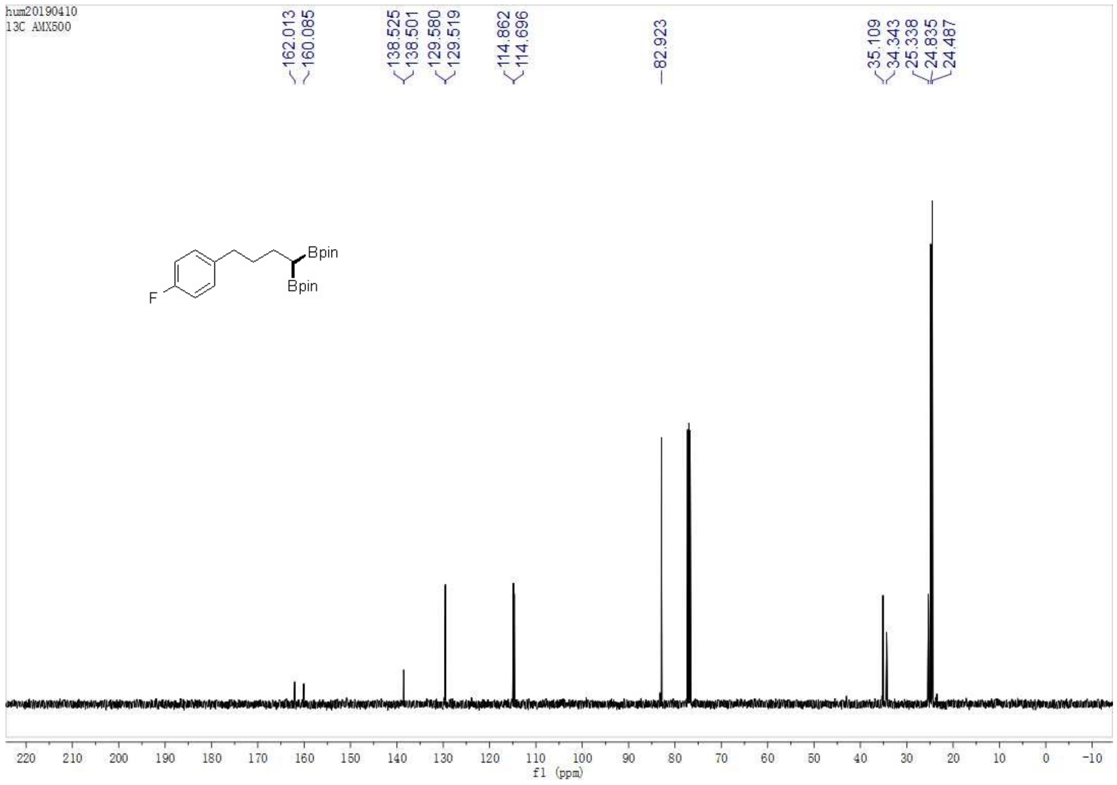

Supplementary Figure 67. ^{13}C{^{1}H} NMR spectrum of compound **2ab**.

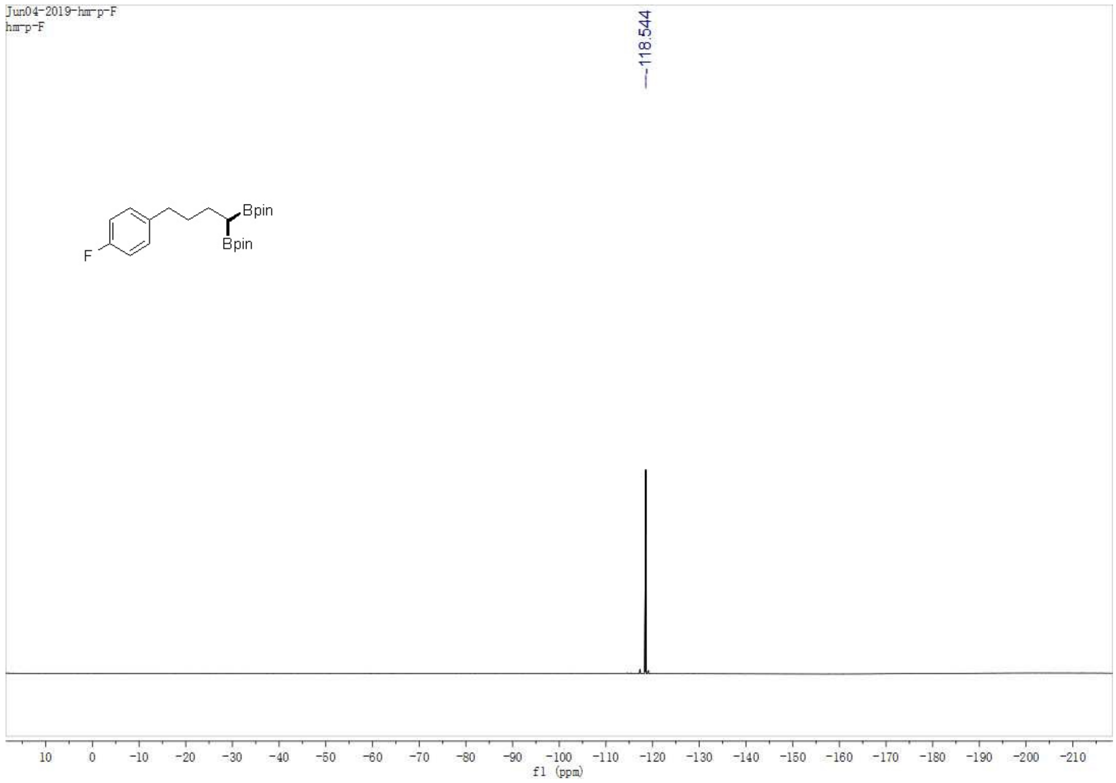

Supplementary Figure 68. ^{19}F NMR spectrum of compound **2ab**.

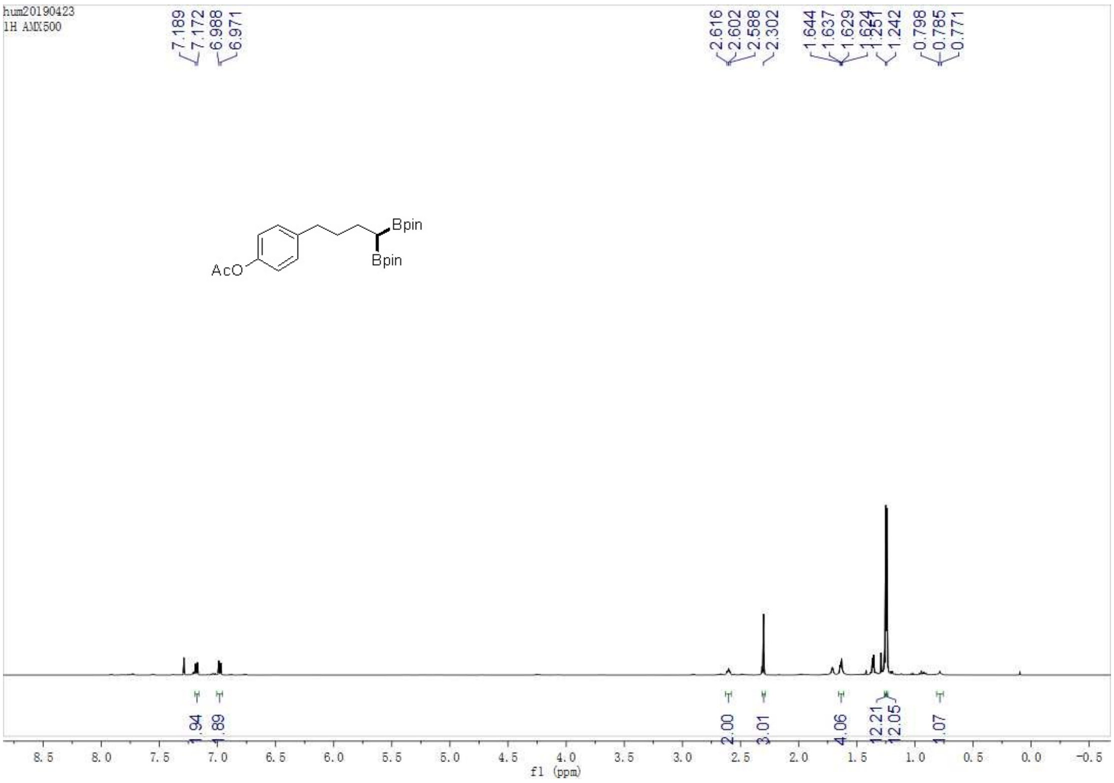

Supplementary Figure 69. ^{1}H NMR spectrum of compound **2ac**.

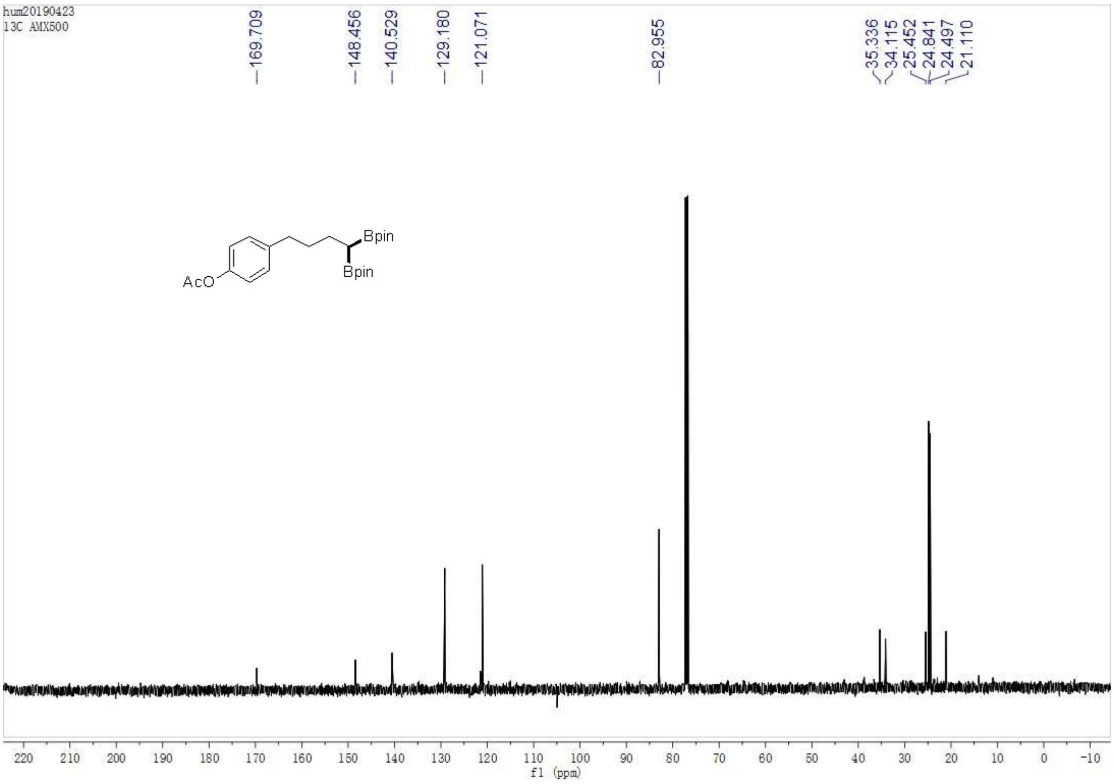

Supplementary Figure 70. ^{13}C{^{1}H} NMR spectrum of compound **2ad**.

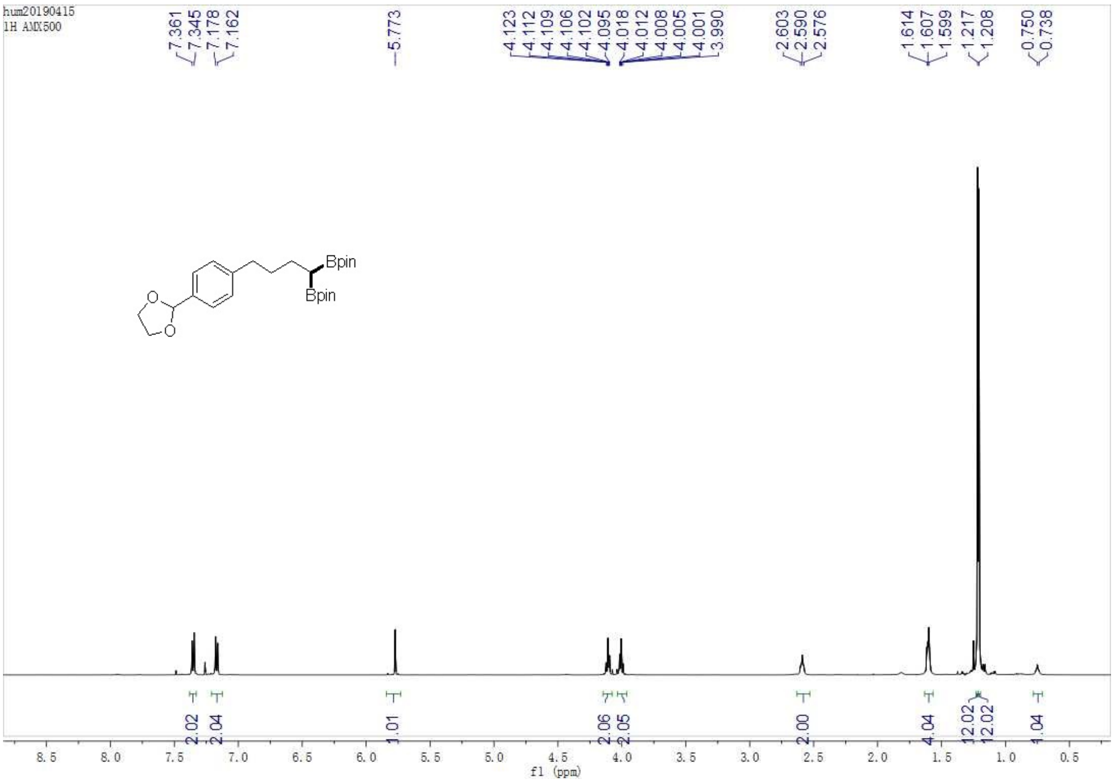

Supplementary Figure 71. ^{1}H NMR spectrum of compound **2ad**.

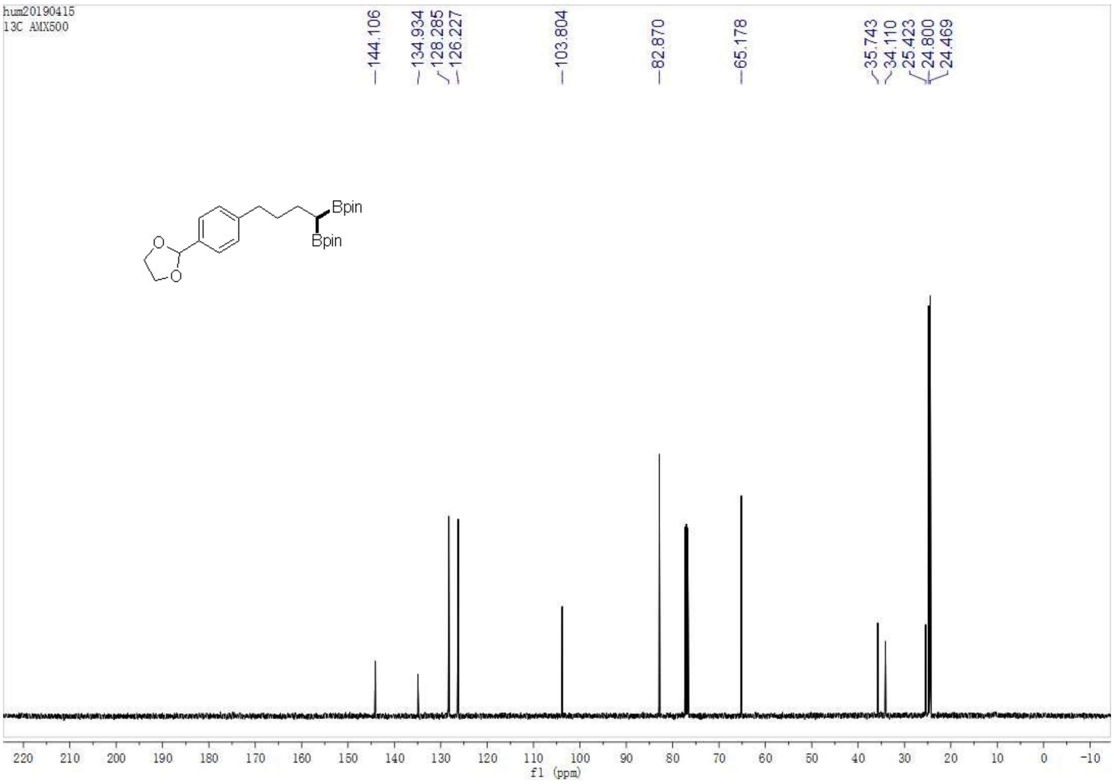

Supplementary Figure 72. ^{13}C{^{1}H} NMR spectrum of compound **2ad**.

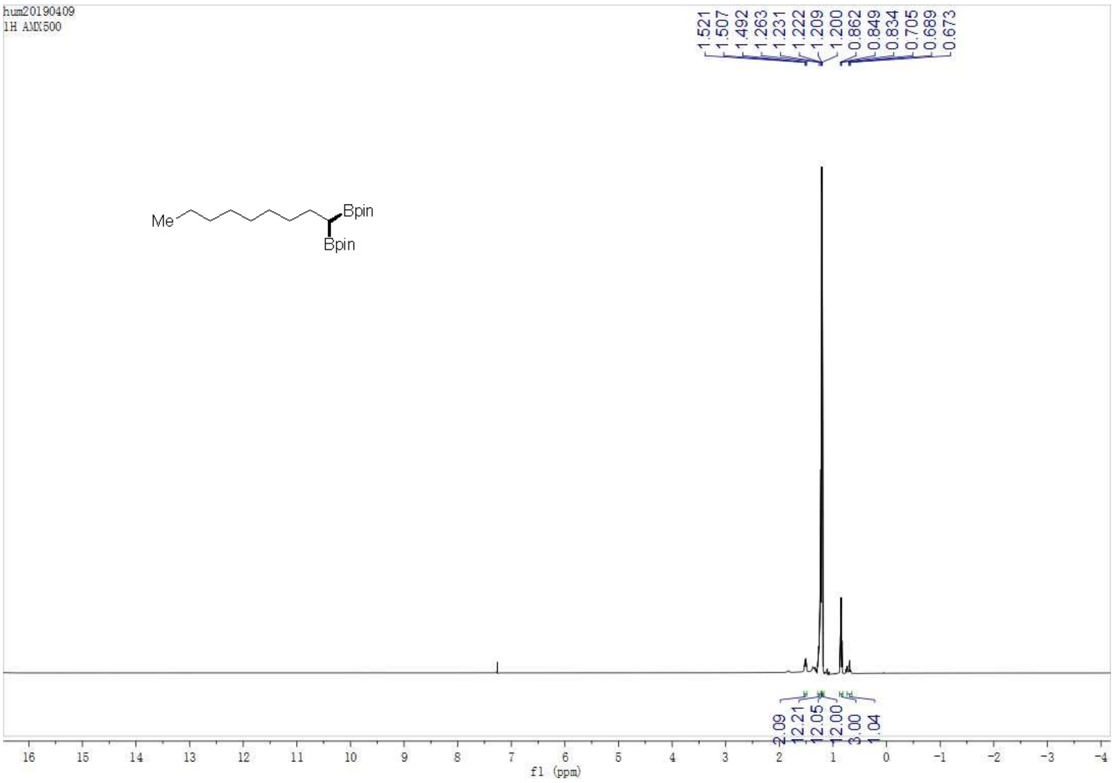

Supplementary Figure 73. ^{1}H NMR spectrum of compound **2ae**.

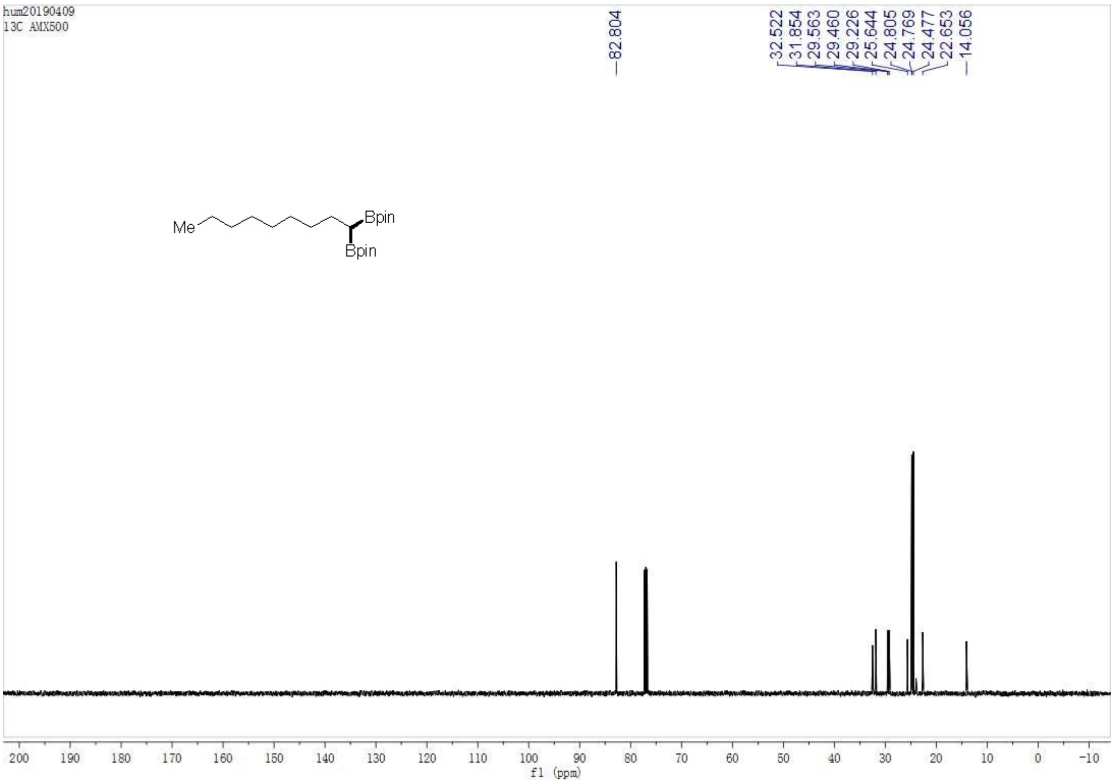

Supplementary Figure 74. ^{13}C{^{1}H} NMR spectrum of compound **2ae**.

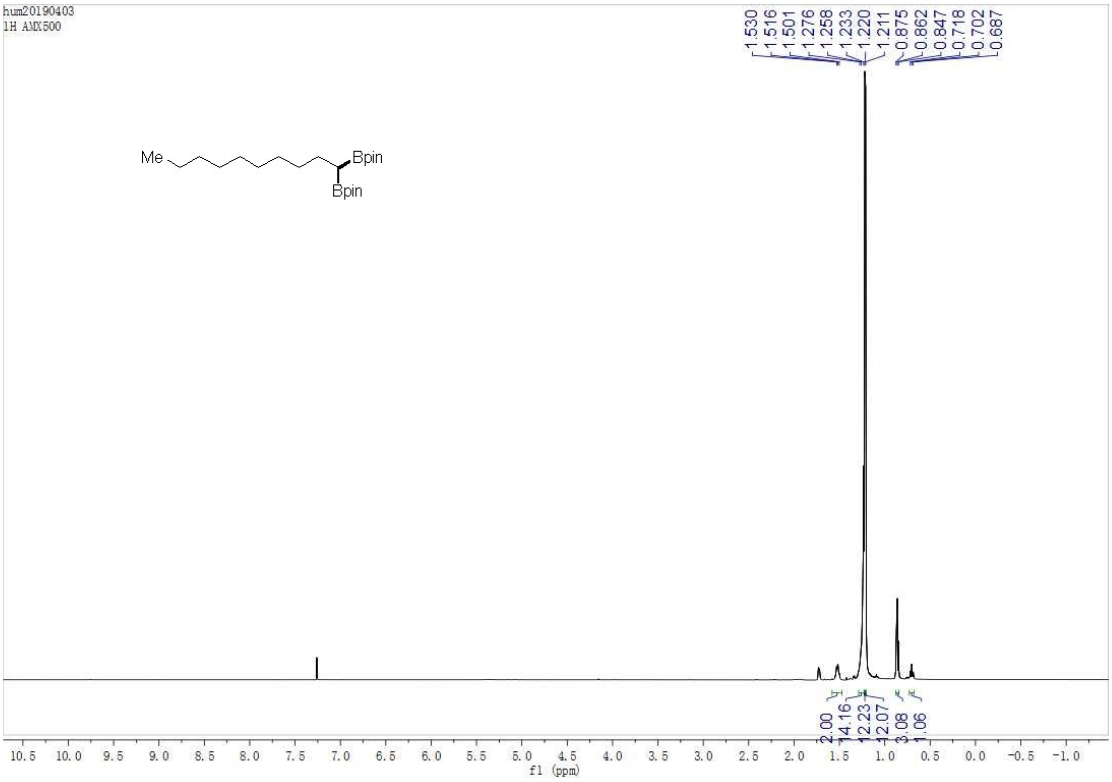

Supplementary Figure 75. ^{1}H NMR spectrum of compound **2af**.

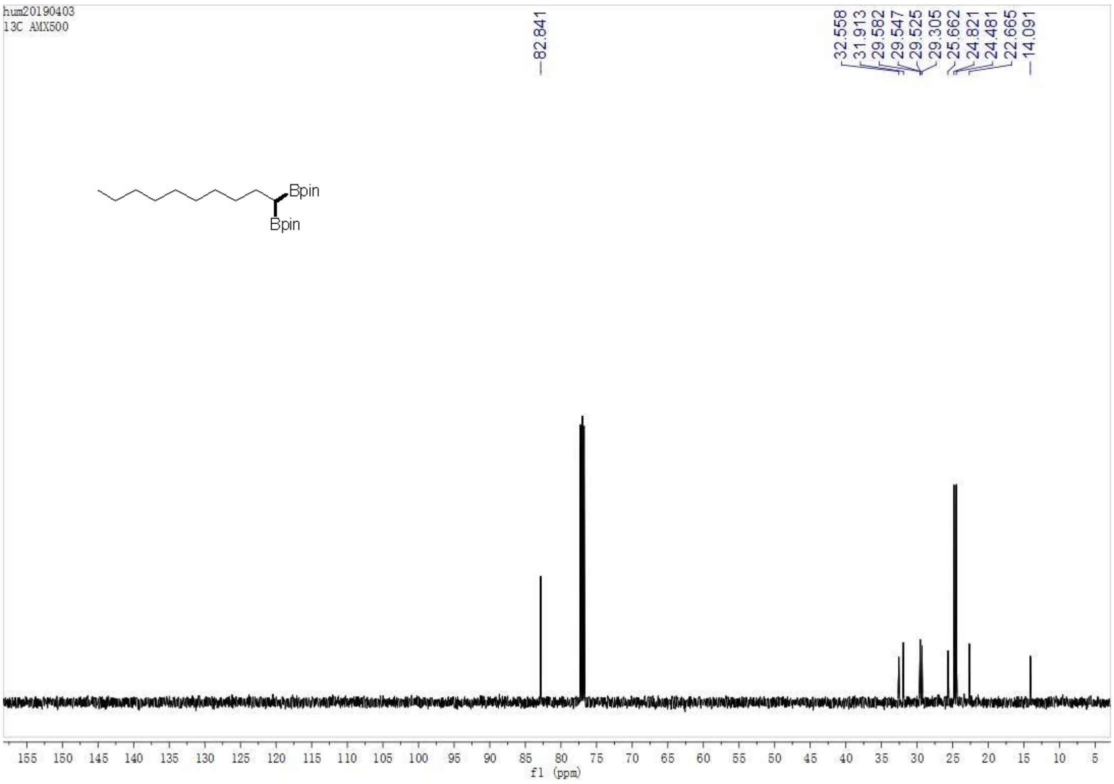

Supplementary Figure 76. $^{13}C\{^{1}H\}$ NMR spectrum of compound **2af**.

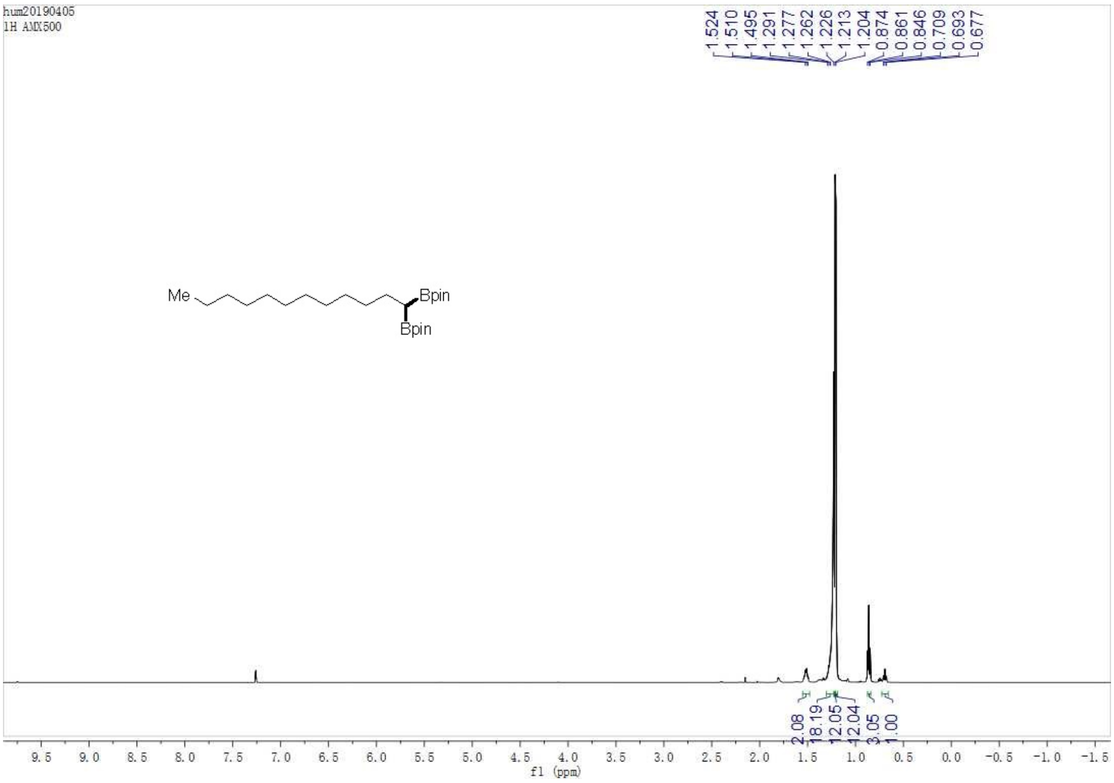

Supplementary Figure 77. ^{1}H NMR spectrum of compound **2ag**.

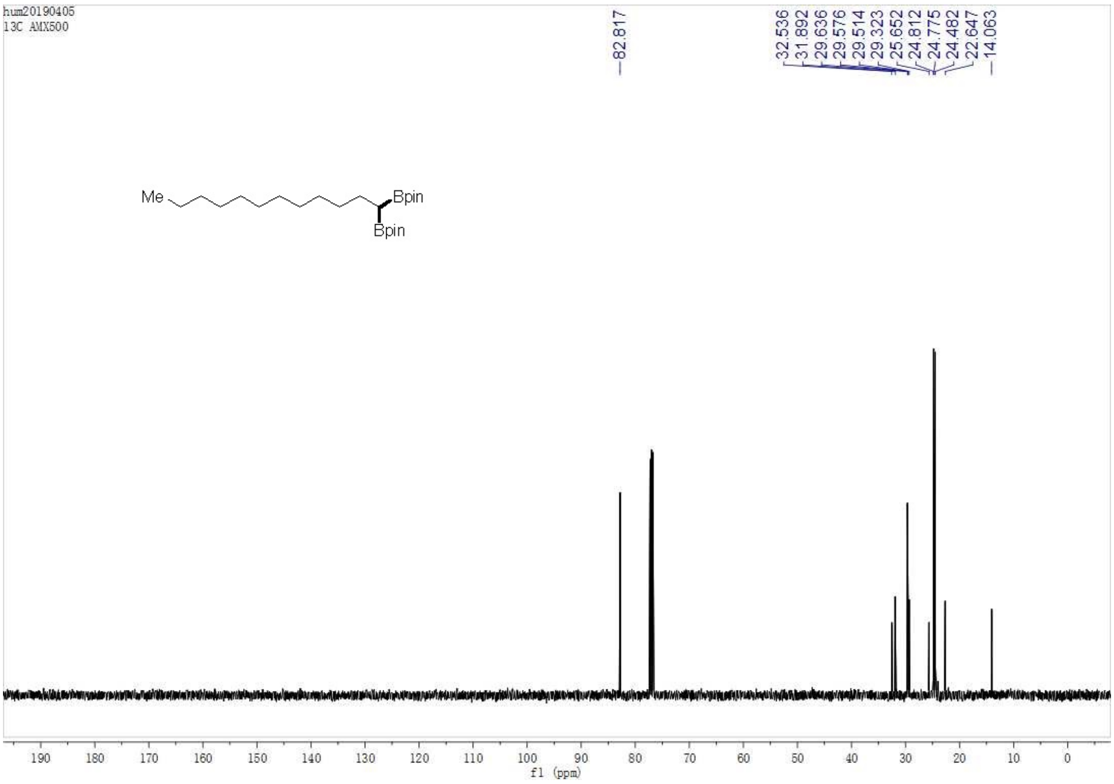

Supplementary Figure 78. $^{13}C\{^{1}H\}$ NMR spectrum of compound **2ag**.

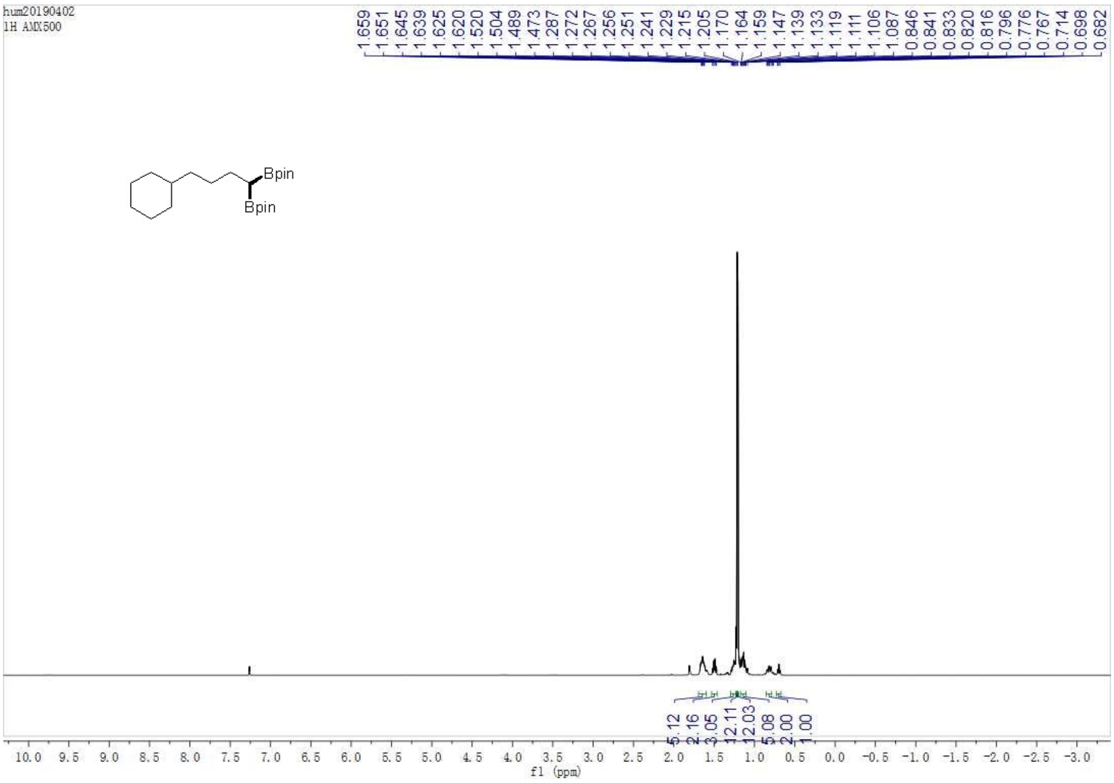

Supplementary Figure 79. ^{1}H NMR spectrum of compound **2ah**.

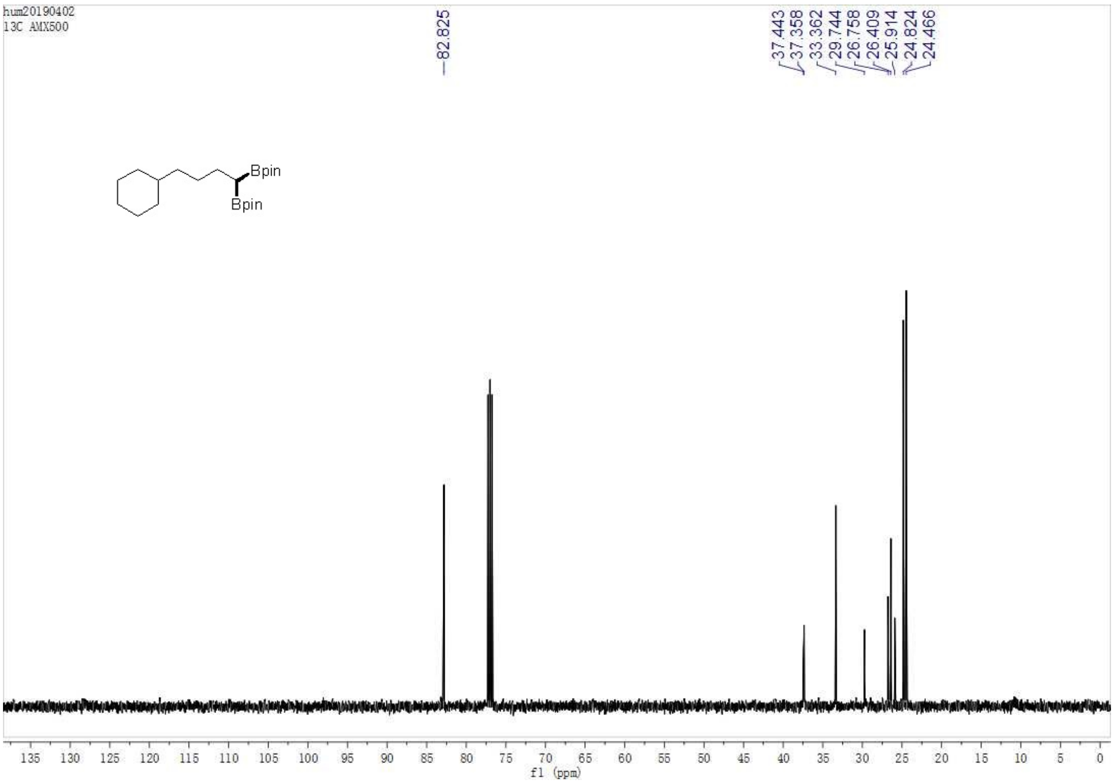

Supplementary Figure 80. ^{13}C{^{1}H} NMR spectrum of compound **2ah**.

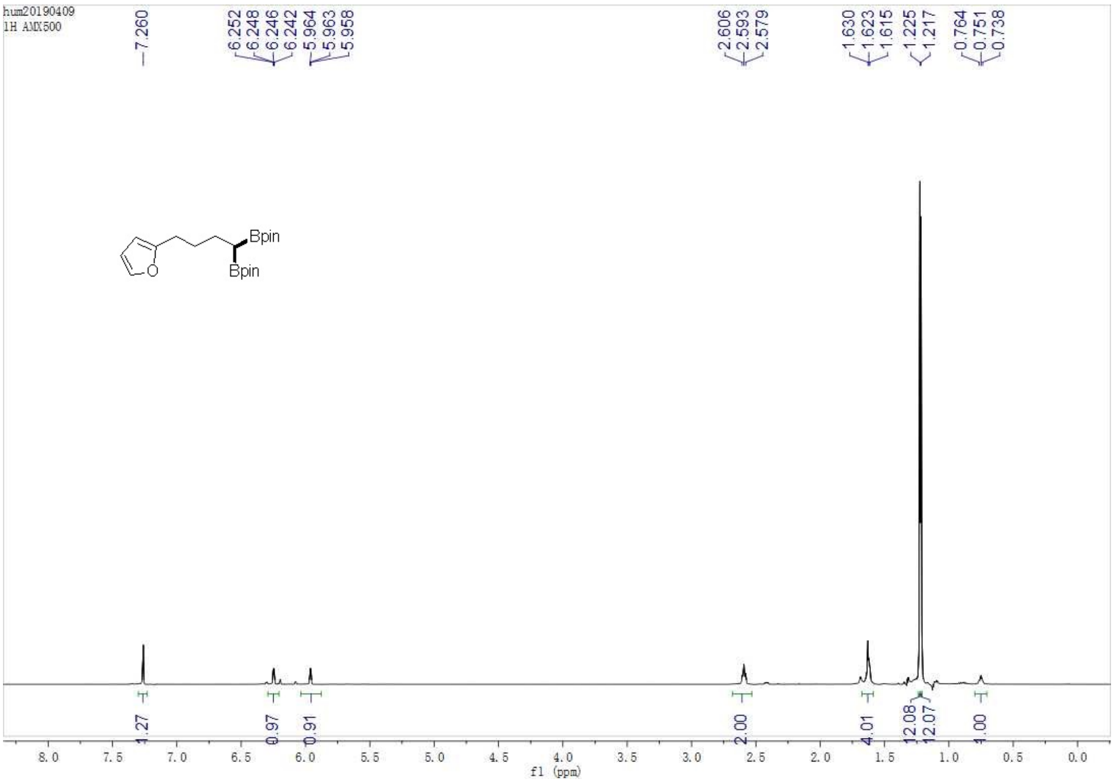

Supplementary Figure 81. ^{1}H NMR spectrum of compound **2aj**.

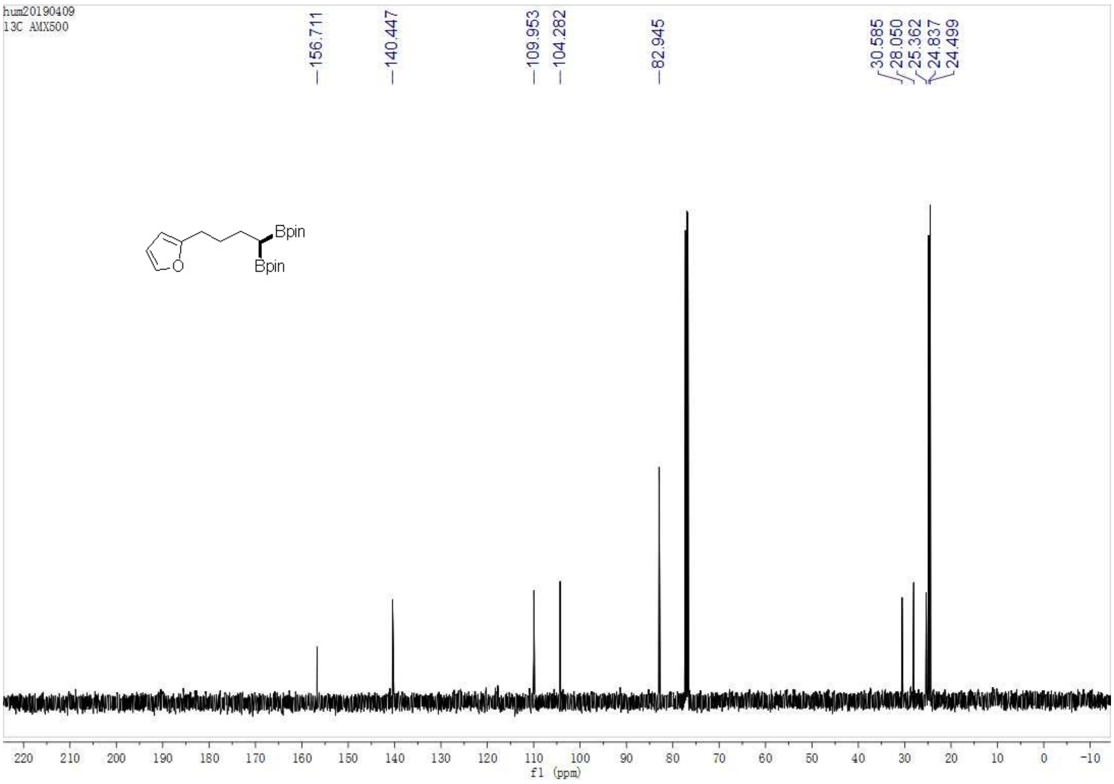

Supplementary Figure 82. ^{13}C{^{1}H} NMR spectrum of compound **2aj**.

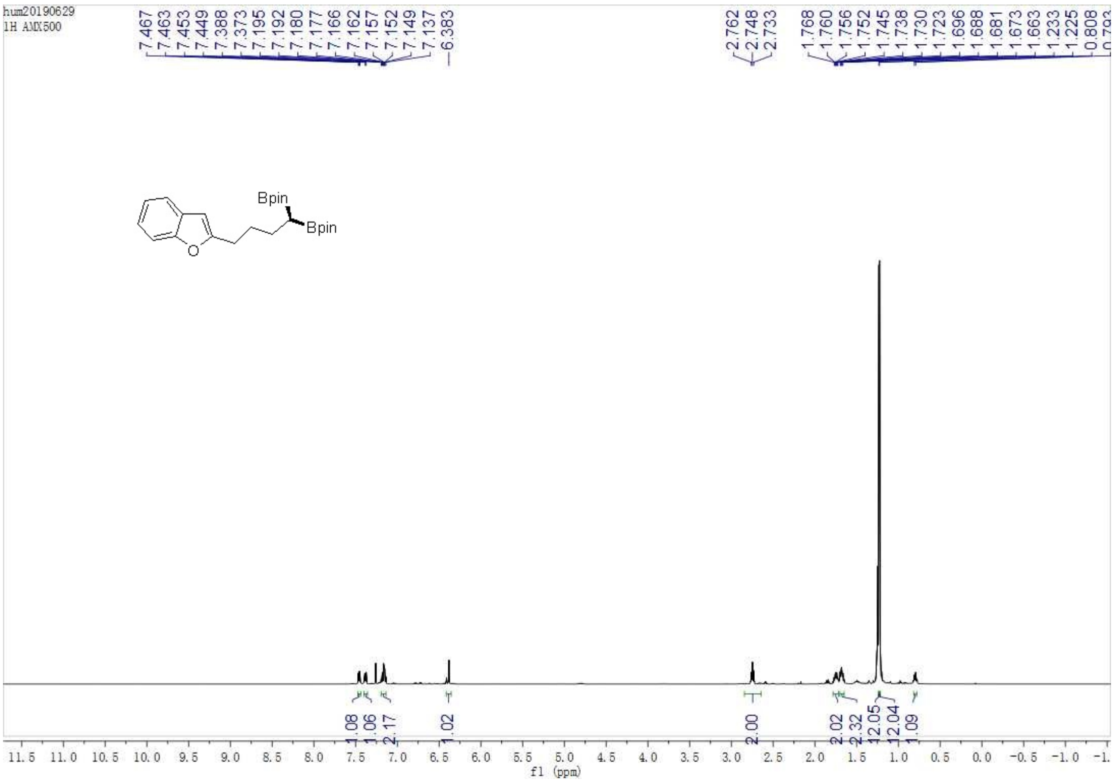

Supplementary Figure 83. ^{1}H NMR spectrum of compound **2ak**.

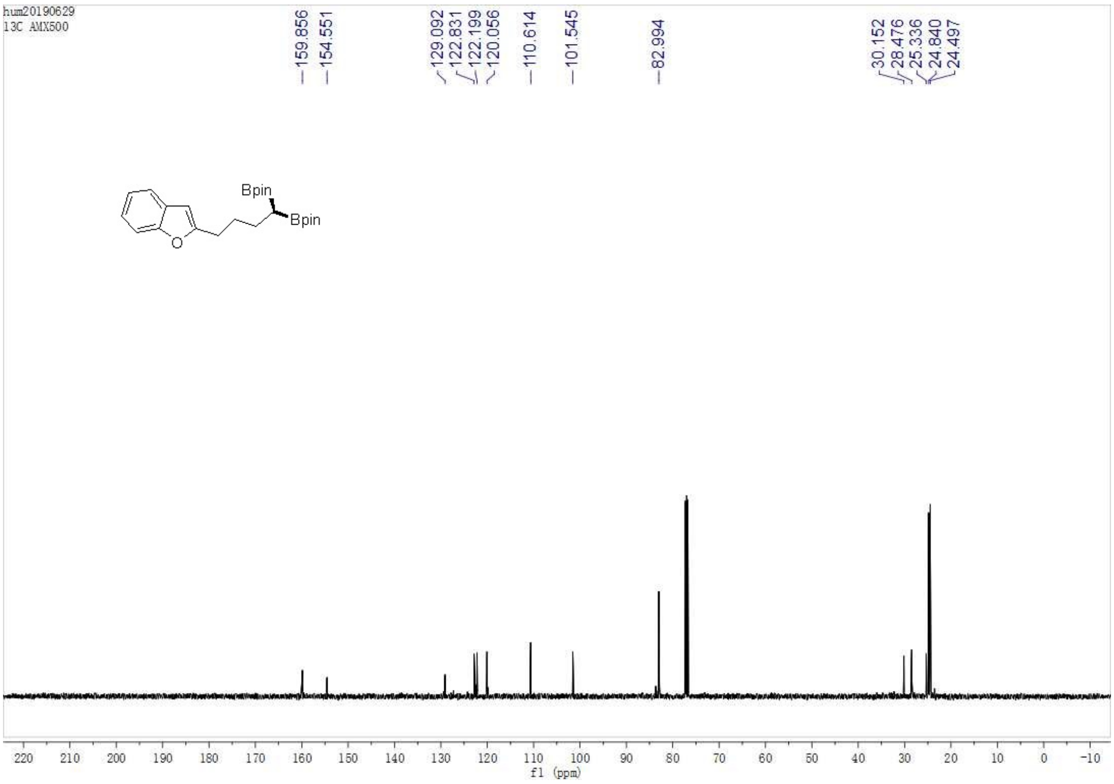

Supplementary Figure 84. ^{13}C{^{1}H} NMR spectrum of compound **2ak**.

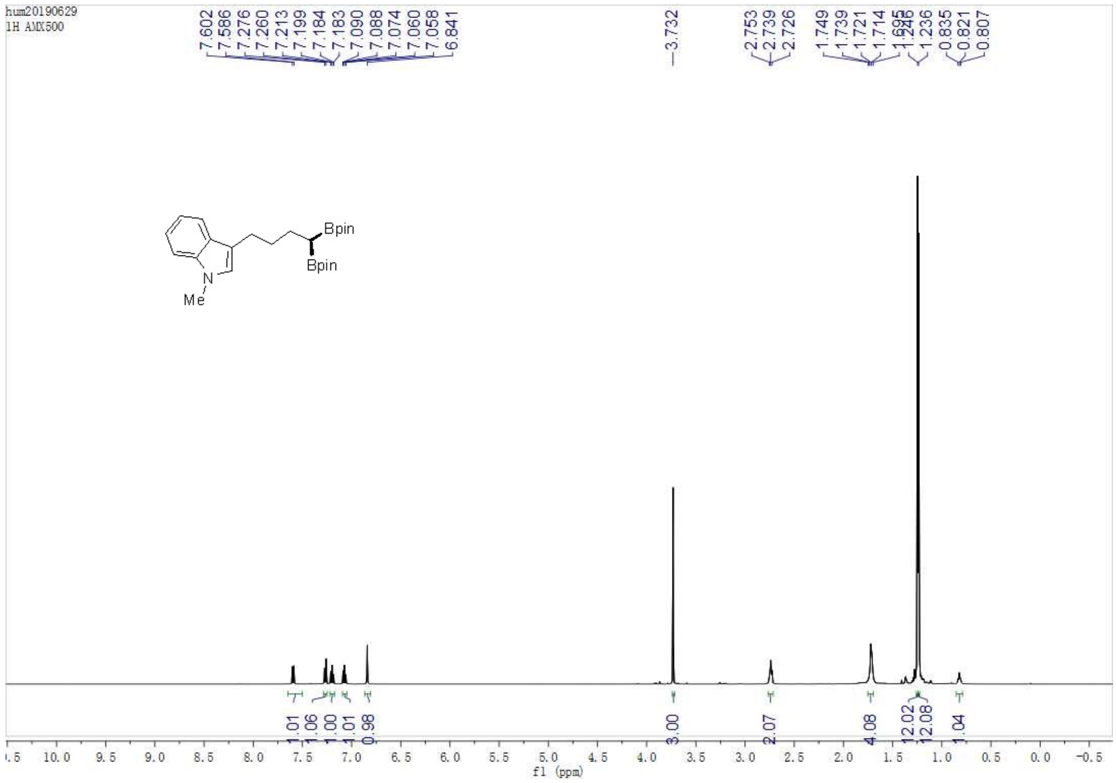

Supplementary Figure 85. ^{1}H NMR spectrum of compound **2al**.

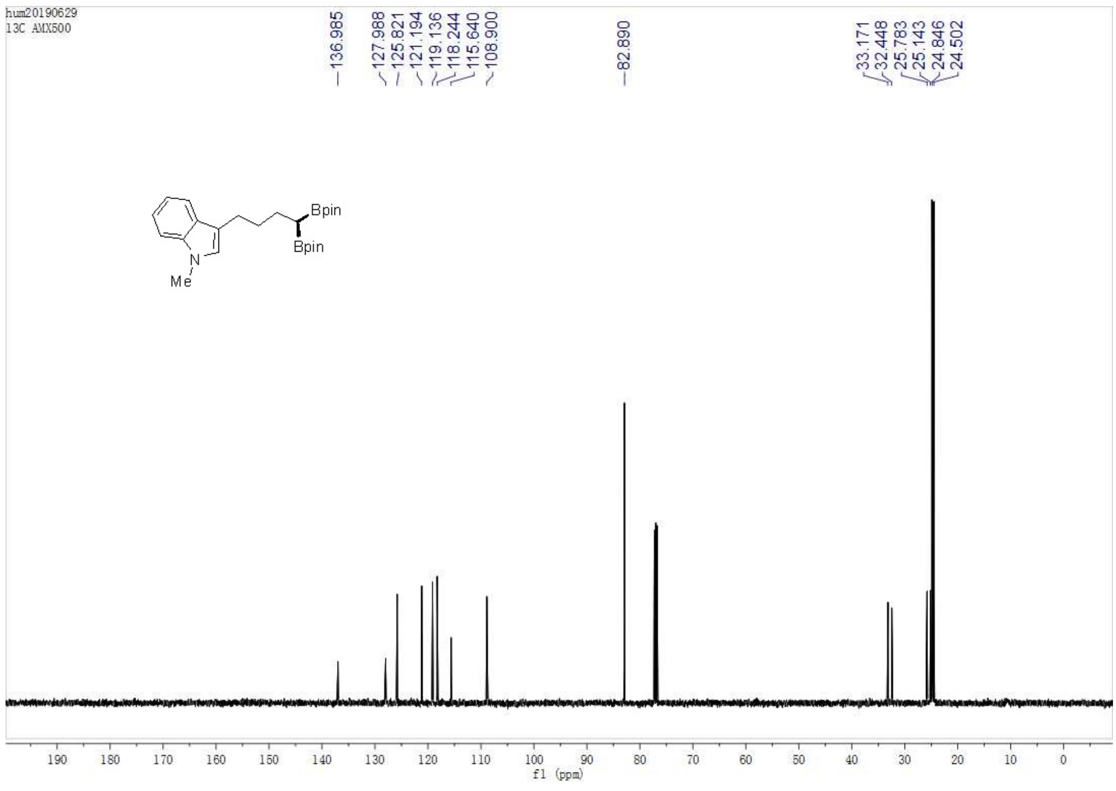

Supplementary Figure 86. $^{13}C\{^{1}H\}$ NMR spectrum of compound **2al**.

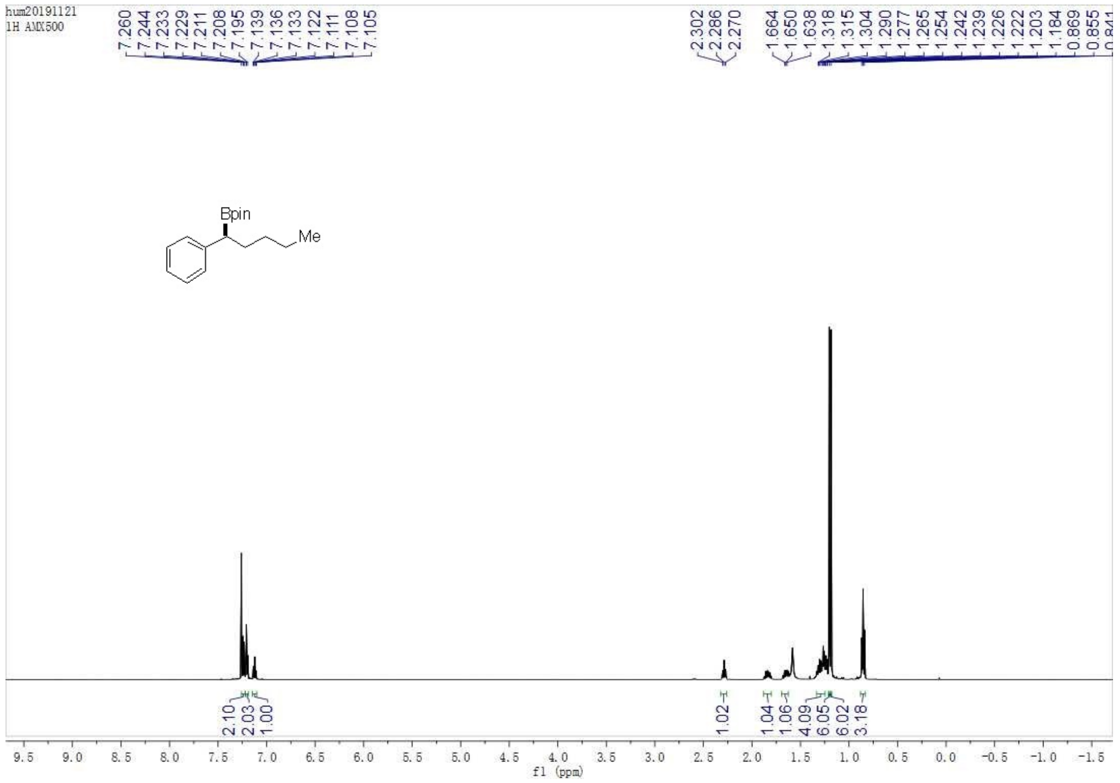

Supplementary Figure 87. ^{1}H NMR spectrum of compound **3**.

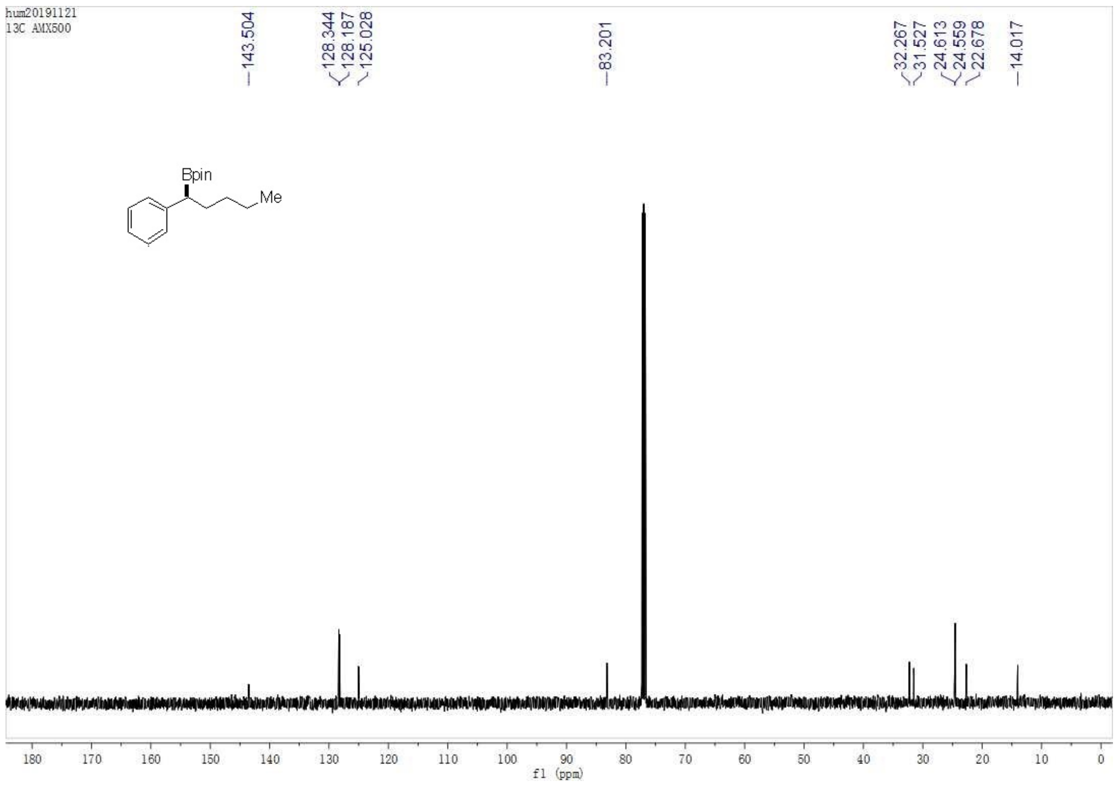

Supplementary Figure 88. $^{13}C\{^{1}H\}$ NMR spectrum of compound **3**.

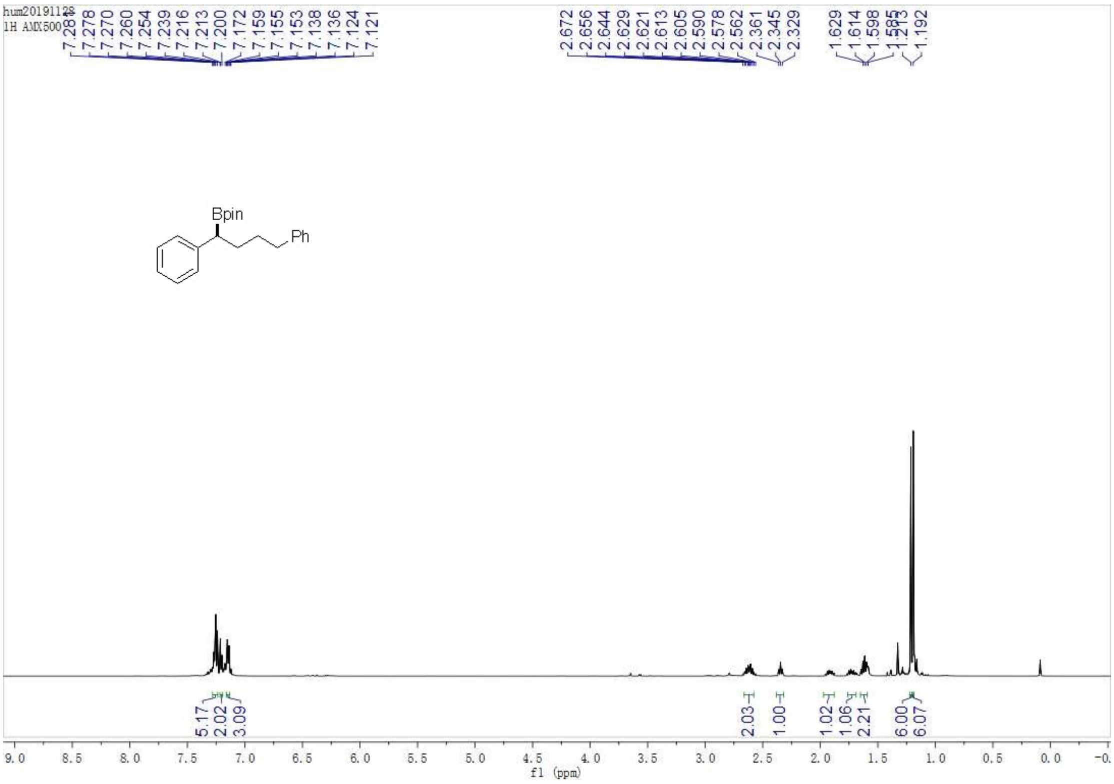

Supplementary Figure 89. ^{1}H NMR spectrum of compound **4**.

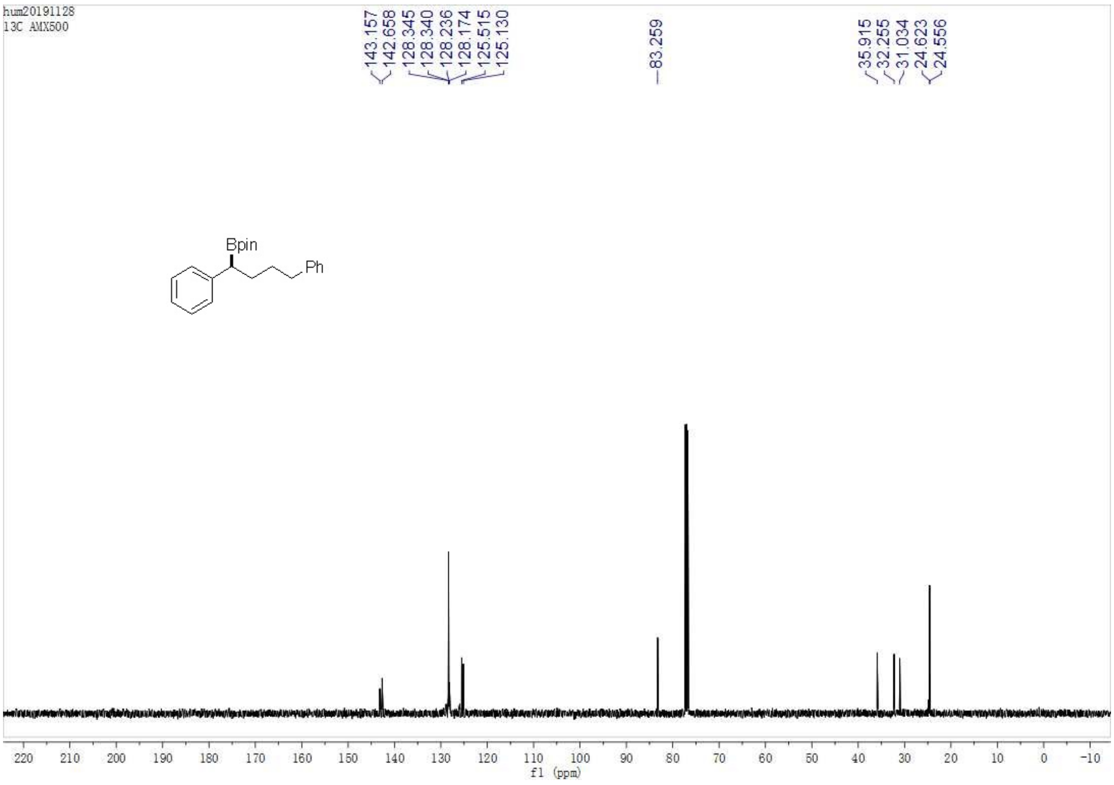

Supplementary Figure 90. $^{13}C\{^{1}H\}$ NMR spectrum of compound **4**.

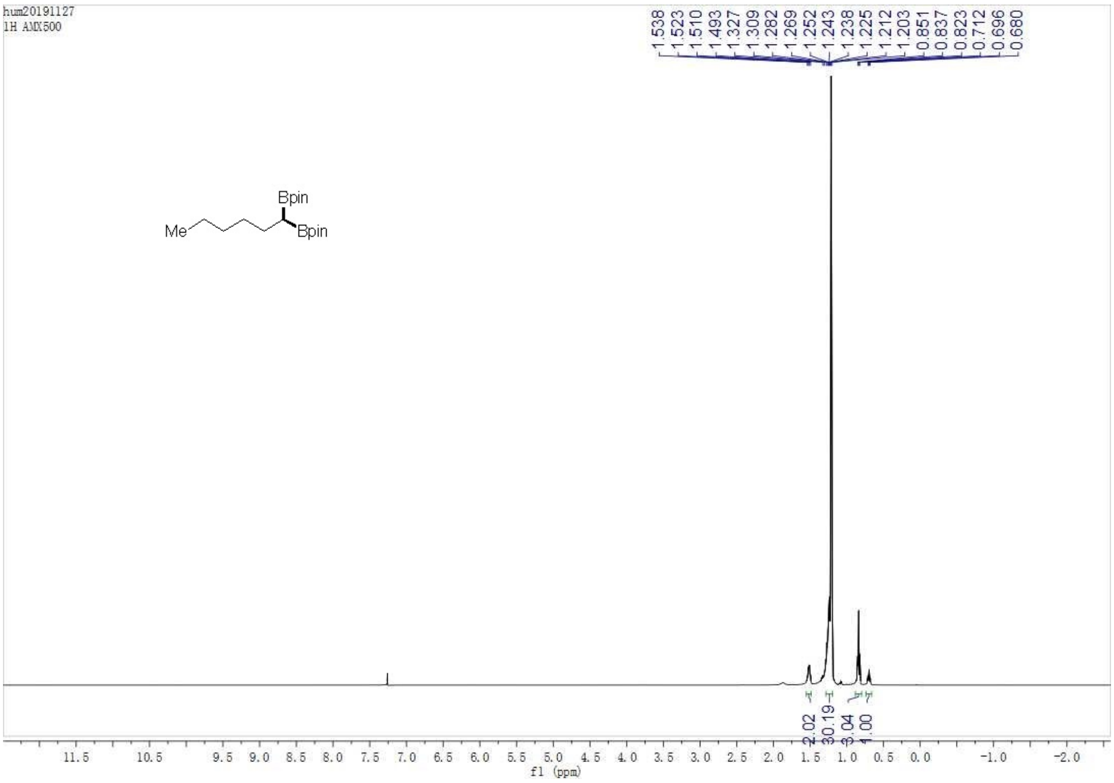

Supplementary Figure 91. ^{1}H NMR spectrum of compound **2ap**.

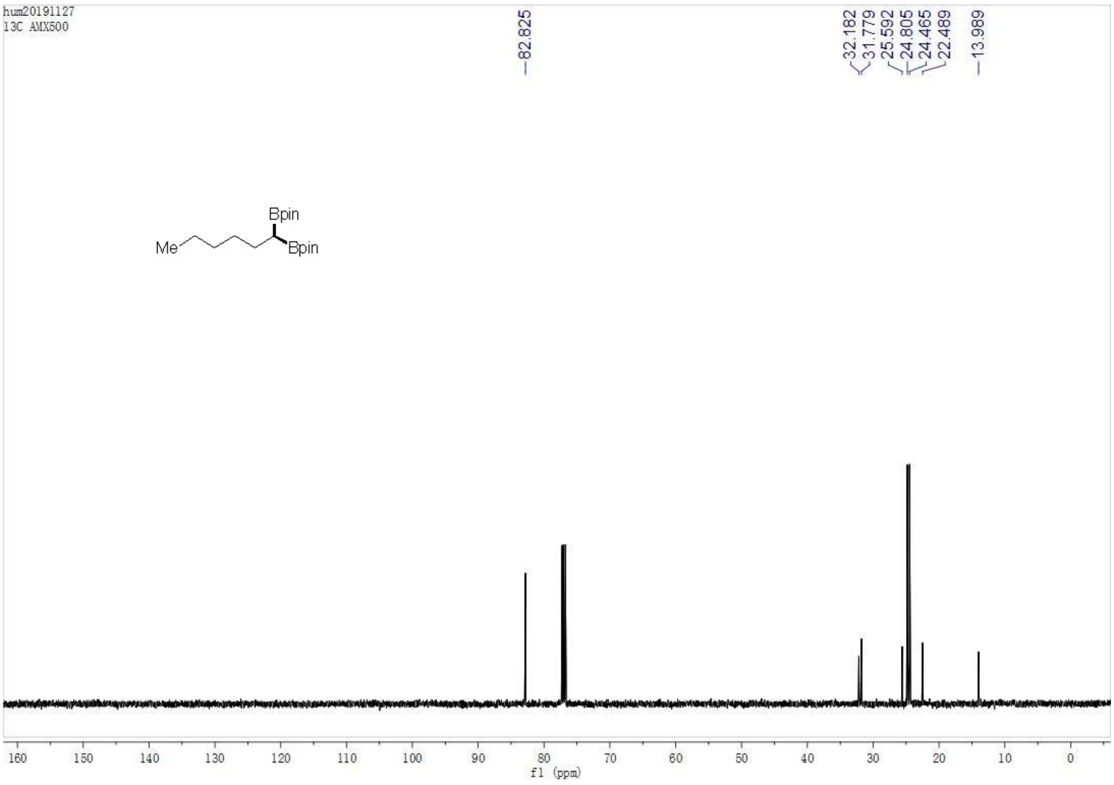

Supplementary Figure 92. $^{13}C\{^{1}H\}$ NMR spectrum of compound **2ap**.

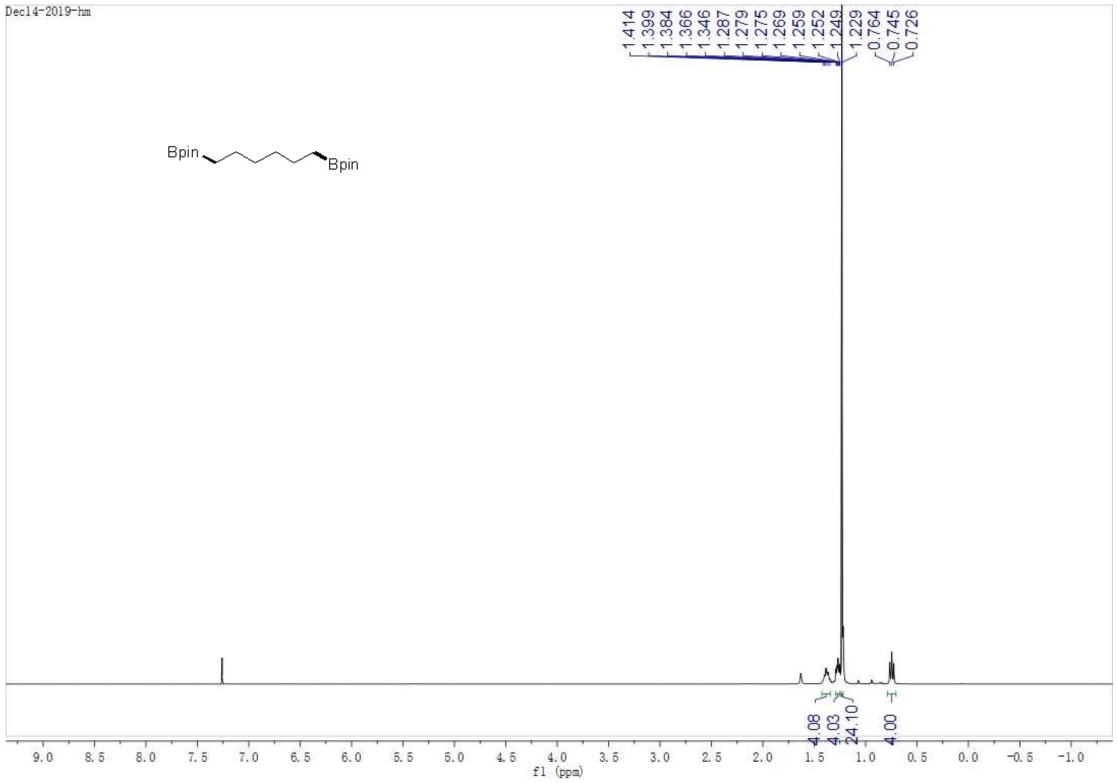

Supplementary Figure 93. ^{1}H NMR spectrum of compound **2ap'**.

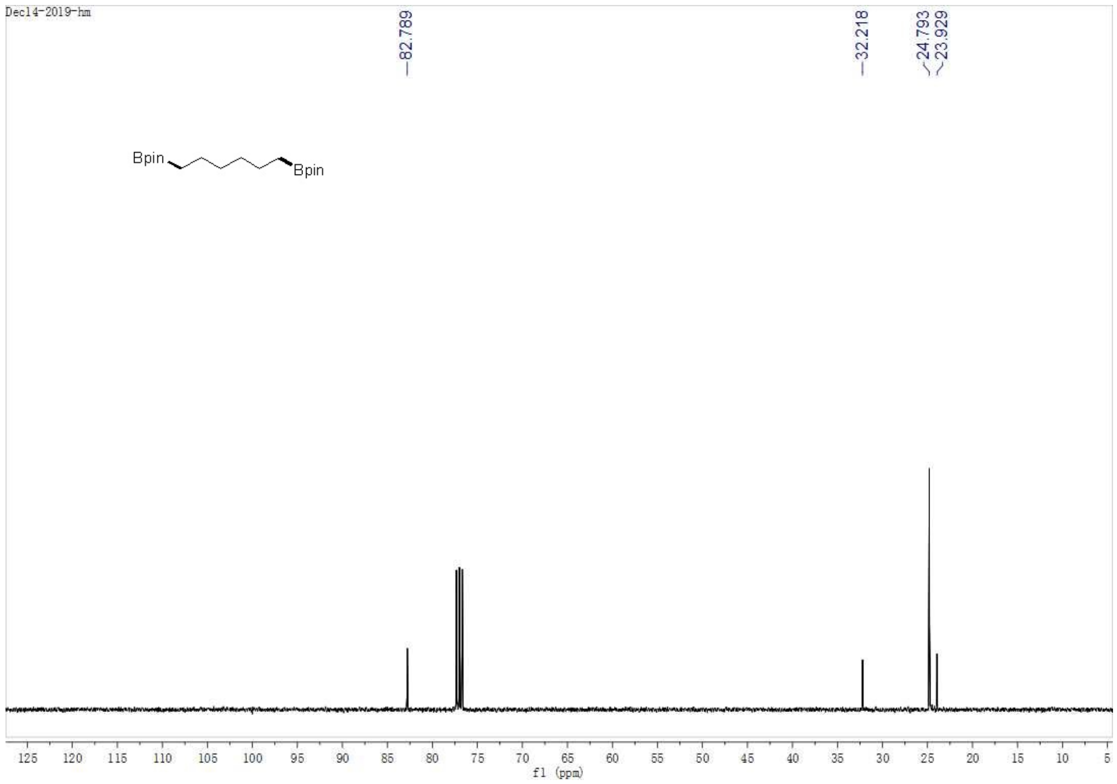

Supplementary Figure 94. $^{13}C\{^{1}H\}$ NMR spectrum of compound **2ap'**.

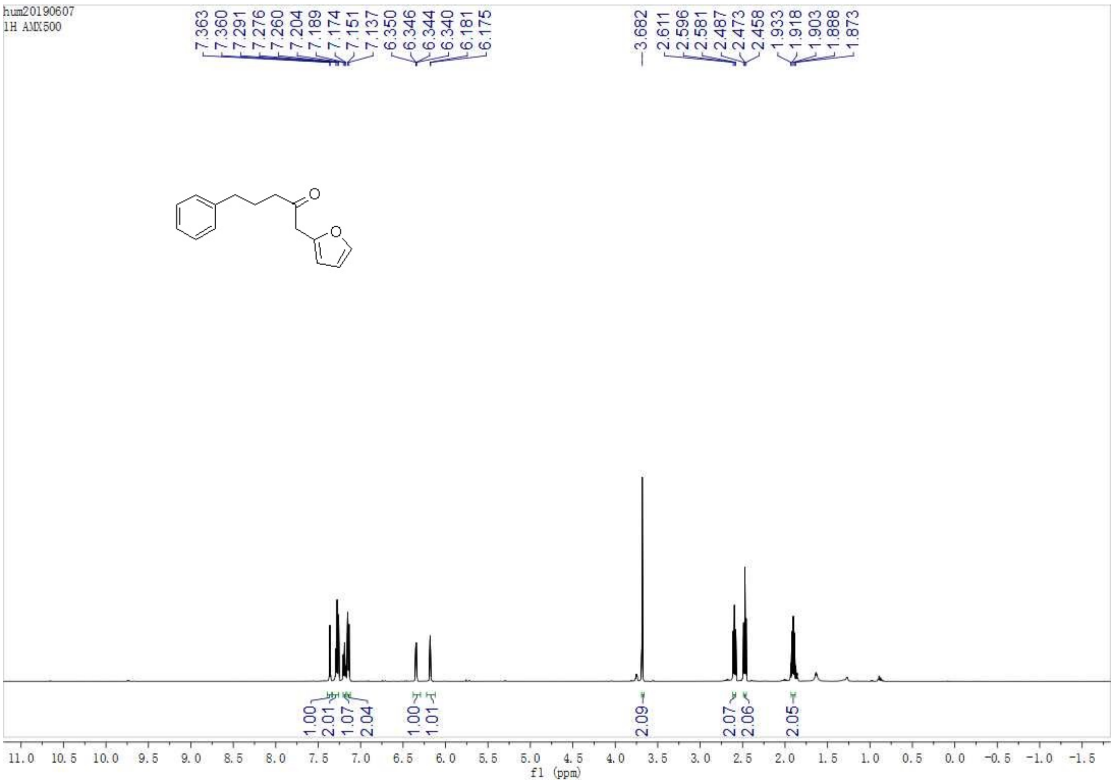

Supplementary Figure 95. ^{1}H NMR spectrum of compound **5**.

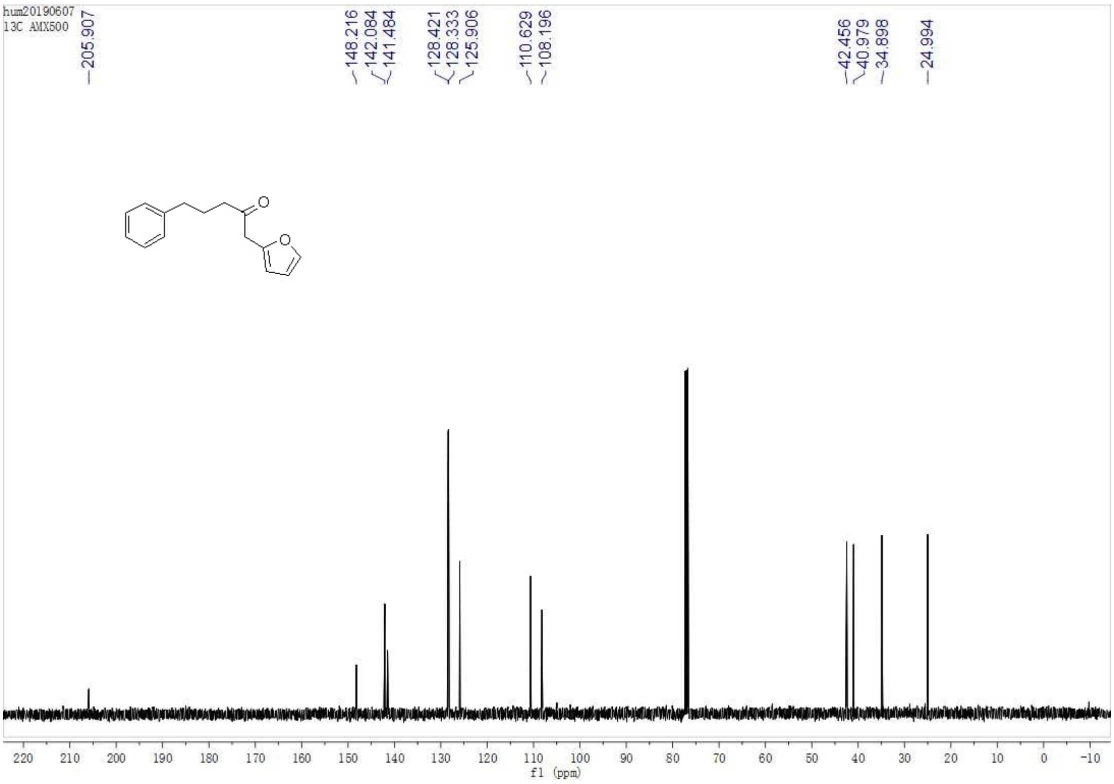

Supplementary Figure 96. ^{13}C{^{1}H} NMR spectrum of compound **5**.

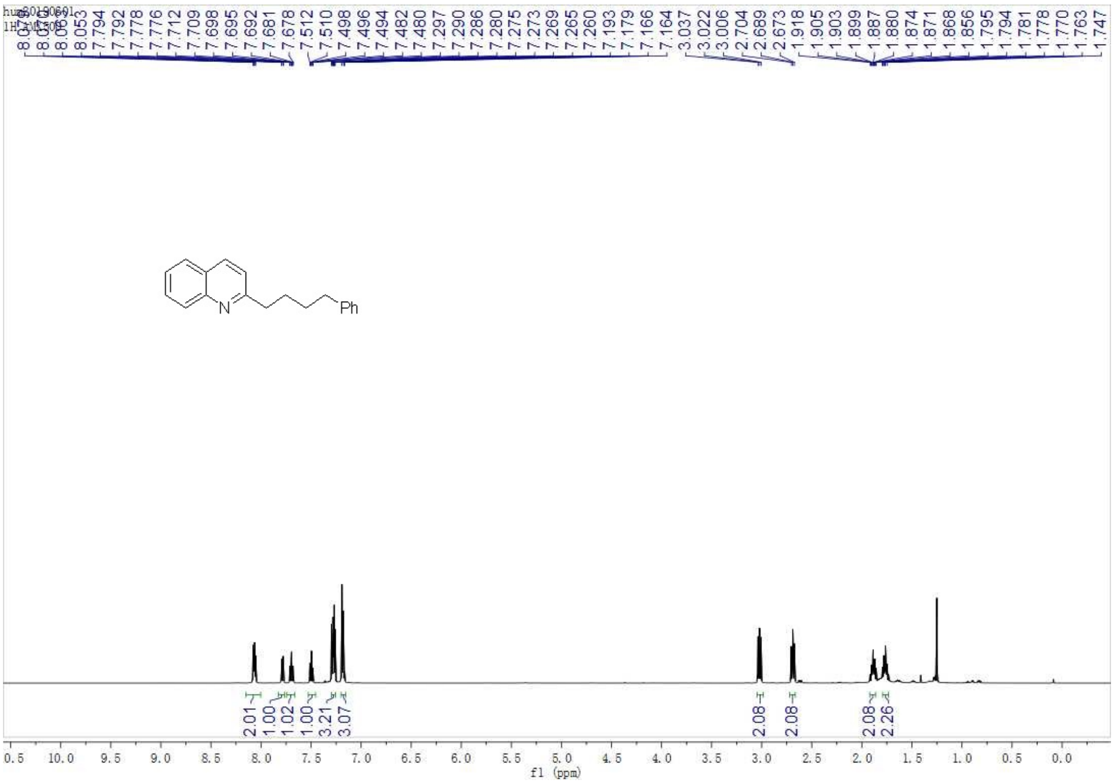

Supplementary Figure 97. ^{1}H NMR spectrum of compound **6**.

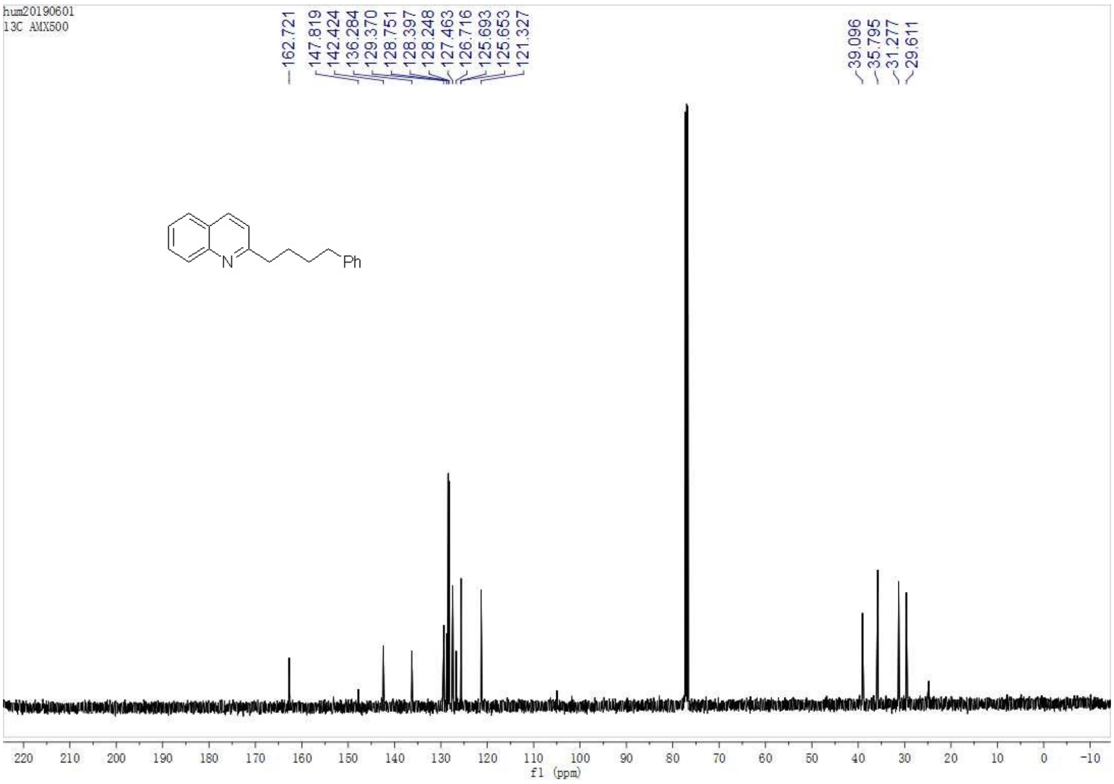

Supplementary Figure 98. $^{13}C\{^{1}H\}$ NMR spectrum of compound **6**.

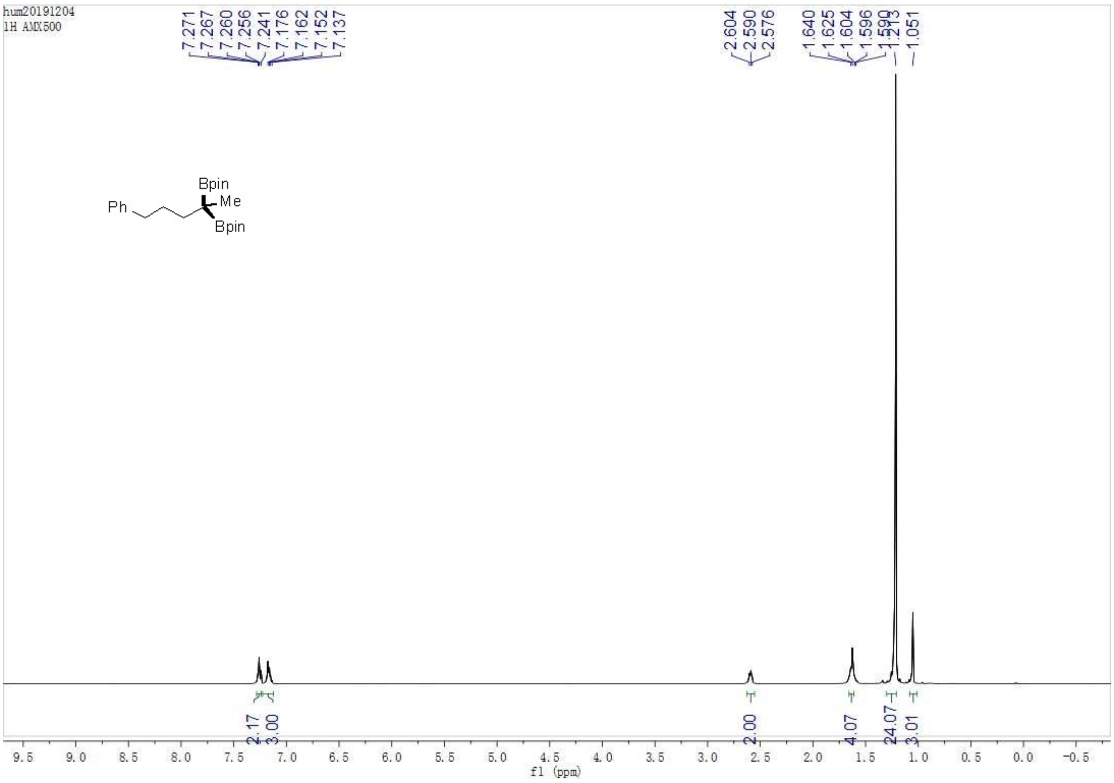

Supplementary Figure 99. ^{1}H NMR spectrum of compound **7**.

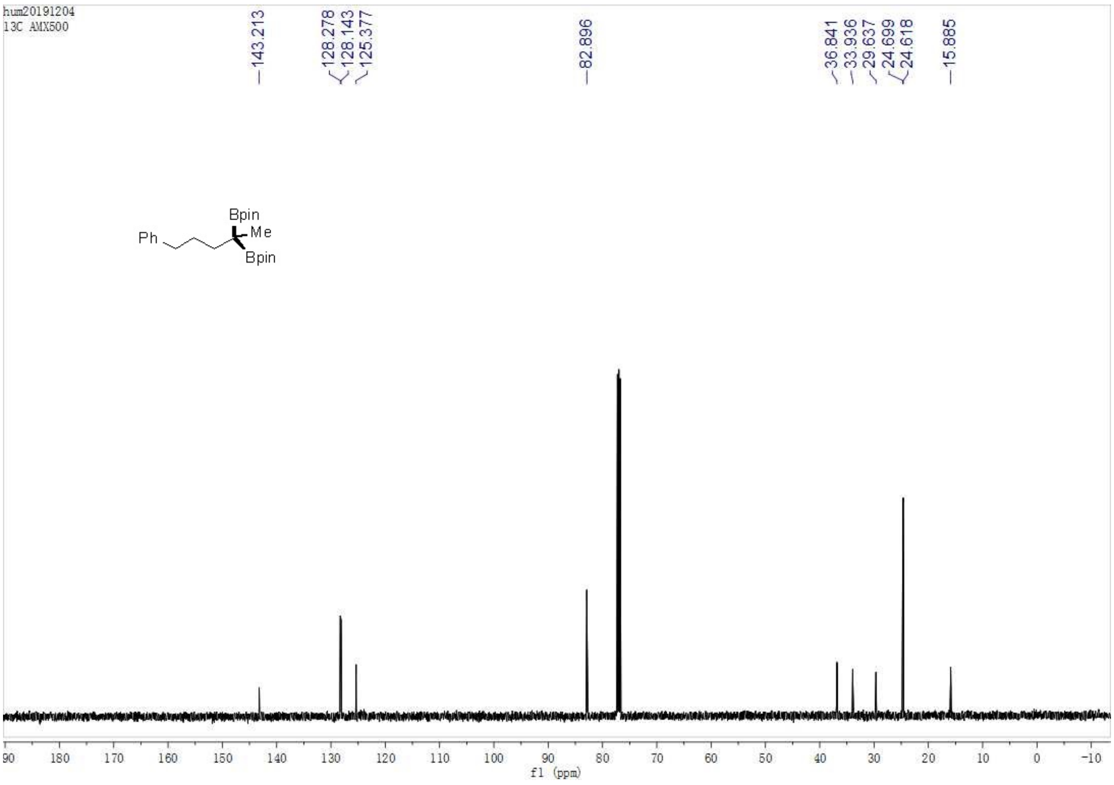

Supplementary Figure 100. $^{13}C\{^{1}H\}$ NMR spectrum of compound **7**.

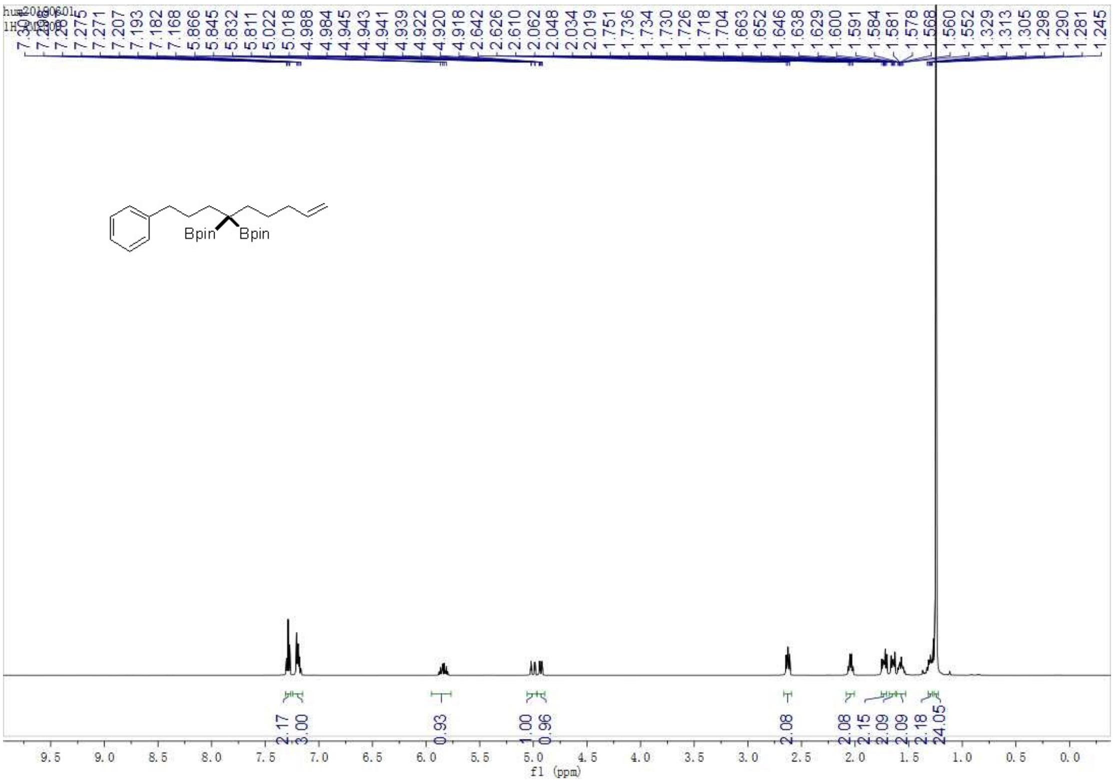

Supplementary Figure 101. ^{1}H NMR spectrum of compound **8**.

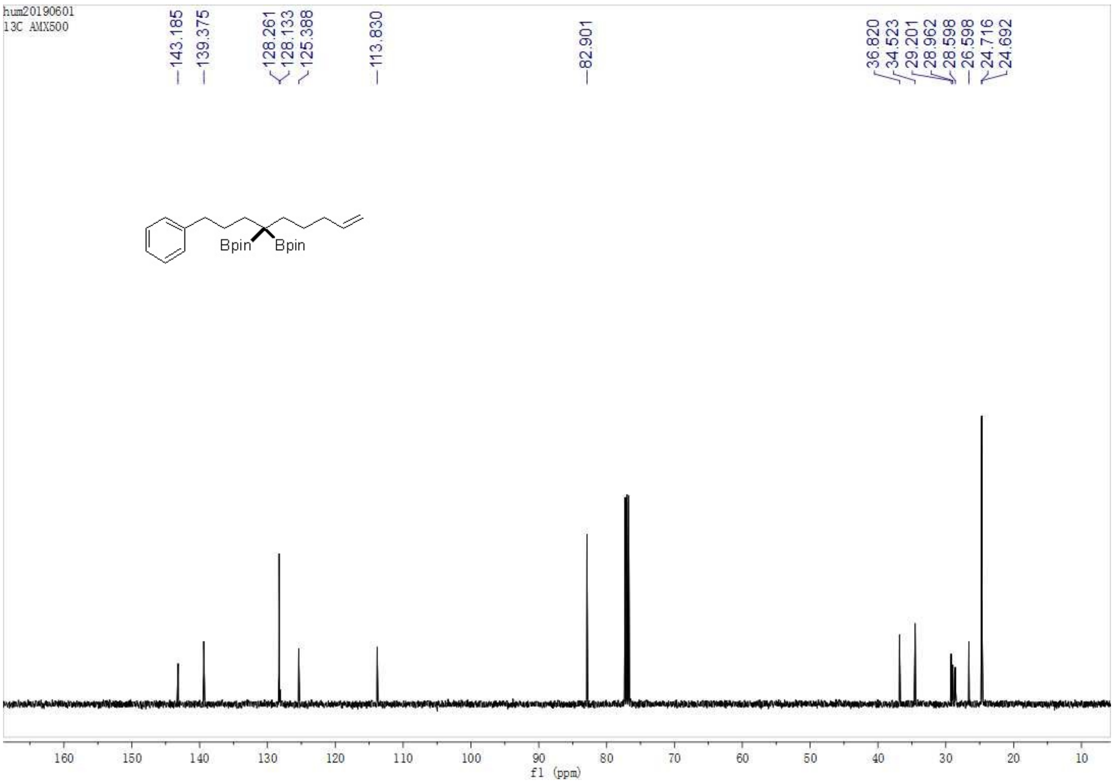

Supplementary Figure 102. $^{13}C\{^{1}H\}$ NMR spectrum of compound **8**.

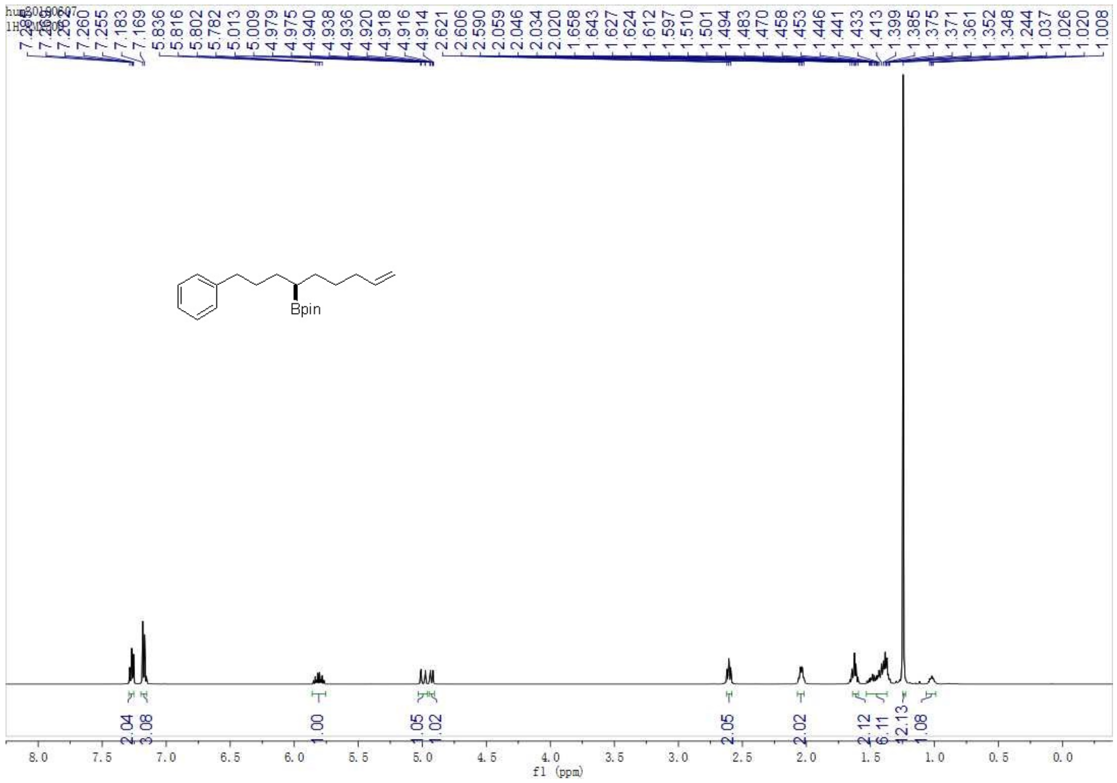

Supplementary Figure 103. ^{1}H NMR spectrum of compound **9**.

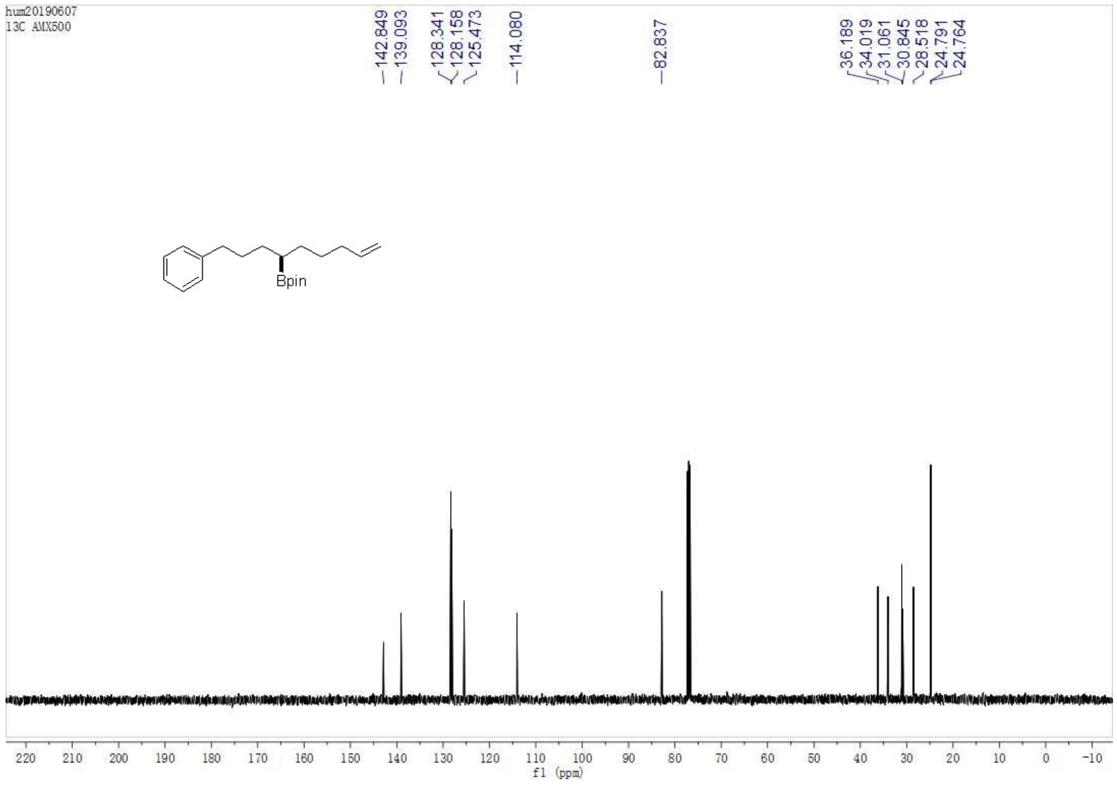

Supplementary Figure 104. $^{13}C\{^{1}H\}$ NMR spectrum of compound **9**.

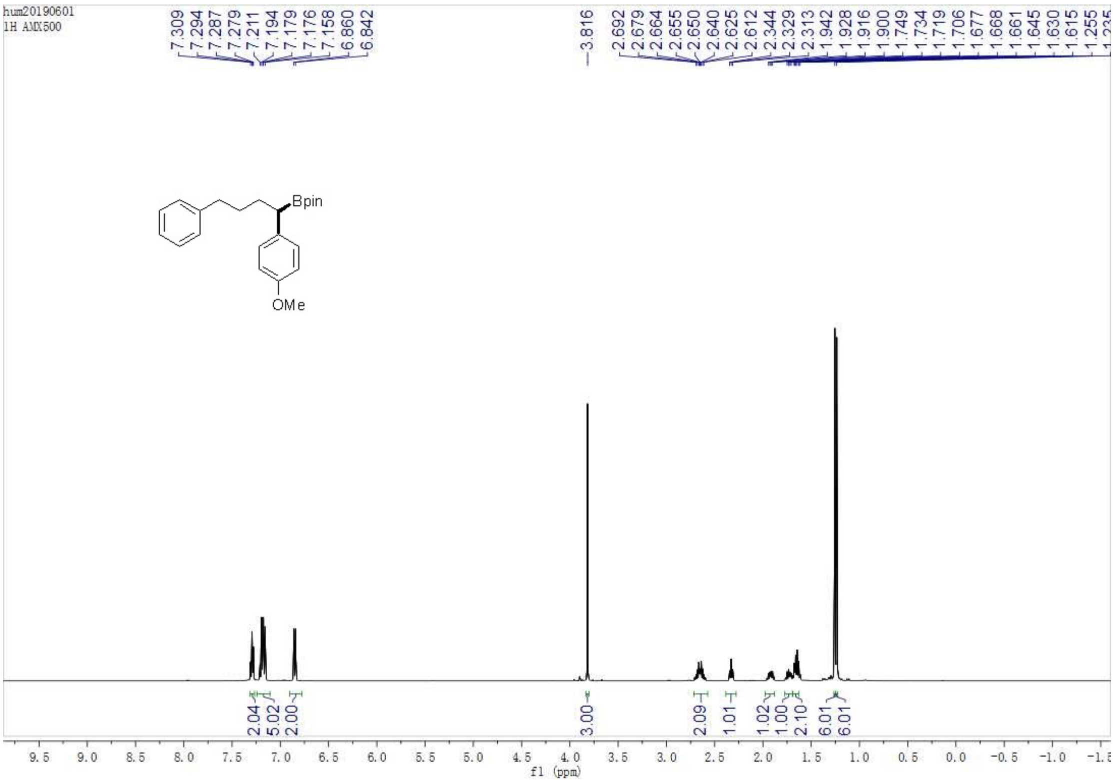

Supplementary Figure 105. ^{1}H NMR spectrum of compound **10**.

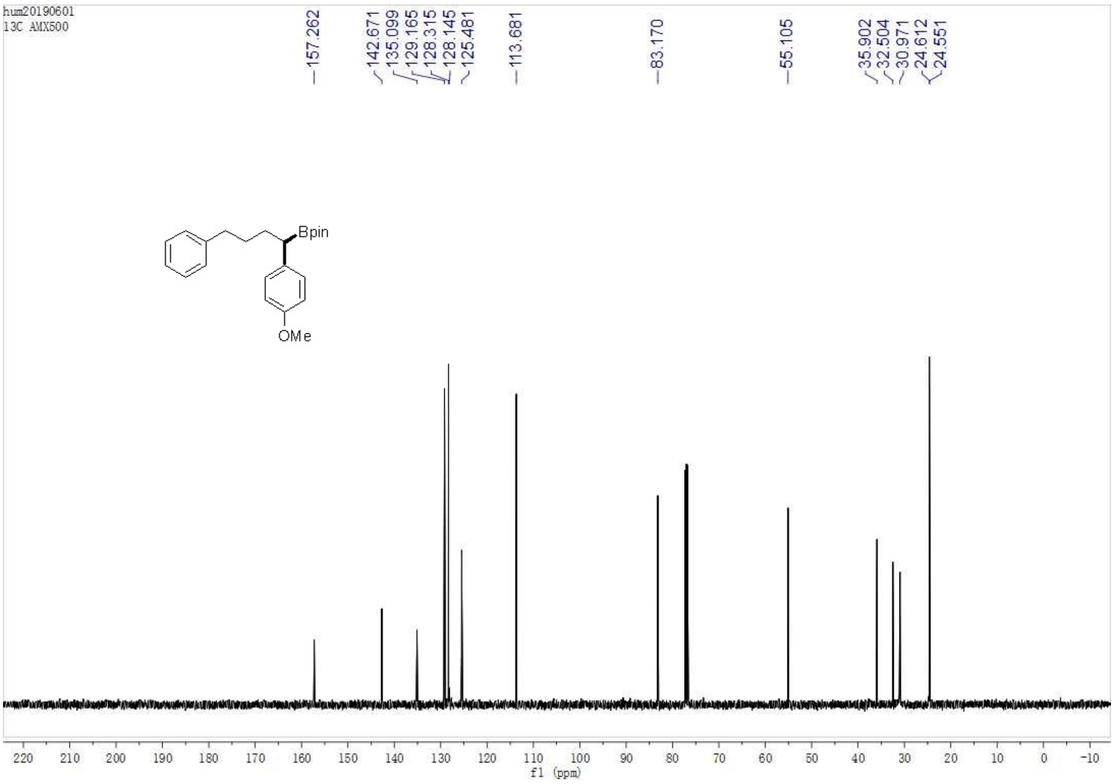

Supplementary Figure 106. $^{13}C\{^{1}H\}$ NMR spectrum of compound **10**.

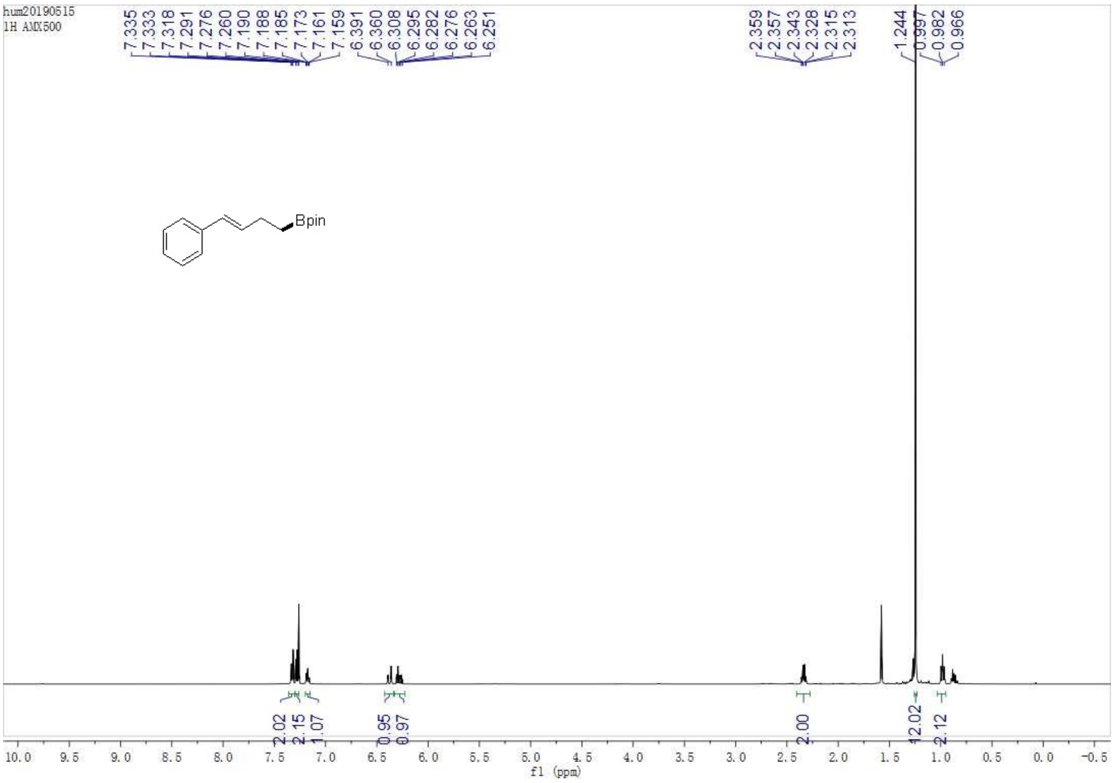

Supplementary Figure 107. ^{1}H NMR spectrum of compound **11s**.

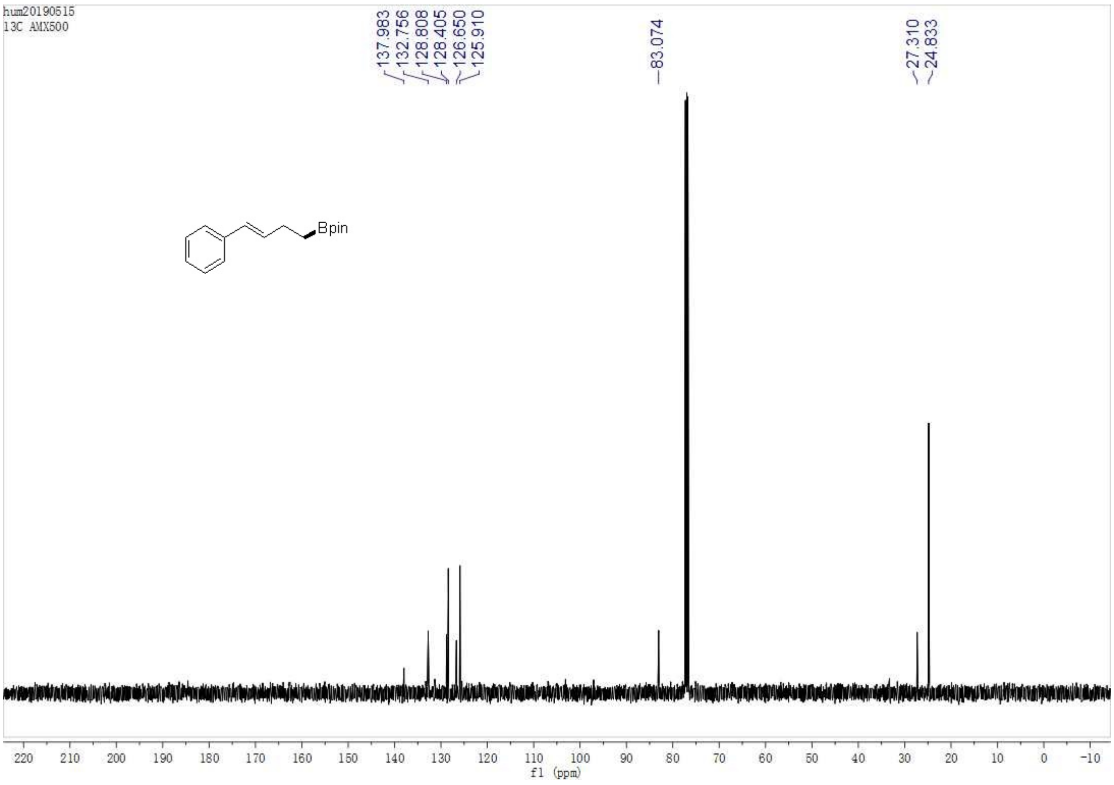

Supplementary Figure 108. $^{13}C\{^{1}H\}$ NMR spectrum of compound **11s**.

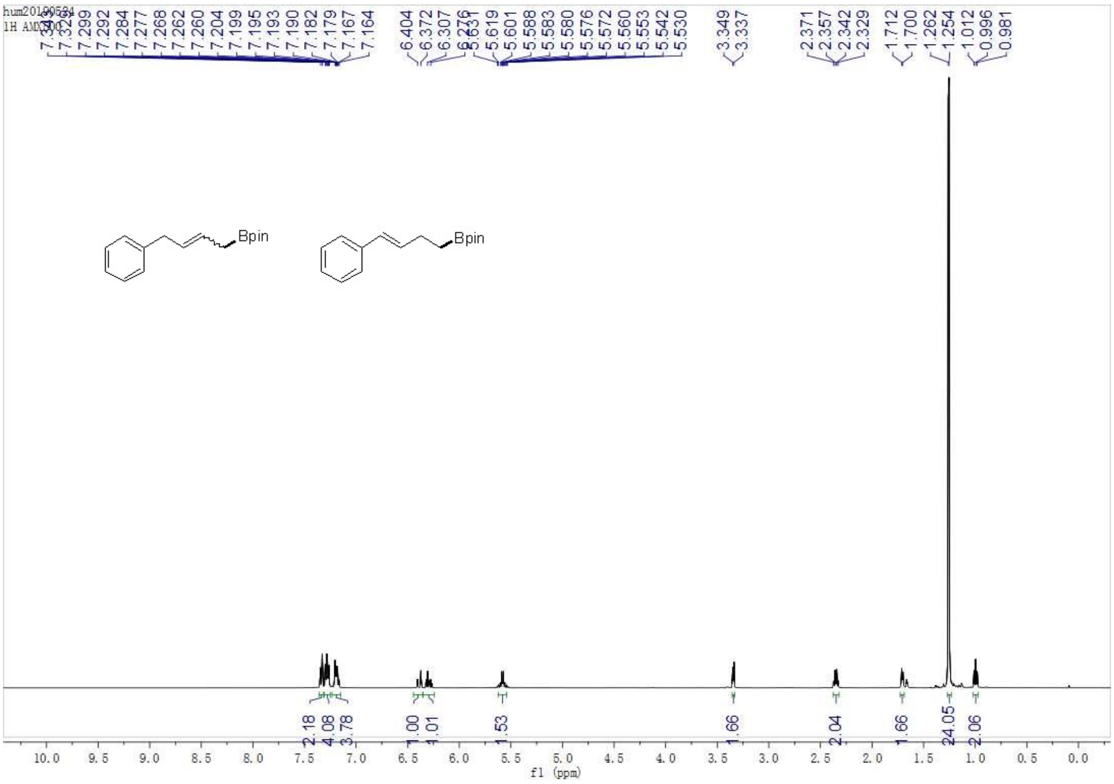

Supplementary Figure 109. ^{1}H NMR spectrum of compounds **11s** and **11s'**.

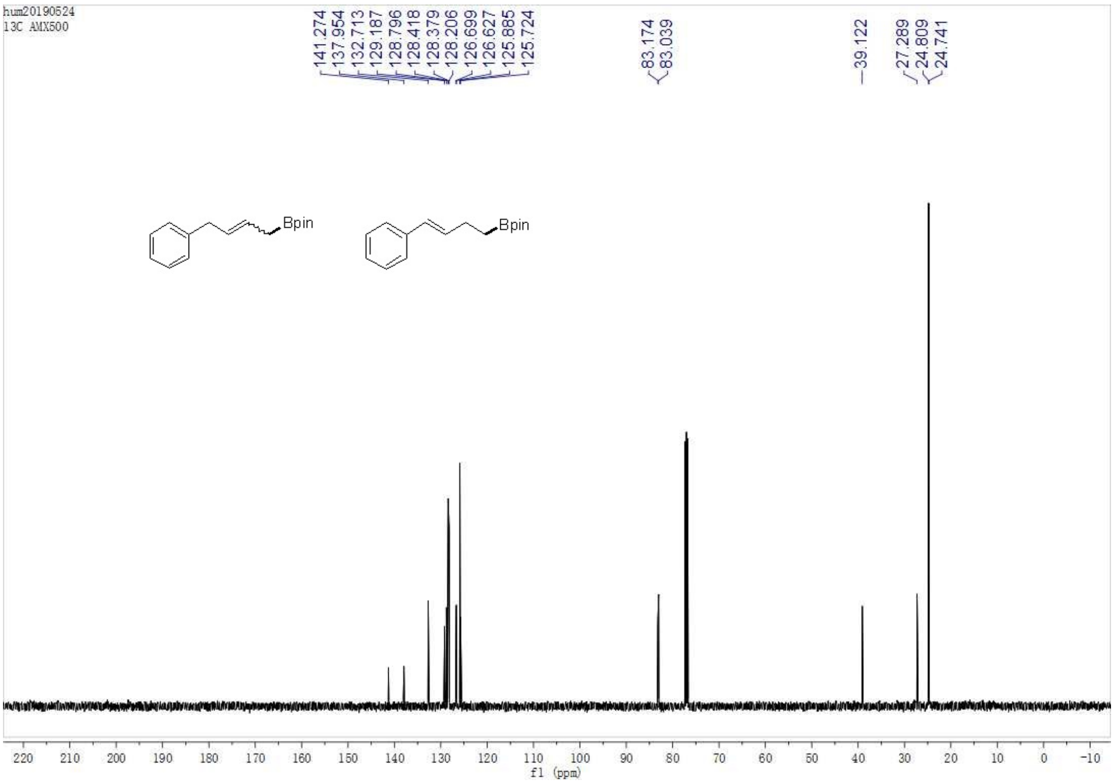

Supplementary Figure 110. $^{13}C\{^{1}H\}$ NMR spectrum of compounds **11s** and **11s'**.

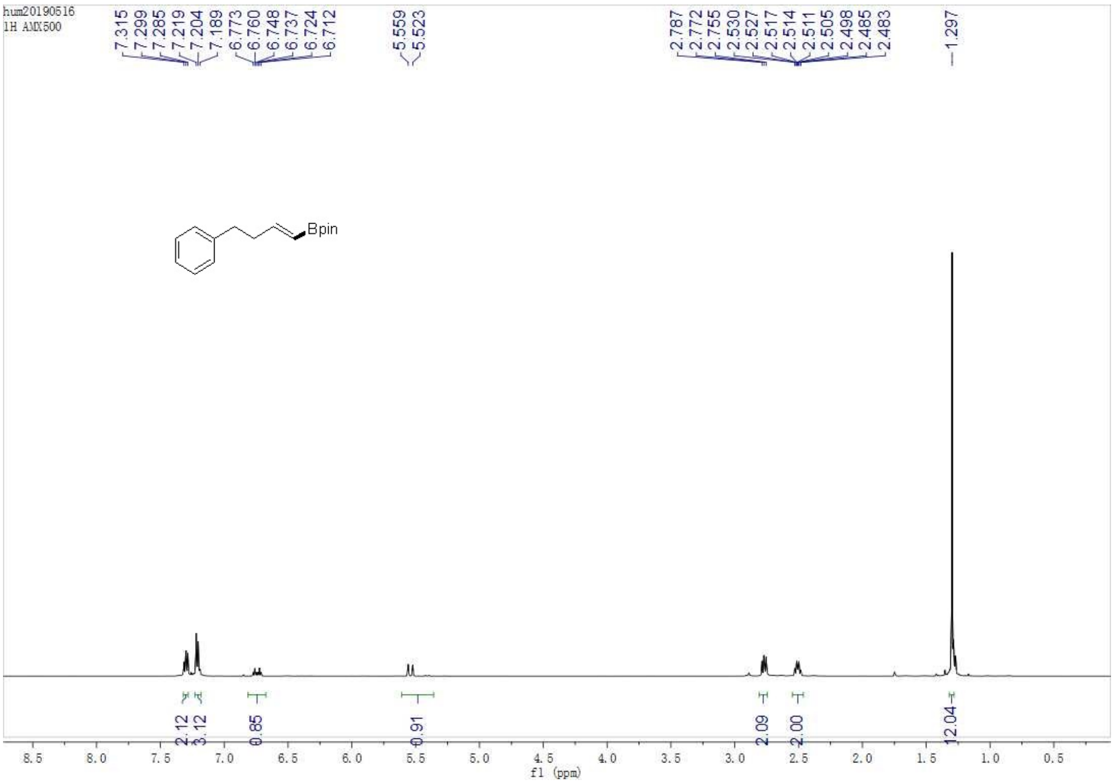

Supplementary Figure 111. ^{1}H NMR spectrum of compound **11s''**.

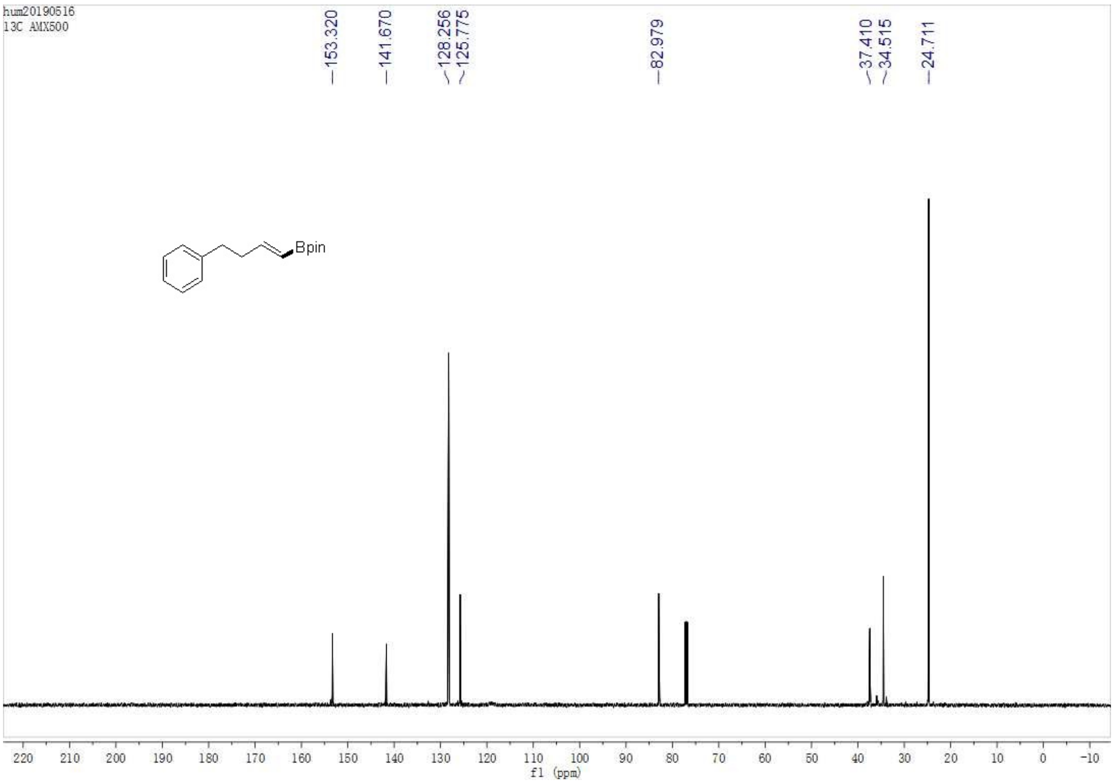

Supplementary Figure 112. ^{13}C{^{1}H} NMR spectrum of compound **11s''**.

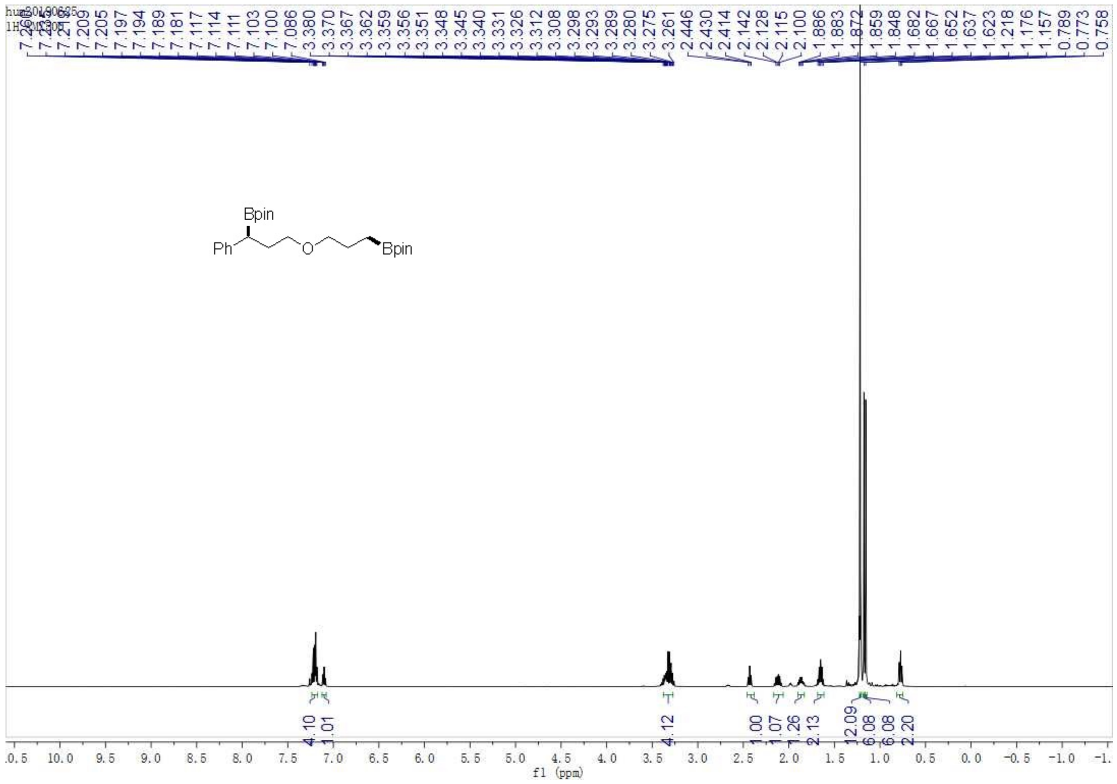

Supplementary Figure 113. ^{1}H NMR spectrum of compound **13’**.

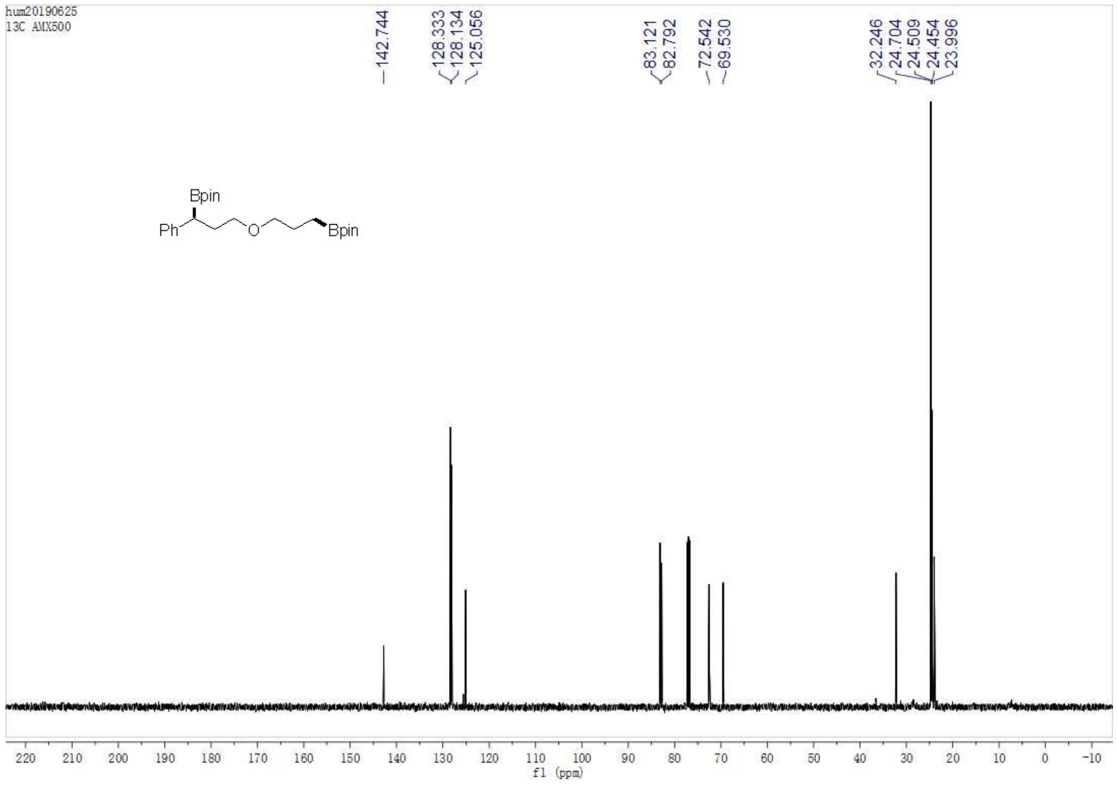

Supplementary Figure 114. $^{13}C\{^{1}H\}$ NMR spectrum of compound **13**.

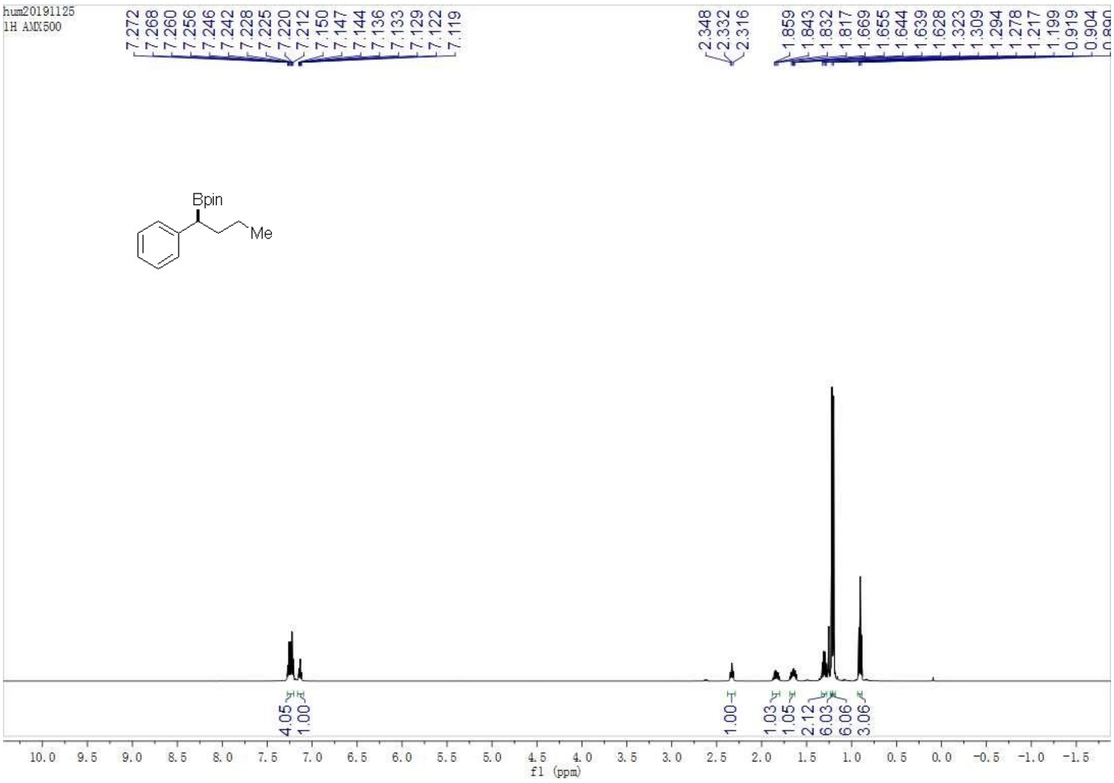

Supplementary Figure 115. ^{1}H NMR spectrum of compound **15**.

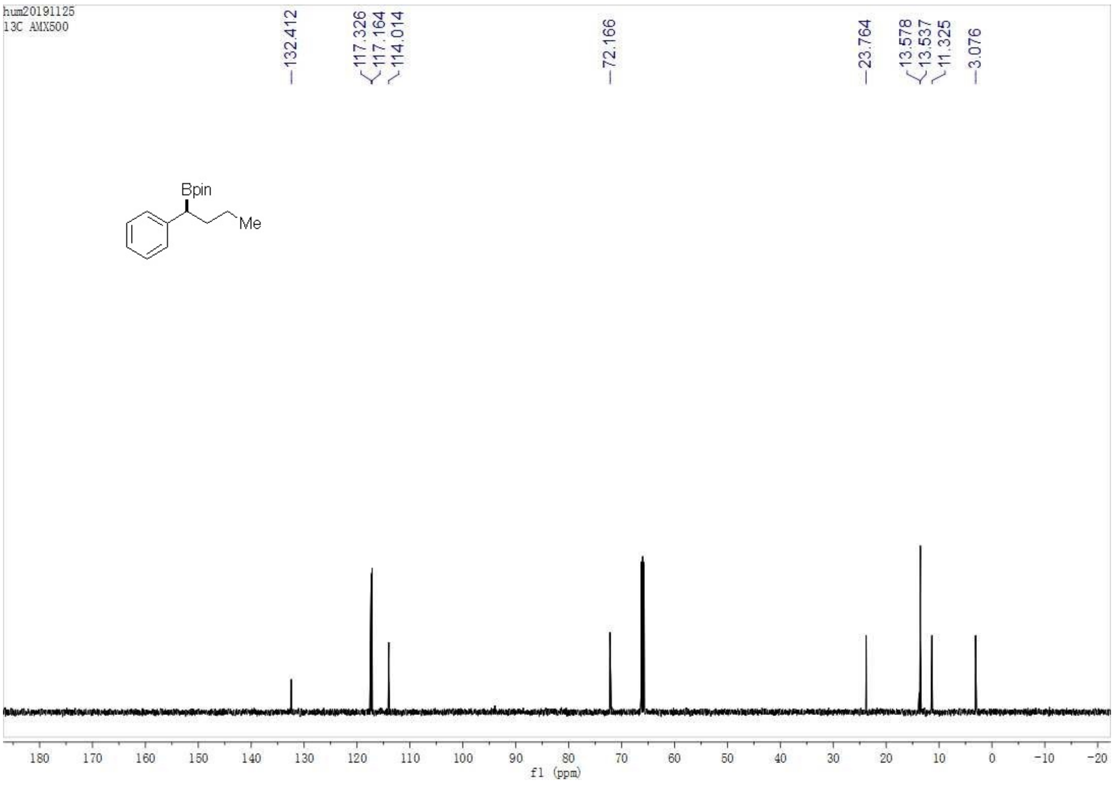

Supplementary Figure 116. ^{13}C{^{1}H} NMR spectrum of compound **15**.

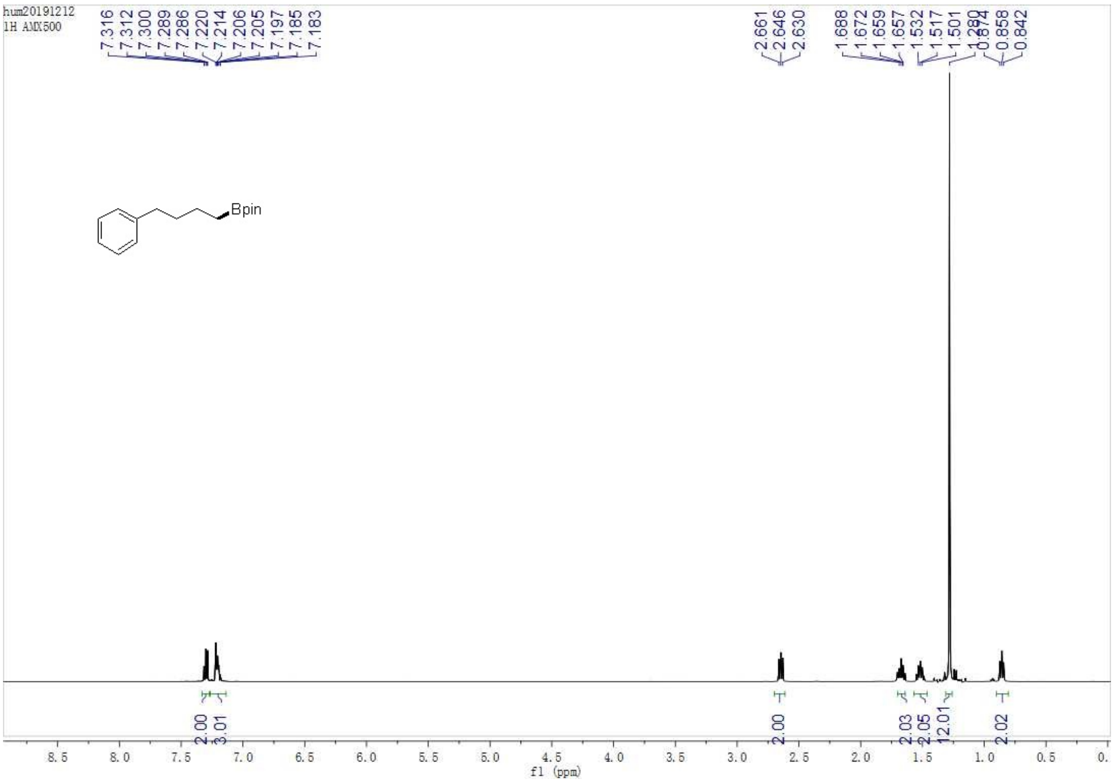

Supplementary Figure 117. ^{1}H NMR spectrum of compound **16**.

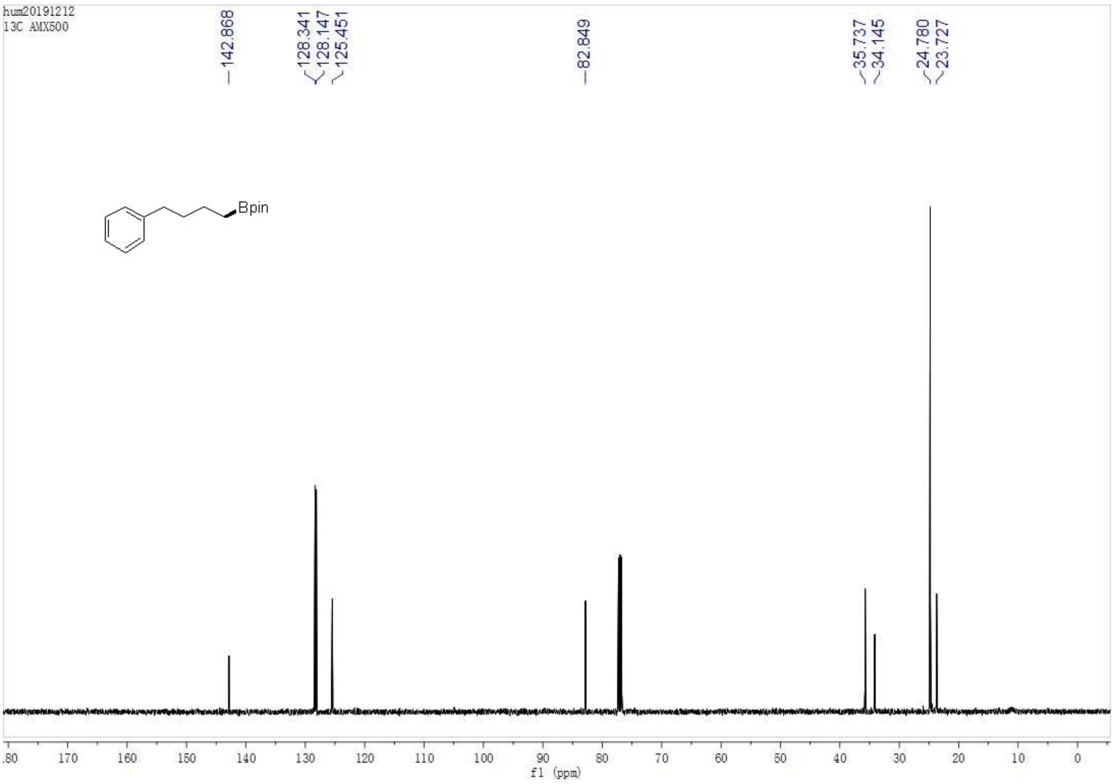

Supplementary Figure 118. $^{13}C\{^{1}H\}$ NMR spectrum of compound **16**.

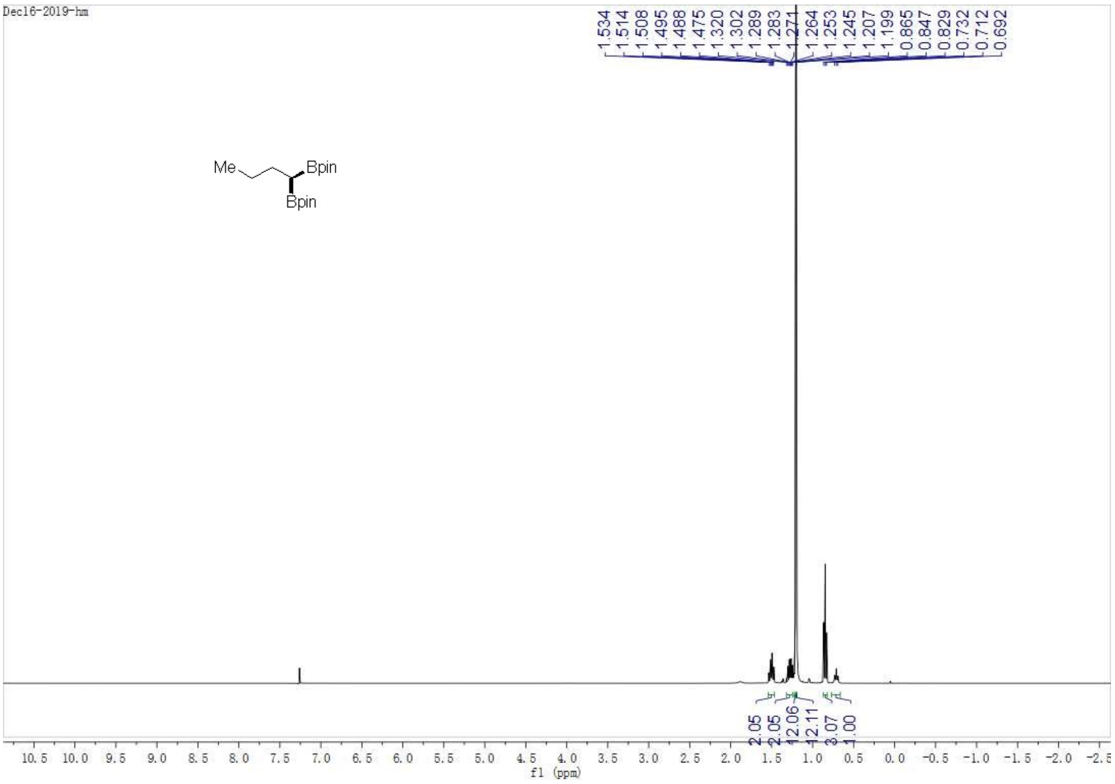

Supplementary Figure 119. ^{1}H NMR spectrum of compound **20**.

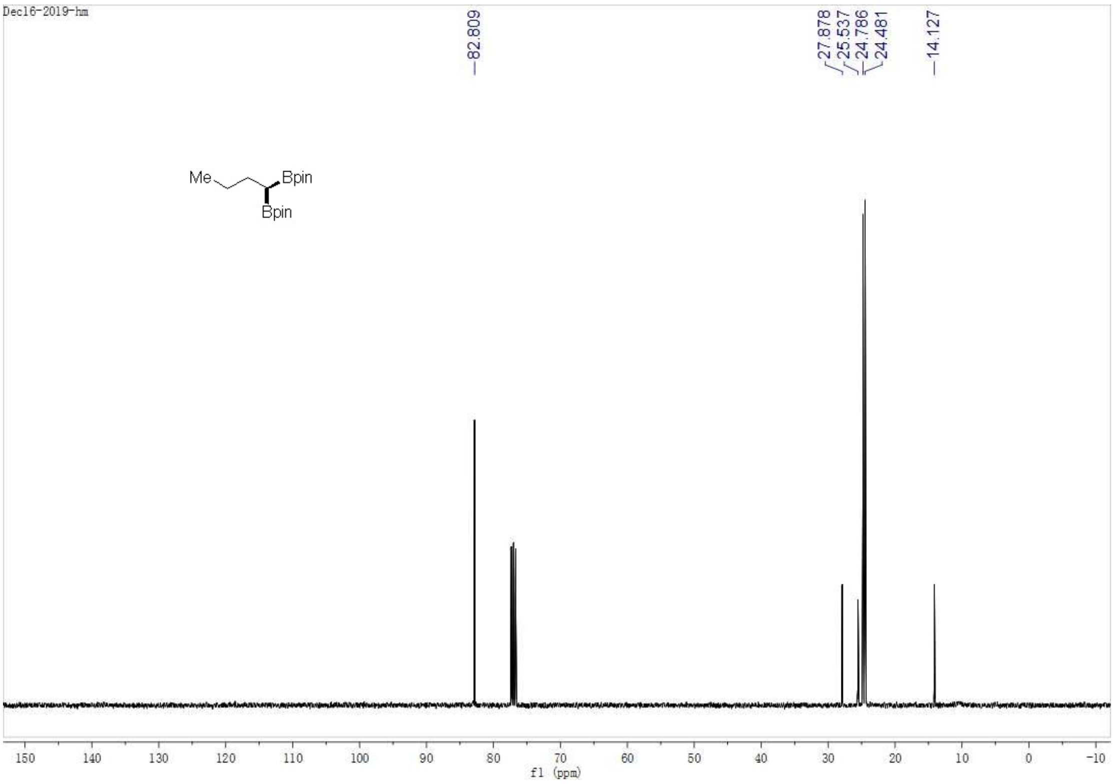

Supplementary Figure 120. $^{13}C\{^{1}H\}$ NMR spectrum of compound **20**.

Supplementary Reference

(1) Yu, S., Wu, C. & Ge, S. Cobalt-catalyzed asymmetric hydroboration/cyclization of 1,6-enynes with pinacolborane. *J. Am. Chem. Soc.* **139**, 6526-6529 (2017).

(2) Sharma, R. K. & RajanBabu, T. V. Asymmetric hydrovinylation of unactivated linear 1,3-dienes. *J. Am. Chem. Soc.* **132**, 3295-3297 (2010).

(3) Zumbansen, K., Dohring, A. & List, B. Morpholinium trifluoroacetate-catalyzed aldol condensation of acetone with both aromatic and aliphatic aldehydes. *Adv. Synth. Catal.* **352**, 1135-1138 (2010).

(4) Jing, S. M., Balasanthiran, V., Pagar, V., Gallucci, J. C. & RajanBabu, T. V. Catalytic enantioselective hetero-dimerization of acrylates and 1,3-dienes. *J. Am. Chem. Soc.* **139**, 18034-18043 (2017).

(5) Chen, F., Zhang, Y., Yu, L. & Zhu, S. Enantioselective NiH/pmrox-catalyzed 1,2-reduction of α,β-unsaturated ketones. *Angew. Chem. Int. Ed.* **56**, 2022-2025 (2017).

(6) Duvvuri, K., Dewese, K. R., Parsutkar, M. M., Jing, S. M., Mehta, M. M., Gallucci, J. C. & RajanBabu, T. V. Cationic Co(I)-intermediates for hydrofunctionalization reactions: regio- and enantioselective cobalt-catalyzed 1,2-hydroboration of 1,3-dienes. *J. Am. Chem. Soc.* **141**, 7365-7375 (2019).

(7) Stephens, T. C. & Pattison, G. Transition-metal-free homologative cross-coupling of aldehydes and ketones with geminal bis(boron) compounds. *Org. Lett.* **19**, 3498-3501 (2017).

(8) a) Jo, W., Kim, J., Choi, S. & Cho, S. H. Transition-metal-free regioselective alkylation of pyridine N-oxides using 1,1-diborylalkanes as alkylating reagents. *Angew. Chem. Int. Ed.* **55**, 9690-9694 (2016). b) Hwang, C., Jo, W. & Cho, S. H.

Base-promoted, deborylative secondary alkylation of N-heteroaromatic N-oxides with internal gem-bis[(pinacolato)boryl]alkanes: a facile derivatization of 2,2′-bipyridyl analogues. *Chem. Commun.* **53**, 7573-7576 (2017).

(9) Hong, K., Liu, X. & Morken, J. P. Simple access to elusive α-boryl carbanions and their alkylation: an umpolung construction for organic synthesis. *J. Am. Chem. Soc.* **136**, 10581-10584 (2014).

(10) Endo, K., Ohkubo, T., Hirokami, M. & Shibata, T. Chemoselective and regiospecific suzuki coupling on a multisubstituted sp^3-carbon in 1,1-diborylalkanes at room temperature. *J. Am. Chem. Soc.* **132**, 11033-11035 (2010).

(11) Maza, R. J., Davenport, E., Miralles, N., Carbó, J. J. & Fernández, E. *Org. Lett.* **21**, 2251-2255 (2019).

(12) Li, R., Ju, C.-W. & Zhao, D. Rhodium(iii) vs. cobalt(iii): a mechanistically distinct three-component C–H bond addition cascade using a Cp^*Rh^{III} catalyst. *Chem. Commun.* **55**, 695-698 (2019).
